# Supplementary figures and images for: Tissue-selective COPII modulator SEC16B aggravates cardiovascular disease by promoting lipid export (part 1 of 3)
Source: EMBO J. 2026 Apr 24;45(11):3731–62. doi: 10.1038/s44318-026-00754-8 (PMC13226660; doi:10.1038/s44318-026-00754-8)

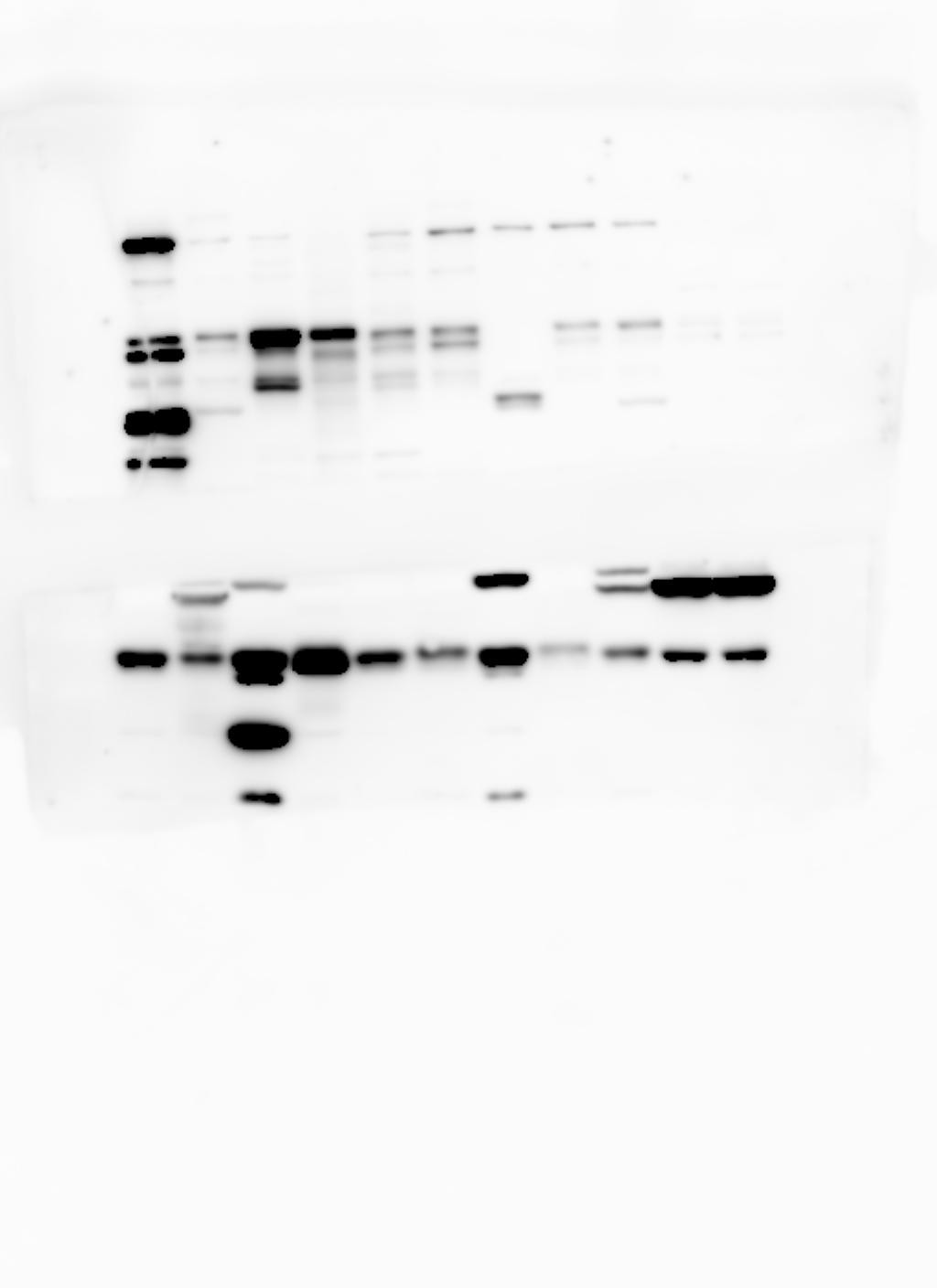

Supplement: Supplementary file 4 — Source data Fig. 1 [file 44318_2026_754_MOESM4_ESM.zip › Figure 1/1E/1E_western_SEC13.jpg]

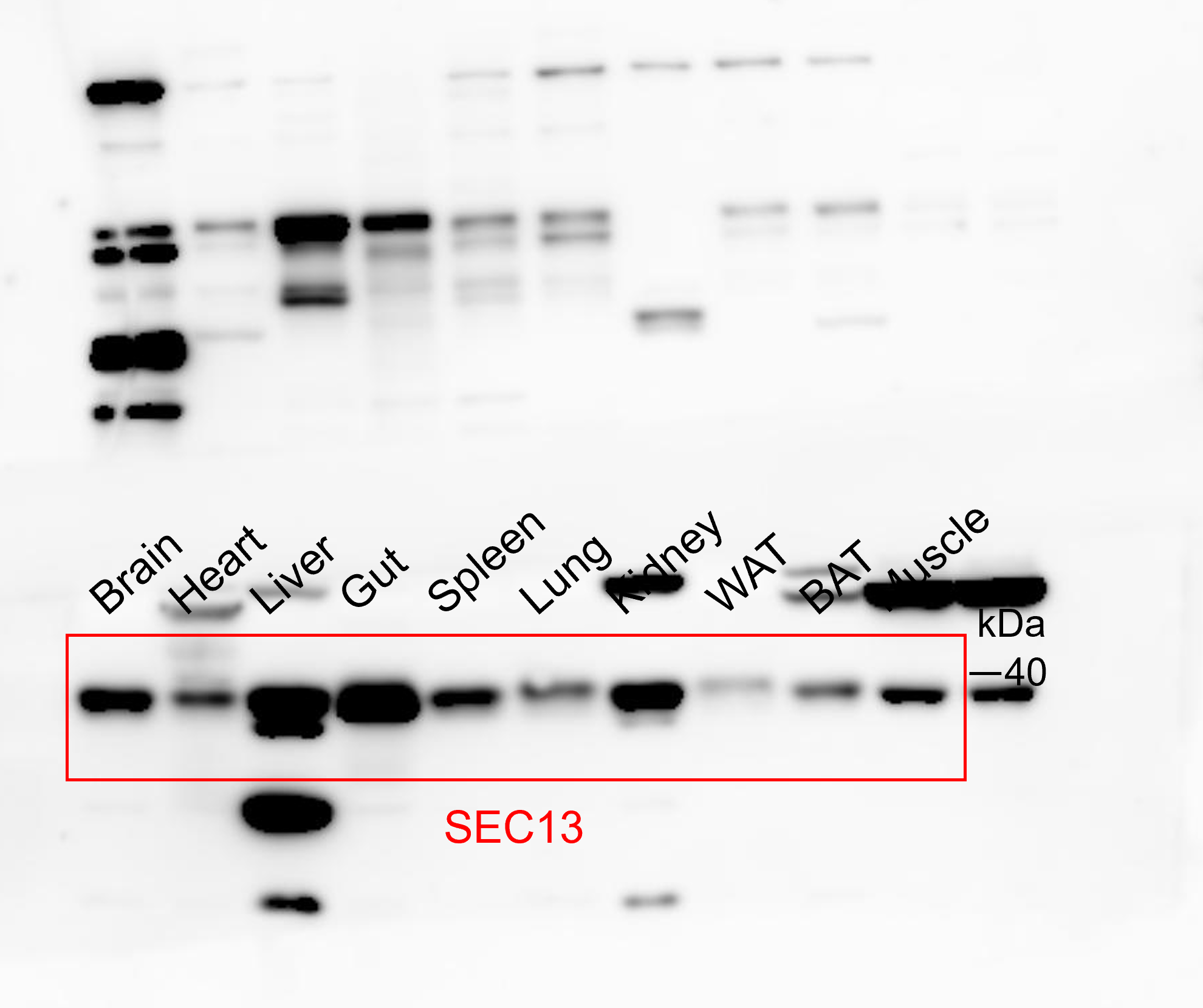

Supplement: Supplementary file 4 — Source data Fig. 1 [file 44318_2026_754_MOESM4_ESM.zip › Figure 1/1E/1E_western_SEC13_label.tif]

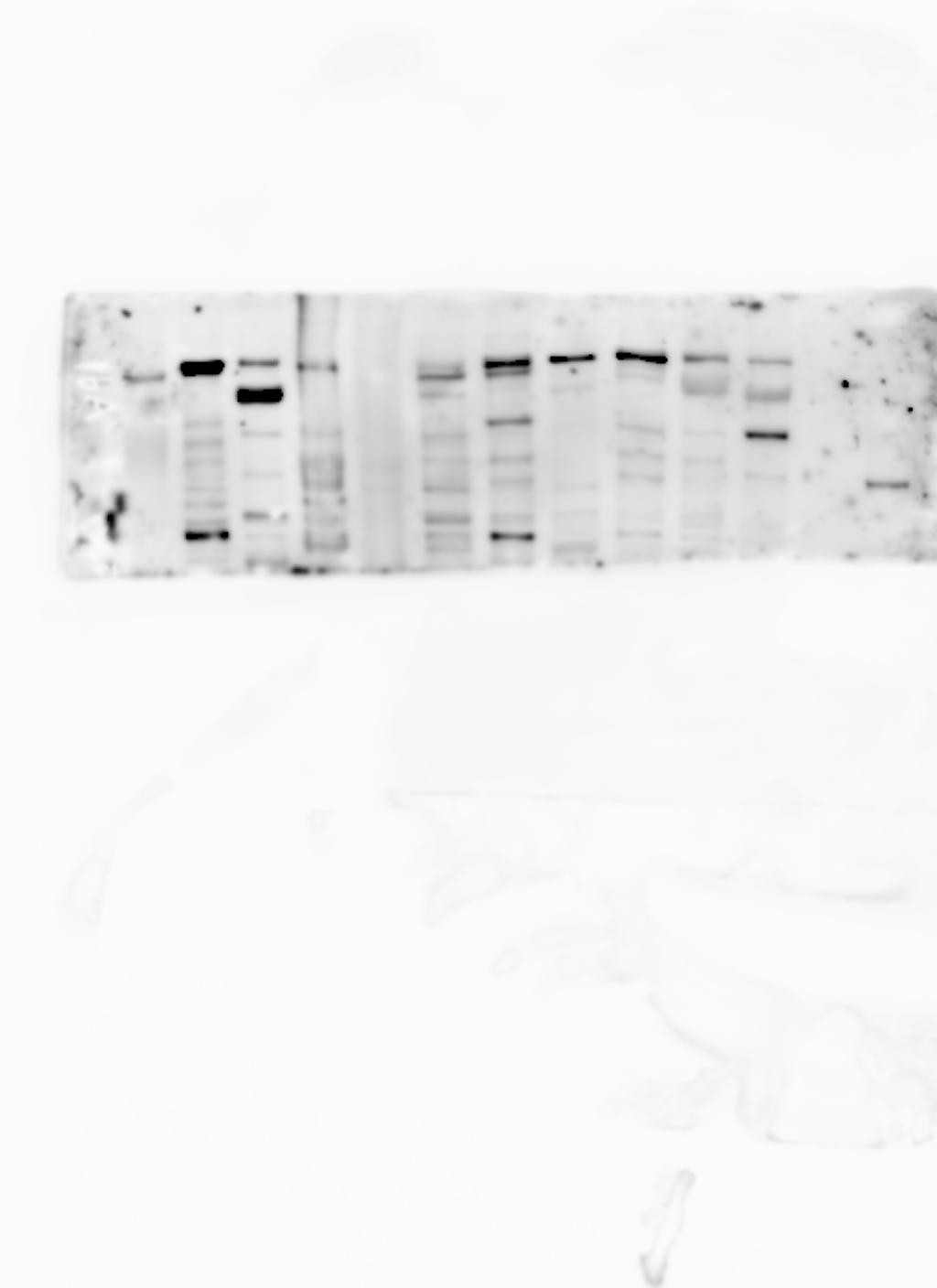

Supplement: Supplementary file 4 — Source data Fig. 1 [file 44318_2026_754_MOESM4_ESM.zip › Figure 1/1E/1E_western_SEC16A.jpg]

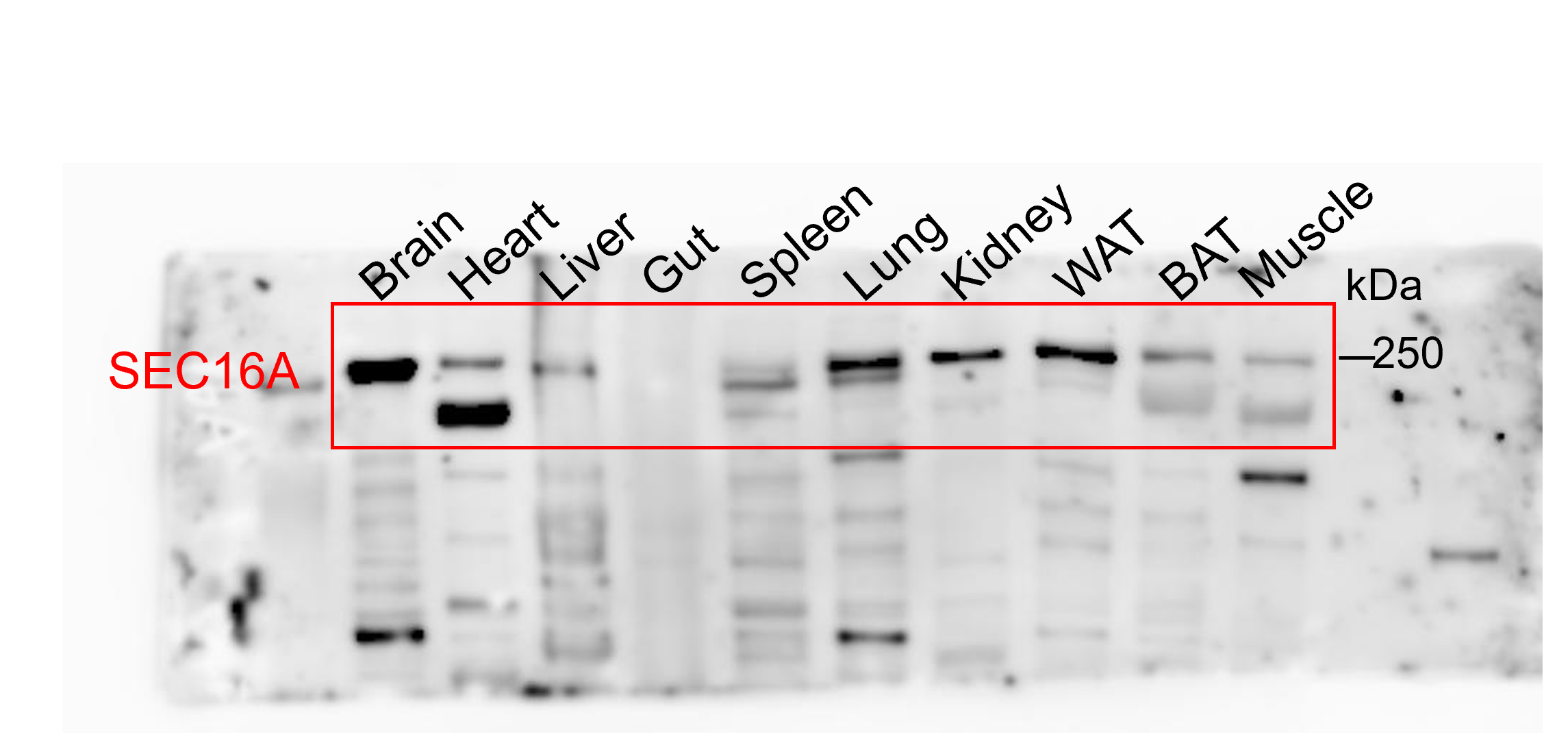

Supplement: Supplementary file 4 — Source data Fig. 1 [file 44318_2026_754_MOESM4_ESM.zip › Figure 1/1E/1E_western_SEC16A_label.tif]

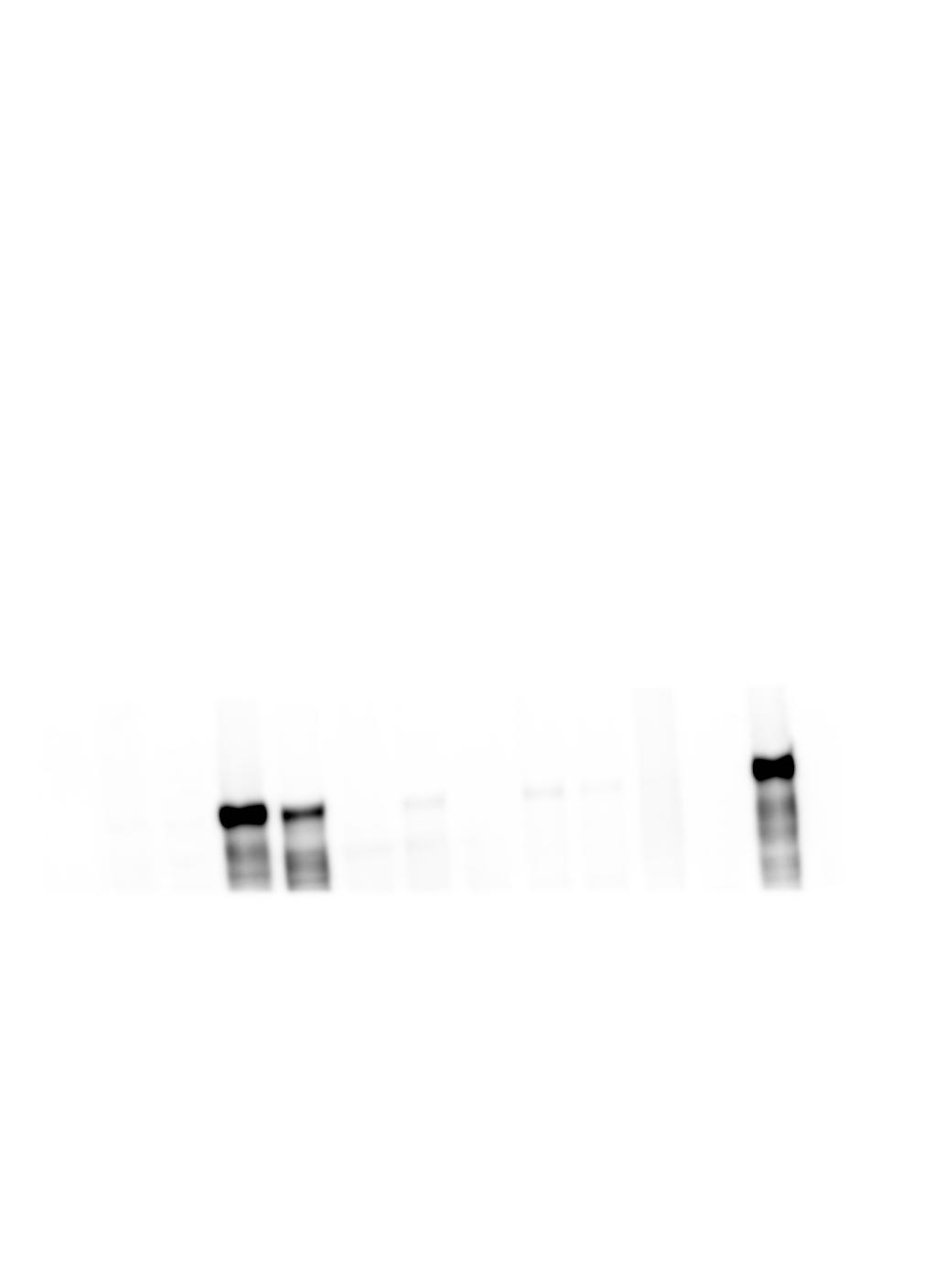

Supplement: Supplementary file 4 — Source data Fig. 1 [file 44318_2026_754_MOESM4_ESM.zip › Figure 1/1E/1E_western_SEC16B.jpg]

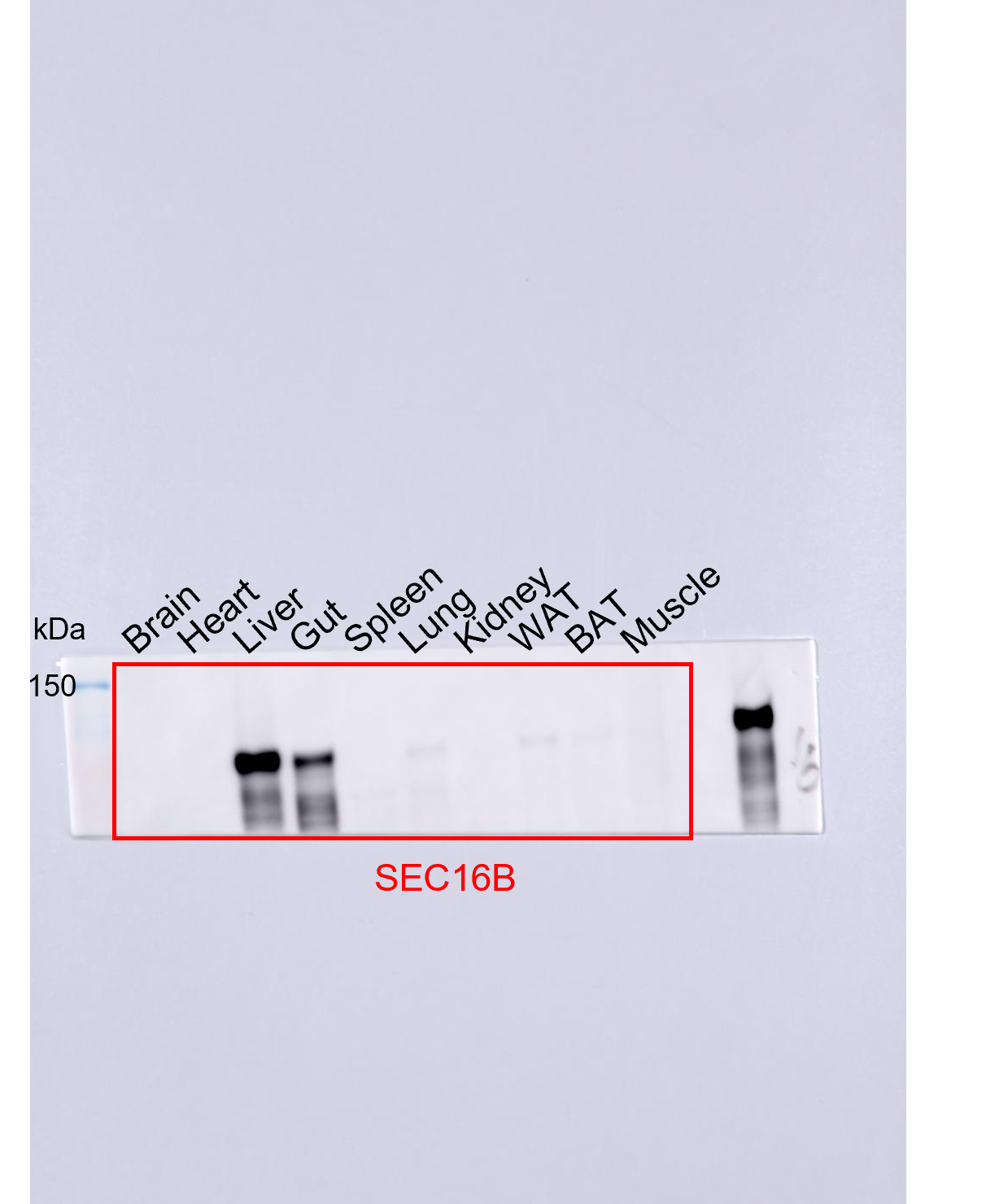

Supplement: Supplementary file 4 — Source data Fig. 1 [file 44318_2026_754_MOESM4_ESM.zip › Figure 1/1E/1E_western_SEC16B_label.tif]

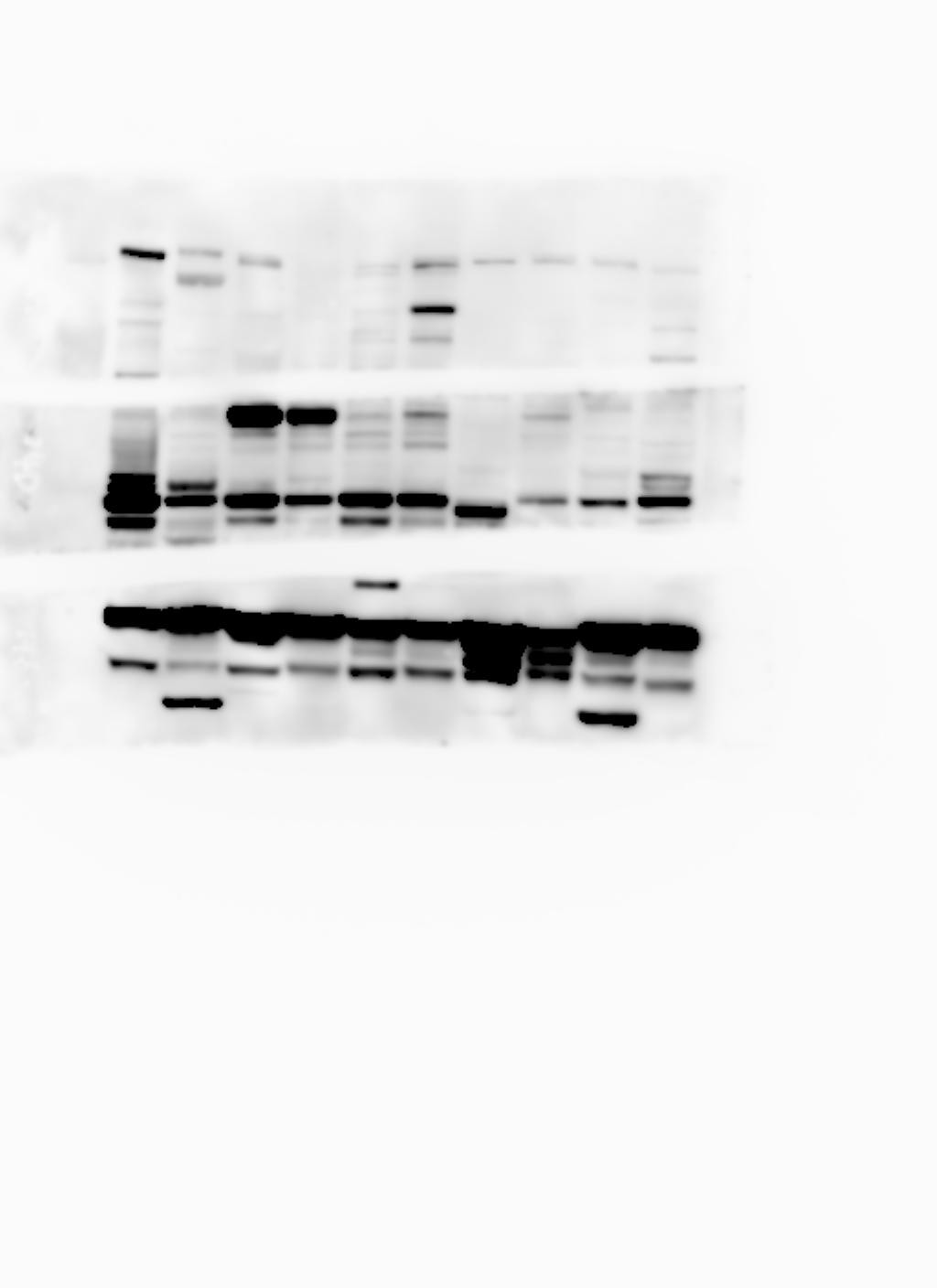

Supplement: Supplementary file 4 — Source data Fig. 1 [file 44318_2026_754_MOESM4_ESM.zip › Figure 1/1E/1E_western_SEC24D.jpg]

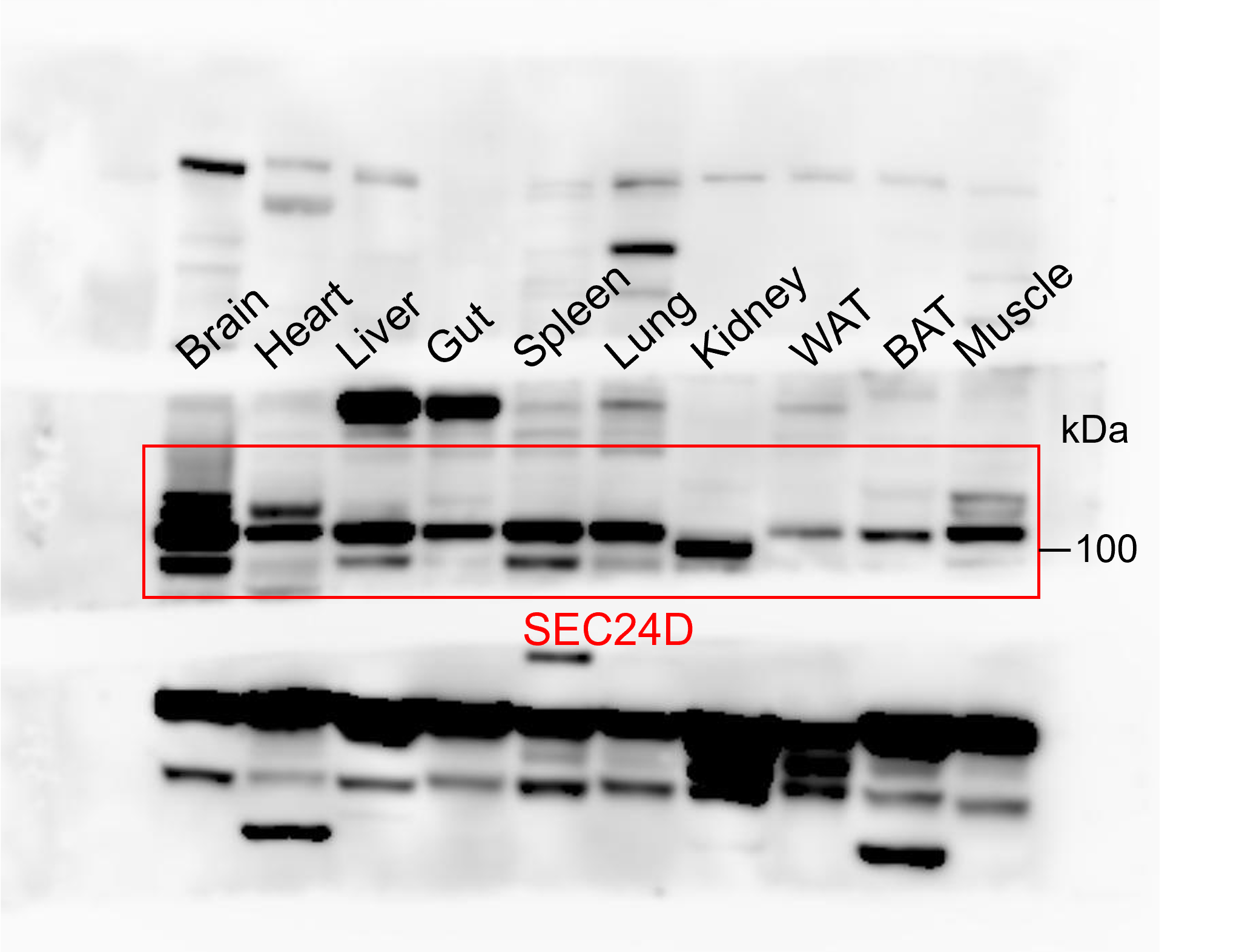

Supplement: Supplementary file 4 — Source data Fig. 1 [file 44318_2026_754_MOESM4_ESM.zip › Figure 1/1E/1E_western_SEC24D_label.tif]

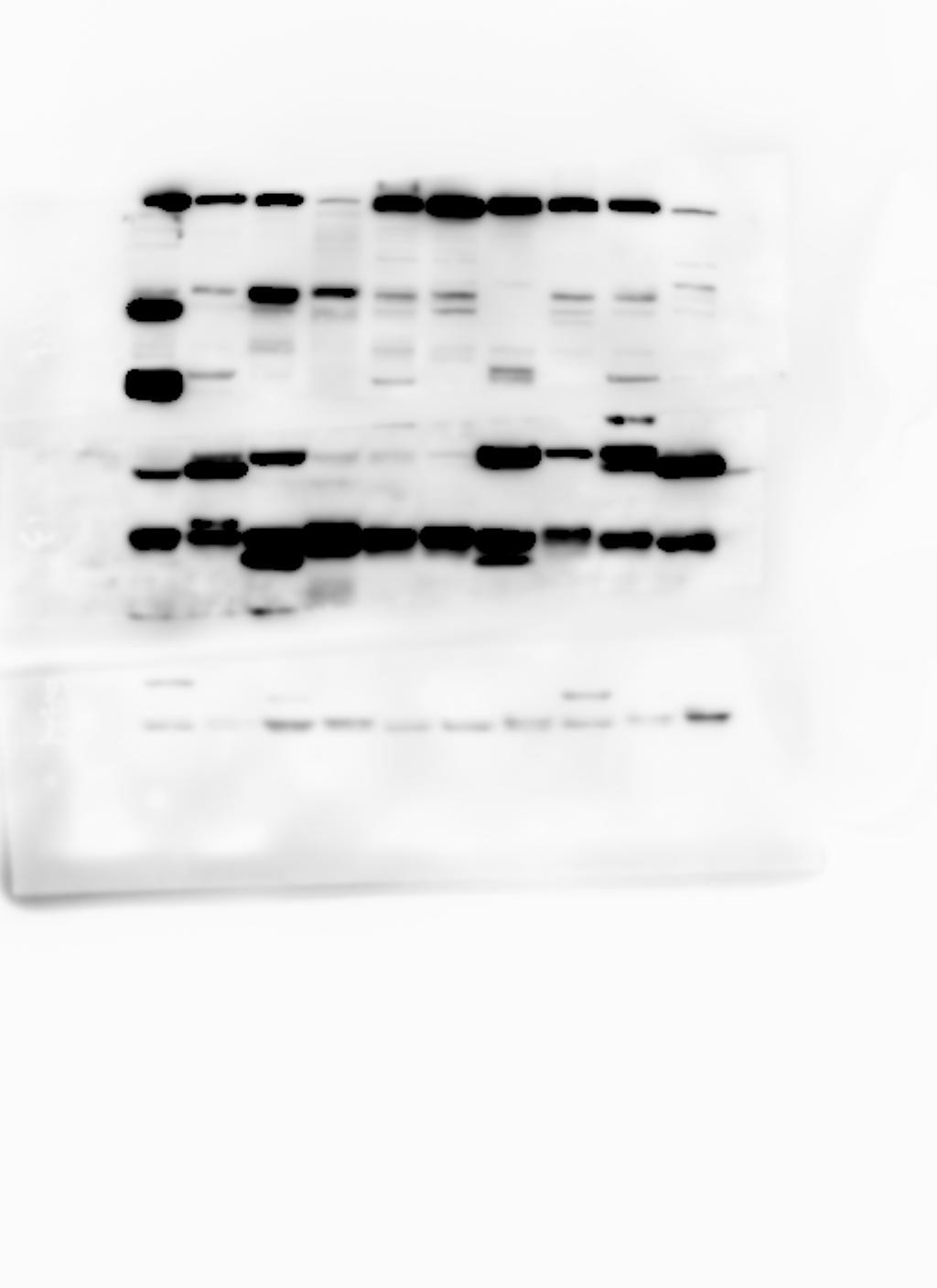

Supplement: Supplementary file 4 — Source data Fig. 1 [file 44318_2026_754_MOESM4_ESM.zip › Figure 1/1E/1E_western_SEC31A.jpg]

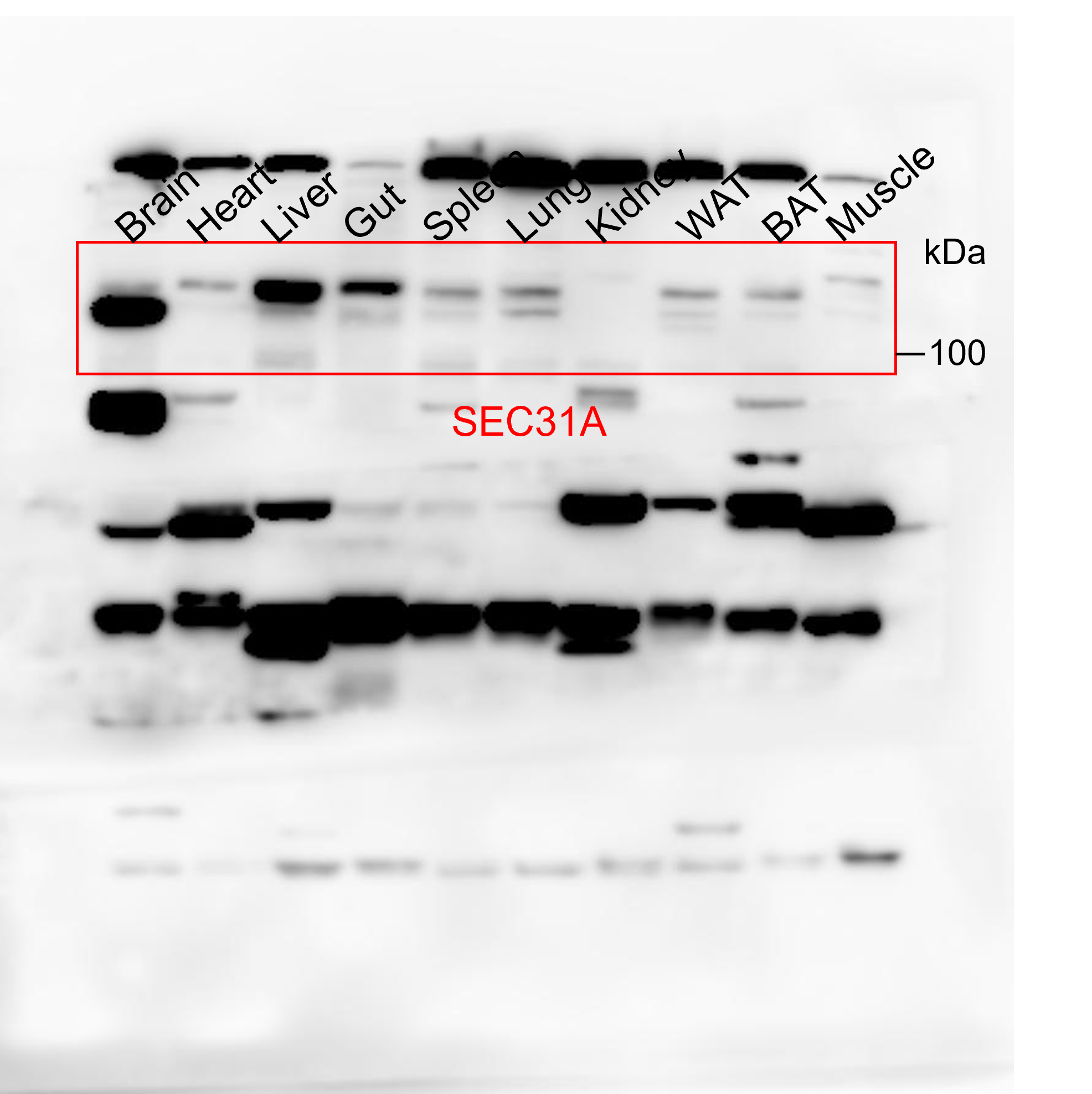

Supplement: Supplementary file 4 — Source data Fig. 1 [file 44318_2026_754_MOESM4_ESM.zip › Figure 1/1E/1E_western_SEC31A_label.tif]

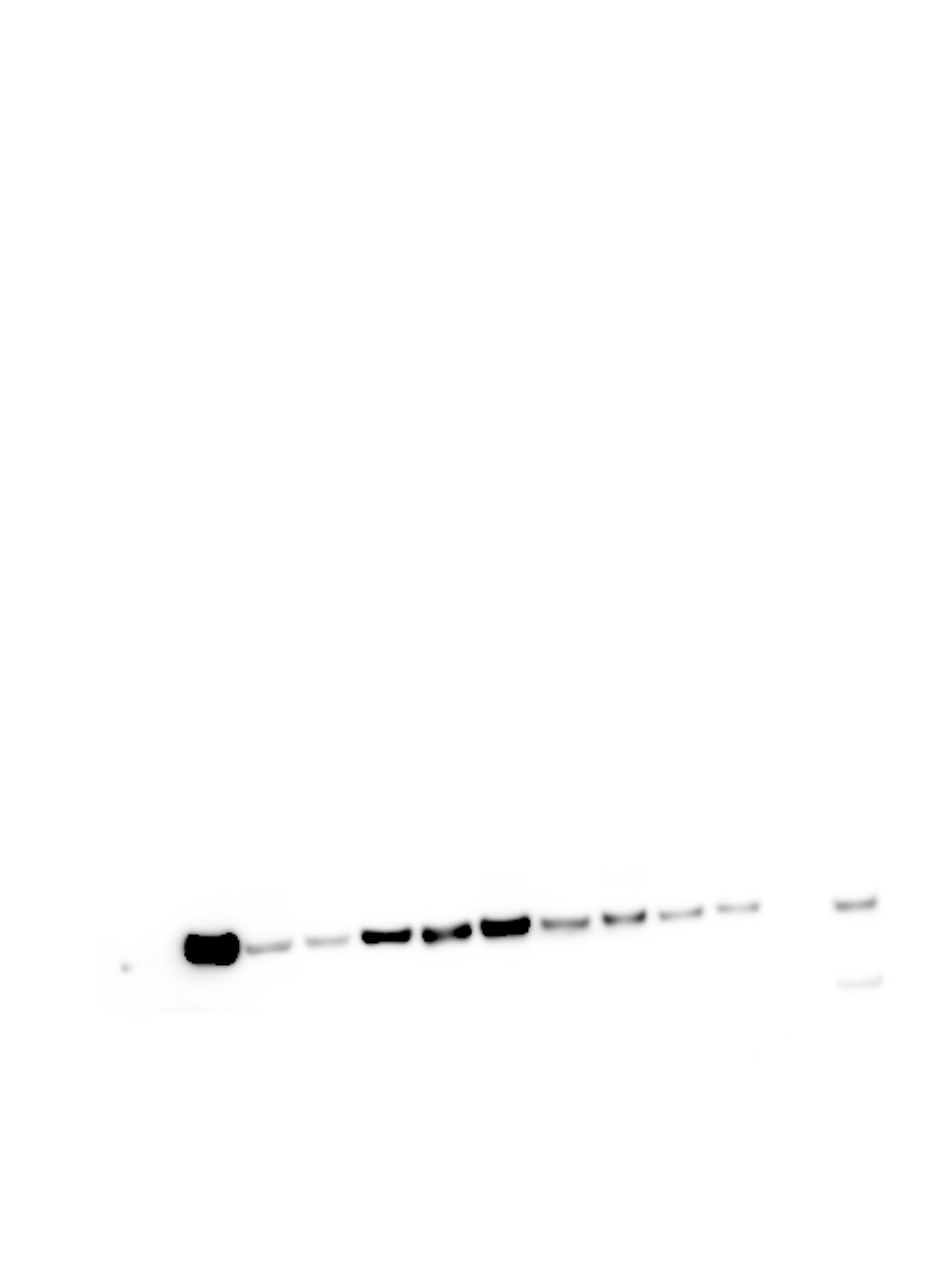

Supplement: Supplementary file 4 — Source data Fig. 1 [file 44318_2026_754_MOESM4_ESM.zip › Figure 1/1E/1E_western_Tub.jpg]

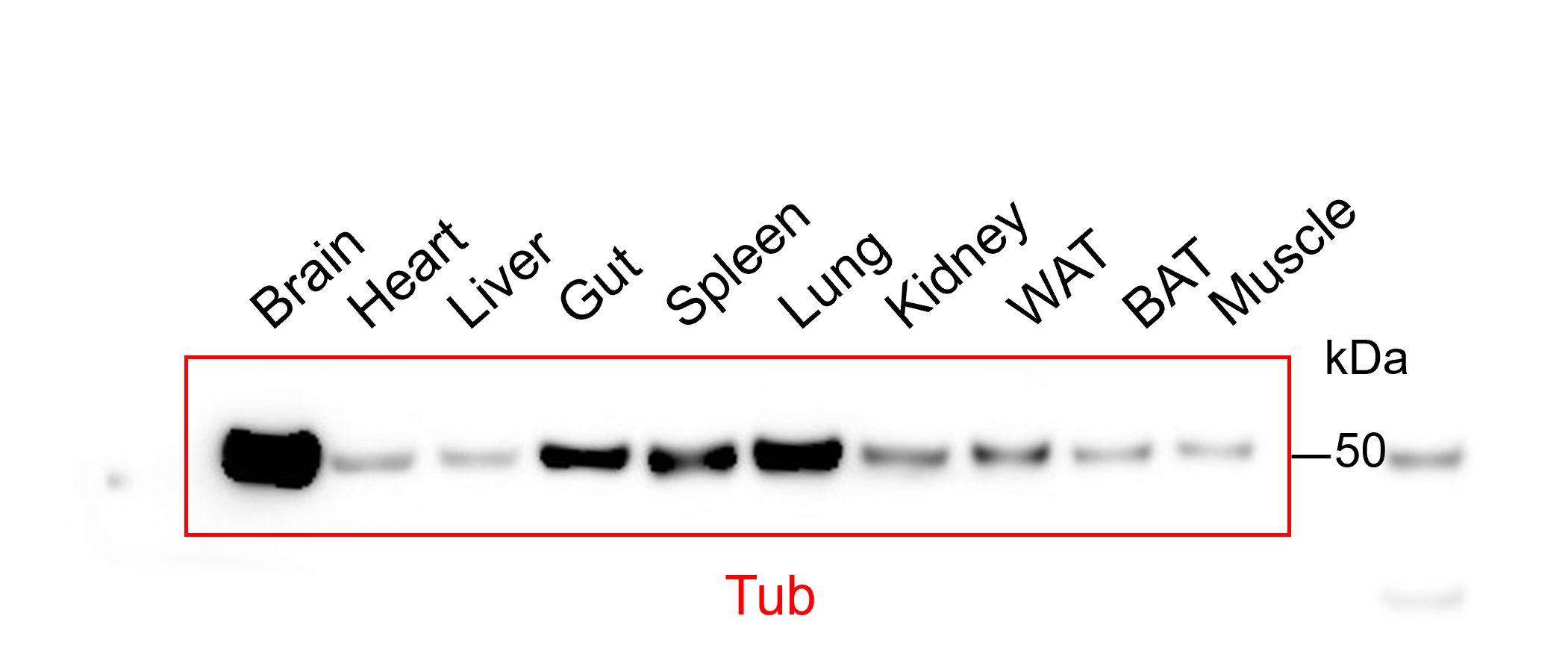

Supplement: Supplementary file 4 — Source data Fig. 1 [file 44318_2026_754_MOESM4_ESM.zip › Figure 1/1E/1E_western_Tub_label.tif]

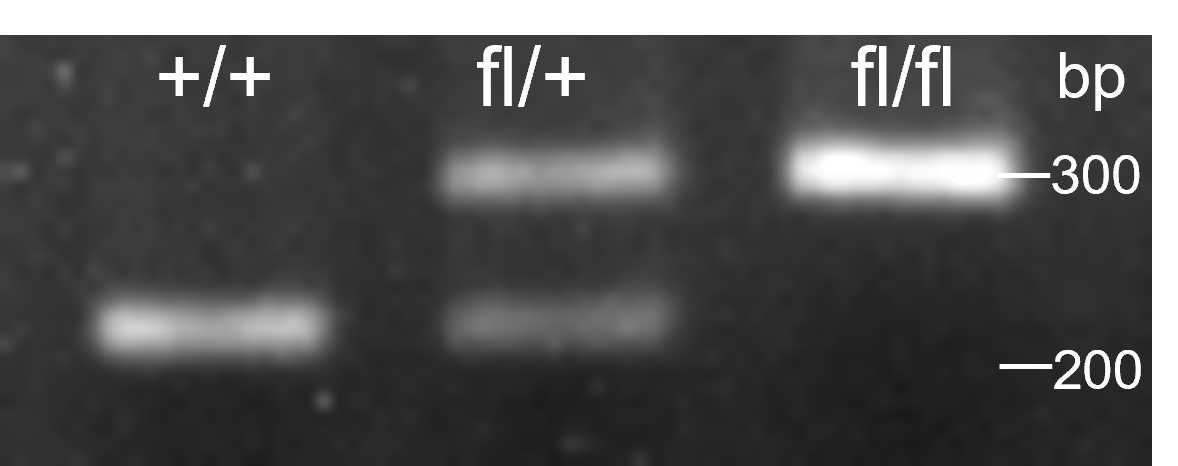

Supplement: Supplementary file 5 — Source data Fig. 2 [file 44318_2026_754_MOESM5_ESM.zip › Figure 2/2A/2A_gel_Genotyping.tif]

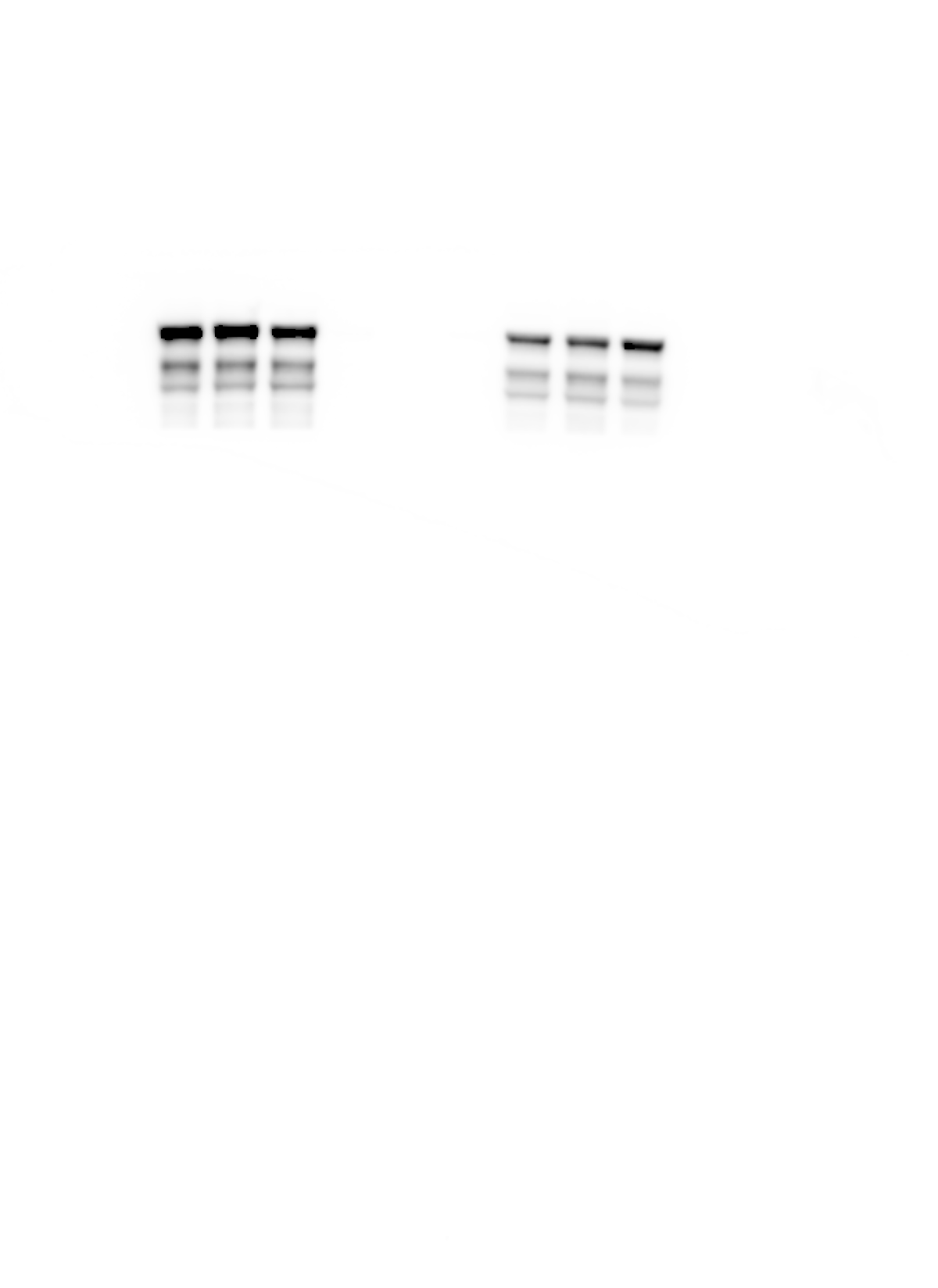

Supplement: Supplementary file 5 — Source data Fig. 2 [file 44318_2026_754_MOESM5_ESM.zip › Figure 2/2C/2C_western_SEC16B.tif]

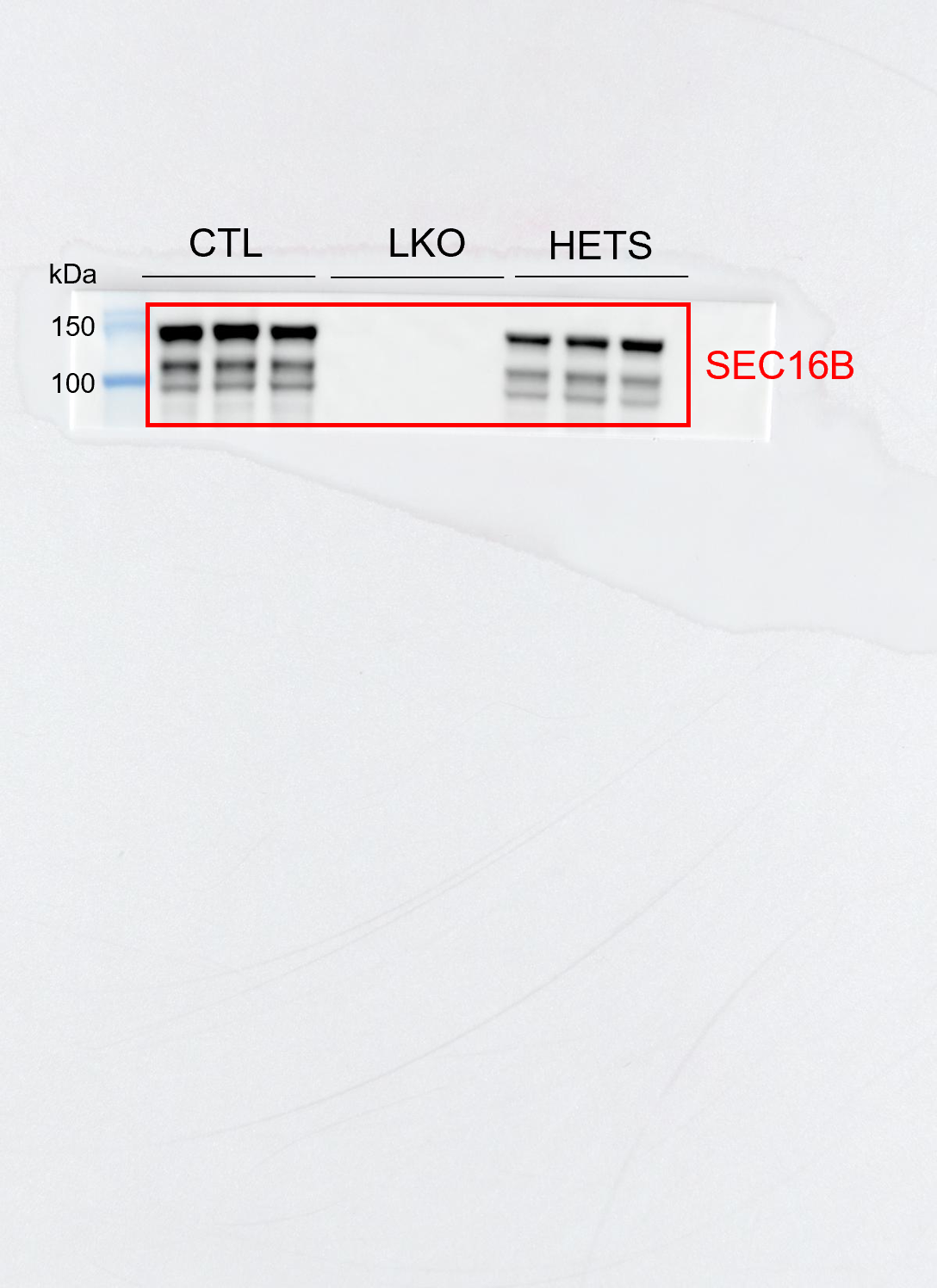

Supplement: Supplementary file 5 — Source data Fig. 2 [file 44318_2026_754_MOESM5_ESM.zip › Figure 2/2C/2C_western_SEC16B_label.tif]

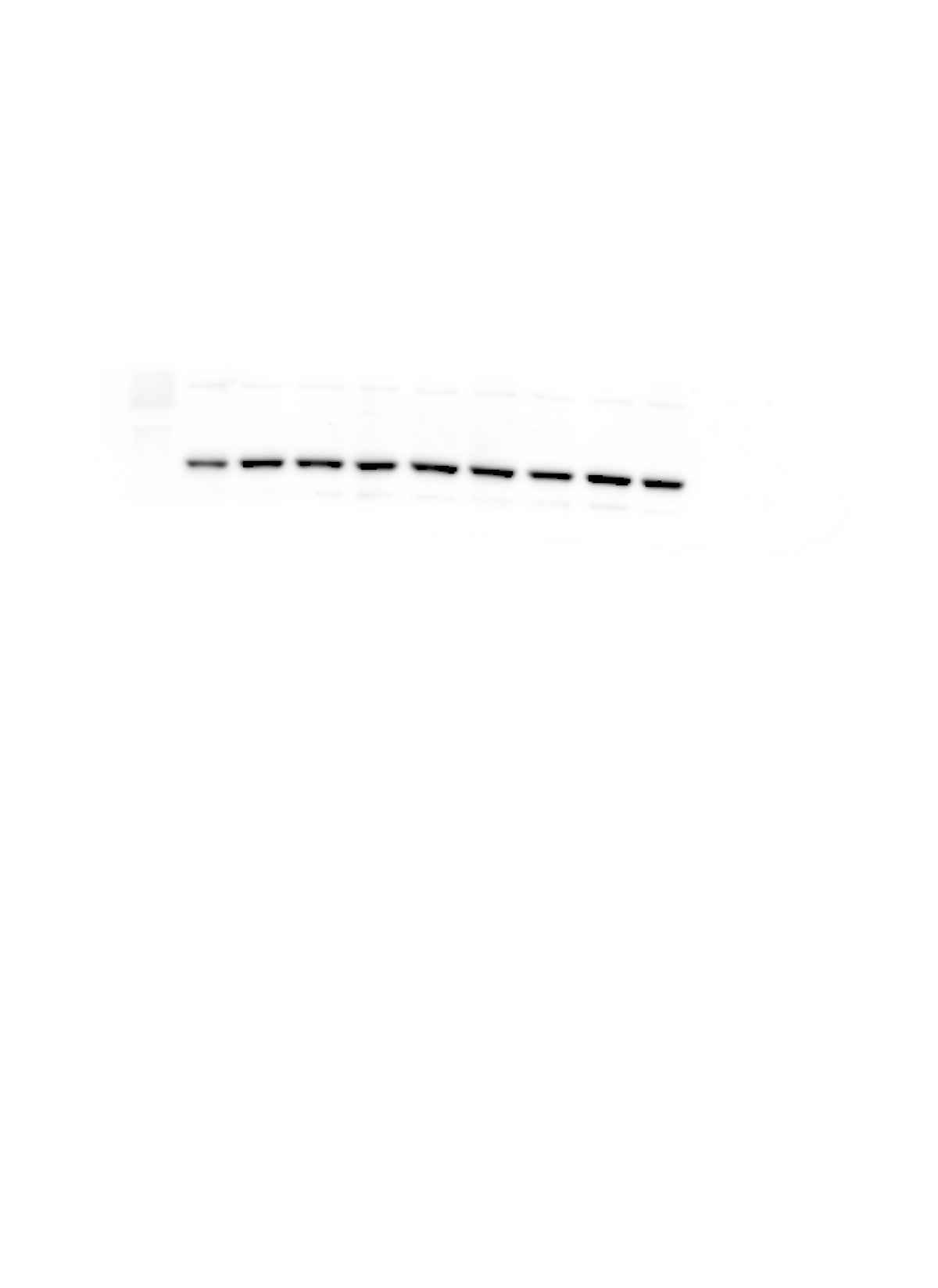

Supplement: Supplementary file 5 — Source data Fig. 2 [file 44318_2026_754_MOESM5_ESM.zip › Figure 2/2C/2C_western_Tub.tif]

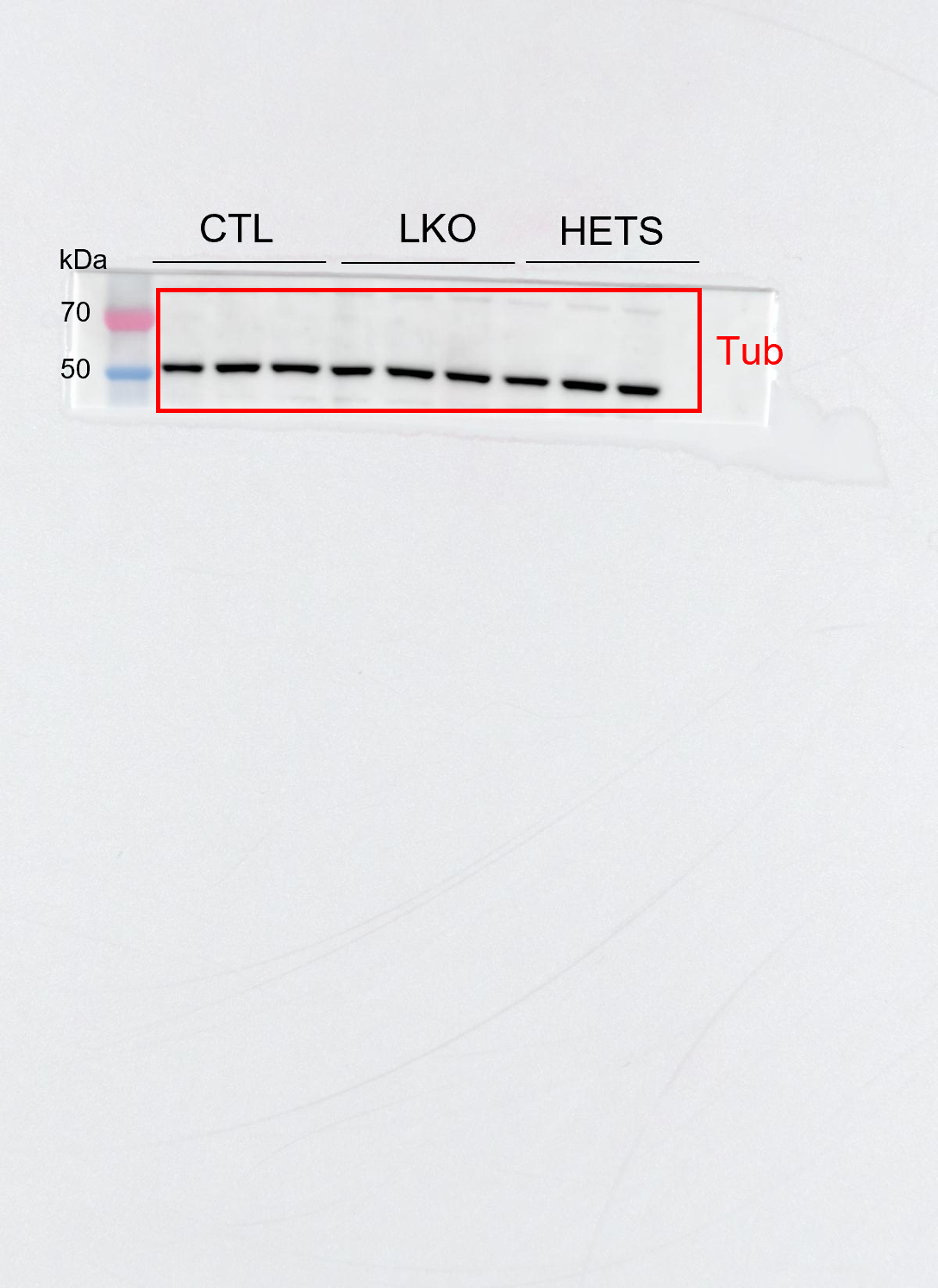

Supplement: Supplementary file 5 — Source data Fig. 2 [file 44318_2026_754_MOESM5_ESM.zip › Figure 2/2C/2C_western_Tub_label.tif]

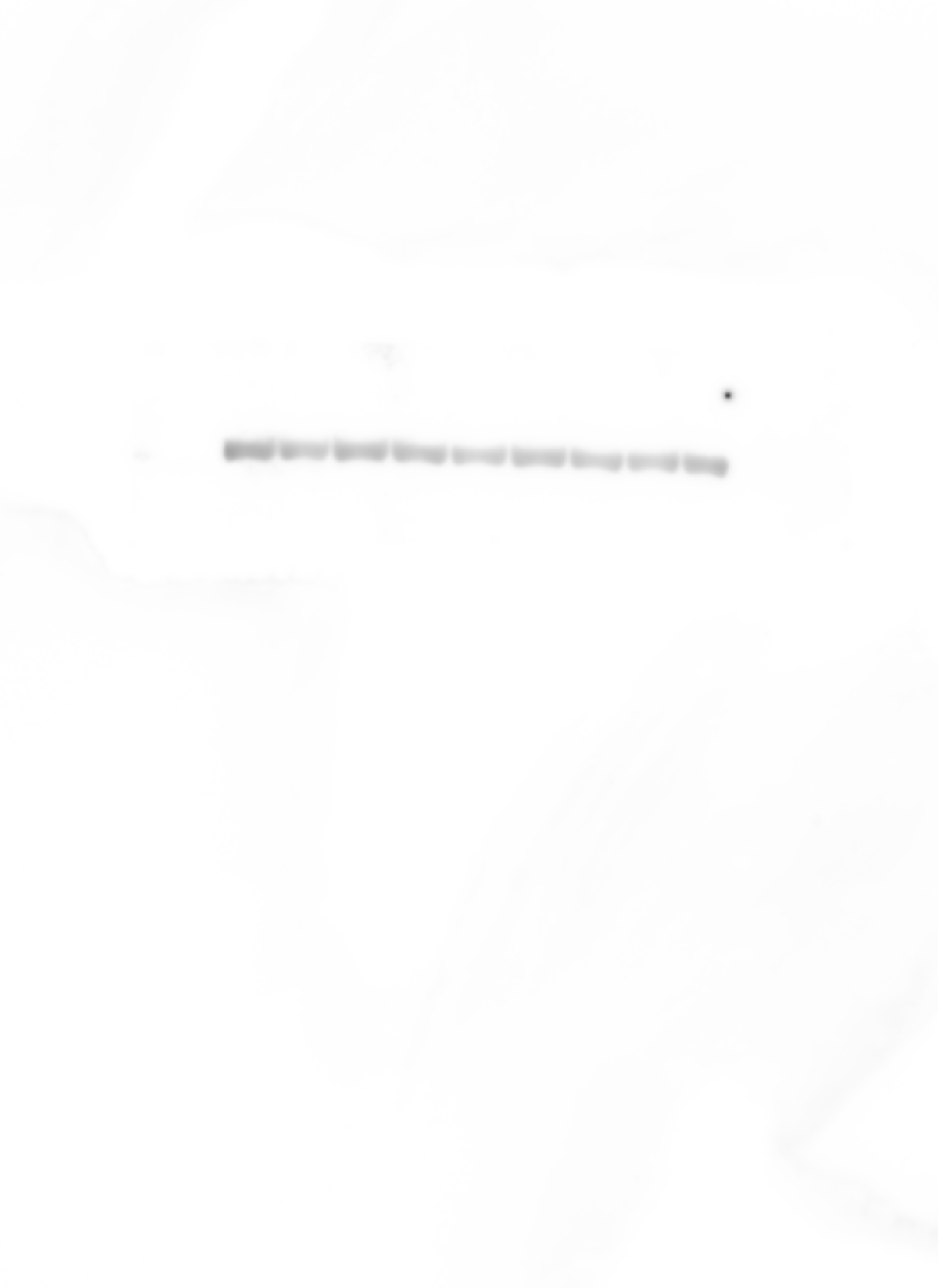

Supplement: Supplementary file 5 — Source data Fig. 2 [file 44318_2026_754_MOESM5_ESM.zip › Figure 2/2J-K/2J_western_ALB.tif]

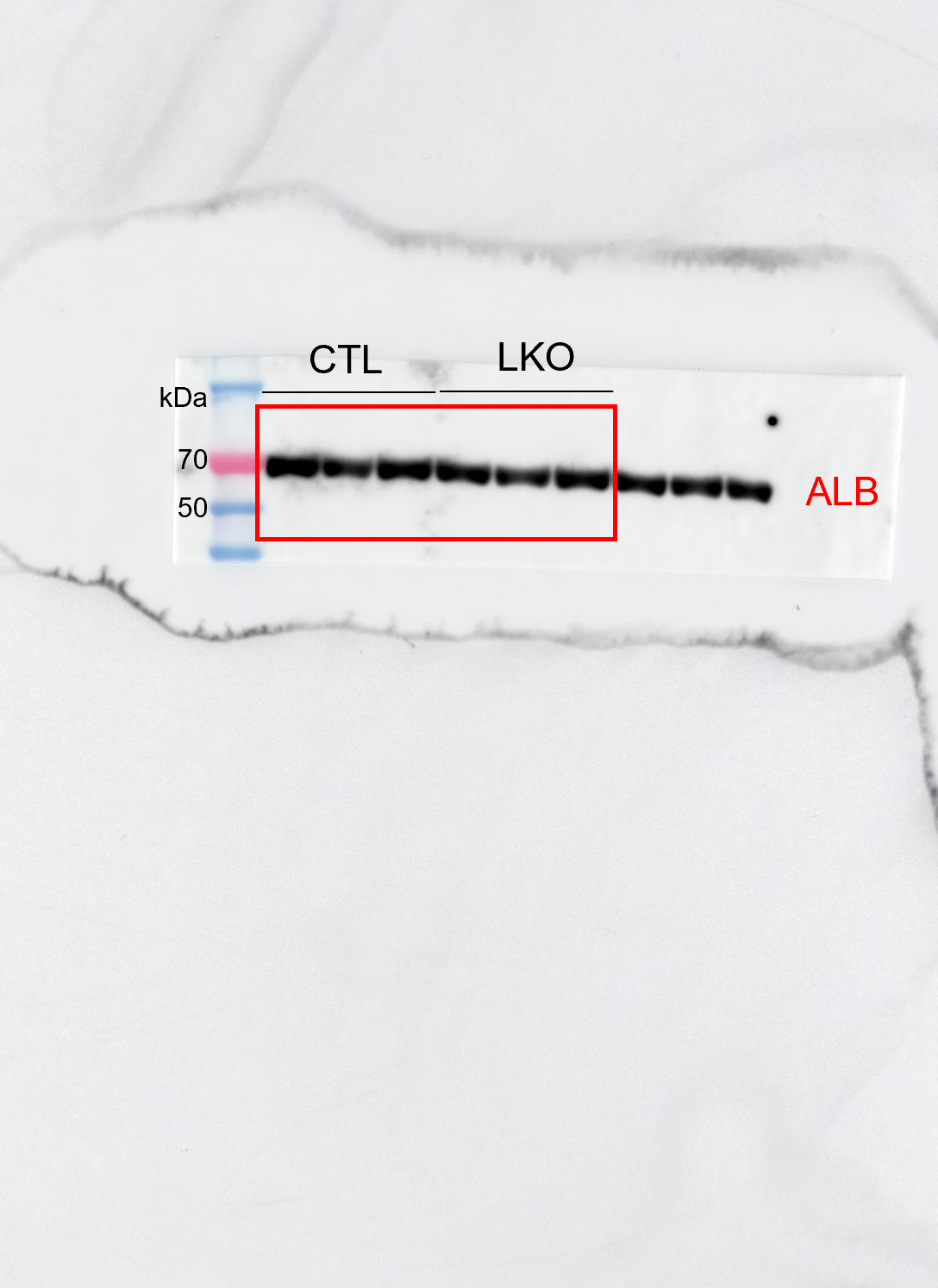

Supplement: Supplementary file 5 — Source data Fig. 2 [file 44318_2026_754_MOESM5_ESM.zip › Figure 2/2J-K/2J_western_ALB_label.tif]

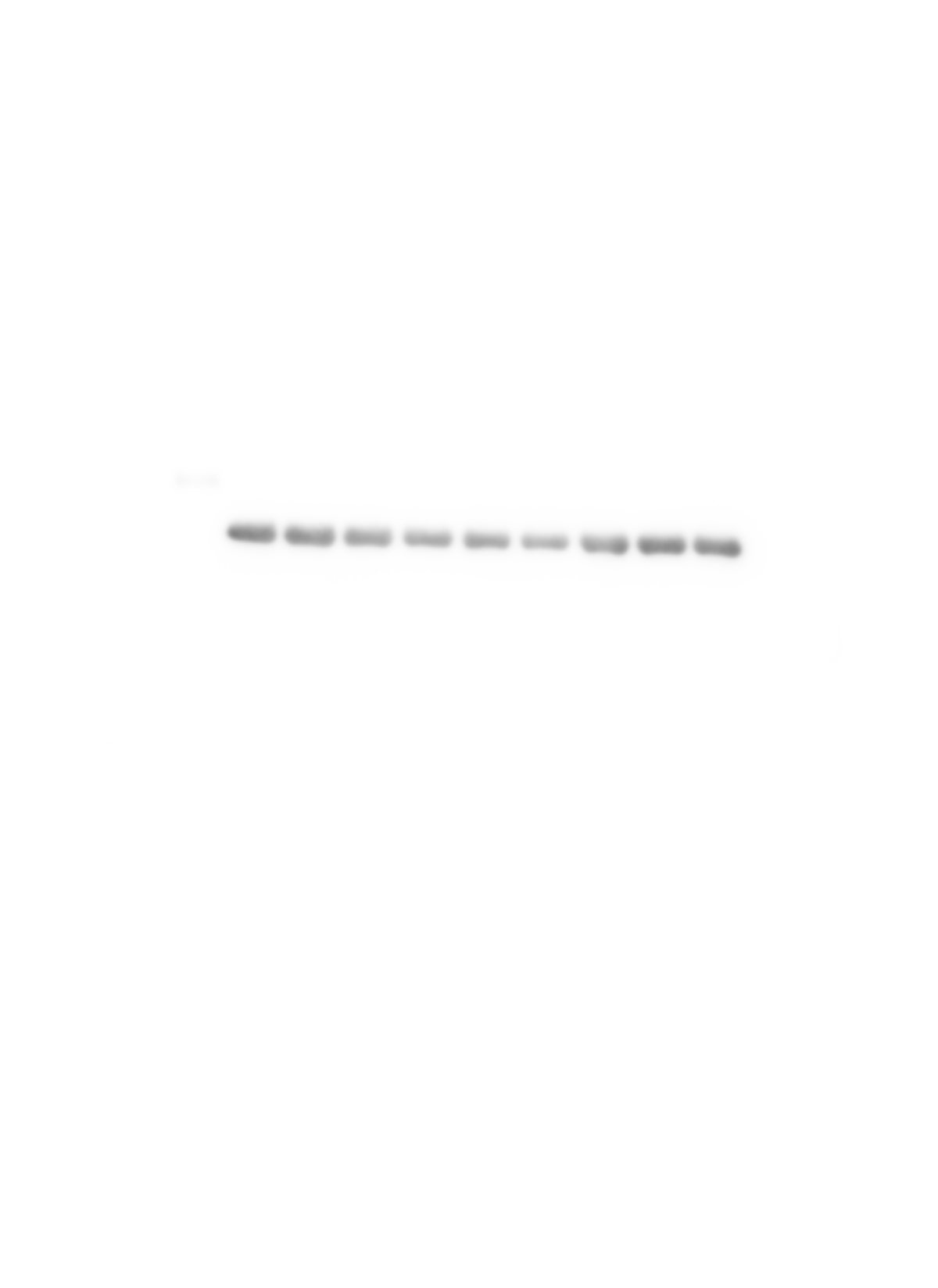

Supplement: Supplementary file 5 — Source data Fig. 2 [file 44318_2026_754_MOESM5_ESM.zip › Figure 2/2J-K/2J_western_APOA1.tif]

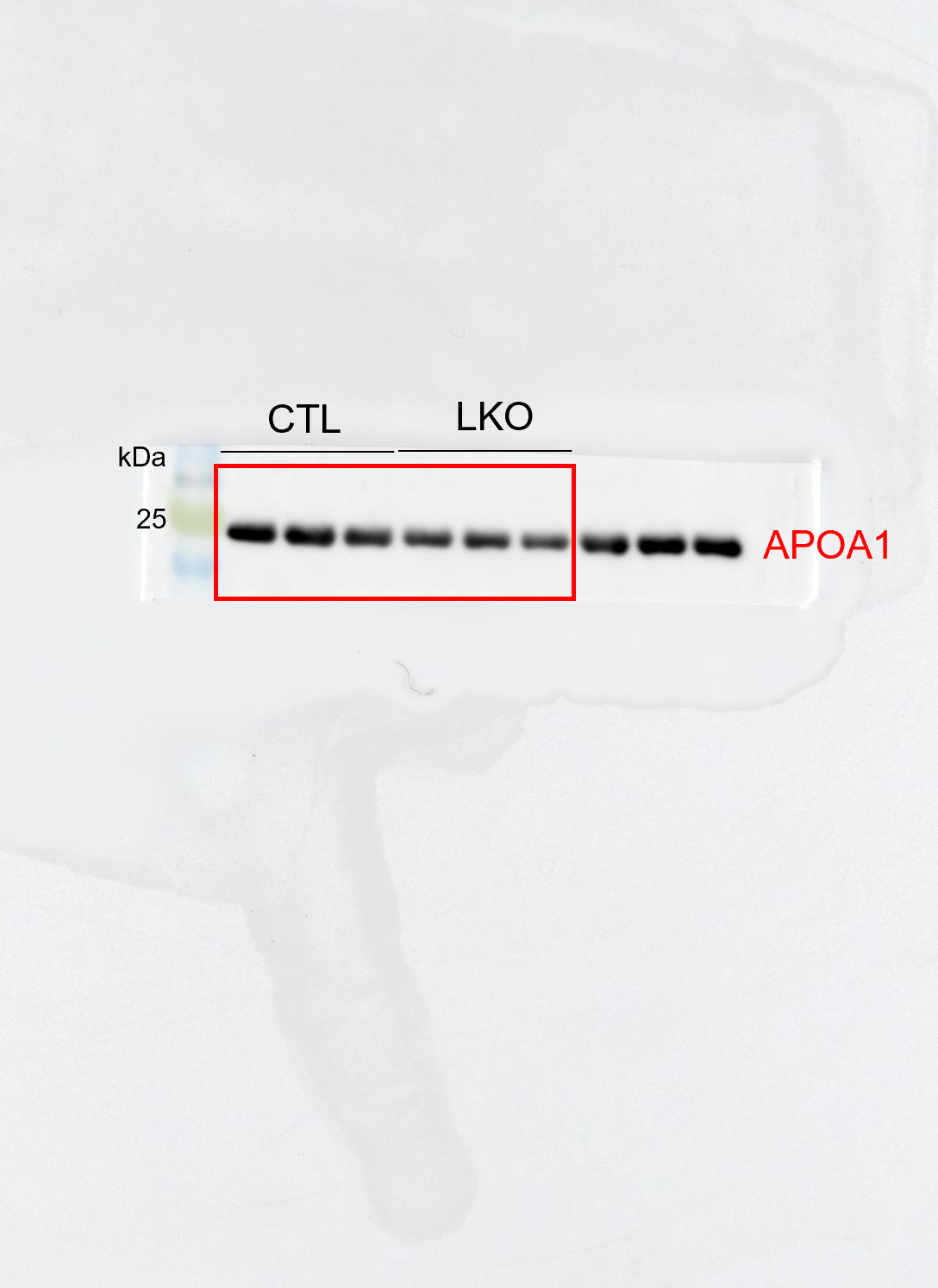

Supplement: Supplementary file 5 — Source data Fig. 2 [file 44318_2026_754_MOESM5_ESM.zip › Figure 2/2J-K/2J_western_APOA1_label.tif]

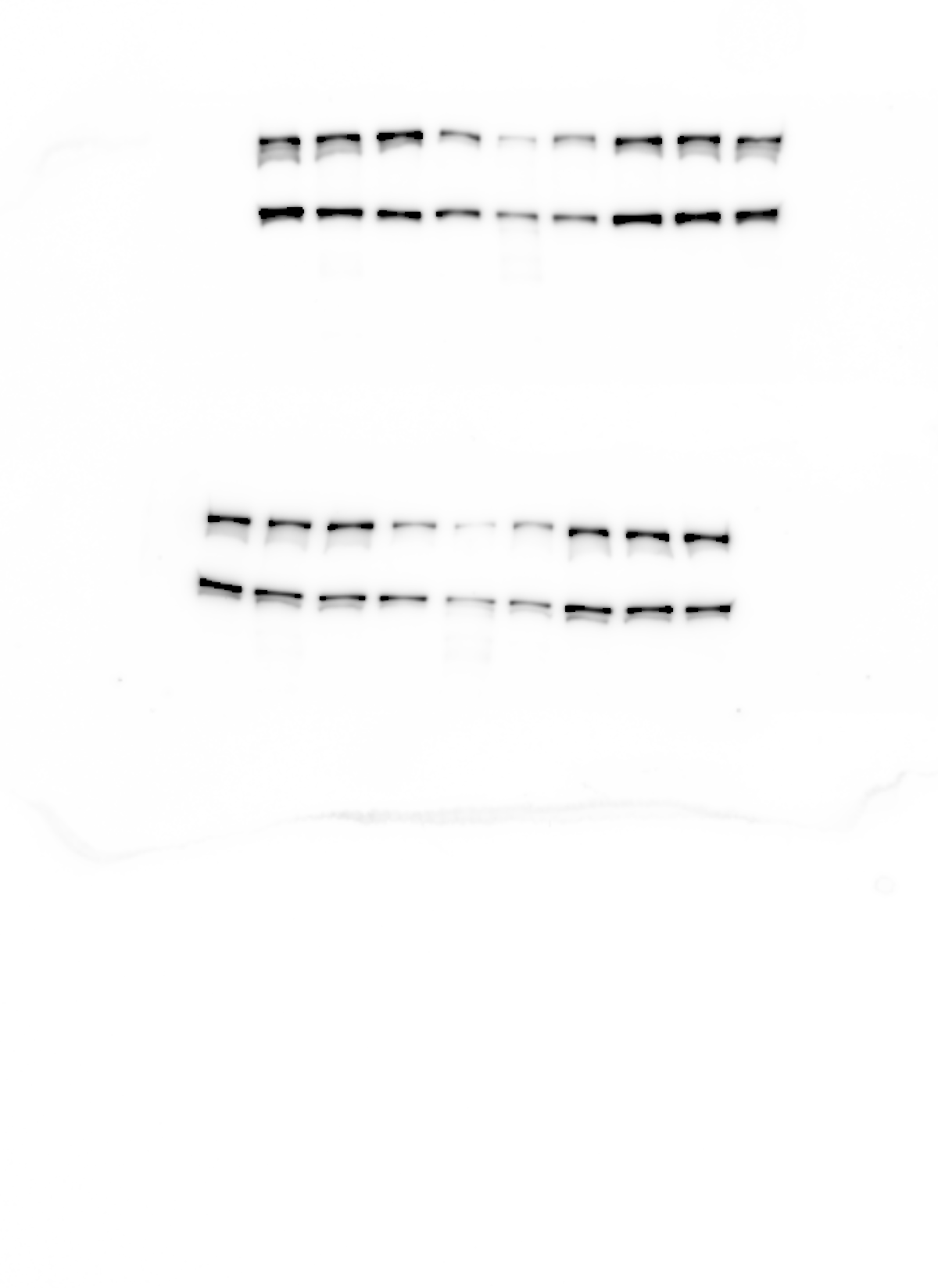

Supplement: Supplementary file 5 — Source data Fig. 2 [file 44318_2026_754_MOESM5_ESM.zip › Figure 2/2J-K/2J_western_APOB.tif]

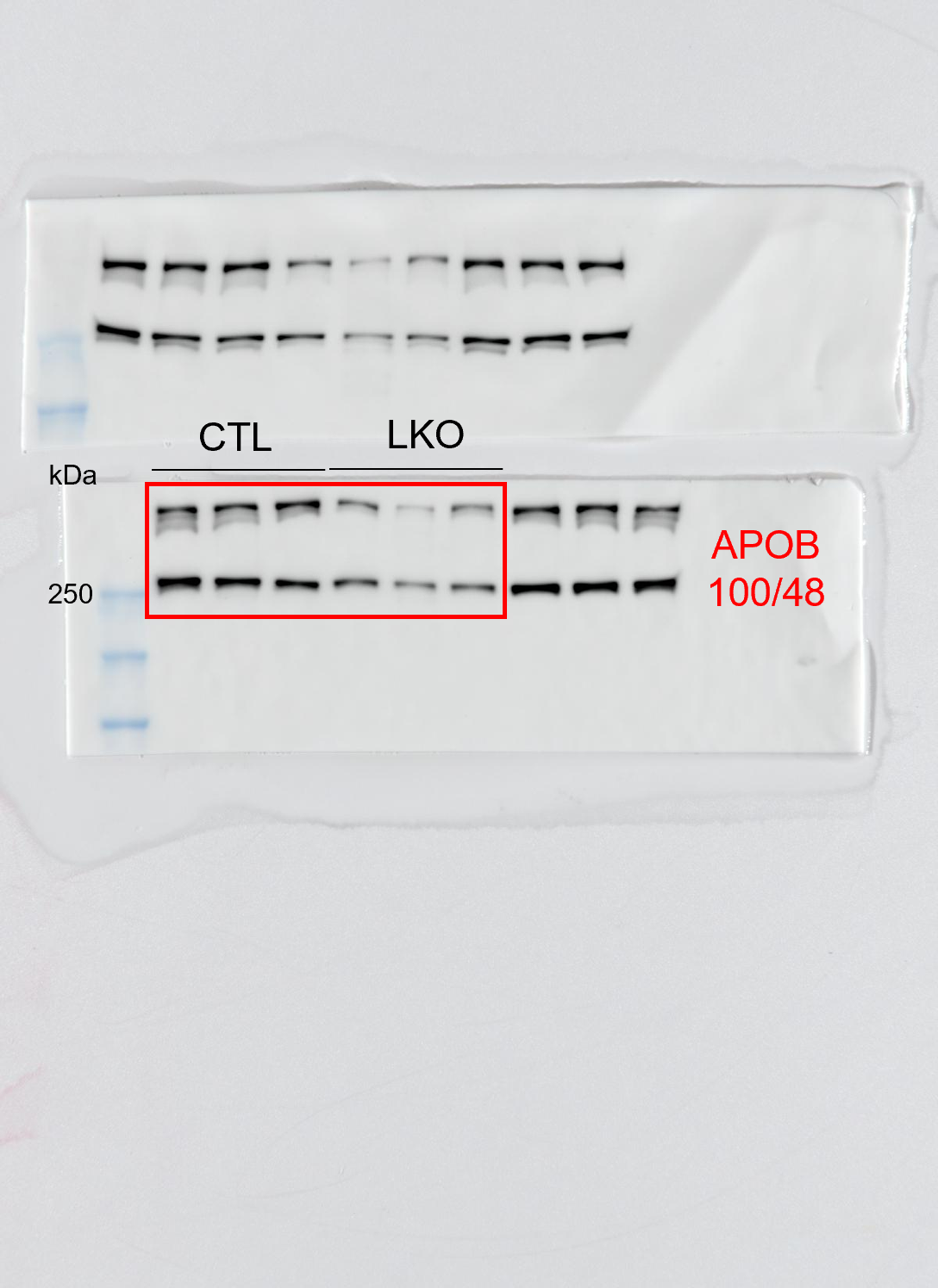

Supplement: Supplementary file 5 — Source data Fig. 2 [file 44318_2026_754_MOESM5_ESM.zip › Figure 2/2J-K/2J_western_APOB_label.tif]

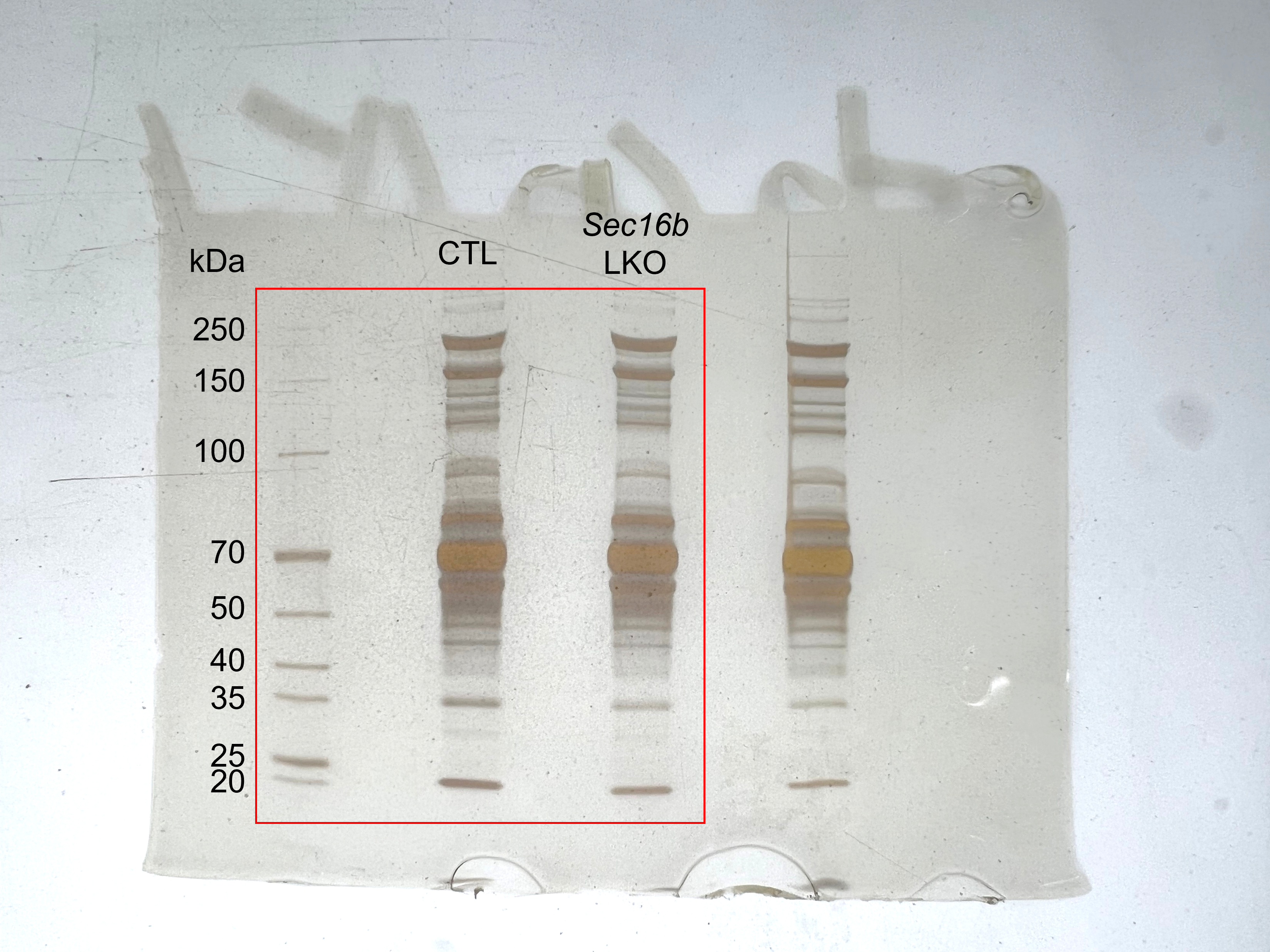

Supplement: Supplementary file 5 — Source data Fig. 2 [file 44318_2026_754_MOESM5_ESM.zip › Figure 2/2L/2L_Silver Staining.tif]

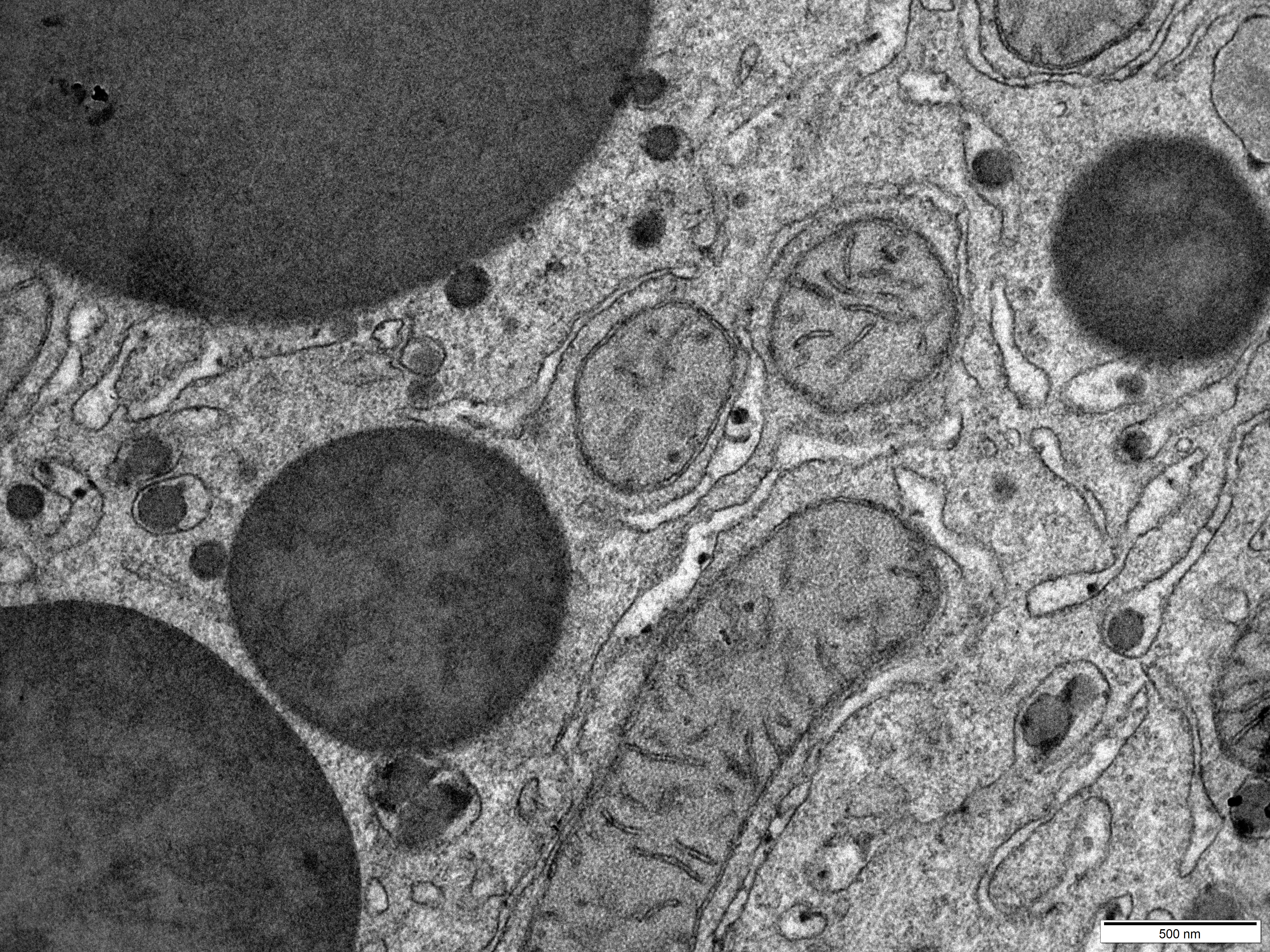

Supplement: Supplementary file 6 — Source data Fig. 3 [file 44318_2026_754_MOESM6_ESM.zip › Figure 3/3A-C/3A_KO ER.tiff]

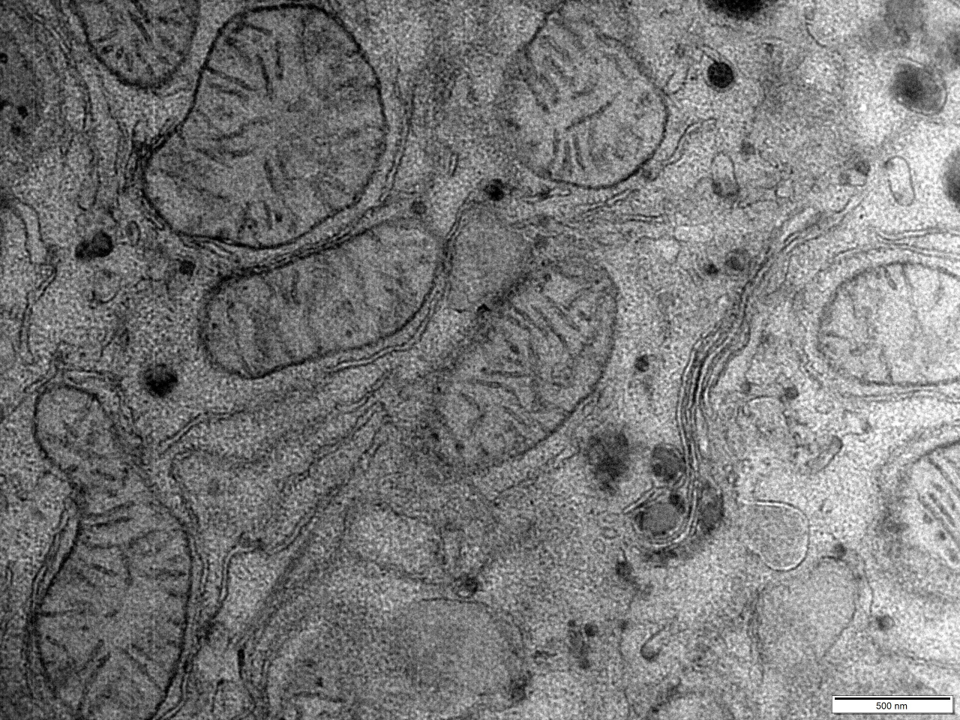

Supplement: Supplementary file 6 — Source data Fig. 3 [file 44318_2026_754_MOESM6_ESM.zip › Figure 3/3A-C/3A_KO Golgi.tif]

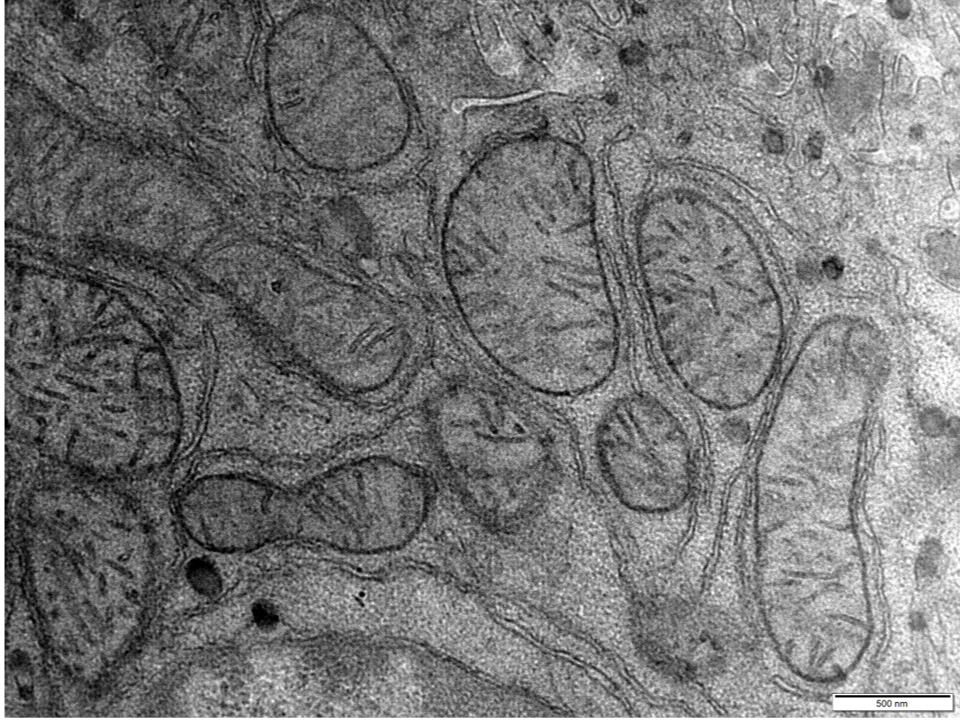

Supplement: Supplementary file 6 — Source data Fig. 3 [file 44318_2026_754_MOESM6_ESM.zip › Figure 3/3A-C/3A_KO Mitochondrial.tif]

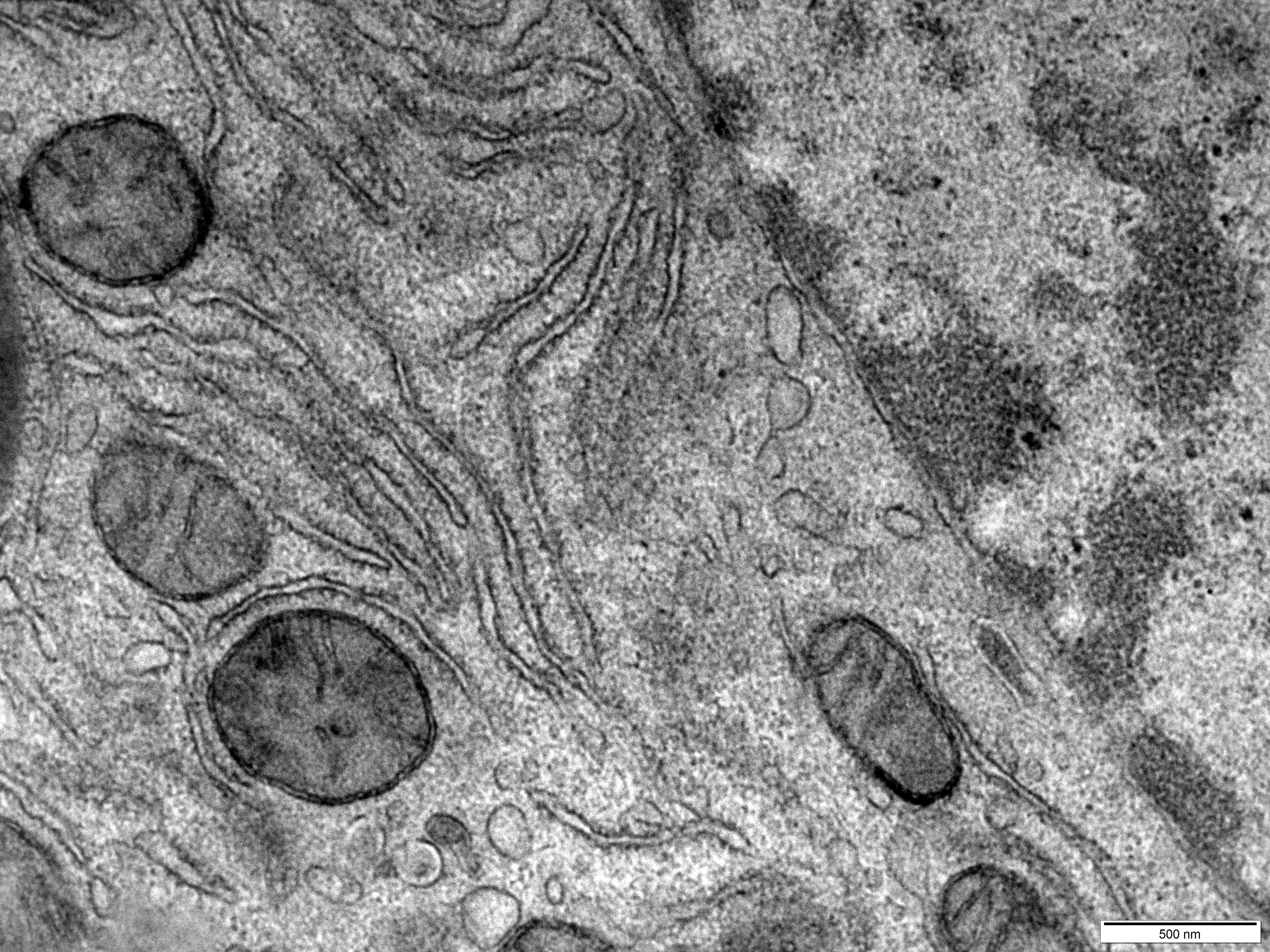

Supplement: Supplementary file 6 — Source data Fig. 3 [file 44318_2026_754_MOESM6_ESM.zip › Figure 3/3A-C/3A_WT ER.tiff]

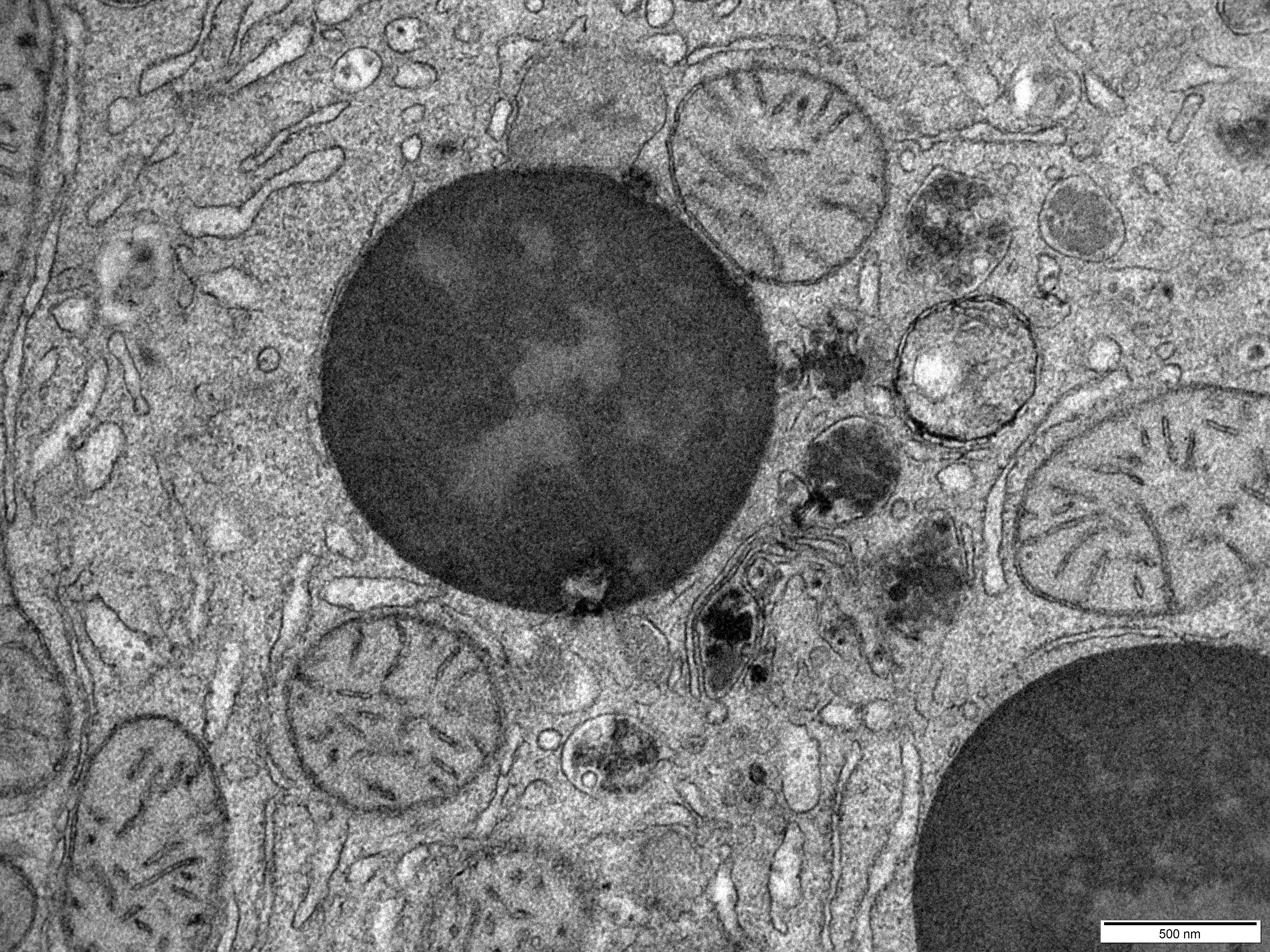

Supplement: Supplementary file 6 — Source data Fig. 3 [file 44318_2026_754_MOESM6_ESM.zip › Figure 3/3A-C/3A_WT Golgi.tiff]

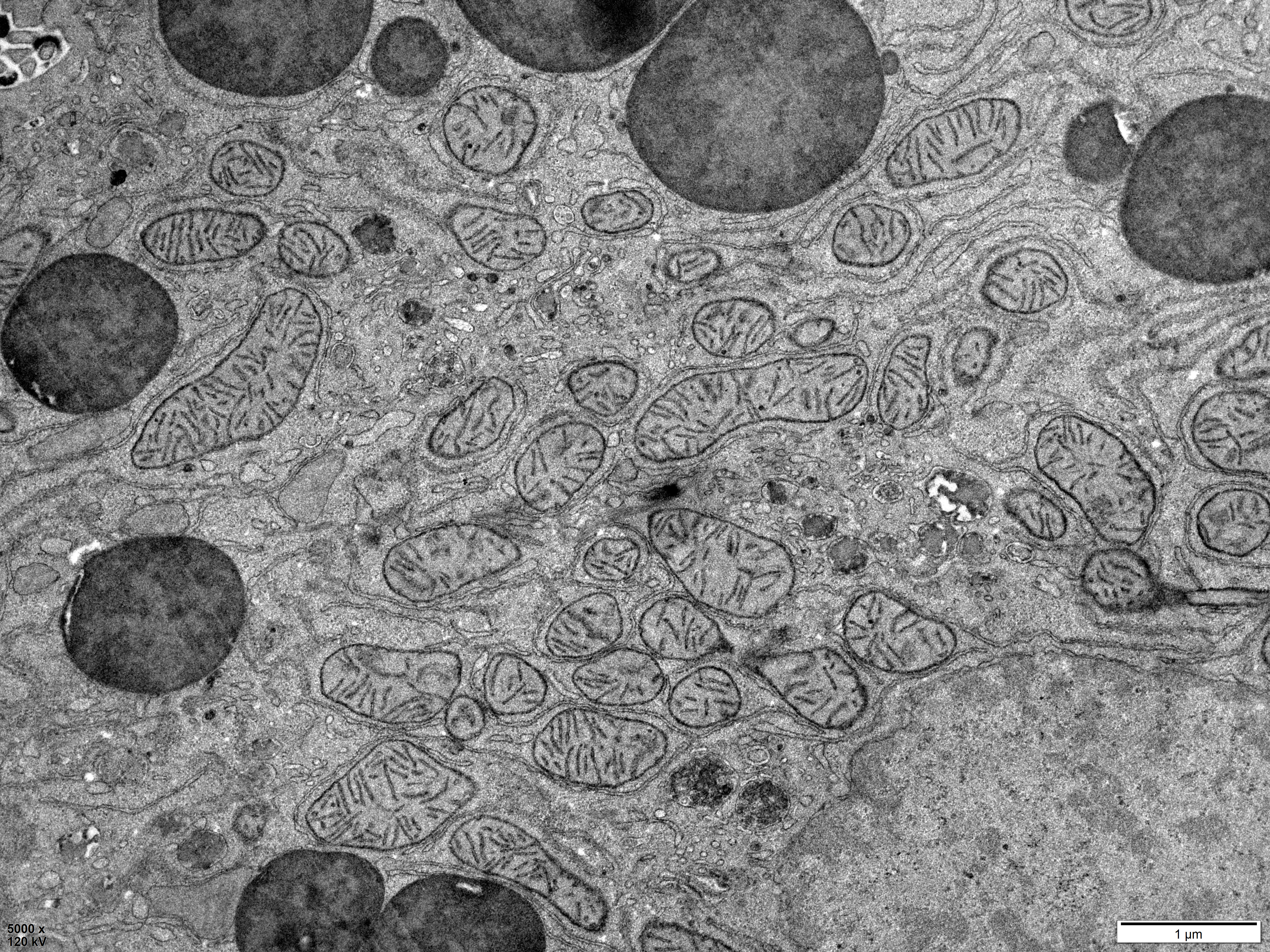

Supplement: Supplementary file 6 — Source data Fig. 3 [file 44318_2026_754_MOESM6_ESM.zip › Figure 3/3A-C/3A_WT Mitochondrial.tiff]

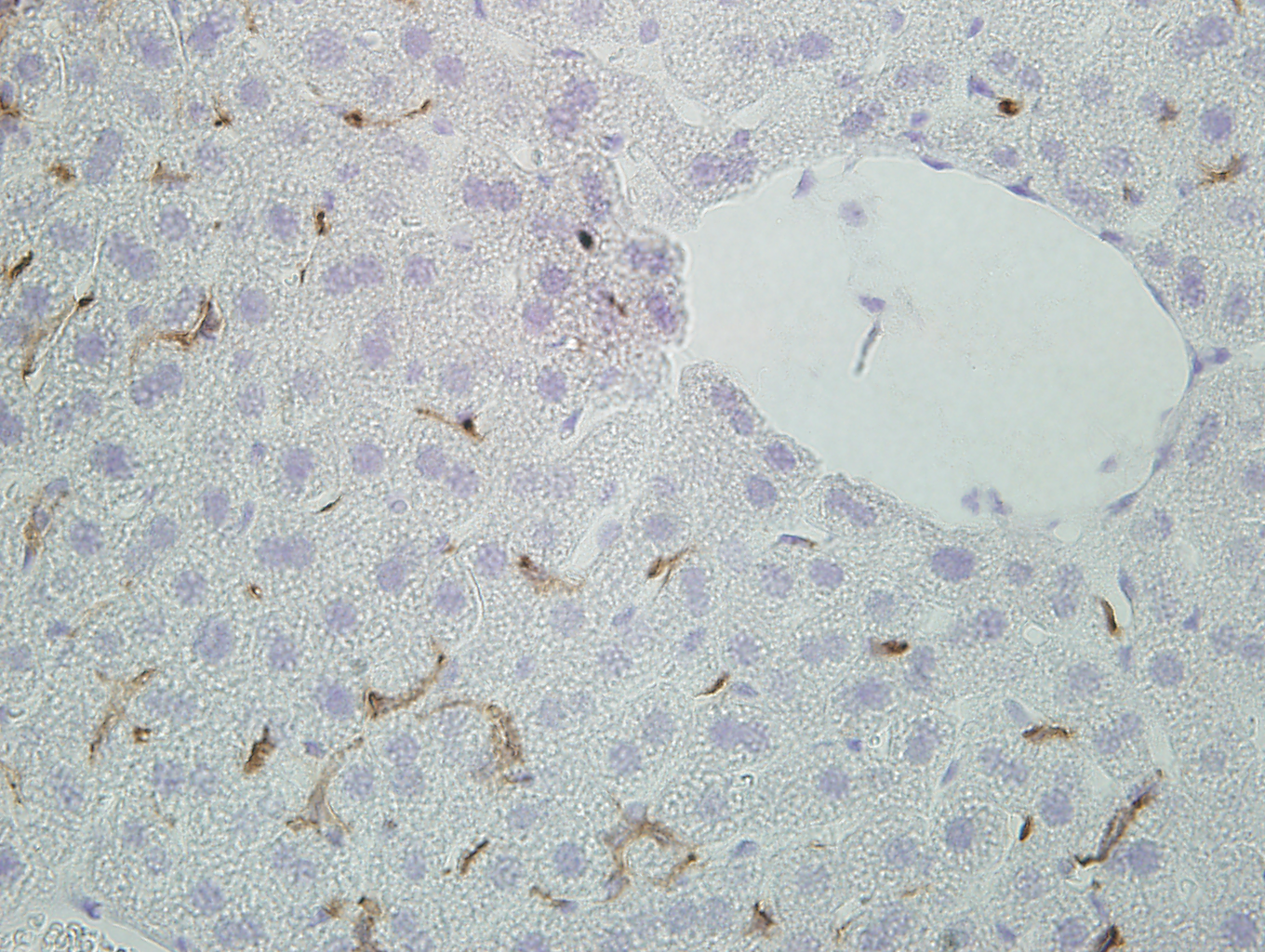

Supplement: Supplementary file 6 — Source data Fig. 3 [file 44318_2026_754_MOESM6_ESM.zip › Figure 3/3D/3D_F480 CTL.tif]

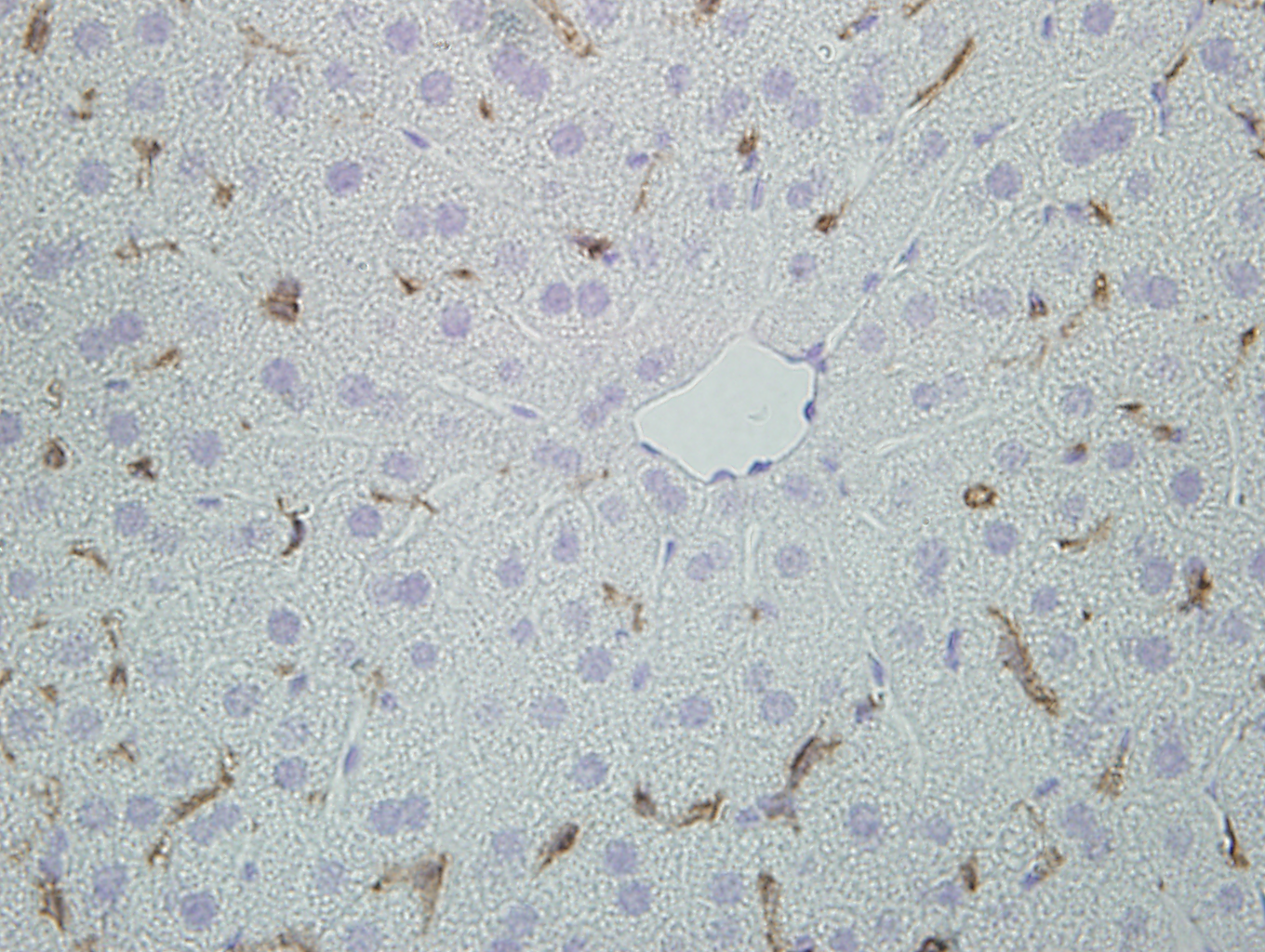

Supplement: Supplementary file 6 — Source data Fig. 3 [file 44318_2026_754_MOESM6_ESM.zip › Figure 3/3D/3D_F480 KO.tif]

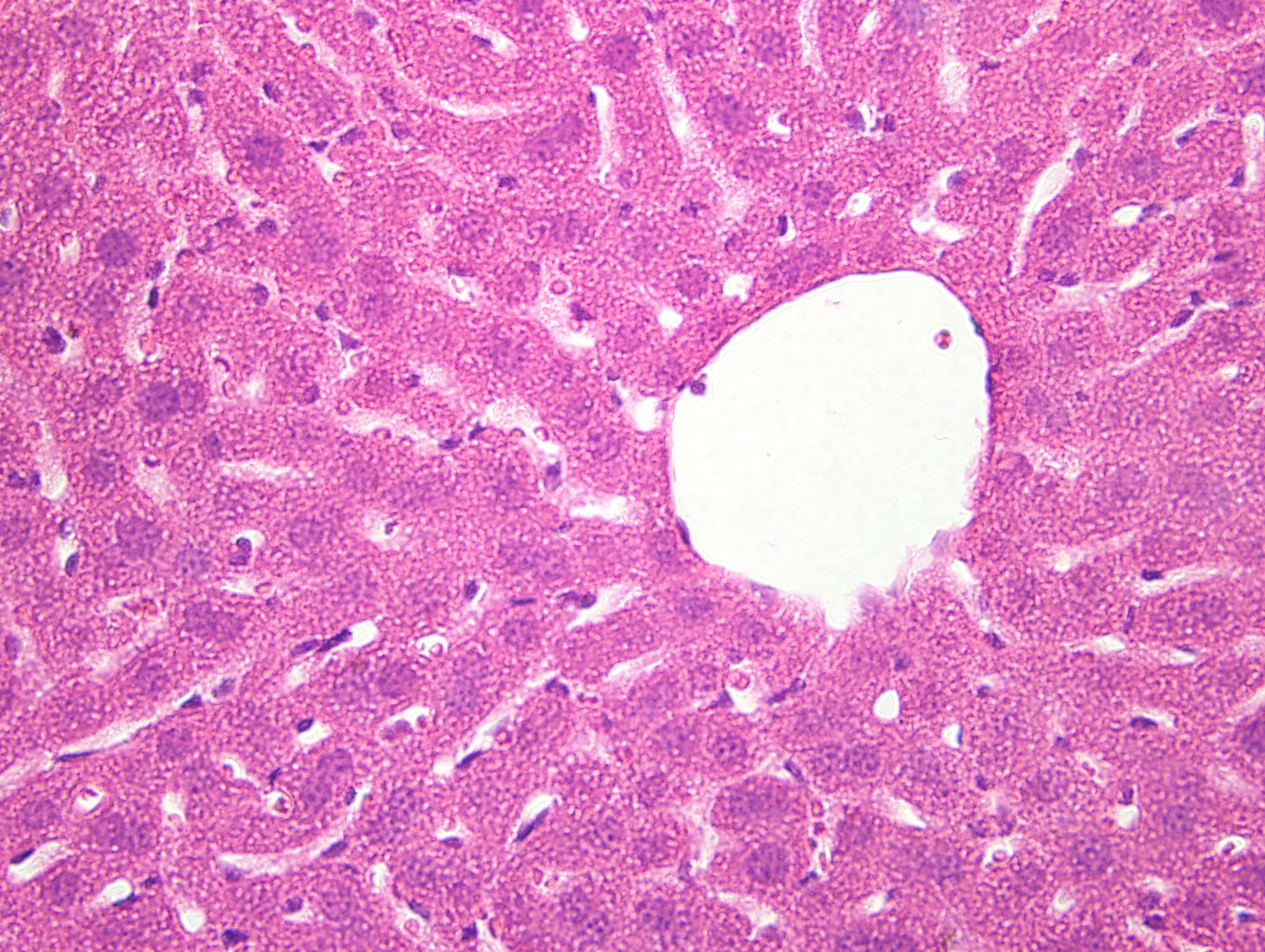

Supplement: Supplementary file 6 — Source data Fig. 3 [file 44318_2026_754_MOESM6_ESM.zip › Figure 3/3D/3D_H&E CTL.tif]

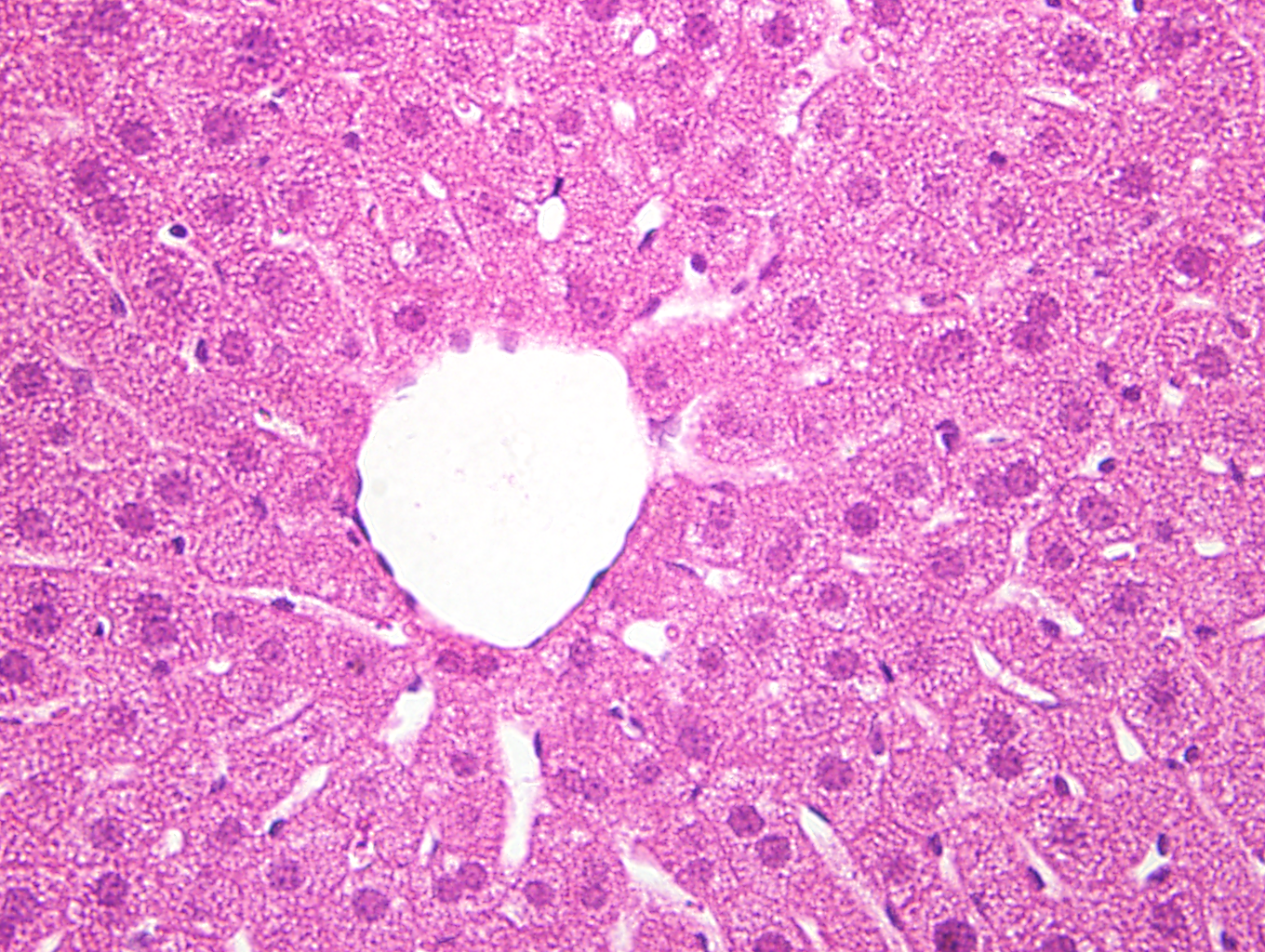

Supplement: Supplementary file 6 — Source data Fig. 3 [file 44318_2026_754_MOESM6_ESM.zip › Figure 3/3D/3D_H&E KO.tif]

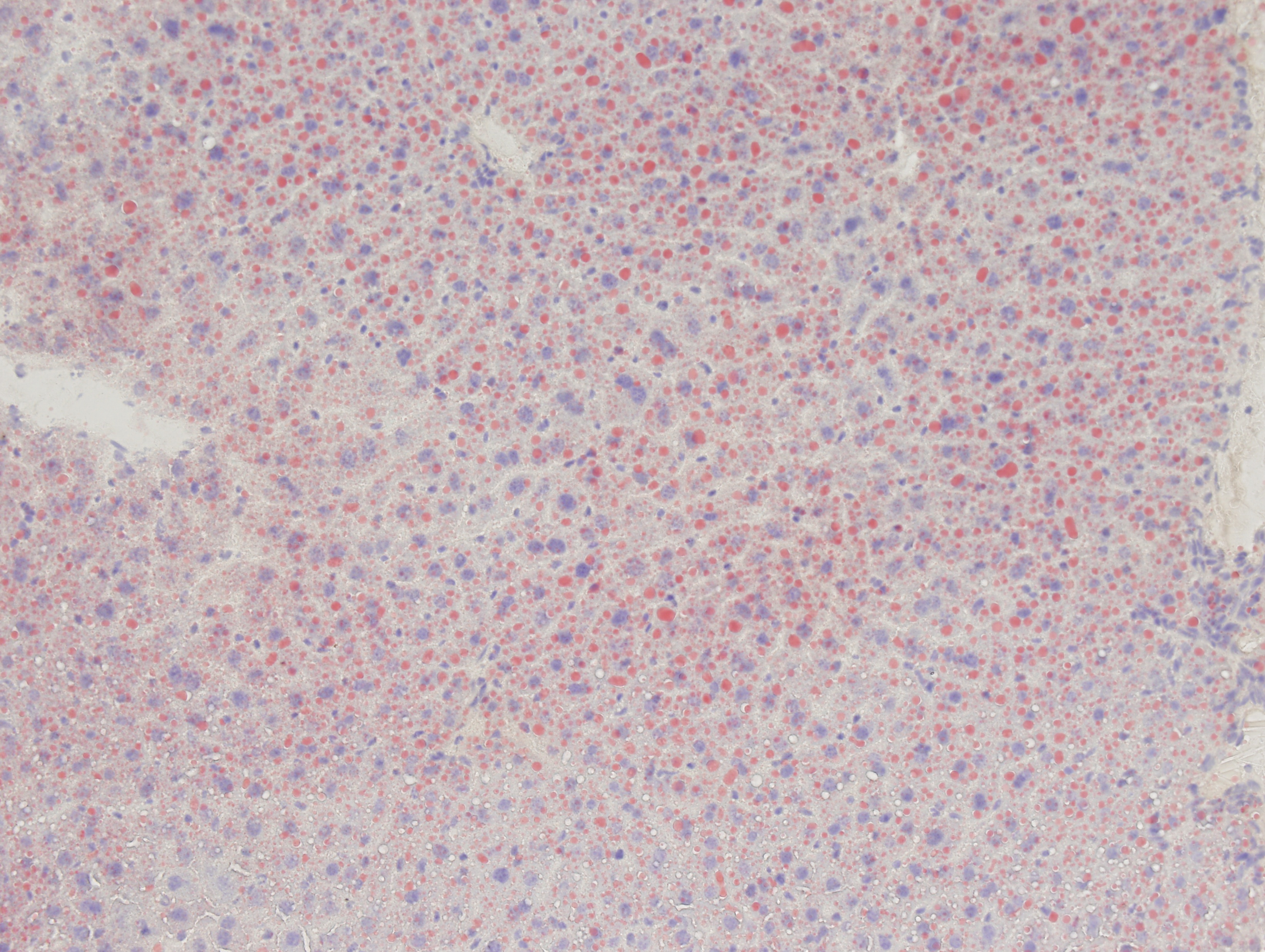

Supplement: Supplementary file 6 — Source data Fig. 3 [file 44318_2026_754_MOESM6_ESM.zip › Figure 3/3D/3D_ORO CTL.tif]

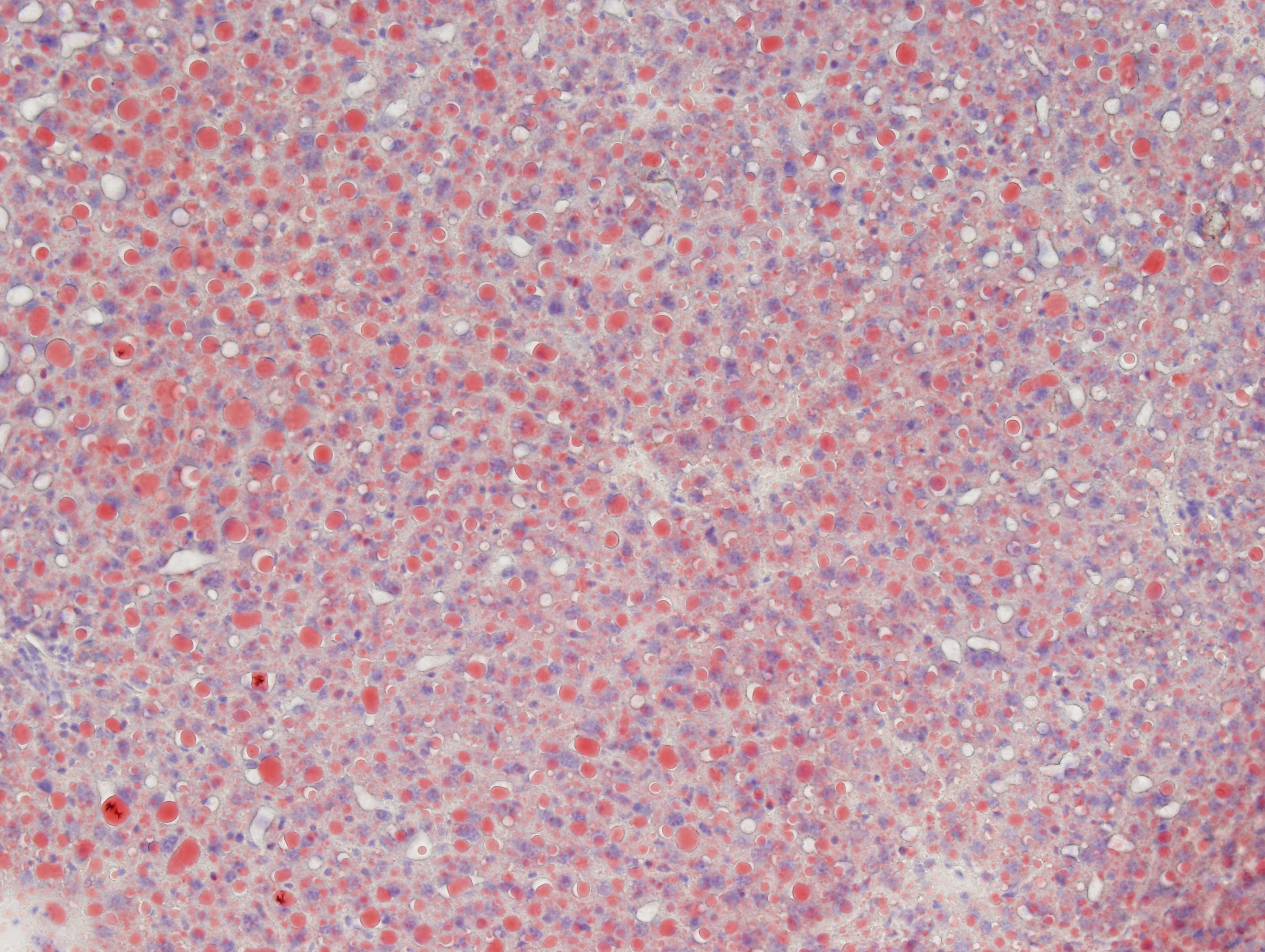

Supplement: Supplementary file 6 — Source data Fig. 3 [file 44318_2026_754_MOESM6_ESM.zip › Figure 3/3D/3D_ORO KO.tif]

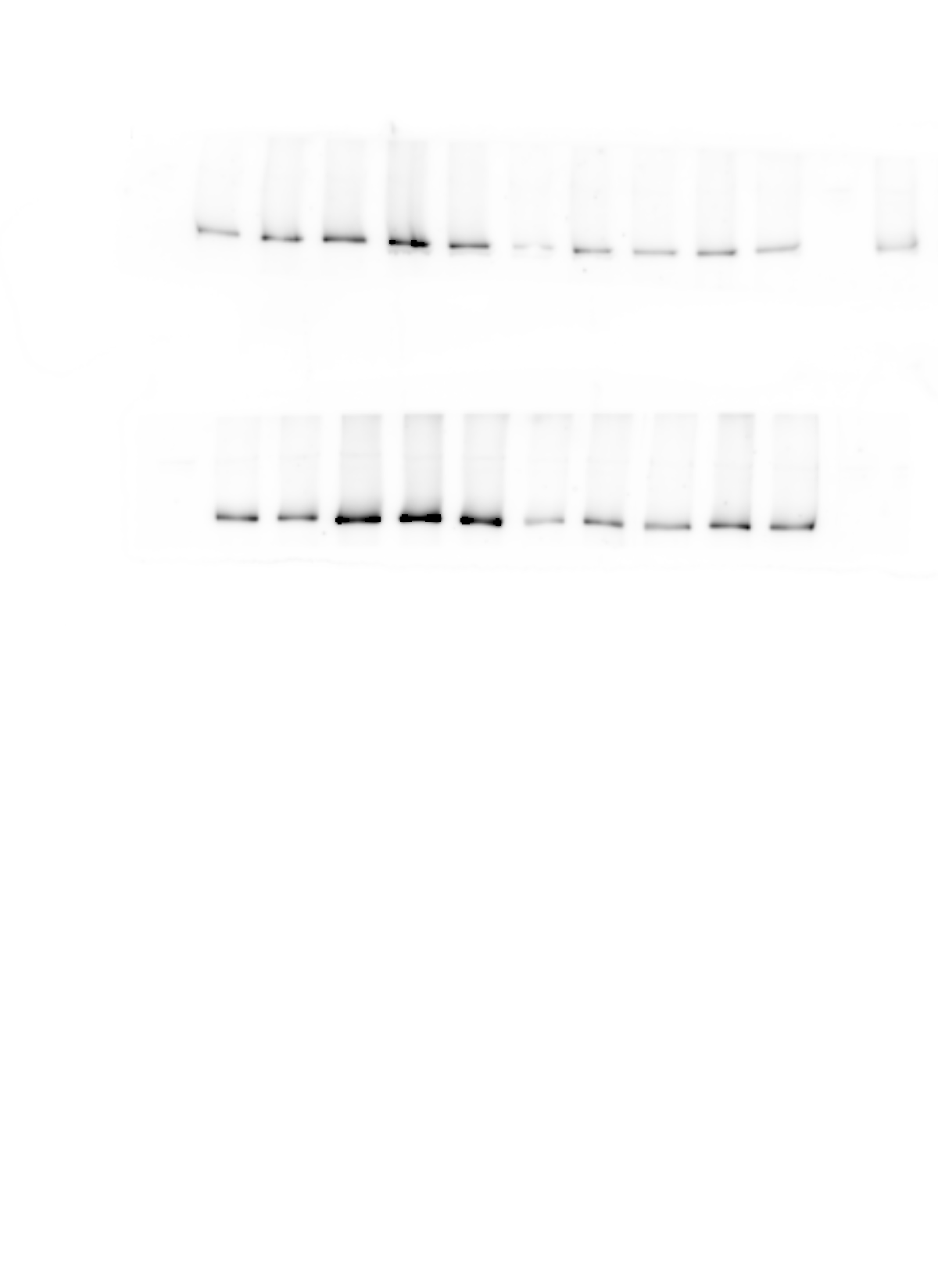

Supplement: Supplementary file 6 — Source data Fig. 3 [file 44318_2026_754_MOESM6_ESM.zip › Figure 3/3L/3L_western_ACC.tif]

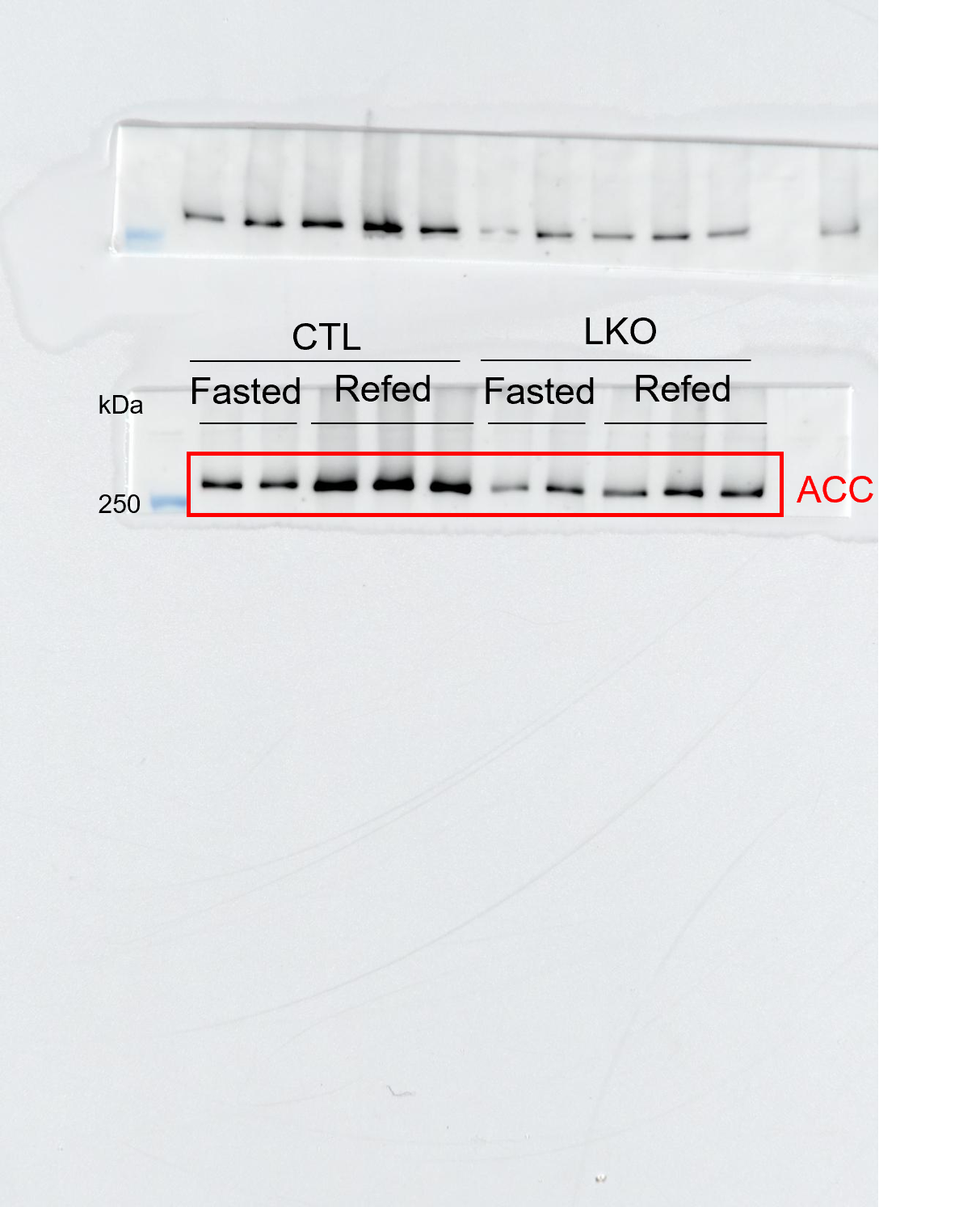

Supplement: Supplementary file 6 — Source data Fig. 3 [file 44318_2026_754_MOESM6_ESM.zip › Figure 3/3L/3L_western_ACC_label.tif]

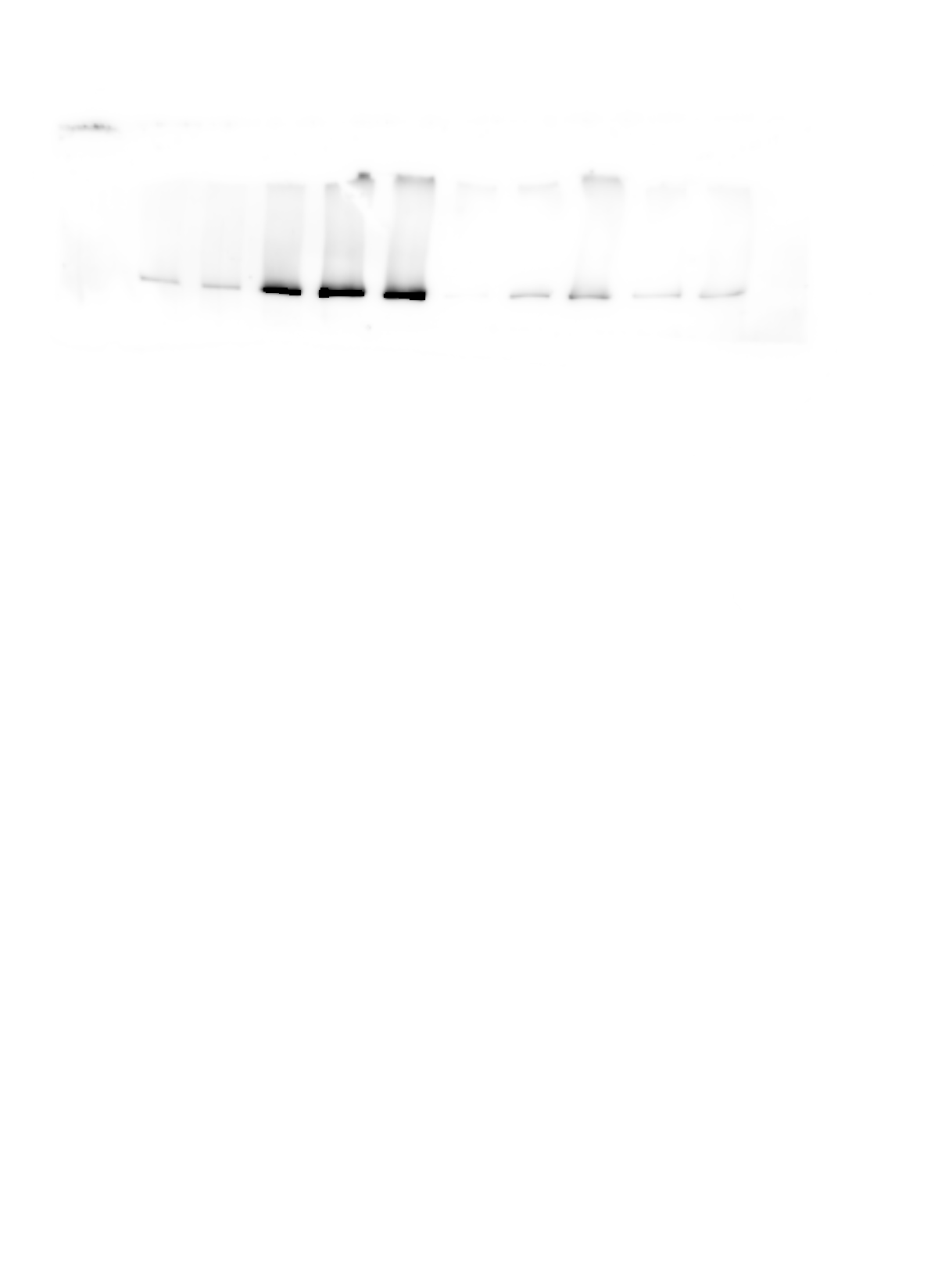

Supplement: Supplementary file 6 — Source data Fig. 3 [file 44318_2026_754_MOESM6_ESM.zip › Figure 3/3L/3L_western_FASN.tif]

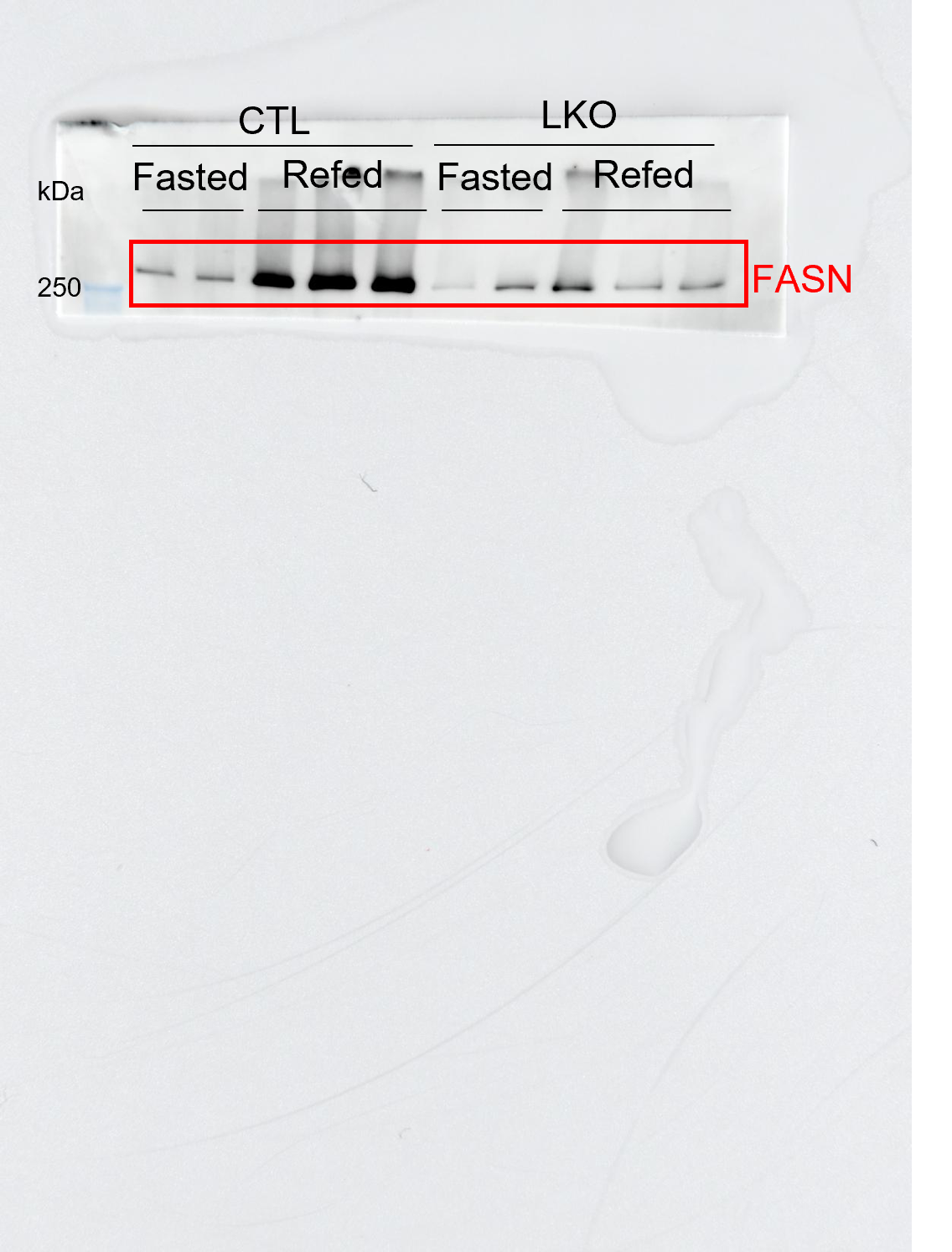

Supplement: Supplementary file 6 — Source data Fig. 3 [file 44318_2026_754_MOESM6_ESM.zip › Figure 3/3L/3L_western_FASN_label.tif]

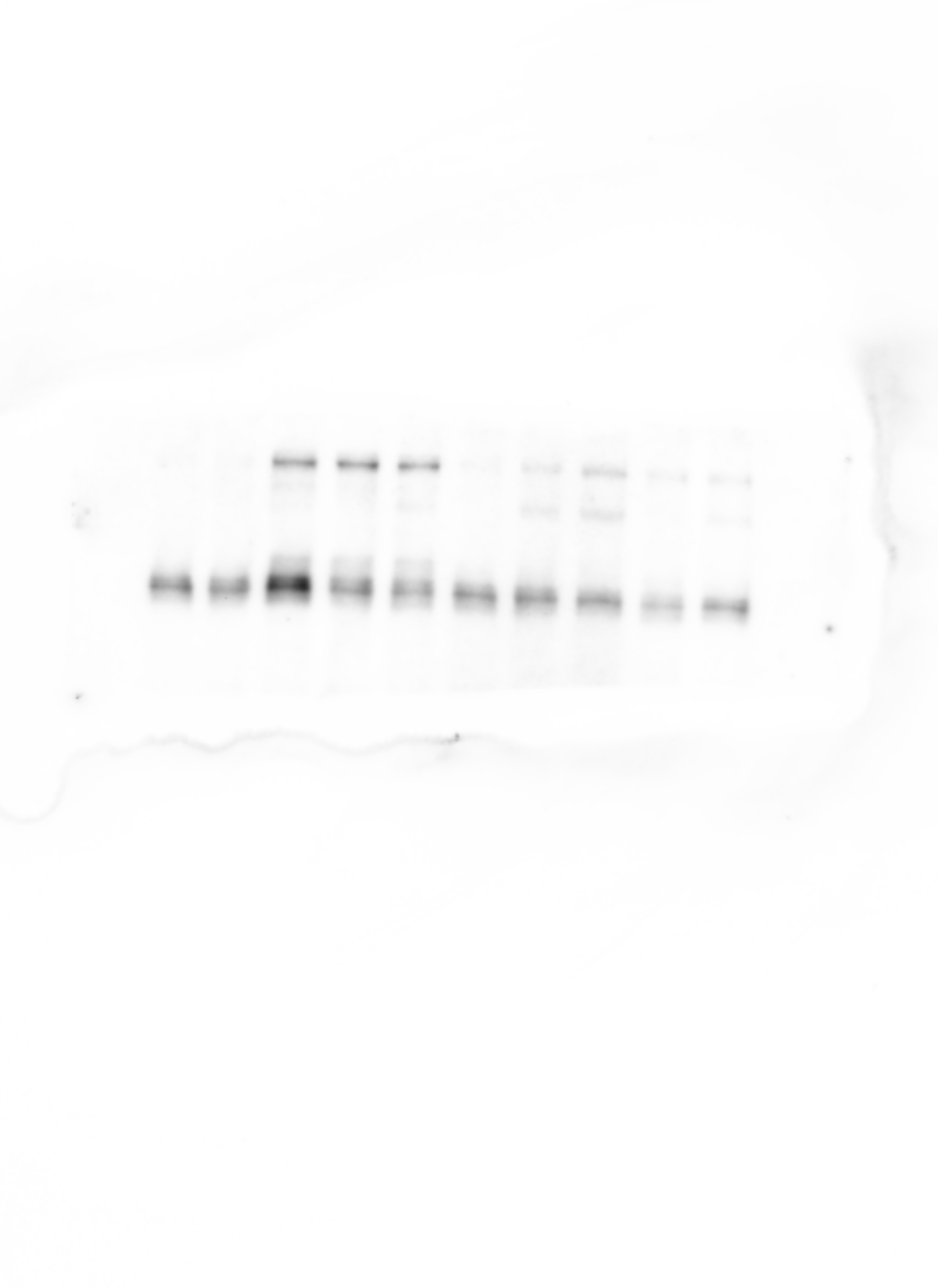

Supplement: Supplementary file 6 — Source data Fig. 3 [file 44318_2026_754_MOESM6_ESM.zip › Figure 3/3L/3L_western_SREBP1.tif]

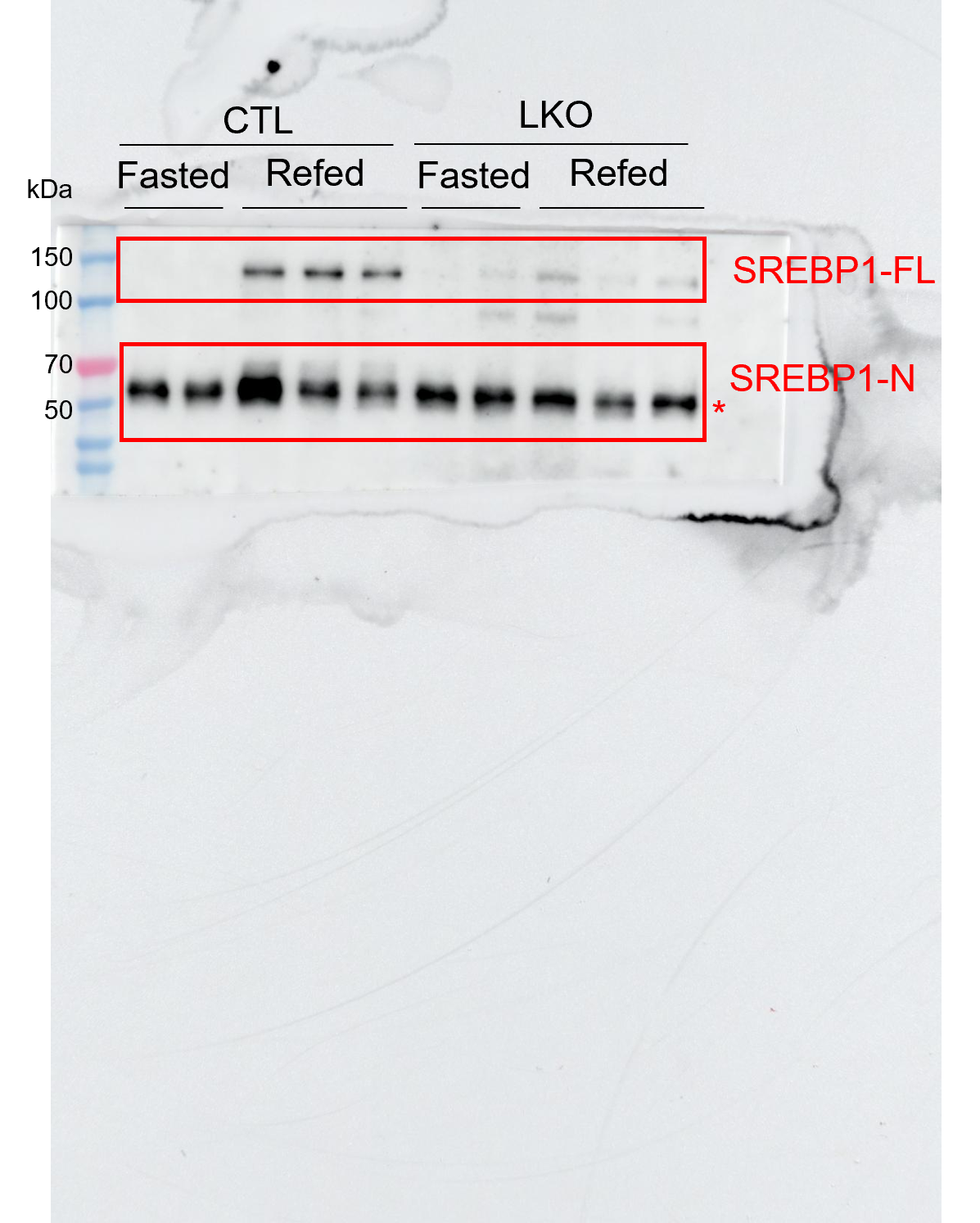

Supplement: Supplementary file 6 — Source data Fig. 3 [file 44318_2026_754_MOESM6_ESM.zip › Figure 3/3L/3L_western_SREBP1_label.tif]

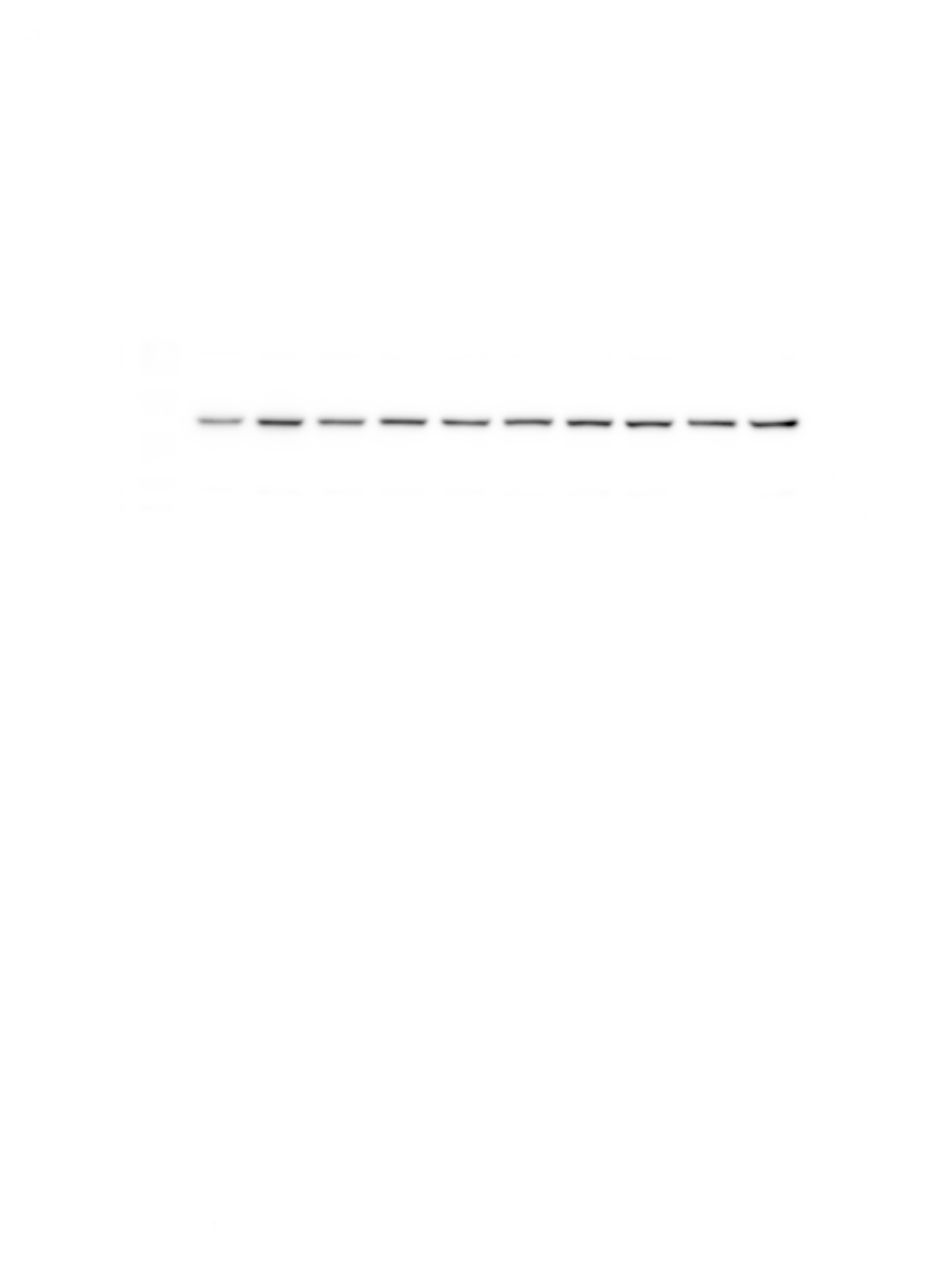

Supplement: Supplementary file 6 — Source data Fig. 3 [file 44318_2026_754_MOESM6_ESM.zip › Figure 3/3L/3L_western_Tub.tif]

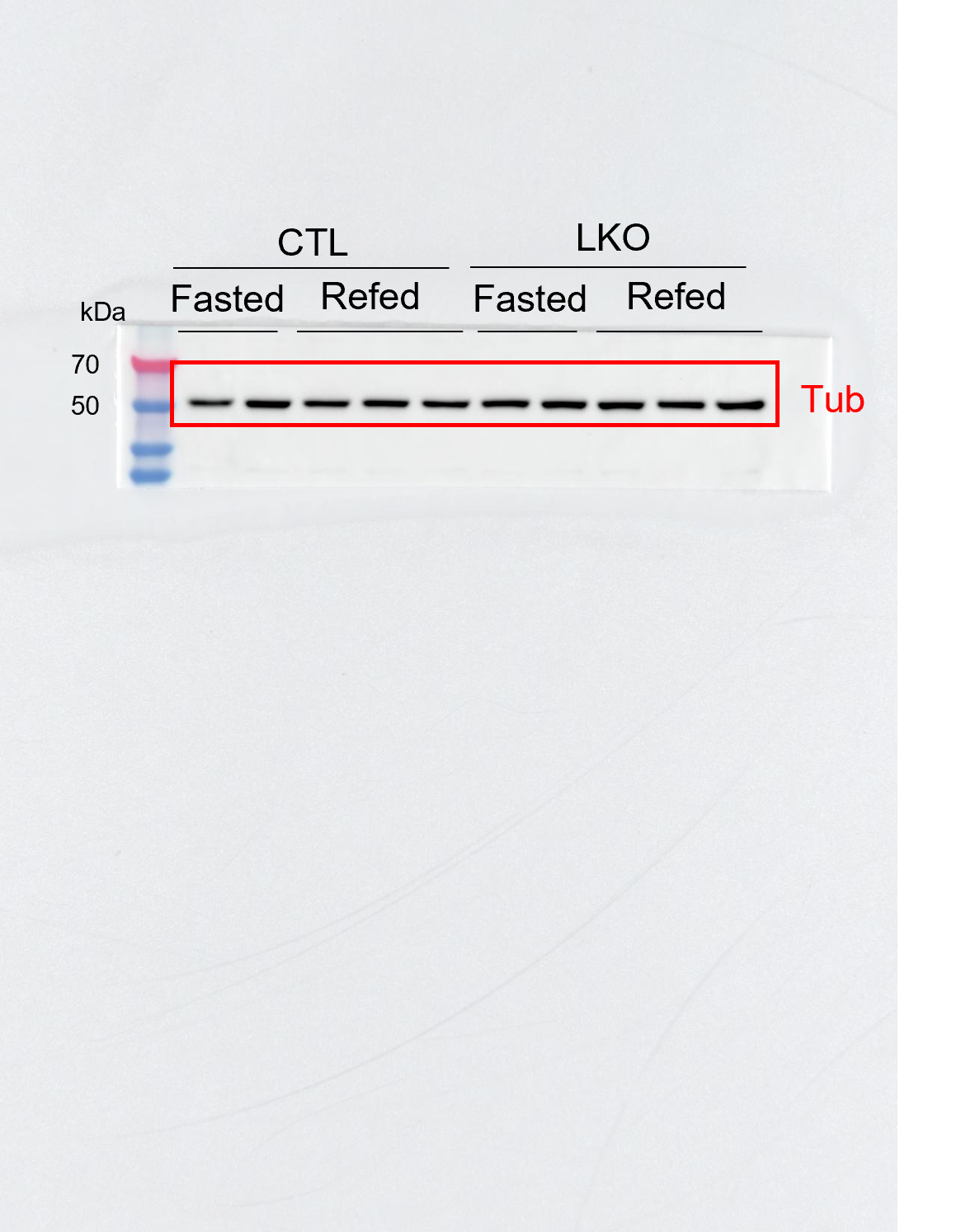

Supplement: Supplementary file 6 — Source data Fig. 3 [file 44318_2026_754_MOESM6_ESM.zip › Figure 3/3L/3L_western_Tub_label.tif]

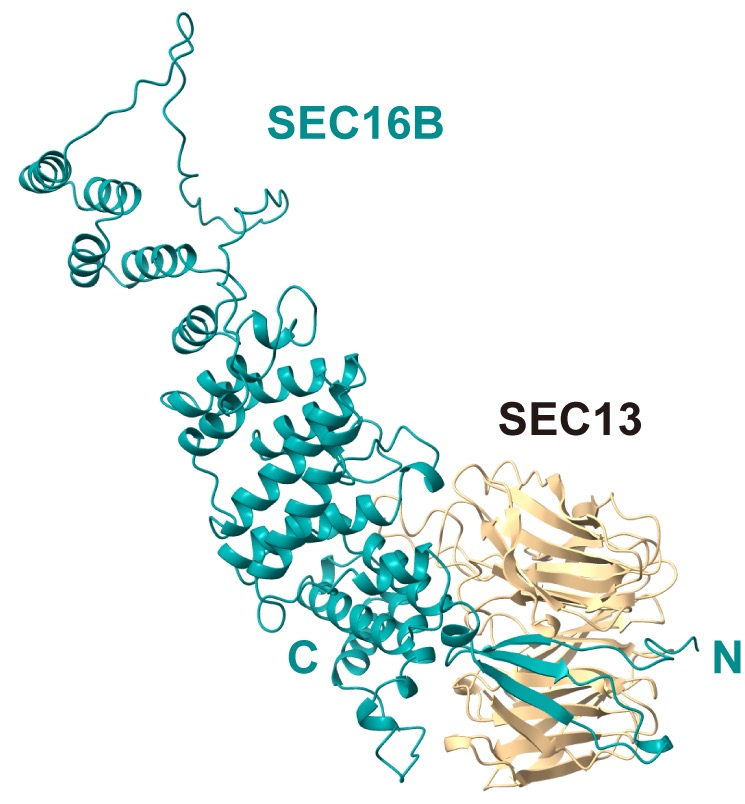

Supplement: Supplementary file 7 — Source data Fig. 4 [file 44318_2026_754_MOESM7_ESM.zip › Figure 4/4A/4A_Structure SEC16B SEC13.tiff]

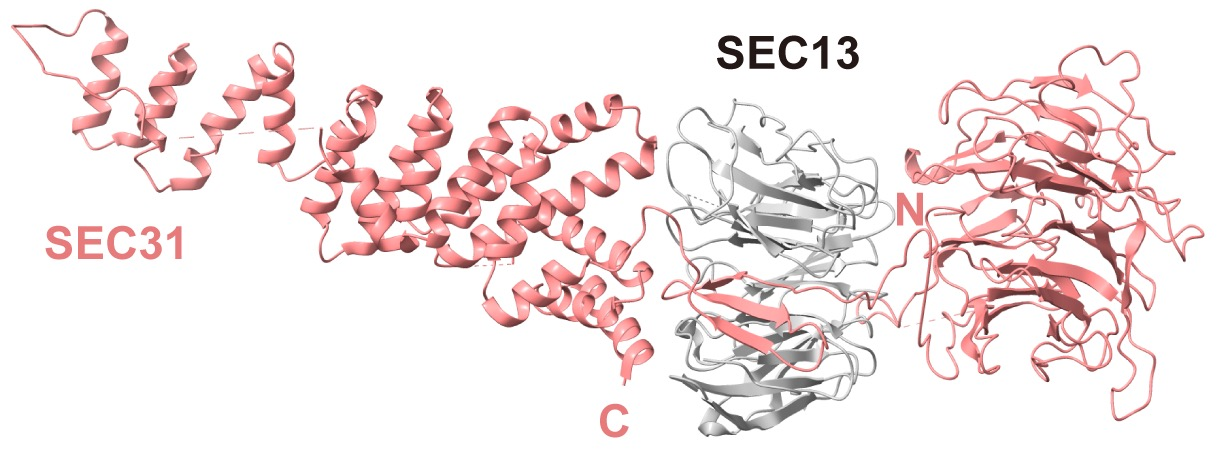

Supplement: Supplementary file 7 — Source data Fig. 4 [file 44318_2026_754_MOESM7_ESM.zip › Figure 4/4A/4A_Structure SEC31 SEC13.tiff]

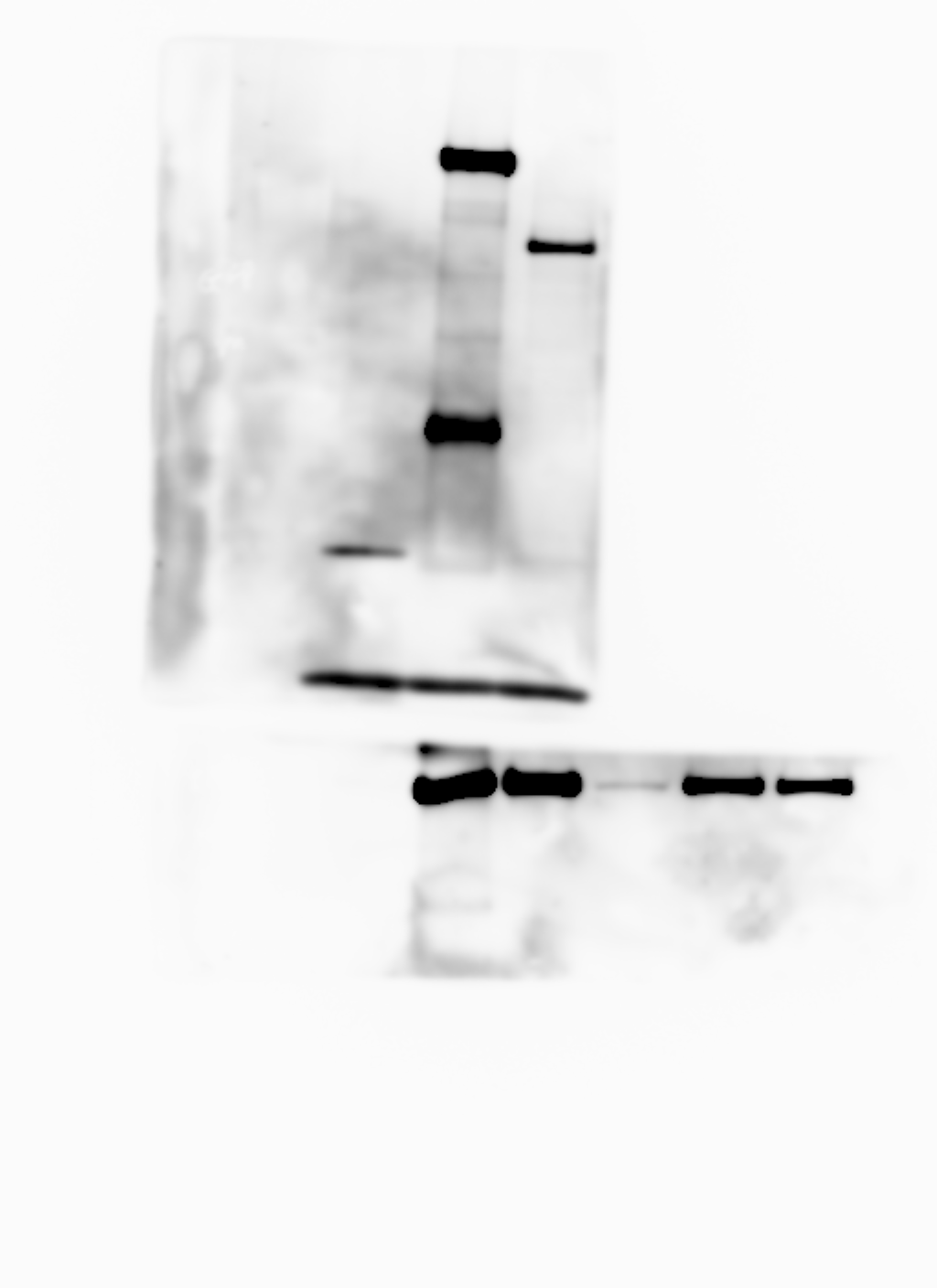

Supplement: Supplementary file 7 — Source data Fig. 4 [file 44318_2026_754_MOESM7_ESM.zip › Figure 4/4B/4B_western_SEC16 SEC13.tif]

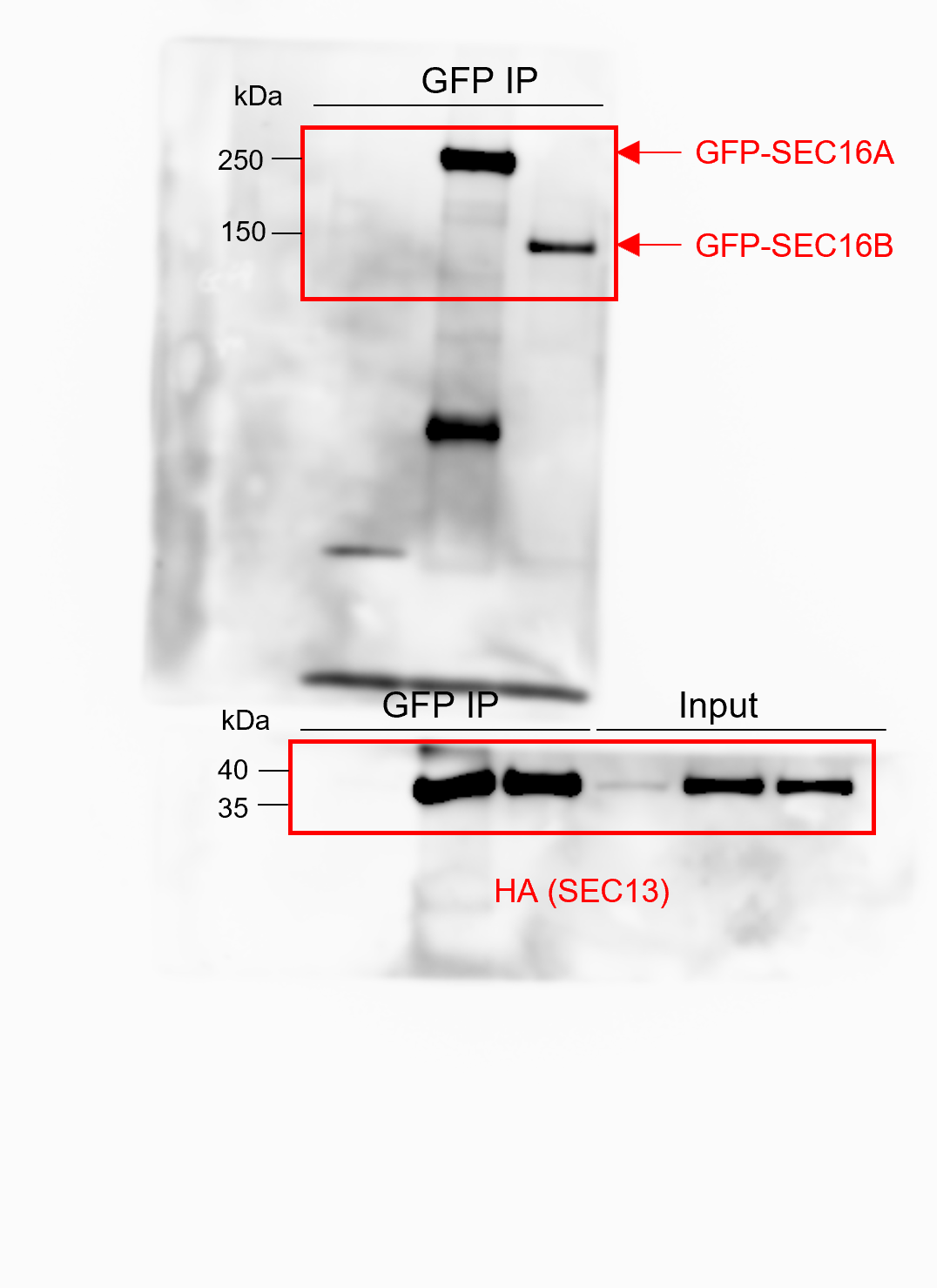

Supplement: Supplementary file 7 — Source data Fig. 4 [file 44318_2026_754_MOESM7_ESM.zip › Figure 4/4B/4B_western_SEC16 SEC13_label.tif]

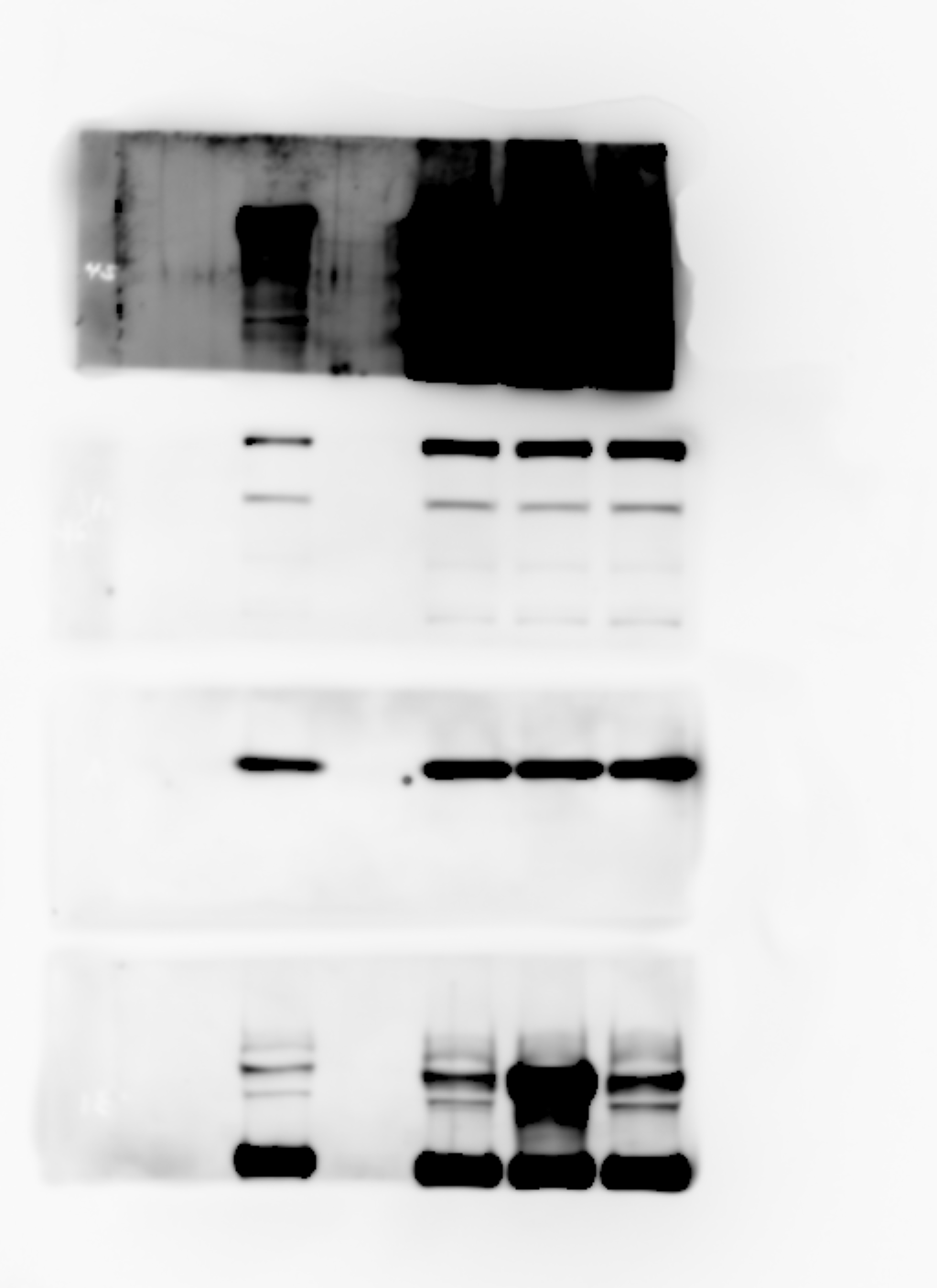

Supplement: Supplementary file 7 — Source data Fig. 4 [file 44318_2026_754_MOESM7_ESM.zip › Figure 4/4B/4B_western_SEC23 SEC24.tif]

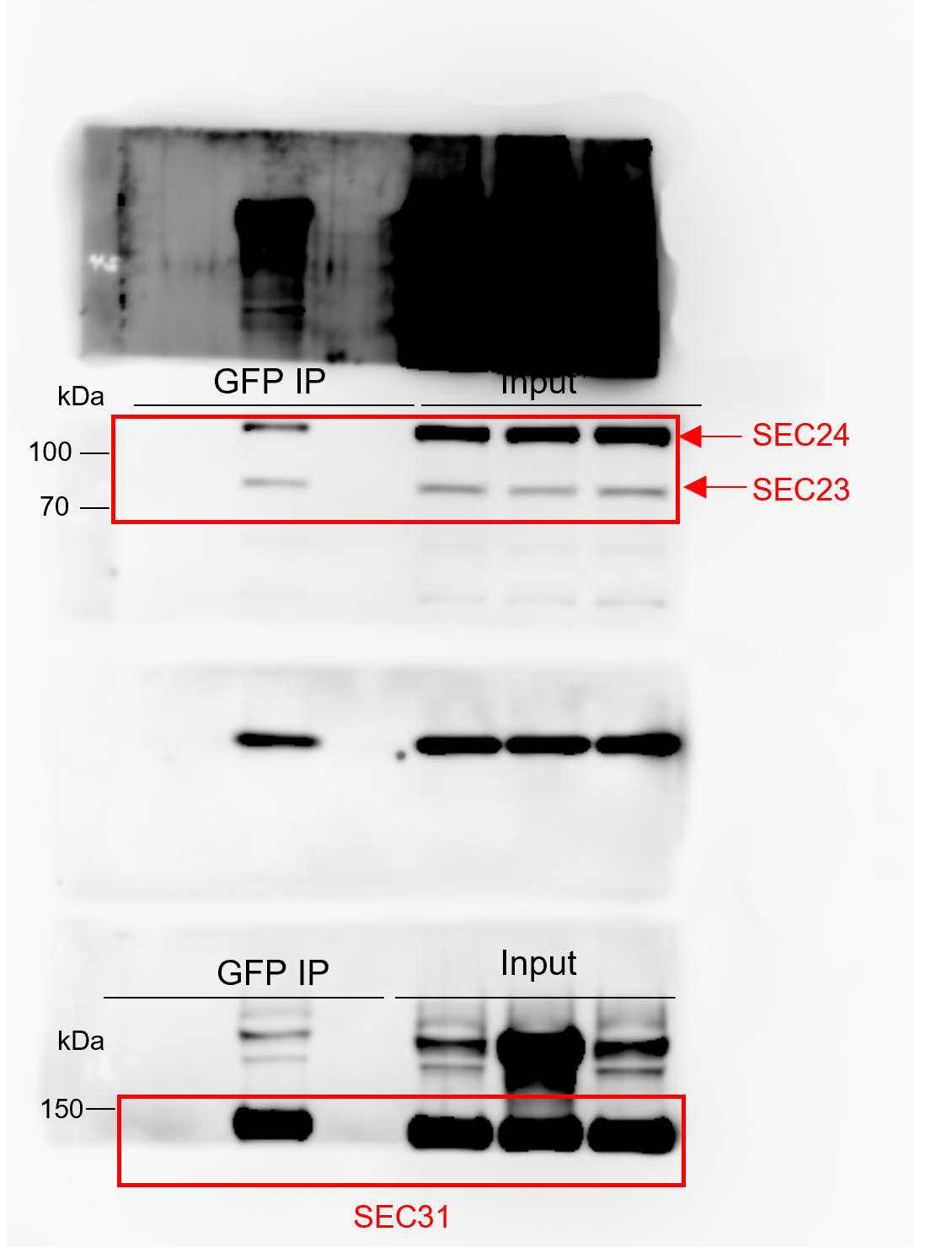

Supplement: Supplementary file 7 — Source data Fig. 4 [file 44318_2026_754_MOESM7_ESM.zip › Figure 4/4B/4B_western_SEC23 SEC24_label.tif]

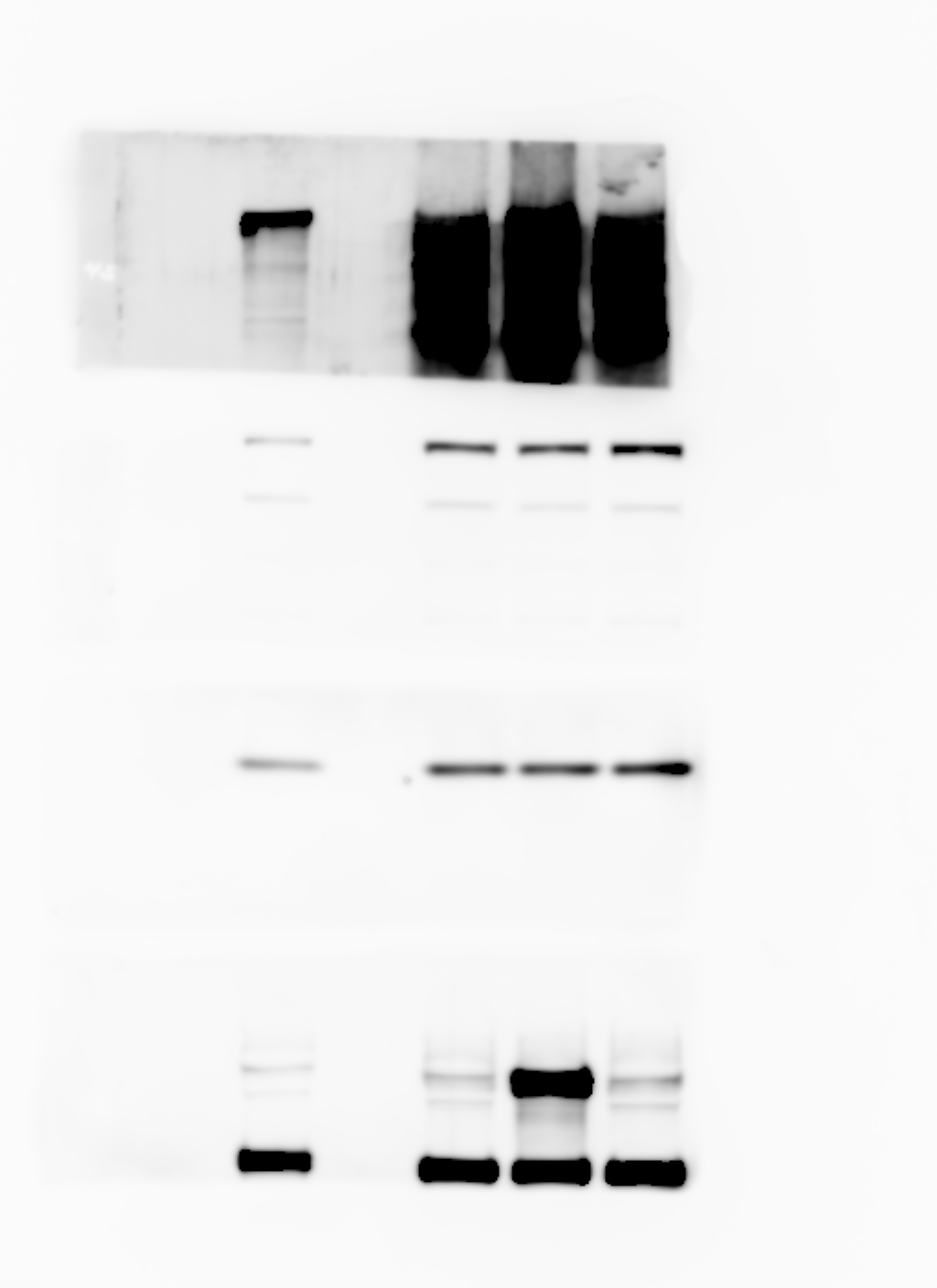

Supplement: Supplementary file 7 — Source data Fig. 4 [file 44318_2026_754_MOESM7_ESM.zip › Figure 4/4B/4B_western_SEC31.tif]

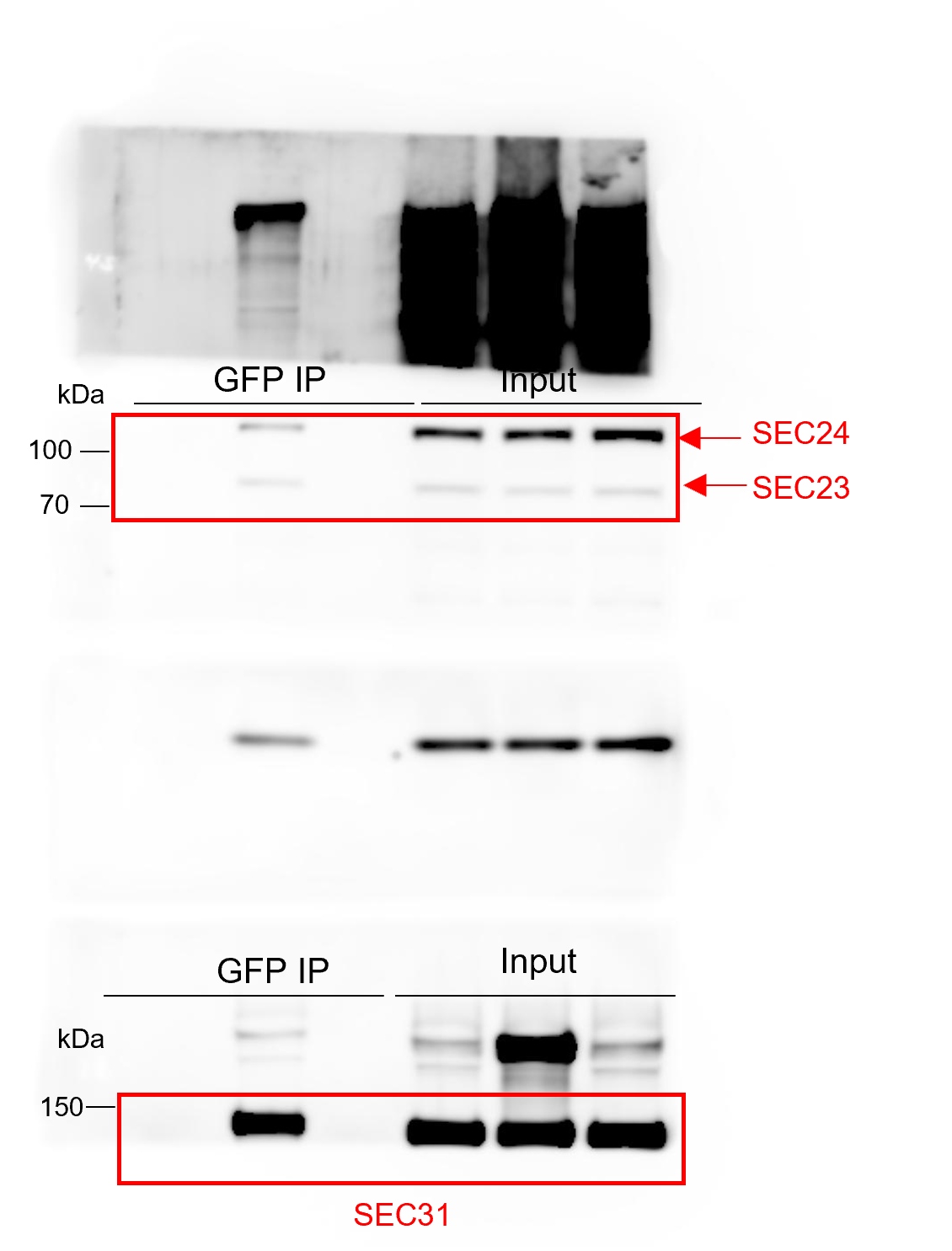

Supplement: Supplementary file 7 — Source data Fig. 4 [file 44318_2026_754_MOESM7_ESM.zip › Figure 4/4B/4B_western_SEC31_label.tif]

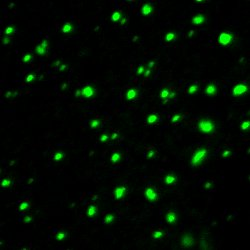

Supplement: Supplementary file 7 — Source data Fig. 4 [file 44318_2026_754_MOESM7_ESM.zip › Figure 4/4C/4C_image_Enlarge_SEC16B OE_GFP-SEC16B.tiff]

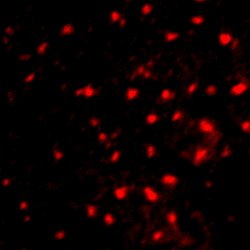

Supplement: Supplementary file 7 — Source data Fig. 4 [file 44318_2026_754_MOESM7_ESM.zip › Figure 4/4C/4C_image_Enlarge_SEC16B OE_HA-SEC13.tiff]

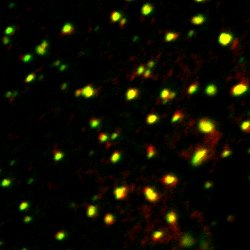

Supplement: Supplementary file 7 — Source data Fig. 4 [file 44318_2026_754_MOESM7_ESM.zip › Figure 4/4C/4C_image_Enlarge_SEC16B OE_Merge.tiff]

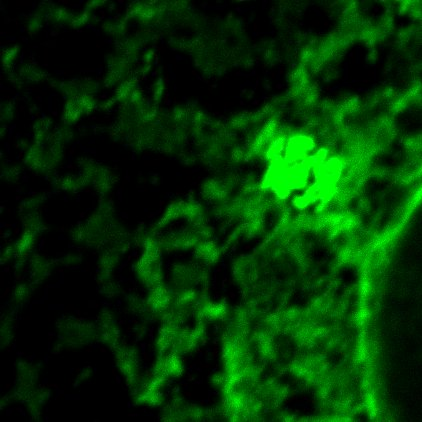

Supplement: Supplementary file 7 — Source data Fig. 4 [file 44318_2026_754_MOESM7_ESM.zip › Figure 4/4C/4C_image_Enlarge_SEC61B OE_GFP-SEC61B.tiff]

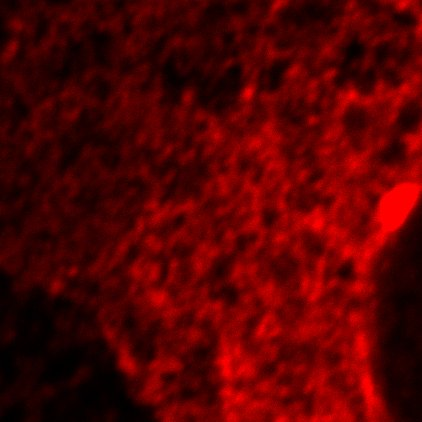

Supplement: Supplementary file 7 — Source data Fig. 4 [file 44318_2026_754_MOESM7_ESM.zip › Figure 4/4C/4C_image_Enlarge_SEC61B OE_HA-SEC13.tiff]

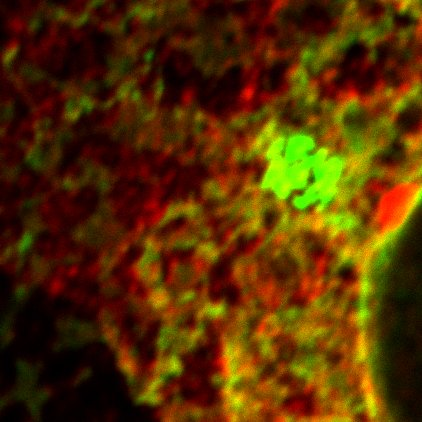

Supplement: Supplementary file 7 — Source data Fig. 4 [file 44318_2026_754_MOESM7_ESM.zip › Figure 4/4C/4C_image_Enlarge_SEC61B OE_Merge.tiff]

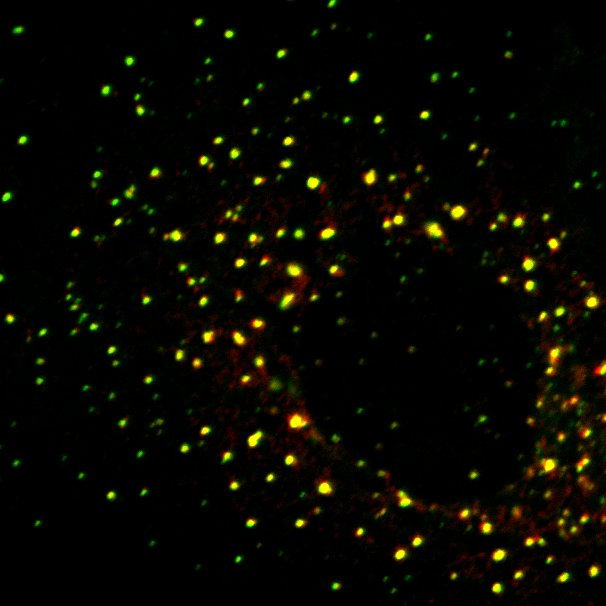

Supplement: Supplementary file 7 — Source data Fig. 4 [file 44318_2026_754_MOESM7_ESM.zip › Figure 4/4C/4C_image_SEC16B OE_Merge.tiff]

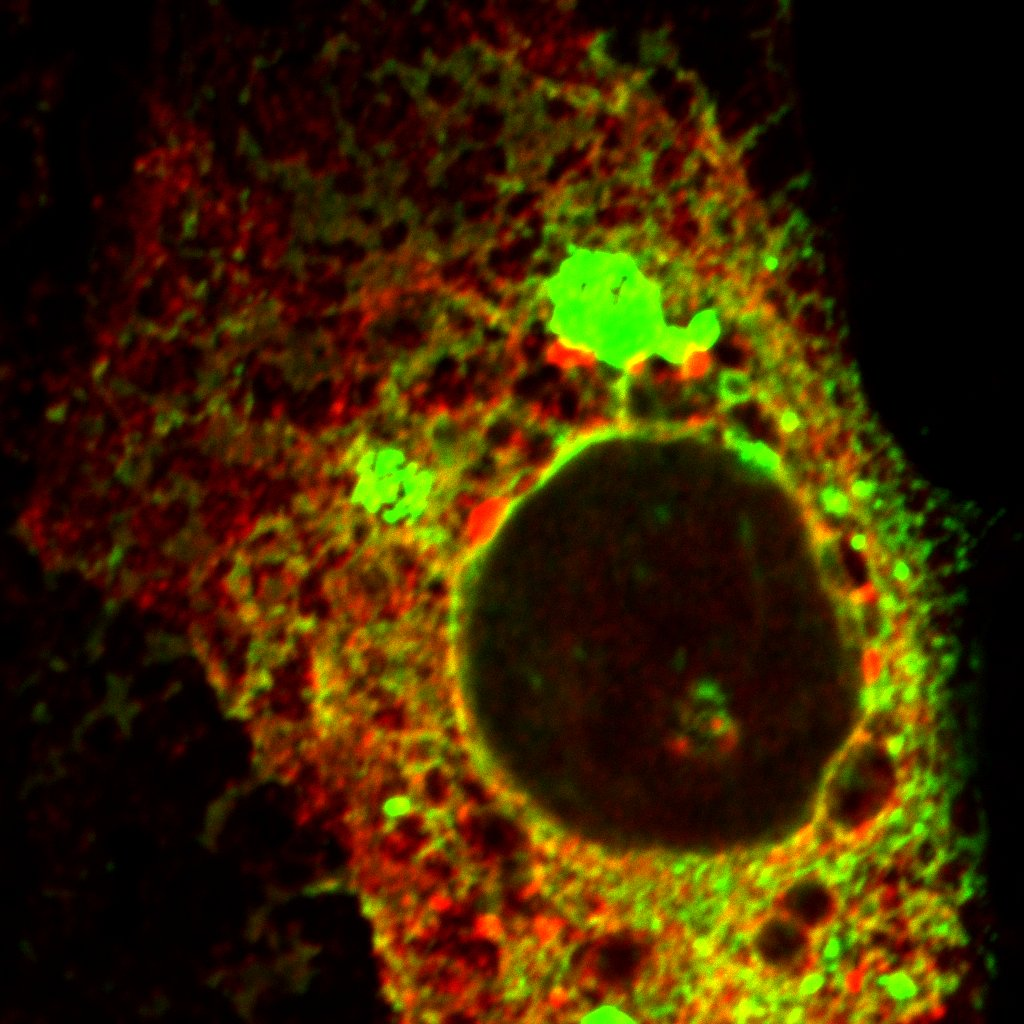

Supplement: Supplementary file 7 — Source data Fig. 4 [file 44318_2026_754_MOESM7_ESM.zip › Figure 4/4C/4C_image_SEC61B OE_Merge.tiff]

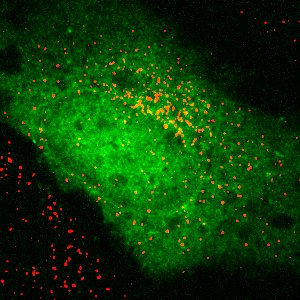

Supplement: Supplementary file 7 — Source data Fig. 4 [file 44318_2026_754_MOESM7_ESM.zip › Figure 4/4D/4D_image_CTL_Merge.tiff]

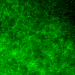

Supplement: Supplementary file 7 — Source data Fig. 4 [file 44318_2026_754_MOESM7_ESM.zip › Figure 4/4D/4D_image_Enlarge_CTL_GFP.tiff]

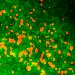

Supplement: Supplementary file 7 — Source data Fig. 4 [file 44318_2026_754_MOESM7_ESM.zip › Figure 4/4D/4D_image_Enlarge_CTL_Merge.tiff]

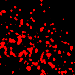

Supplement: Supplementary file 7 — Source data Fig. 4 [file 44318_2026_754_MOESM7_ESM.zip › Figure 4/4D/4D_image_Enlarge_CTL_SEC24A.tiff]

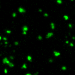

Supplement: Supplementary file 7 — Source data Fig. 4 [file 44318_2026_754_MOESM7_ESM.zip › Figure 4/4D/4D_image_Enlarge_SEC16B OE_GFP-16B.tiff]

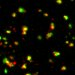

Supplement: Supplementary file 7 — Source data Fig. 4 [file 44318_2026_754_MOESM7_ESM.zip › Figure 4/4D/4D_image_Enlarge_SEC16B OE_Merge.tiff]

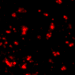

Supplement: Supplementary file 7 — Source data Fig. 4 [file 44318_2026_754_MOESM7_ESM.zip › Figure 4/4D/4D_image_Enlarge_SEC16B OE_SEC24A.tiff]

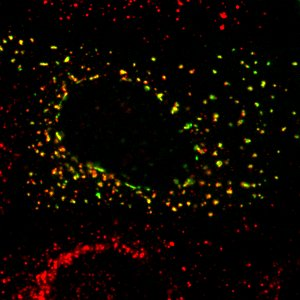

Supplement: Supplementary file 7 — Source data Fig. 4 [file 44318_2026_754_MOESM7_ESM.zip › Figure 4/4D/4D_image_SEC16B OE_Merge.tiff]

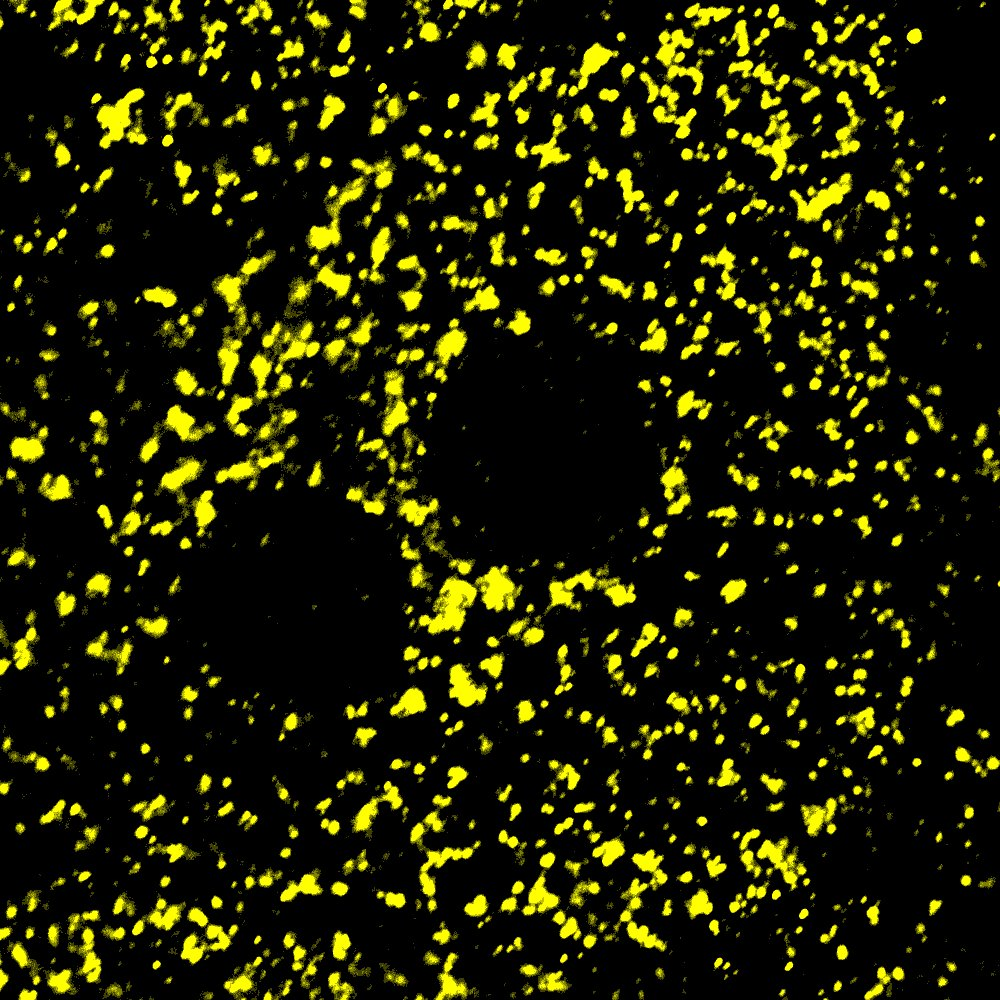

Supplement: Supplementary file 7 — Source data Fig. 4 [file 44318_2026_754_MOESM7_ESM.zip › Figure 4/4E-F/4E_image_LKO_BFA_SEC24A.tiff]

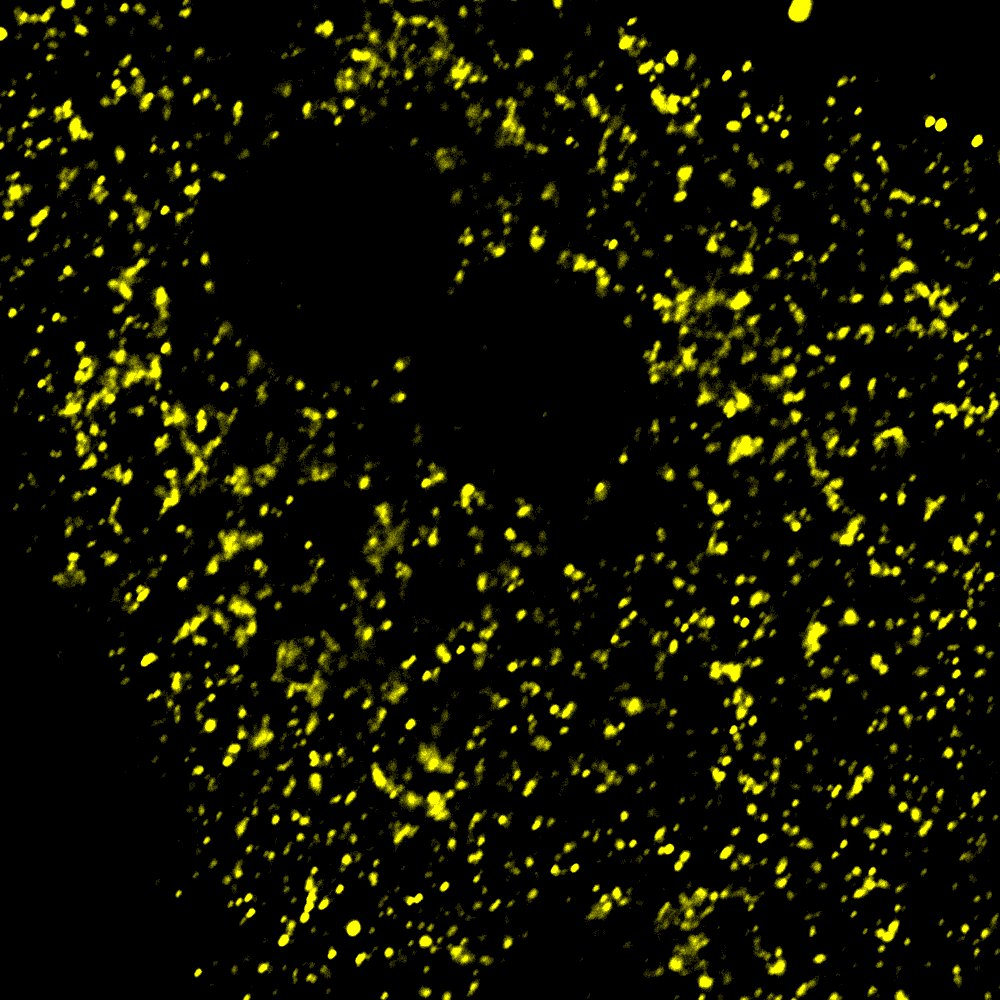

Supplement: Supplementary file 7 — Source data Fig. 4 [file 44318_2026_754_MOESM7_ESM.zip › Figure 4/4E-F/4E_image_LKO_SEC24A.tiff]

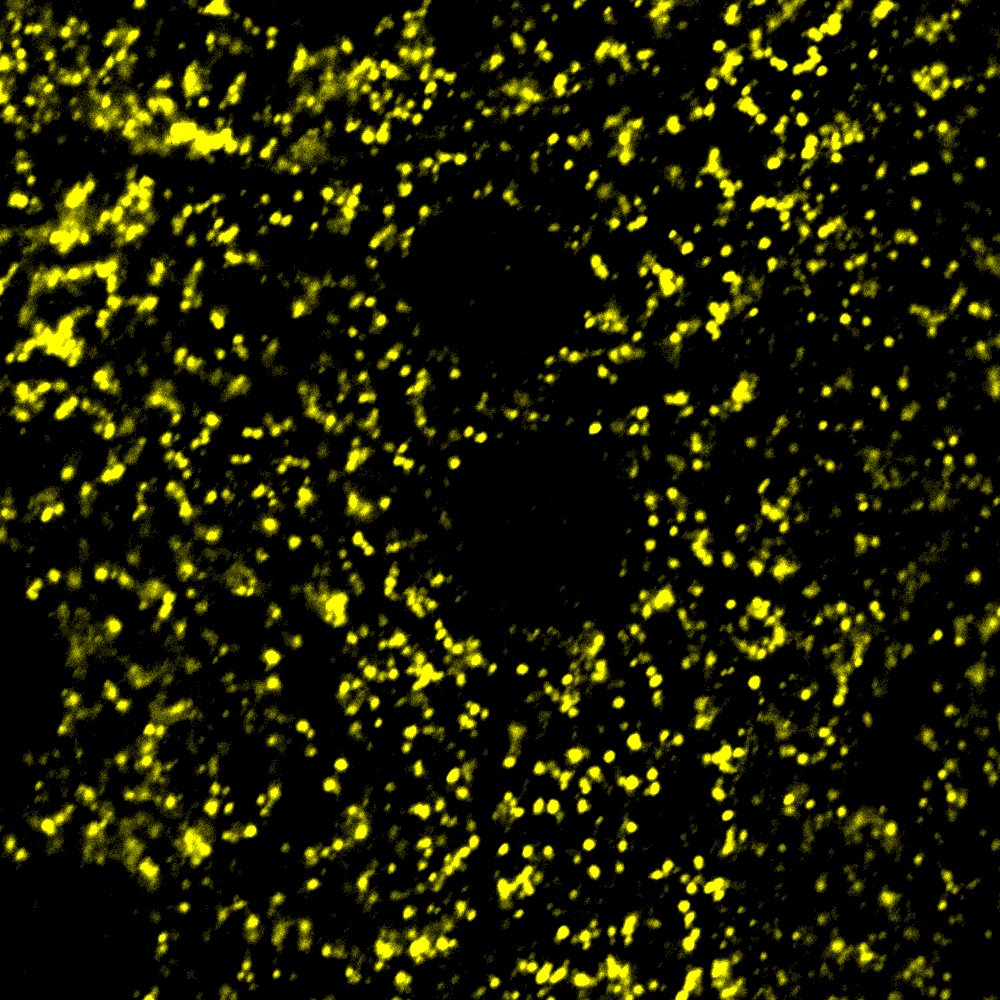

Supplement: Supplementary file 7 — Source data Fig. 4 [file 44318_2026_754_MOESM7_ESM.zip › Figure 4/4E-F/4E_image_WT_BFA_SEC24A.tiff]

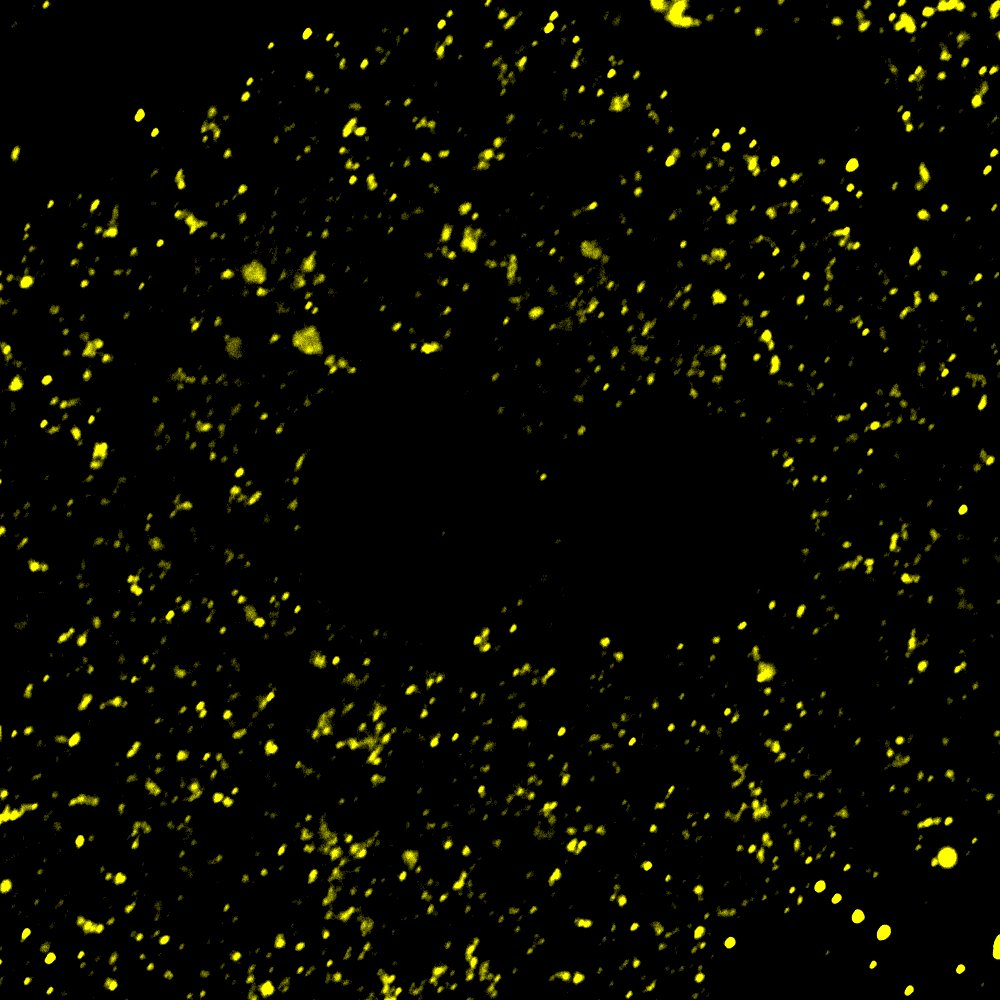

Supplement: Supplementary file 7 — Source data Fig. 4 [file 44318_2026_754_MOESM7_ESM.zip › Figure 4/4E-F/4E_image_WT_SEC24A.tiff]

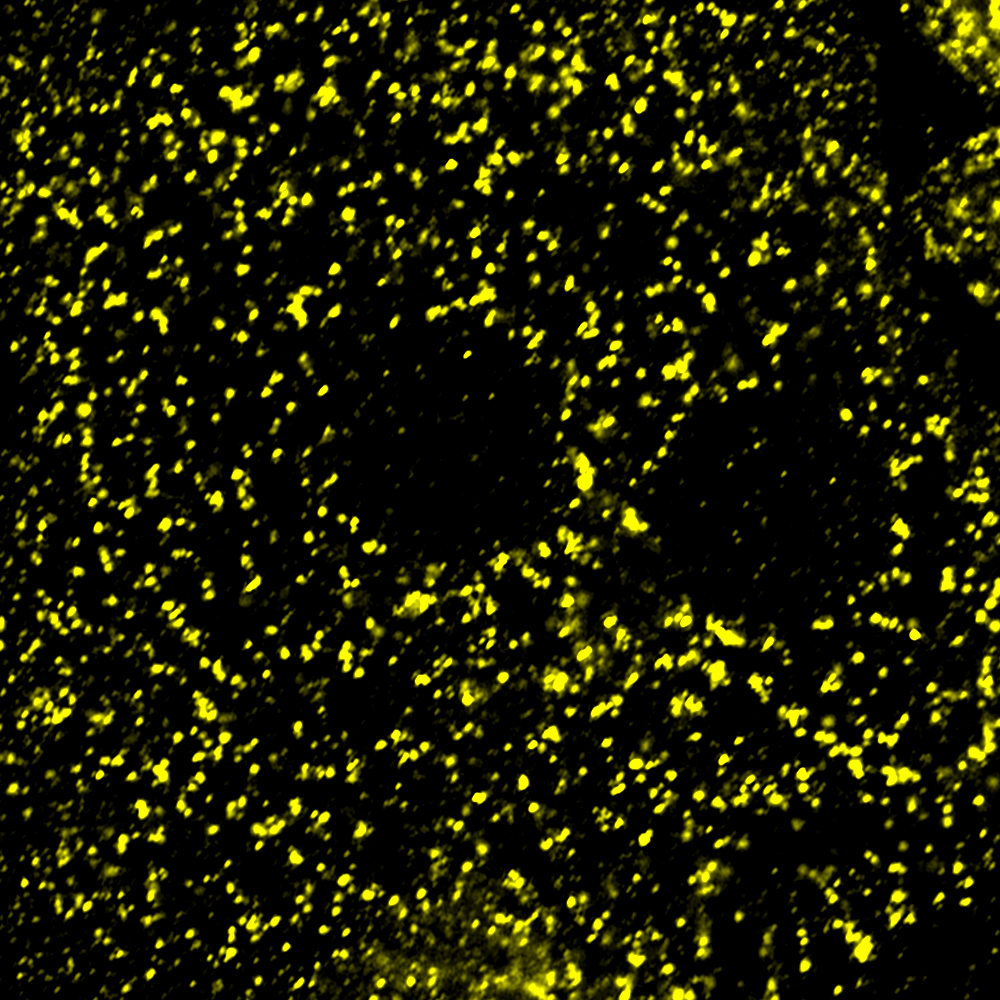

Supplement: Supplementary file 7 — Source data Fig. 4 [file 44318_2026_754_MOESM7_ESM.zip › Figure 4/4G-H/4G_image_LKO_BFA_SEC31A.tiff]

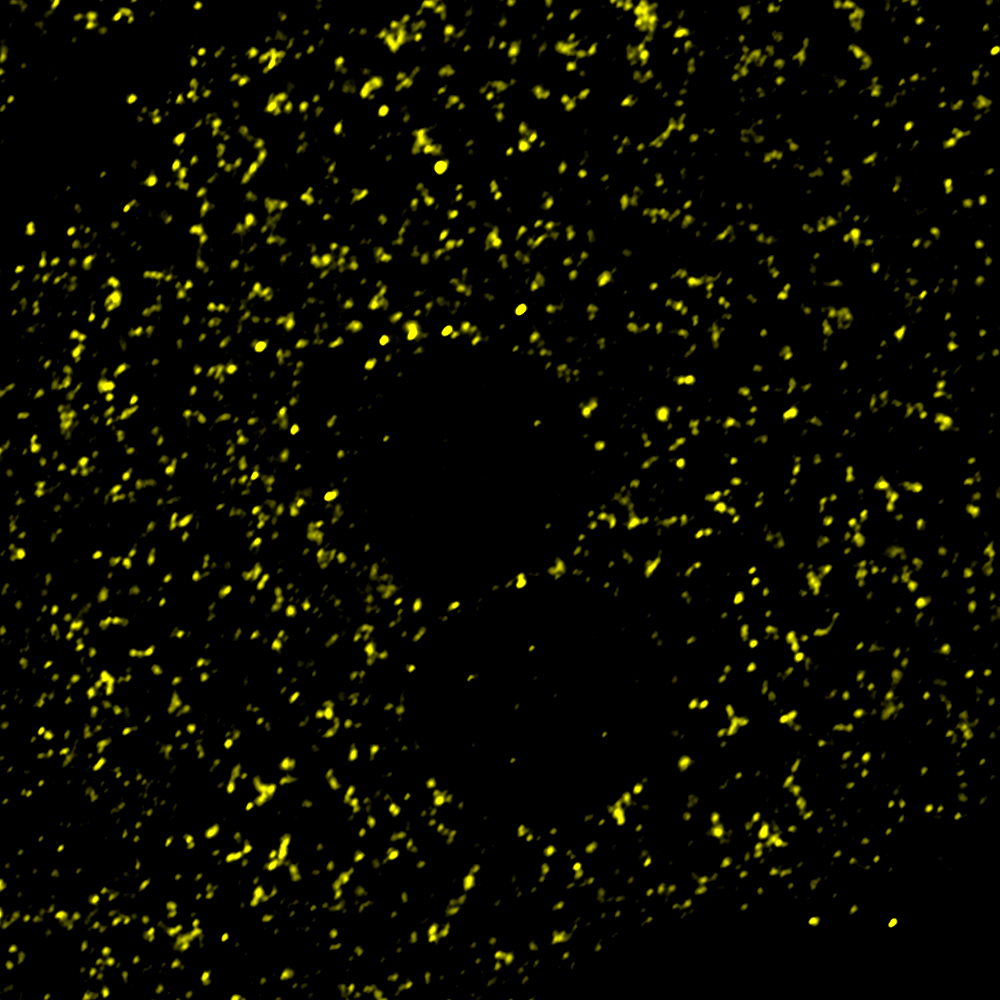

Supplement: Supplementary file 7 — Source data Fig. 4 [file 44318_2026_754_MOESM7_ESM.zip › Figure 4/4G-H/4G_image_LKO_SEC31A.tiff]

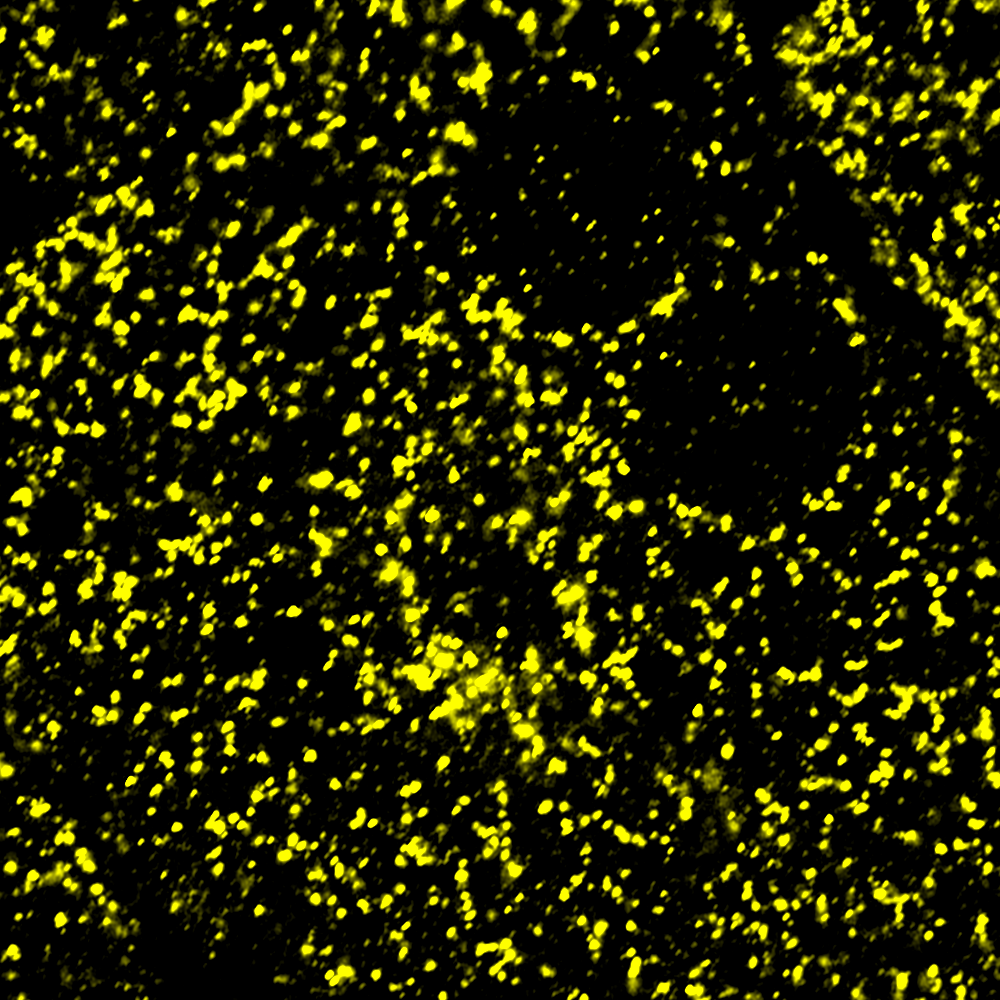

Supplement: Supplementary file 7 — Source data Fig. 4 [file 44318_2026_754_MOESM7_ESM.zip › Figure 4/4G-H/4G_image_WT_BFA_SEC31A.tiff]

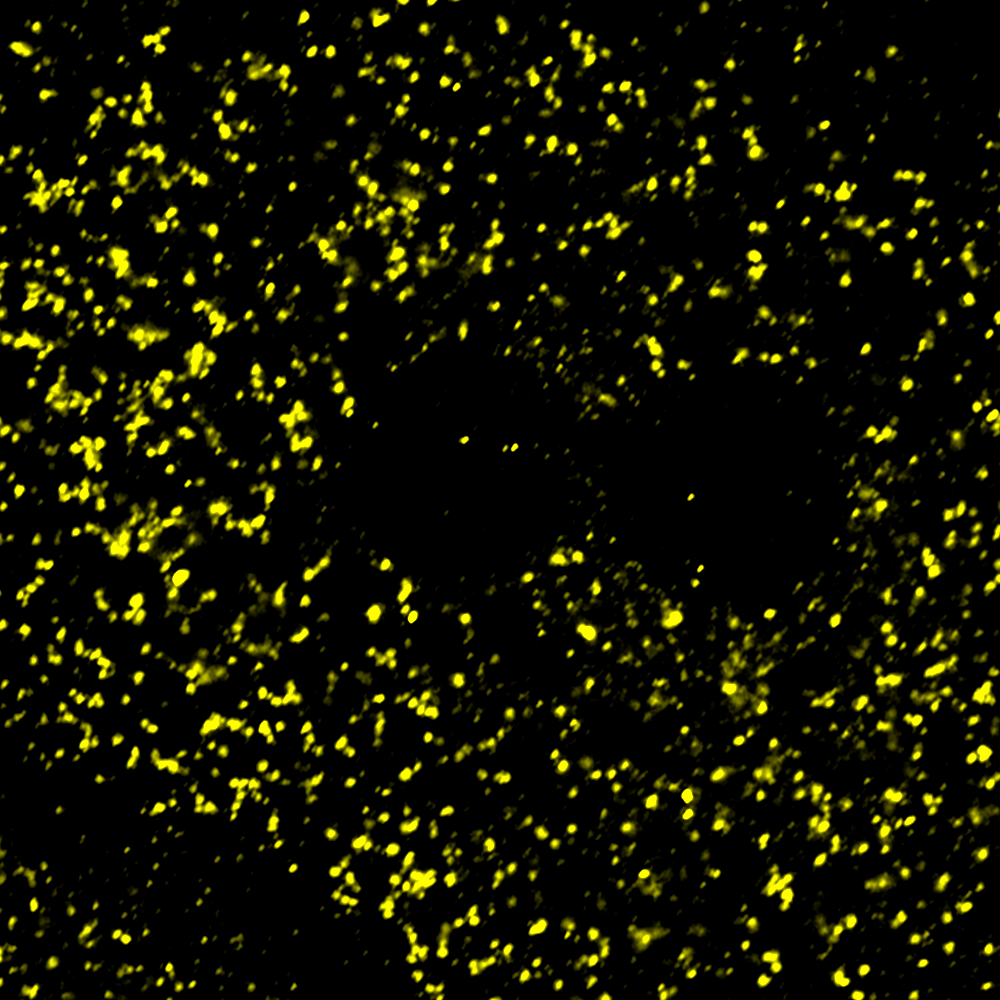

Supplement: Supplementary file 7 — Source data Fig. 4 [file 44318_2026_754_MOESM7_ESM.zip › Figure 4/4G-H/4G_image_WT_SEC31A.tiff]

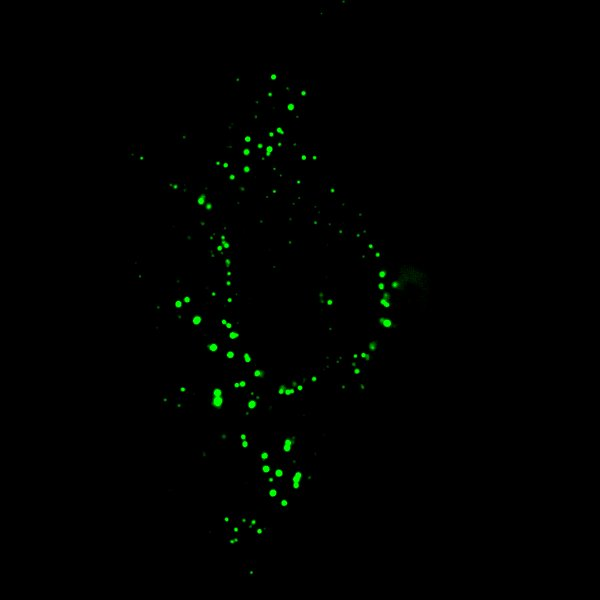

Supplement: Supplementary file 8 — Source data Fig. 5 [file 44318_2026_754_MOESM8_ESM.zip › Figure 5/5A/5A_image_lower_GFP-SEC31A.tiff]

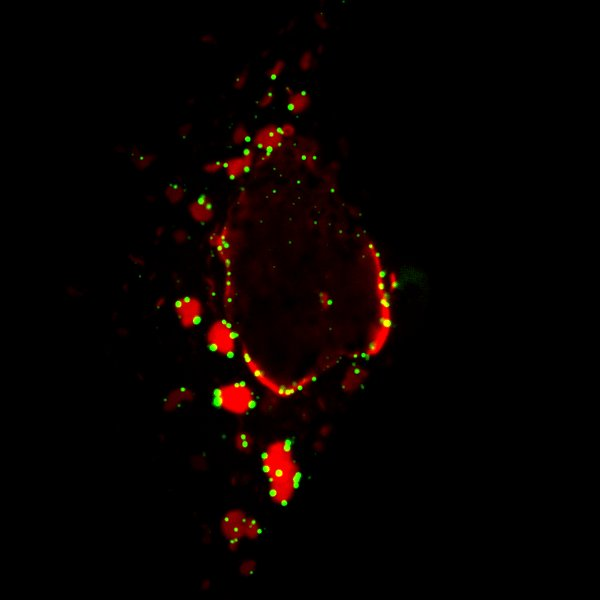

Supplement: Supplementary file 8 — Source data Fig. 5 [file 44318_2026_754_MOESM8_ESM.zip › Figure 5/5A/5A_image_lower_merge.tiff]

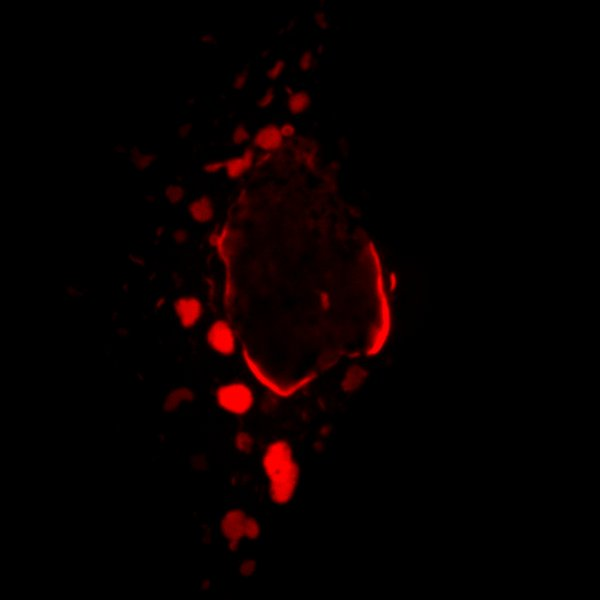

Supplement: Supplementary file 8 — Source data Fig. 5 [file 44318_2026_754_MOESM8_ESM.zip › Figure 5/5A/5A_image_lower_RFP-SEC16B dCCD.tiff]

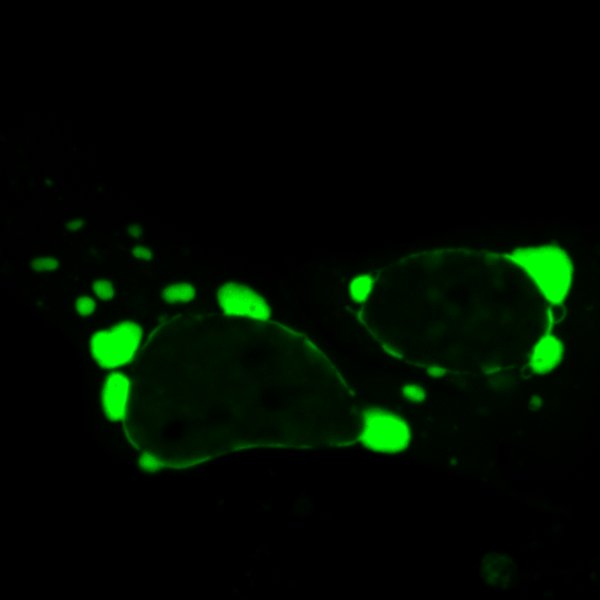

Supplement: Supplementary file 8 — Source data Fig. 5 [file 44318_2026_754_MOESM8_ESM.zip › Figure 5/5A/5A_image_middel_GFP-SEC24A.tiff]

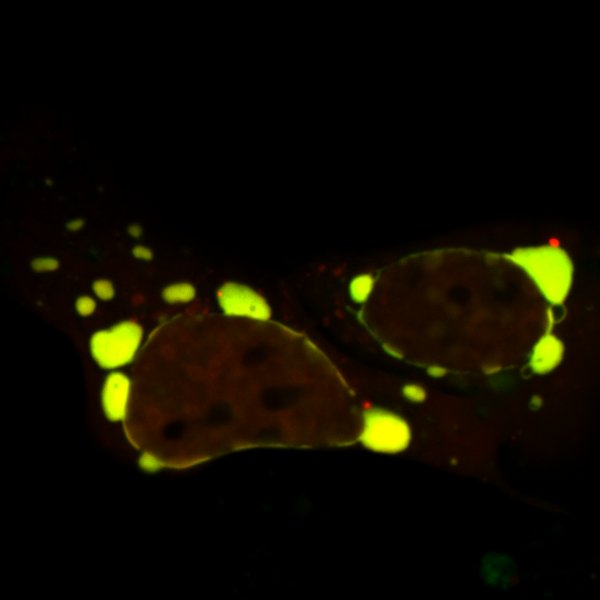

Supplement: Supplementary file 8 — Source data Fig. 5 [file 44318_2026_754_MOESM8_ESM.zip › Figure 5/5A/5A_image_middel_merge.tiff]

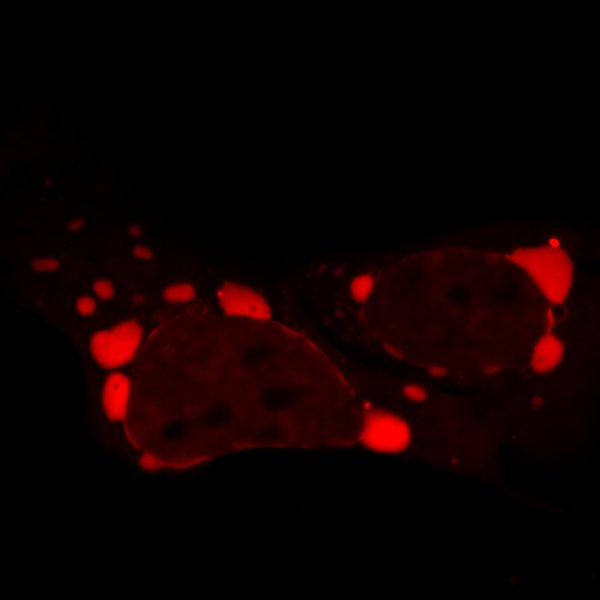

Supplement: Supplementary file 8 — Source data Fig. 5 [file 44318_2026_754_MOESM8_ESM.zip › Figure 5/5A/5A_image_middel_RFP-dCCD SEC16B.tiff]

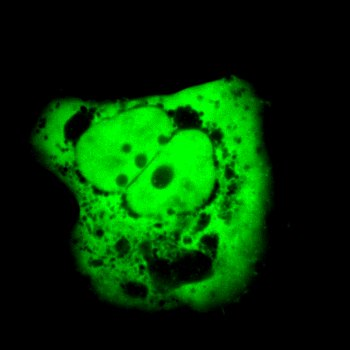

Supplement: Supplementary file 8 — Source data Fig. 5 [file 44318_2026_754_MOESM8_ESM.zip › Figure 5/5A/5A_image_upper_GFP.tiff]

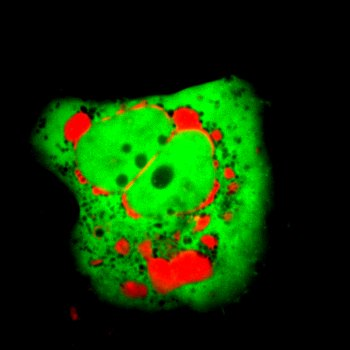

Supplement: Supplementary file 8 — Source data Fig. 5 [file 44318_2026_754_MOESM8_ESM.zip › Figure 5/5A/5A_image_upper_merge.tiff]

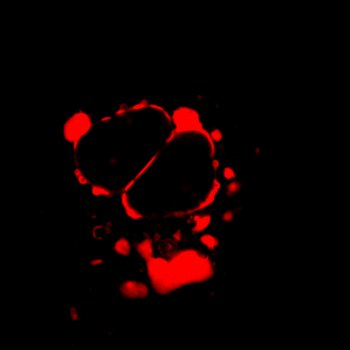

Supplement: Supplementary file 8 — Source data Fig. 5 [file 44318_2026_754_MOESM8_ESM.zip › Figure 5/5A/5A_image_upper_RFP-SEC16B dCCD.tiff]

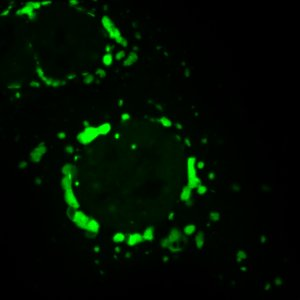

Supplement: Supplementary file 8 — Source data Fig. 5 [file 44318_2026_754_MOESM8_ESM.zip › Figure 5/5B-C/5B_image_lower_GFP-SEC16B dCCD.tiff]

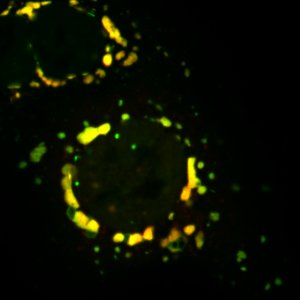

Supplement: Supplementary file 8 — Source data Fig. 5 [file 44318_2026_754_MOESM8_ESM.zip › Figure 5/5B-C/5B_image_lower_merge.tiff]

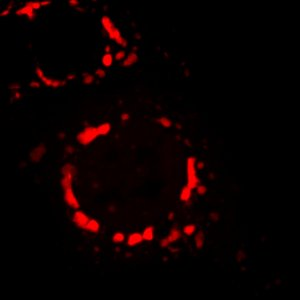

Supplement: Supplementary file 8 — Source data Fig. 5 [file 44318_2026_754_MOESM8_ESM.zip › Figure 5/5B-C/5B_image_lower_RFP-FUS_IDR-SEC24.tiff]

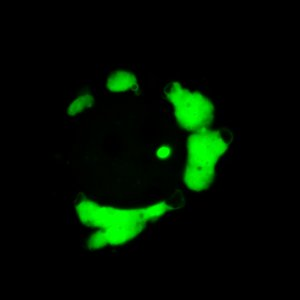

Supplement: Supplementary file 8 — Source data Fig. 5 [file 44318_2026_754_MOESM8_ESM.zip › Figure 5/5B-C/5B_image_middle_GFP-SEC16B dCCD.tiff]

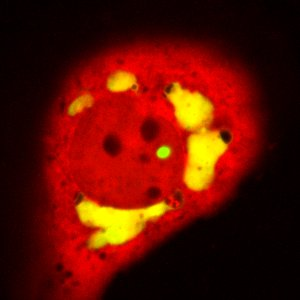

Supplement: Supplementary file 8 — Source data Fig. 5 [file 44318_2026_754_MOESM8_ESM.zip › Figure 5/5B-C/5B_image_middle_merge.tiff]

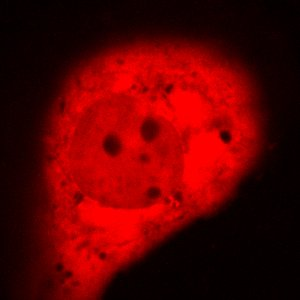

Supplement: Supplementary file 8 — Source data Fig. 5 [file 44318_2026_754_MOESM8_ESM.zip › Figure 5/5B-C/5B_image_middle_RFP-dIDR SEC24.tiff]

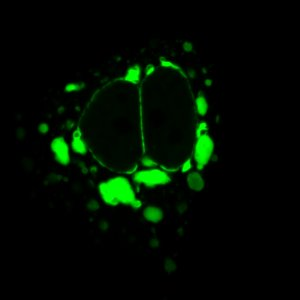

Supplement: Supplementary file 8 — Source data Fig. 5 [file 44318_2026_754_MOESM8_ESM.zip › Figure 5/5B-C/5B_image_upper_GFP-SEC16B dCCD.tiff]

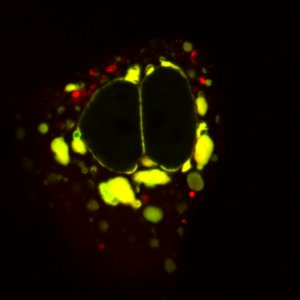

Supplement: Supplementary file 8 — Source data Fig. 5 [file 44318_2026_754_MOESM8_ESM.zip › Figure 5/5B-C/5B_image_upper_merge.tiff]

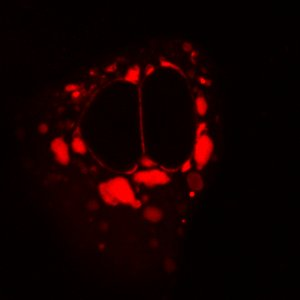

Supplement: Supplementary file 8 — Source data Fig. 5 [file 44318_2026_754_MOESM8_ESM.zip › Figure 5/5B-C/5B_image_upper_RFP-SEC24.tiff]

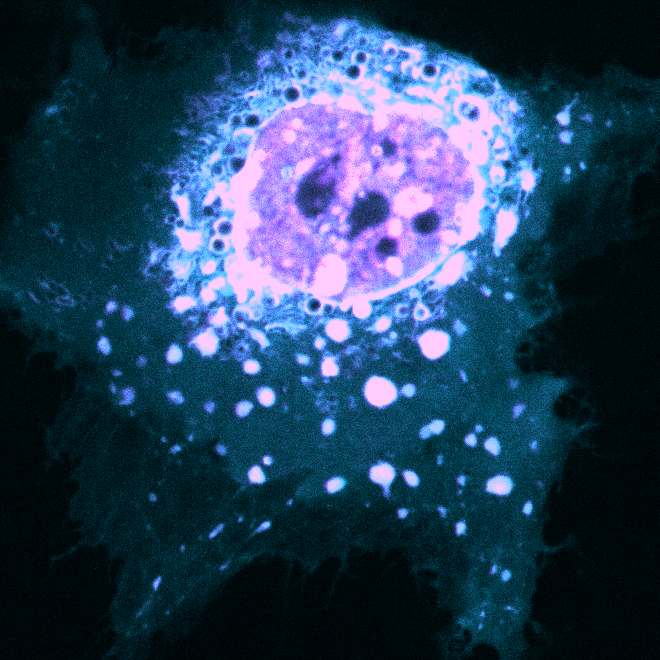

Supplement: Supplementary file 8 — Source data Fig. 5 [file 44318_2026_754_MOESM8_ESM.zip › Figure 5/5E/5E_image_After_merge.tiff]

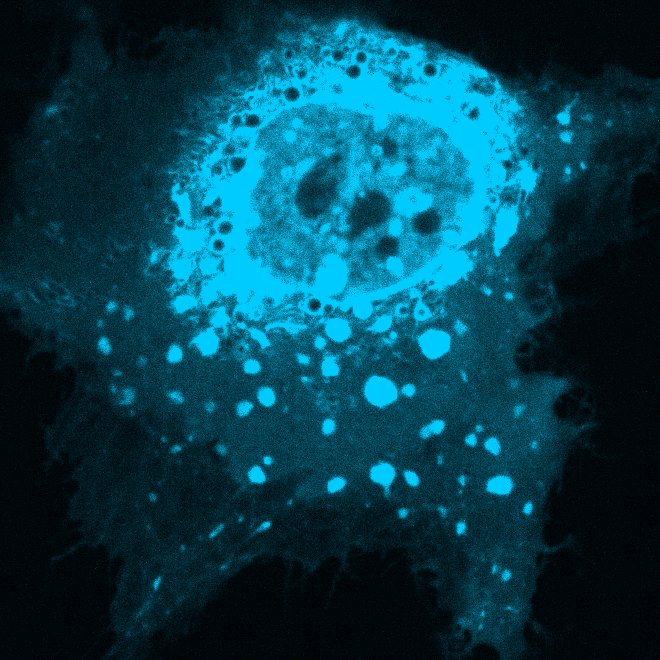

Supplement: Supplementary file 8 — Source data Fig. 5 [file 44318_2026_754_MOESM8_ESM.zip › Figure 5/5E/5E_image_After_SEC16B_CT.tiff]

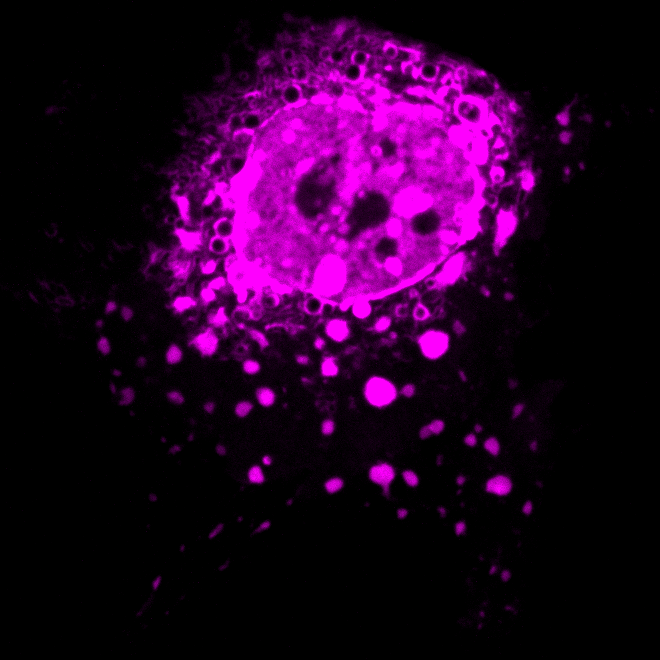

Supplement: Supplementary file 8 — Source data Fig. 5 [file 44318_2026_754_MOESM8_ESM.zip › Figure 5/5E/5E_image_After_SEC16B_NT.tiff]

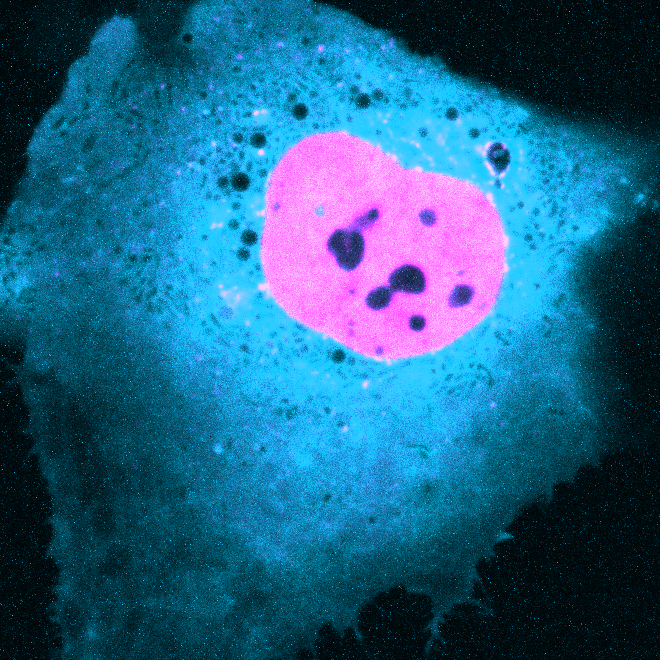

Supplement: Supplementary file 8 — Source data Fig. 5 [file 44318_2026_754_MOESM8_ESM.zip › Figure 5/5E/5E_image_Before_merge.tiff]

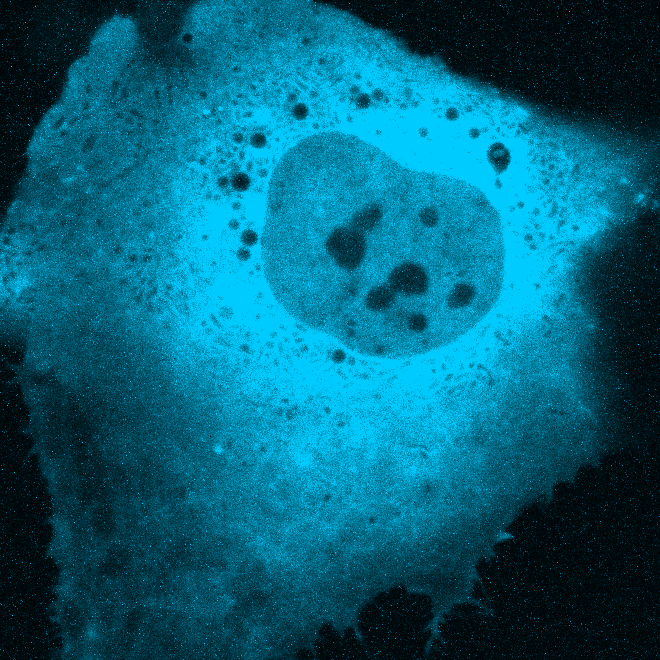

Supplement: Supplementary file 8 — Source data Fig. 5 [file 44318_2026_754_MOESM8_ESM.zip › Figure 5/5E/5E_image_Before_SEC16B_CT.tiff]

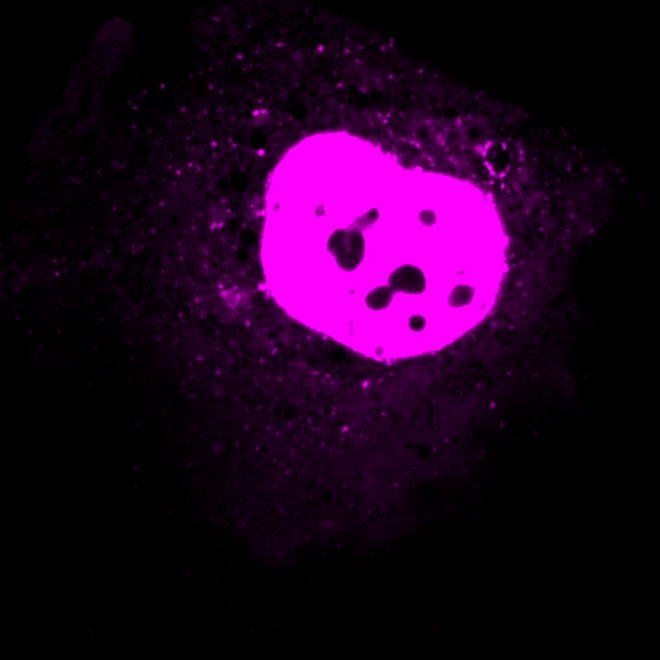

Supplement: Supplementary file 8 — Source data Fig. 5 [file 44318_2026_754_MOESM8_ESM.zip › Figure 5/5E/5E_image_Before_SEC16B_NT.tiff]
