# Supplementary figures and images for: Tissue-selective COPII modulator SEC16B aggravates cardiovascular disease by promoting lipid export (part 2 of 3)
Source: EMBO J. 2026 Apr 24;45(11):3731–62. doi: 10.1038/s44318-026-00754-8 (PMC13226660; doi:10.1038/s44318-026-00754-8)

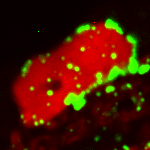

Supplement: Supplementary file 8 — Source data Fig. 5 [file 44318_2026_754_MOESM8_ESM.zip › Figure 5/5F/5F_image_115min_merge.tif]

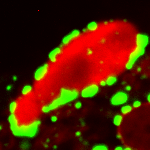

Supplement: Supplementary file 8 — Source data Fig. 5 [file 44318_2026_754_MOESM8_ESM.zip › Figure 5/5F/5F_image_190min_merge.tif]

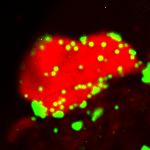

Supplement: Supplementary file 8 — Source data Fig. 5 [file 44318_2026_754_MOESM8_ESM.zip › Figure 5/5F/5F_image_40min _merge.tif]

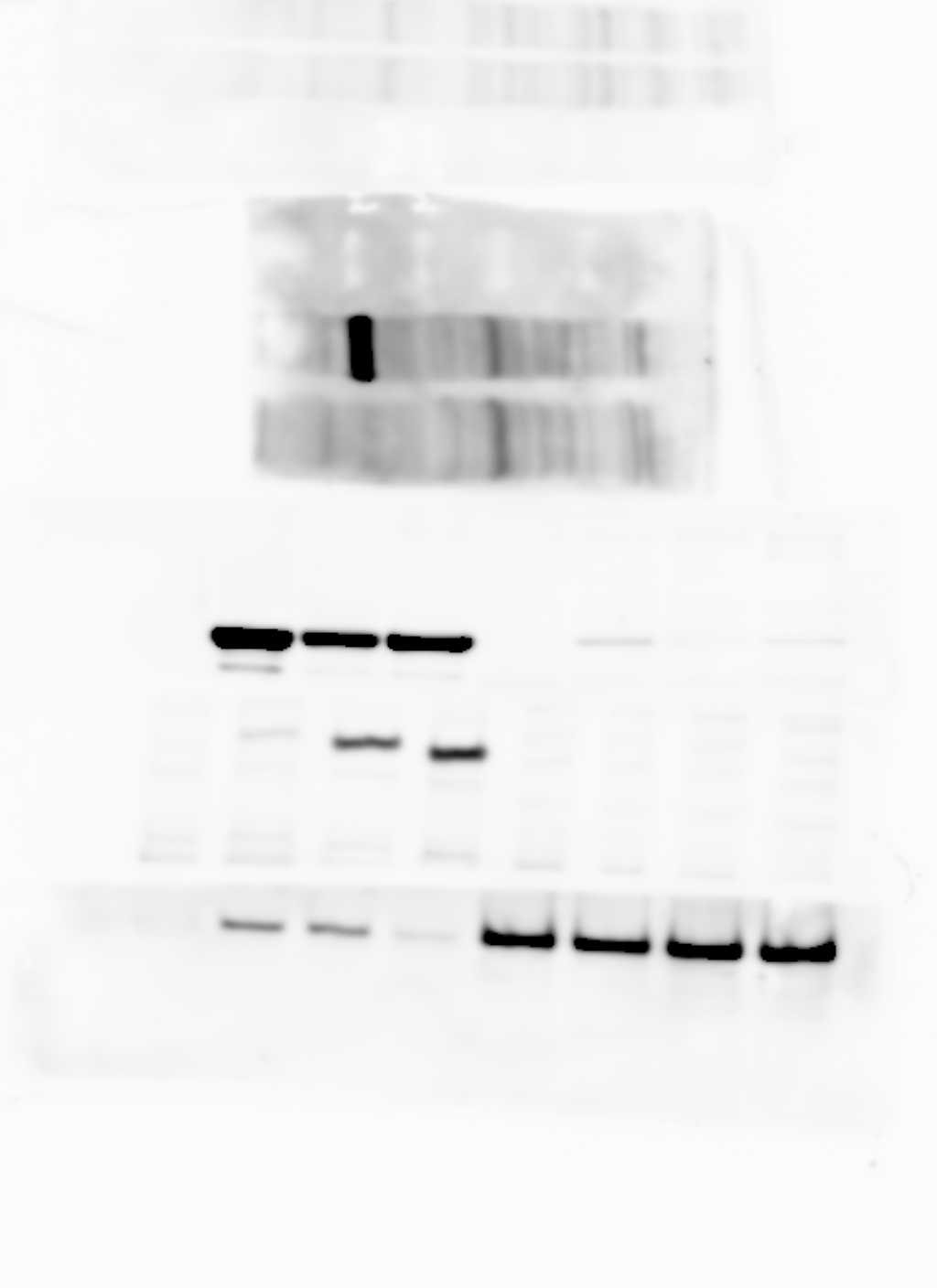

Supplement: Supplementary file 8 — Source data Fig. 5 [file 44318_2026_754_MOESM8_ESM.zip › Figure 5/5G/5G_western_SAR1B (FLAG).tif]

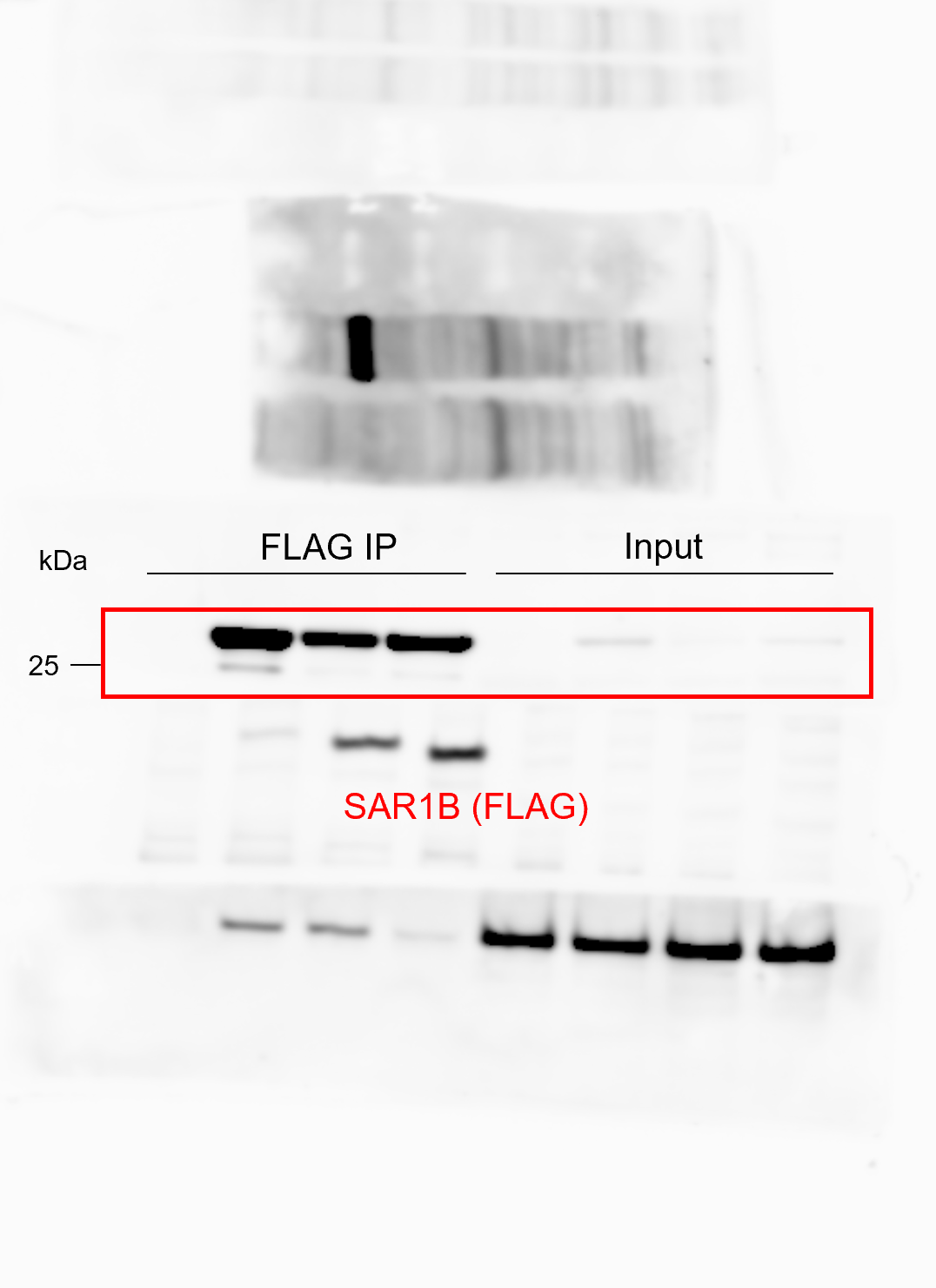

Supplement: Supplementary file 8 — Source data Fig. 5 [file 44318_2026_754_MOESM8_ESM.zip › Figure 5/5G/5G_western_SAR1B (FLAG)_label.tif]

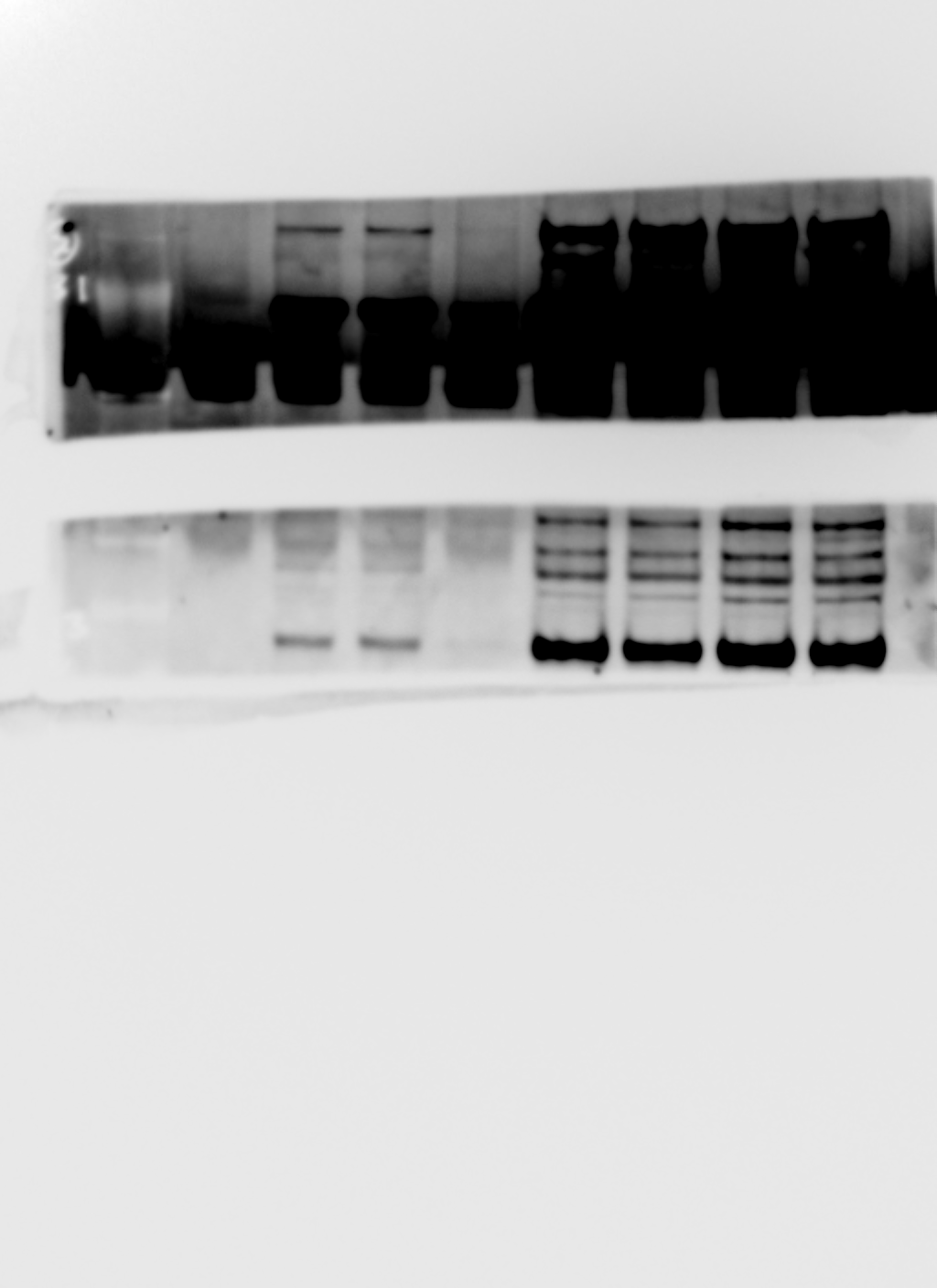

Supplement: Supplementary file 8 — Source data Fig. 5 [file 44318_2026_754_MOESM8_ESM.zip › Figure 5/5G/5G_western_SEC13.tif]

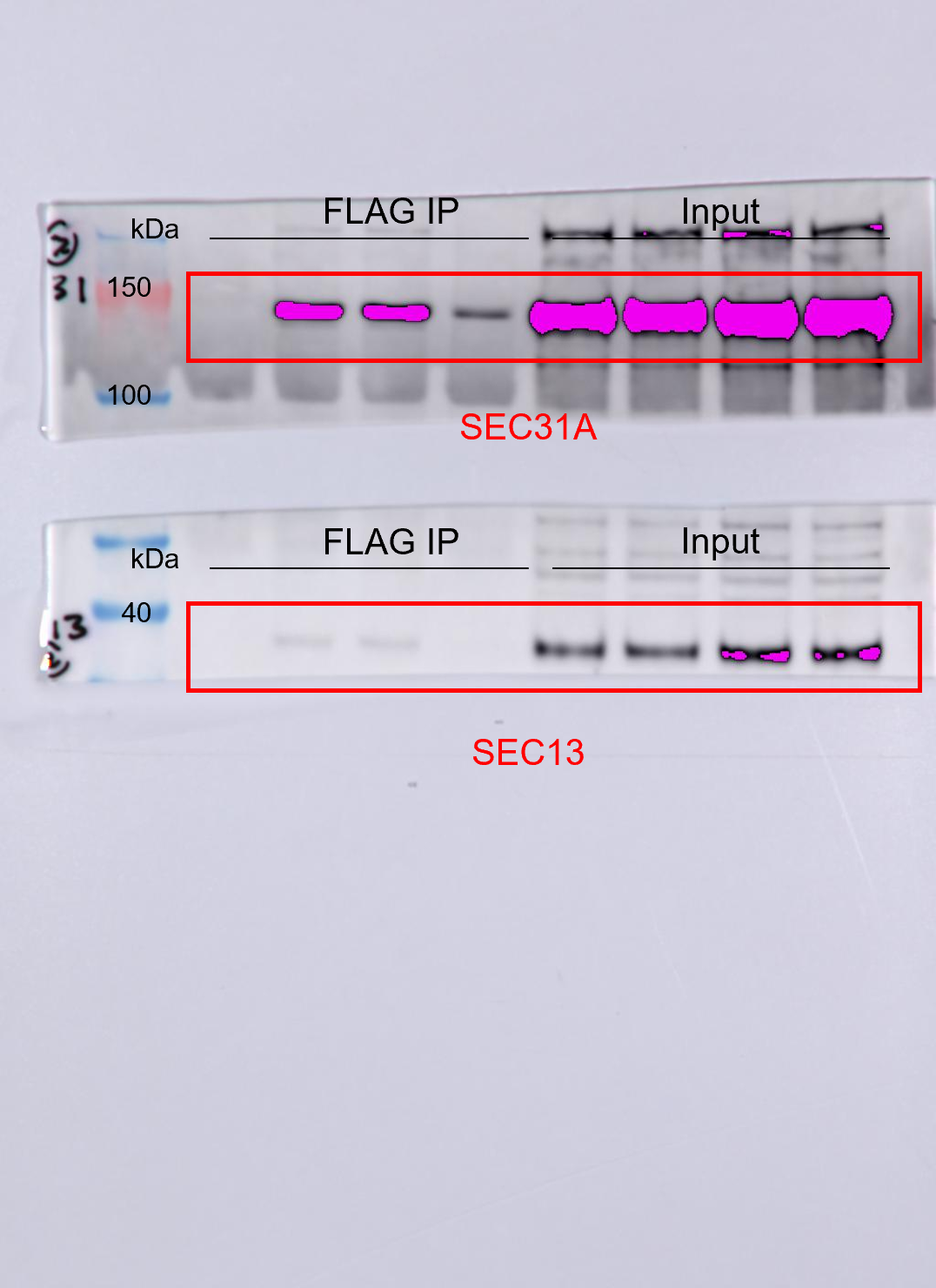

Supplement: Supplementary file 8 — Source data Fig. 5 [file 44318_2026_754_MOESM8_ESM.zip › Figure 5/5G/5G_western_SEC13_label.tif]

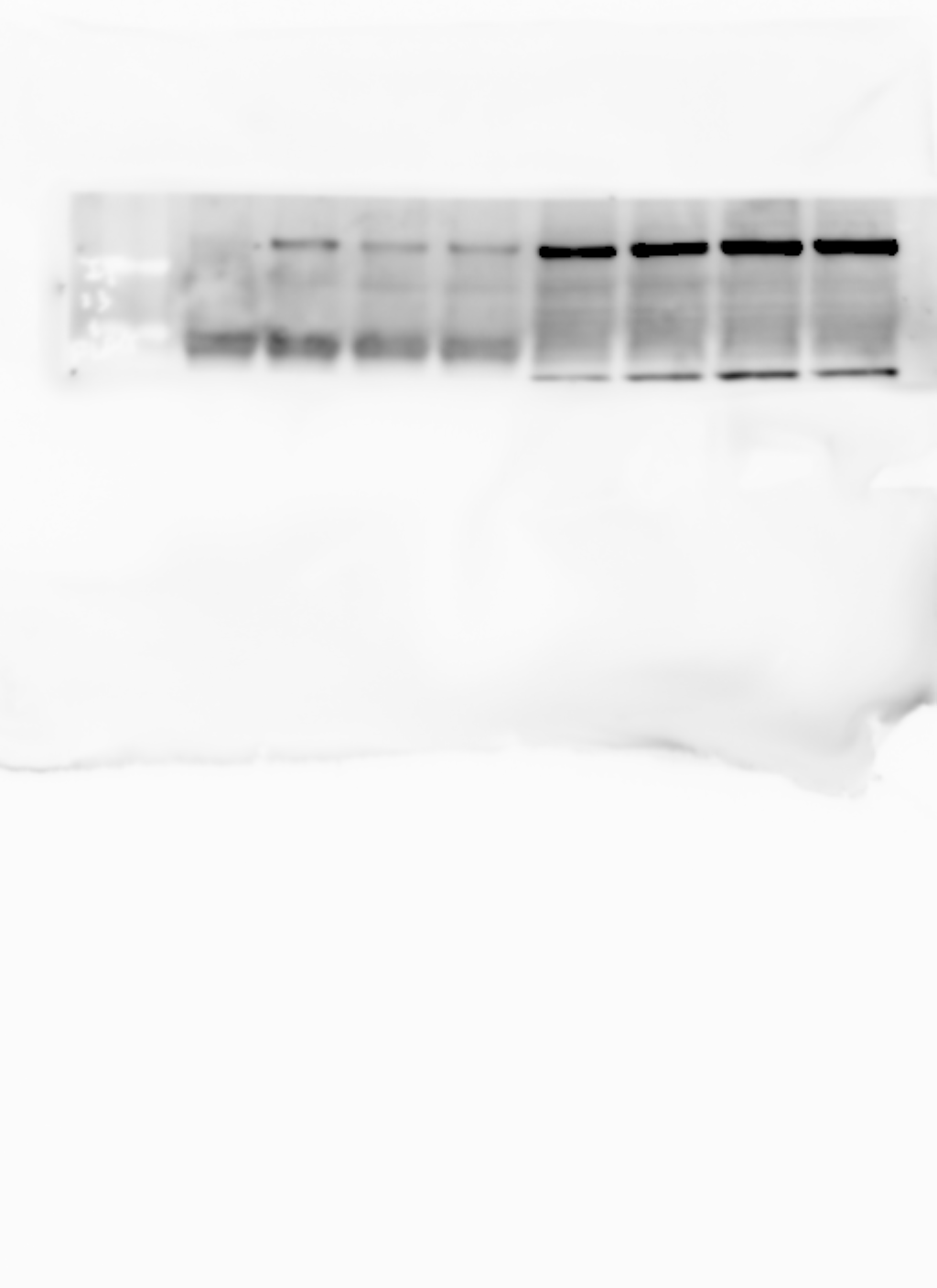

Supplement: Supplementary file 8 — Source data Fig. 5 [file 44318_2026_754_MOESM8_ESM.zip › Figure 5/5G/5G_western_SEC23A.tif]

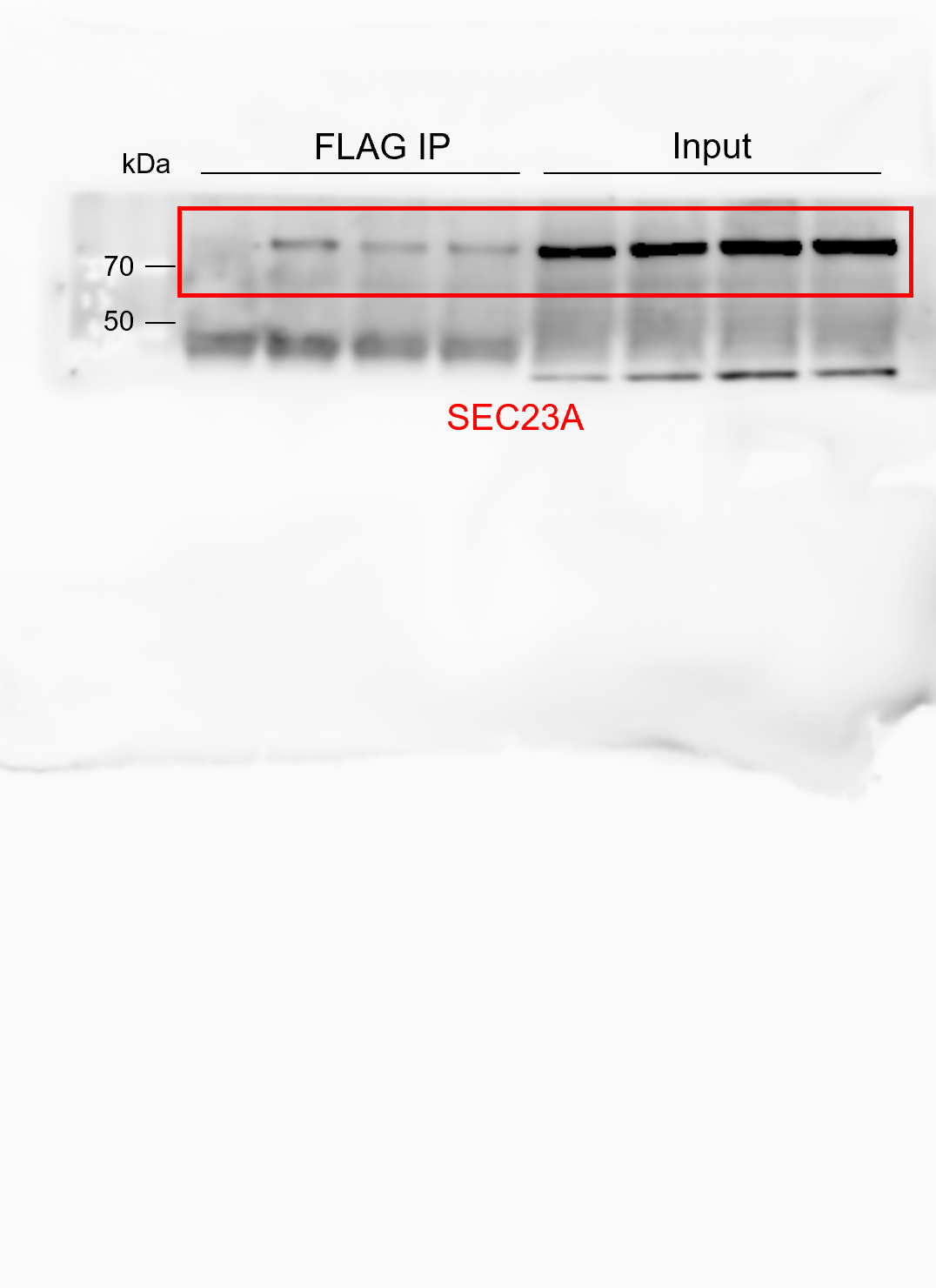

Supplement: Supplementary file 8 — Source data Fig. 5 [file 44318_2026_754_MOESM8_ESM.zip › Figure 5/5G/5G_western_SEC23A_label.tif]

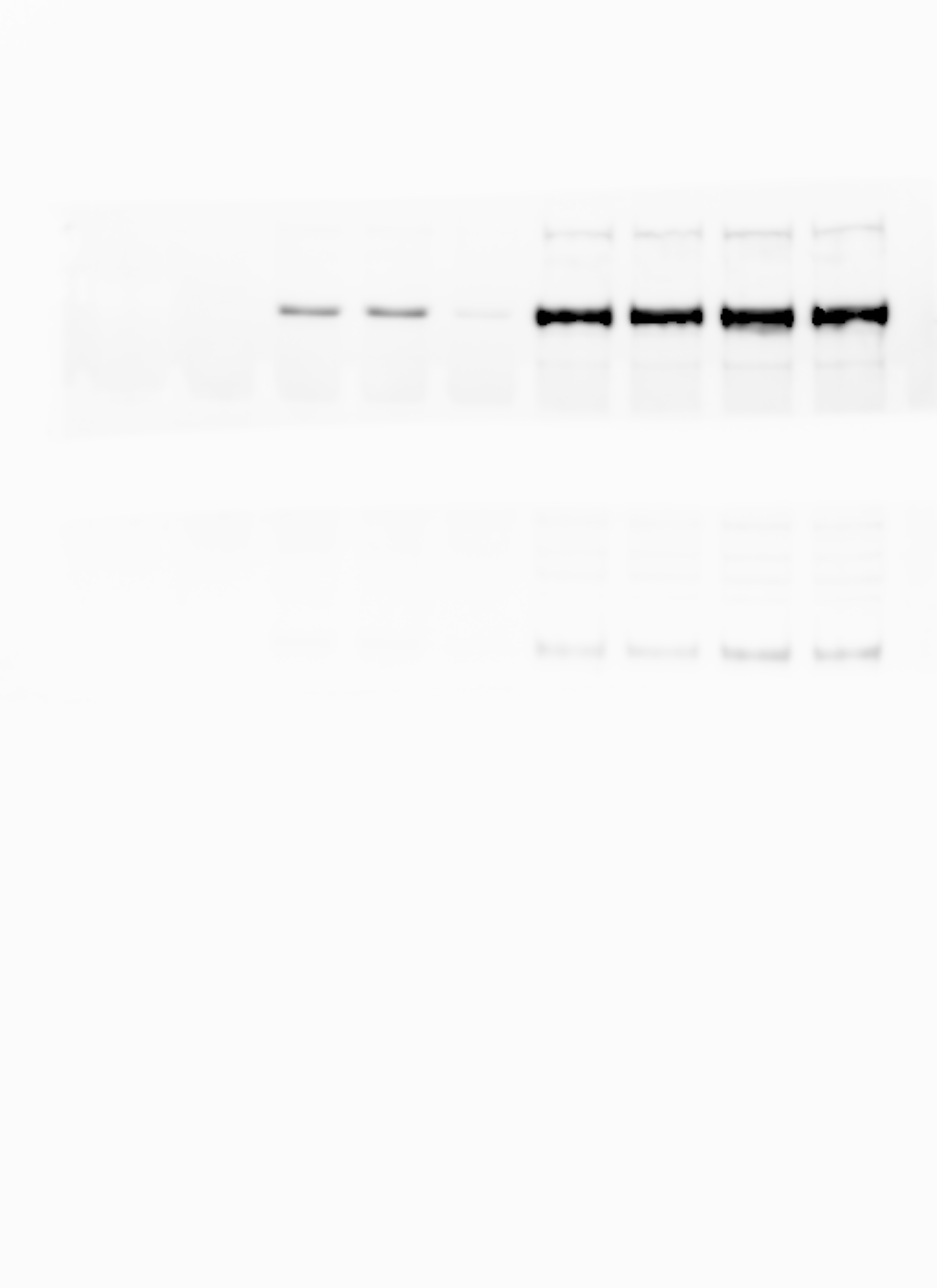

Supplement: Supplementary file 8 — Source data Fig. 5 [file 44318_2026_754_MOESM8_ESM.zip › Figure 5/5G/5G_western_SEC31A.tif]

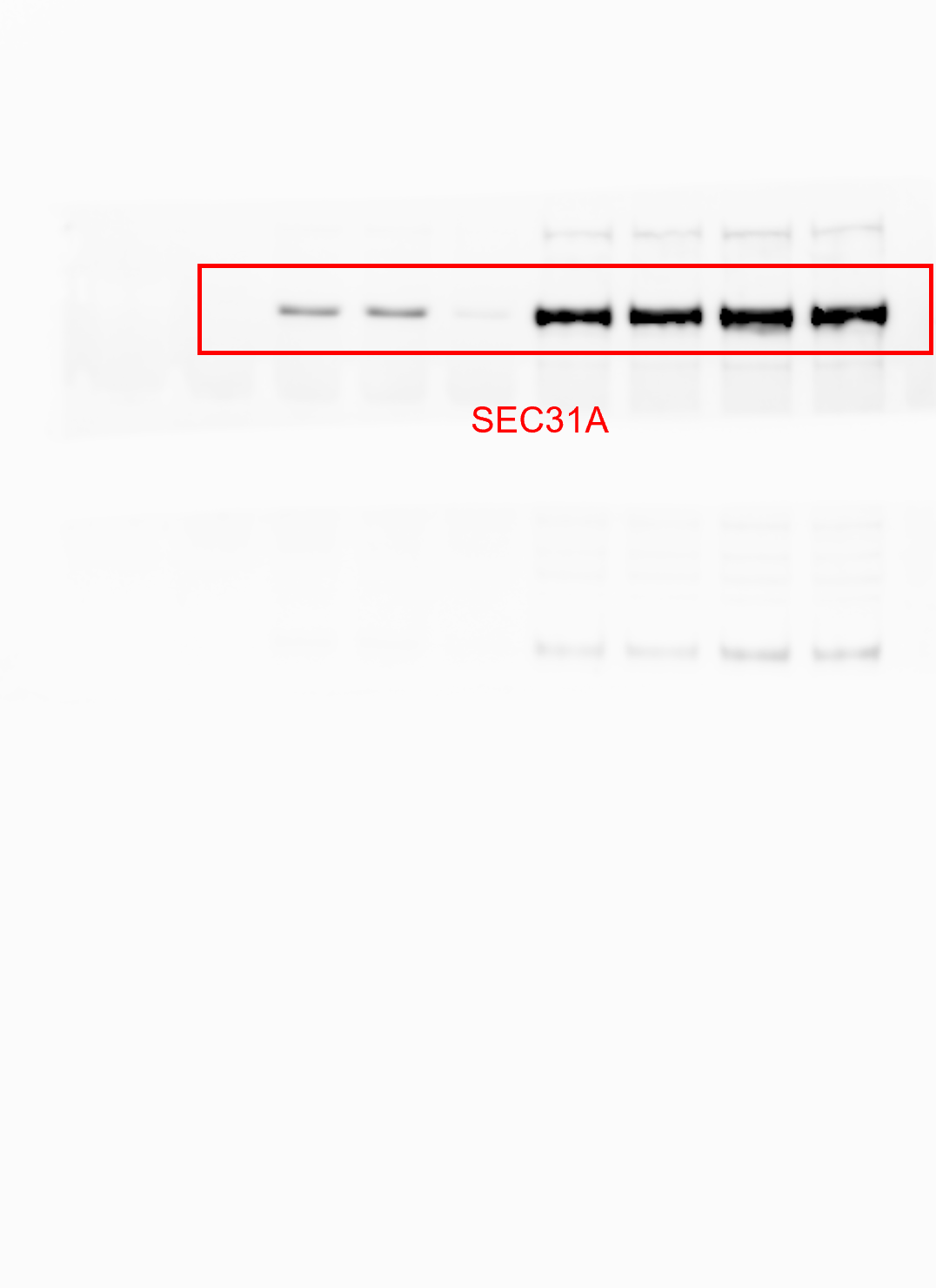

Supplement: Supplementary file 8 — Source data Fig. 5 [file 44318_2026_754_MOESM8_ESM.zip › Figure 5/5G/5G_western_SEC31A_label.tif]

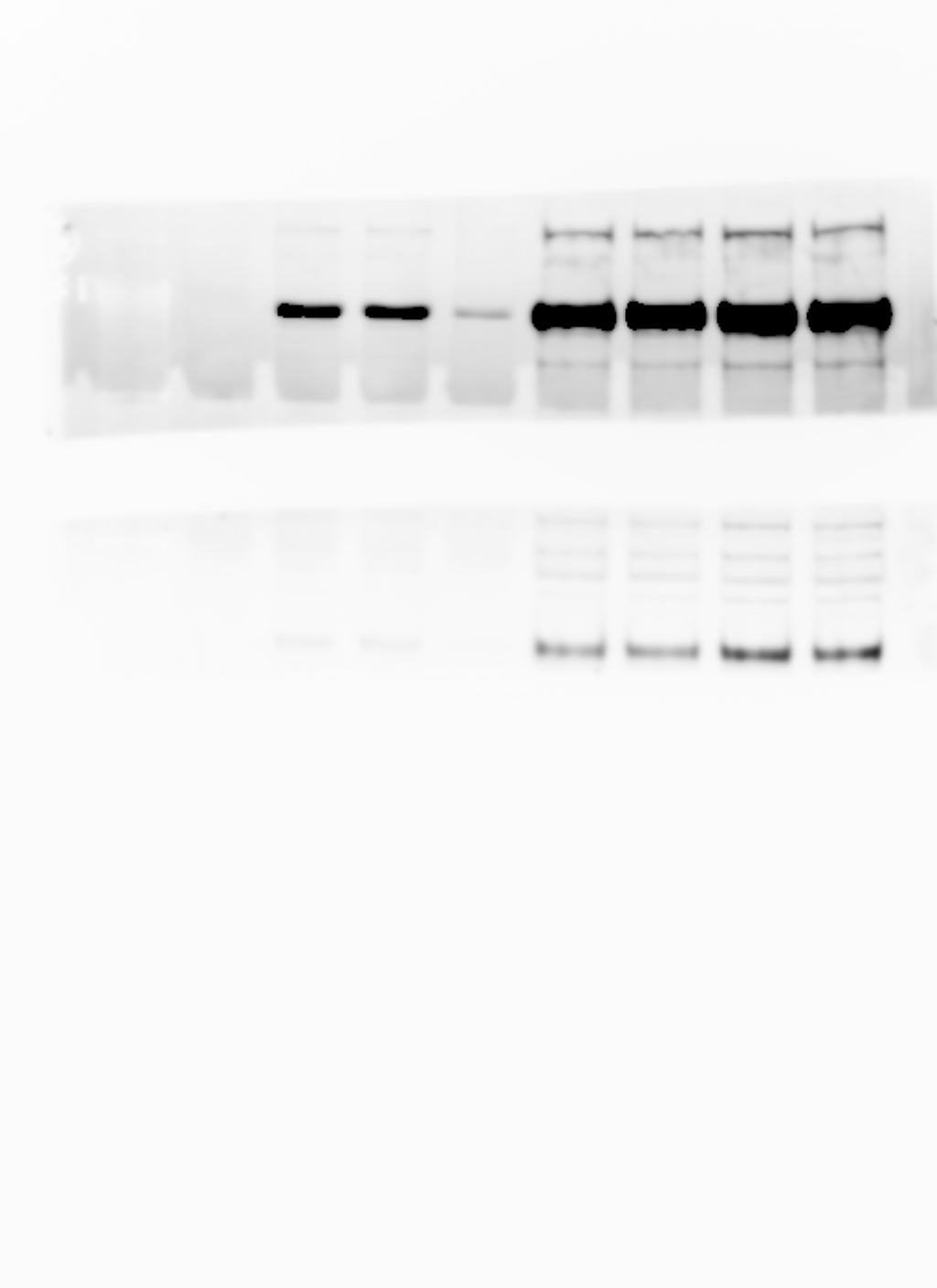

Supplement: Supplementary file 8 — Source data Fig. 5 [file 44318_2026_754_MOESM8_ESM.zip › Figure 5/5G/XWC FLAG IP 31 13 2019.01.30_18.51.55_Ch.tif]

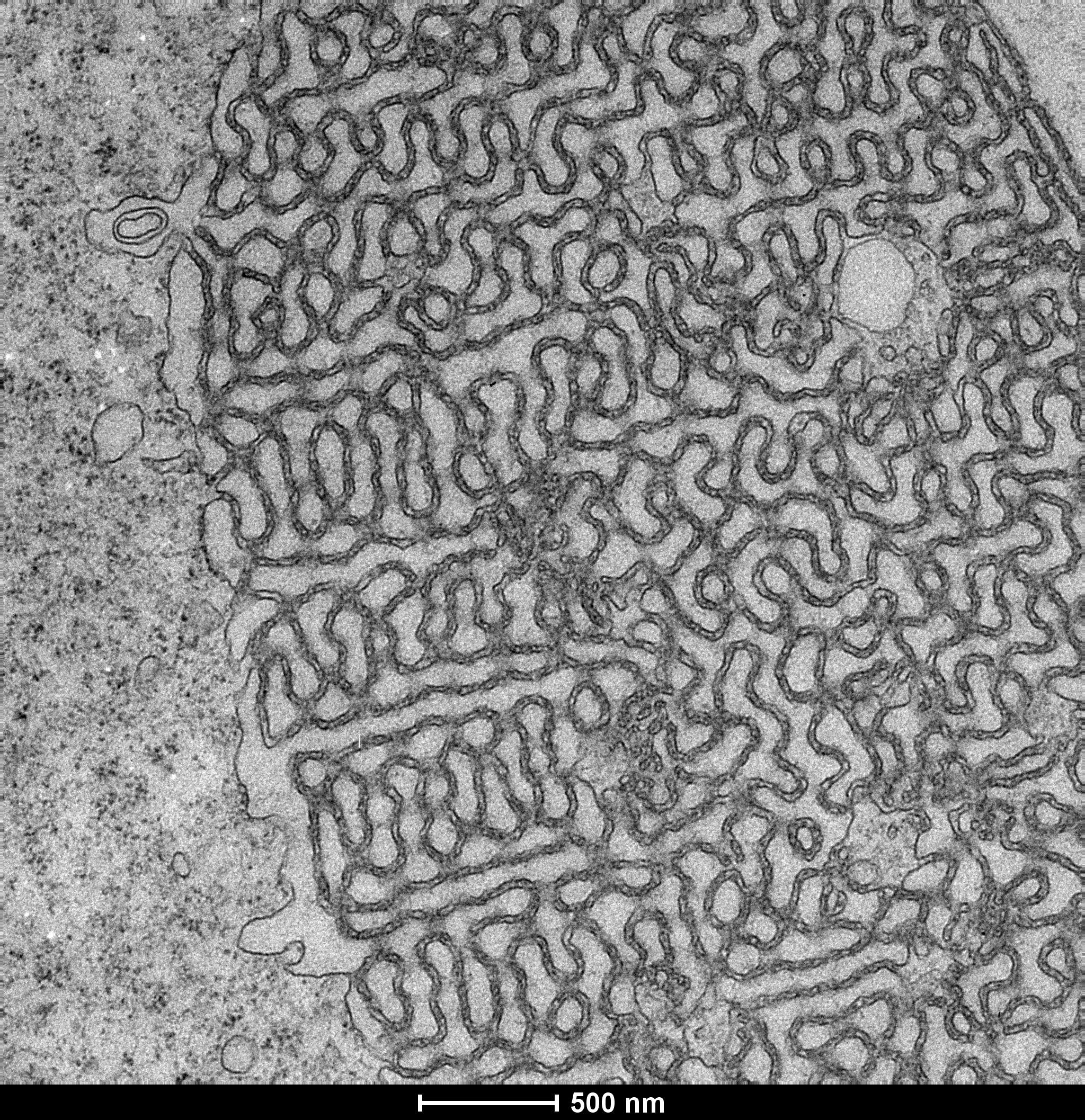

Supplement: Supplementary file 8 — Source data Fig. 5 [file 44318_2026_754_MOESM8_ESM.zip › Figure 5/5H/5H_EM_image.tif]

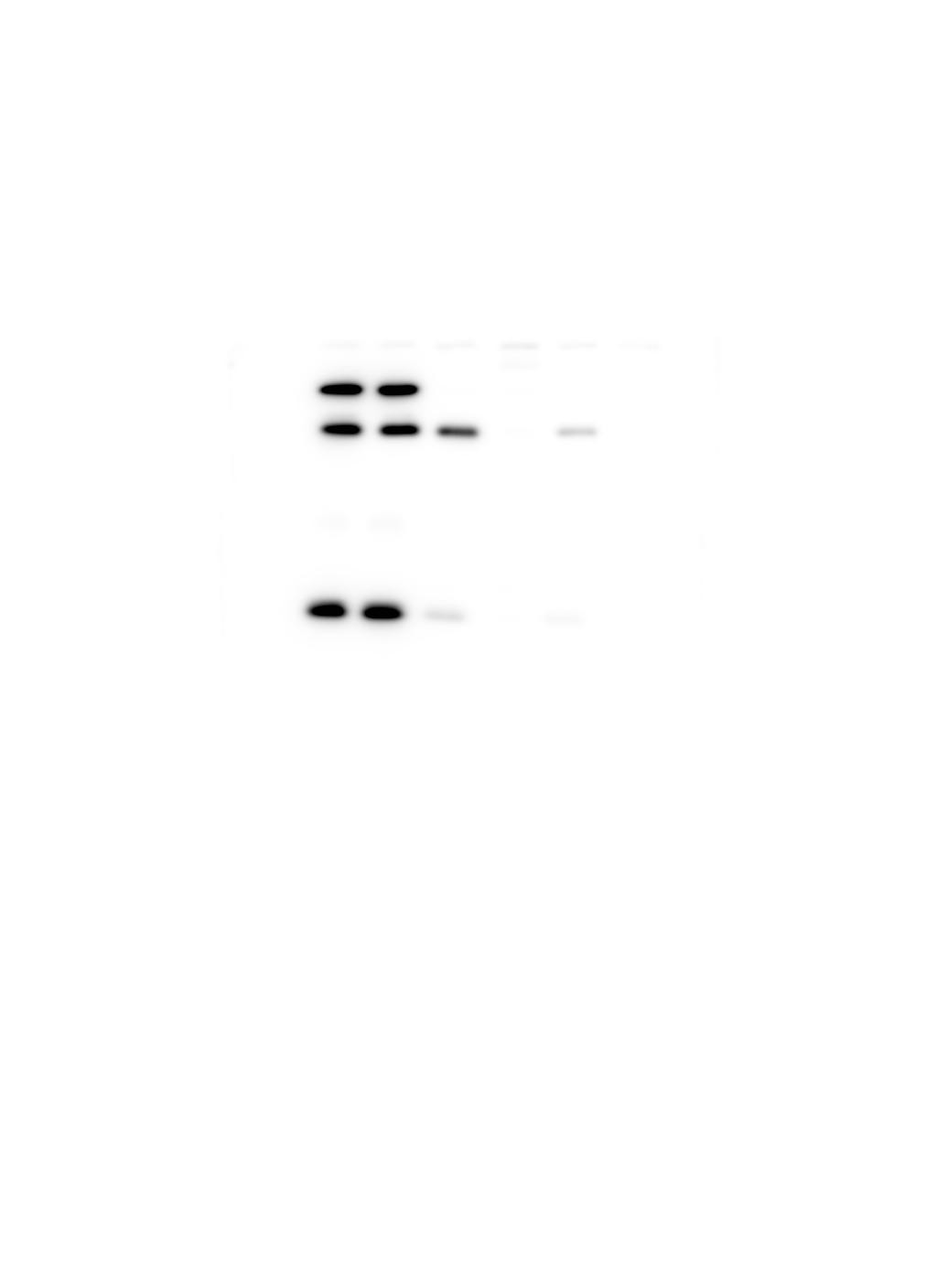

Supplement: Supplementary file 8 — Source data Fig. 5 [file 44318_2026_754_MOESM8_ESM.zip › Figure 5/5I/5I_western_LMAN1.jpg]

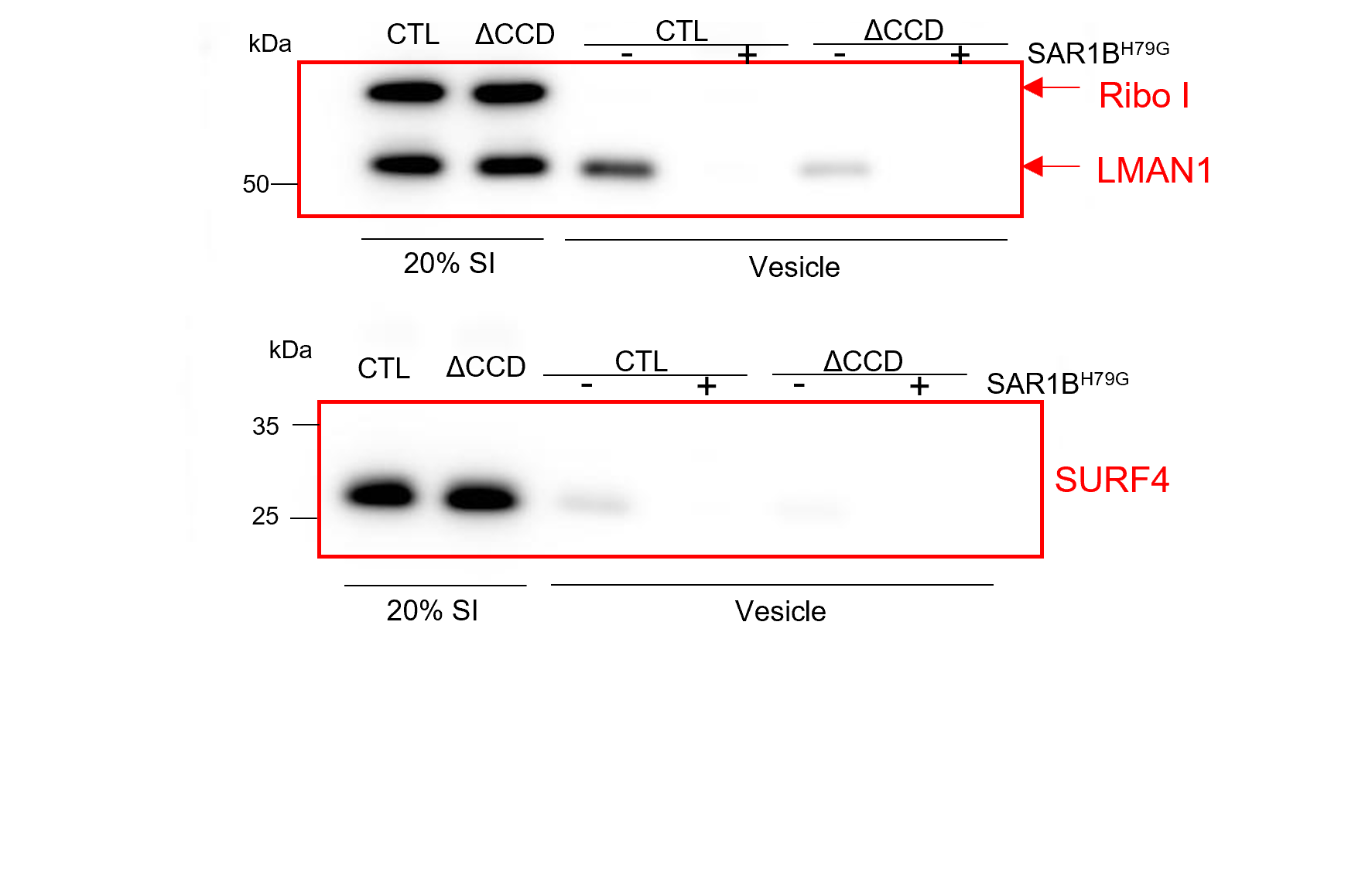

Supplement: Supplementary file 8 — Source data Fig. 5 [file 44318_2026_754_MOESM8_ESM.zip › Figure 5/5I/5I_western_LMAN1_label.tif]

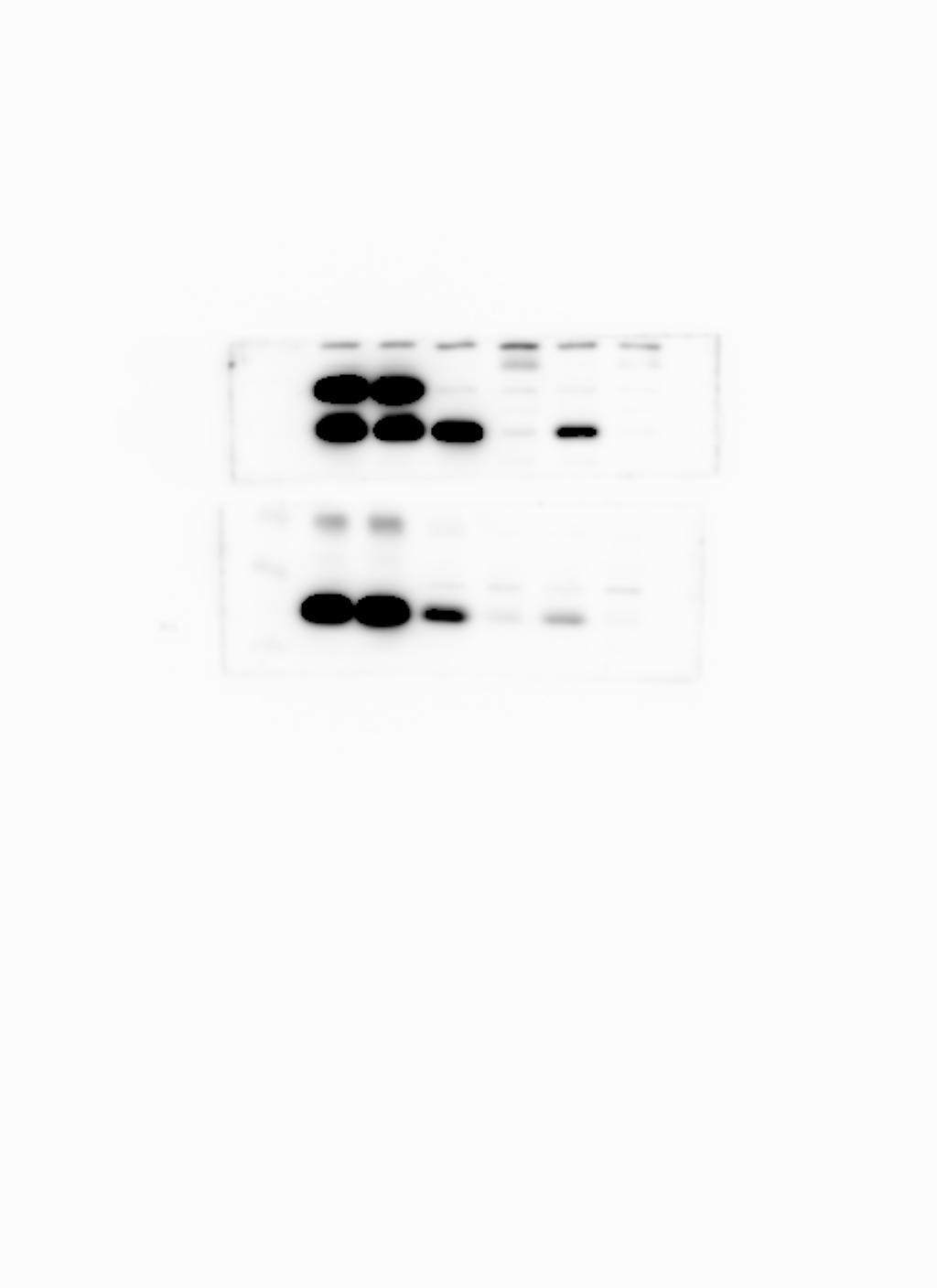

Supplement: Supplementary file 8 — Source data Fig. 5 [file 44318_2026_754_MOESM8_ESM.zip › Figure 5/5I/5I_western_SURF4.jpg]

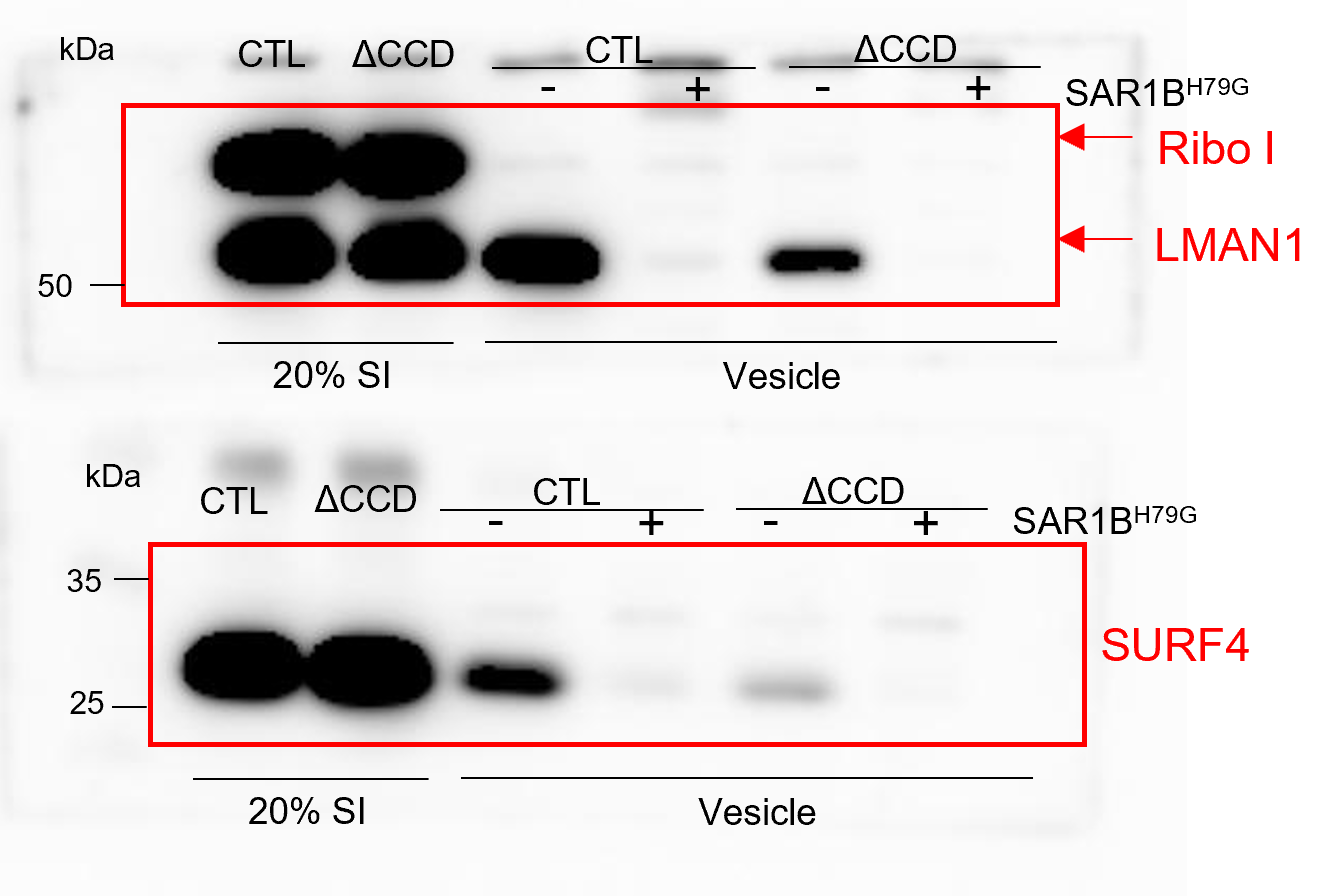

Supplement: Supplementary file 8 — Source data Fig. 5 [file 44318_2026_754_MOESM8_ESM.zip › Figure 5/5I/5I_western_SURF4_label.tif]

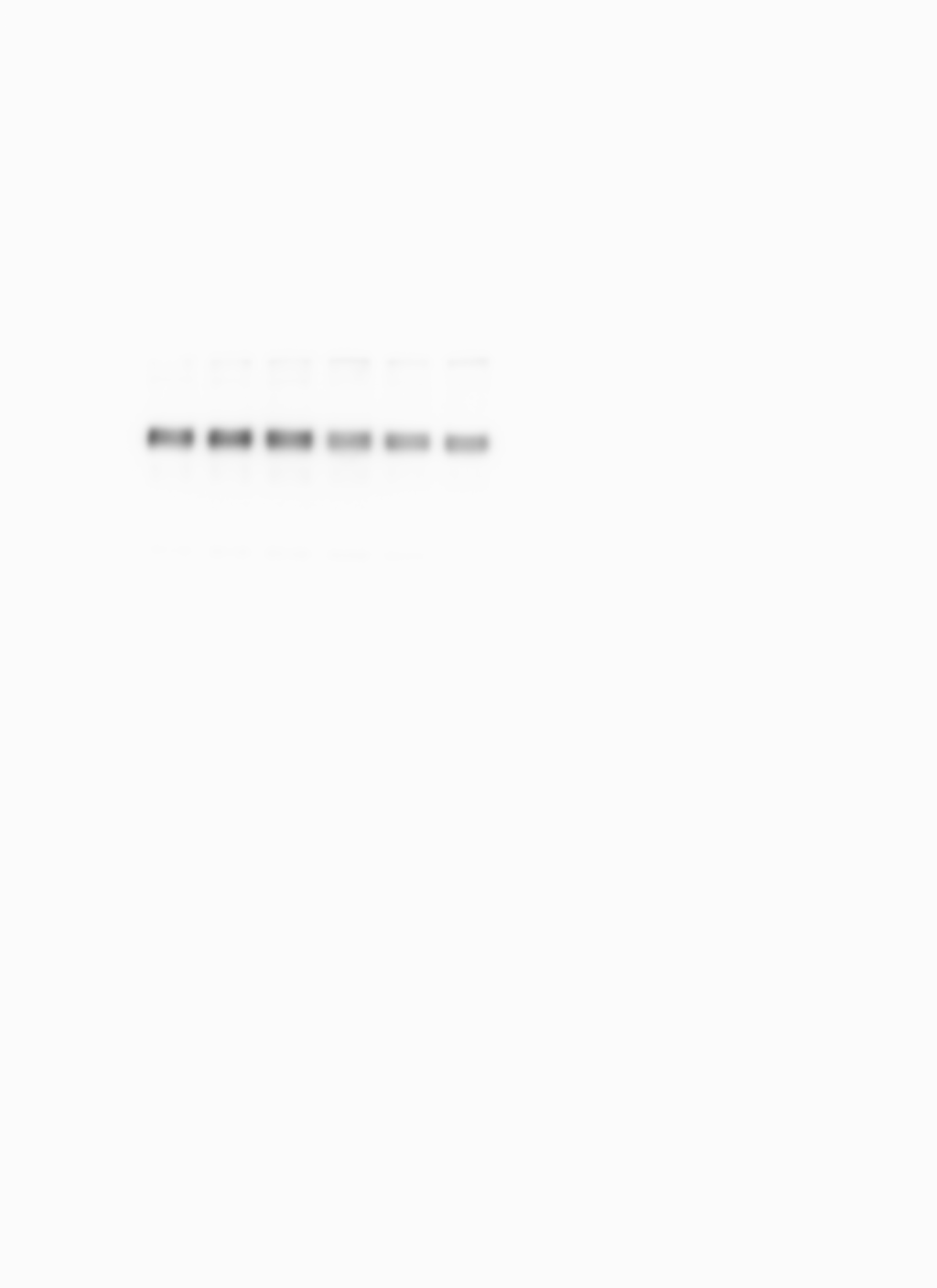

Supplement: Supplementary file 9 — Source data Fig. 6 [file 44318_2026_754_MOESM9_ESM.zip › Figure 6/6I-J/6I_western_HNF4A.tif]

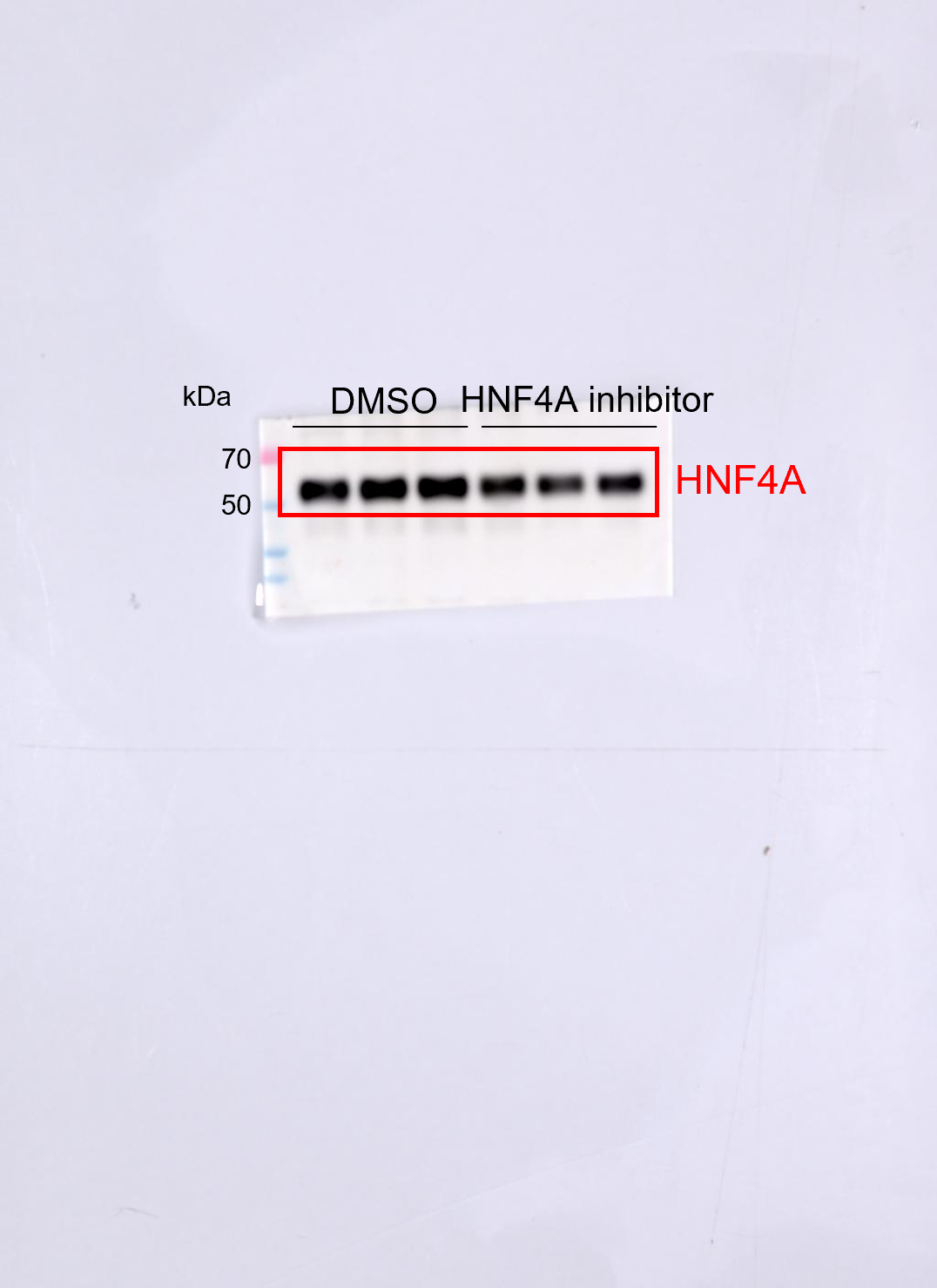

Supplement: Supplementary file 9 — Source data Fig. 6 [file 44318_2026_754_MOESM9_ESM.zip › Figure 6/6I-J/6I_western_HNF4A_label.tif]

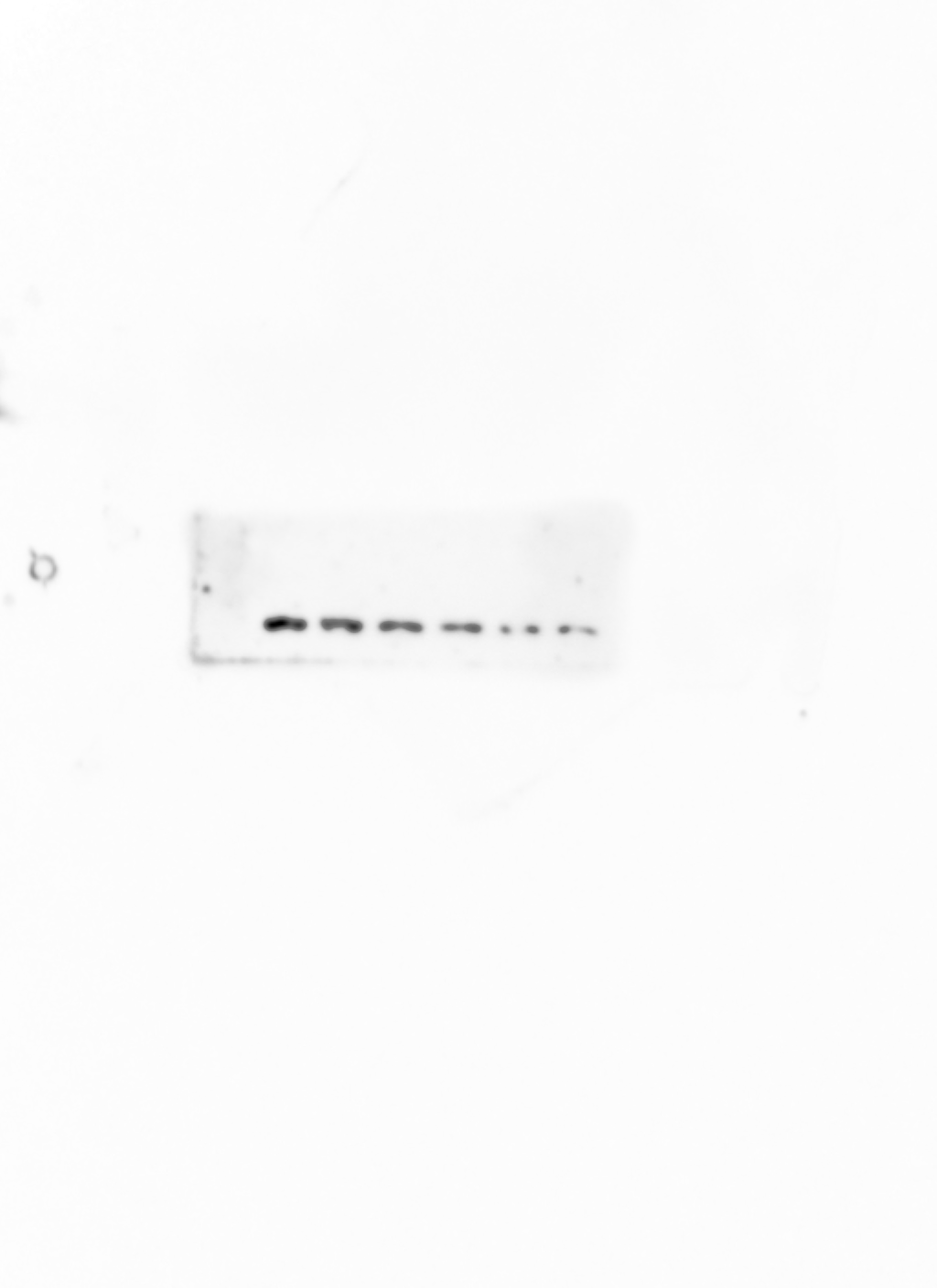

Supplement: Supplementary file 9 — Source data Fig. 6 [file 44318_2026_754_MOESM9_ESM.zip › Figure 6/6I-J/6I_western_MTP.tif]

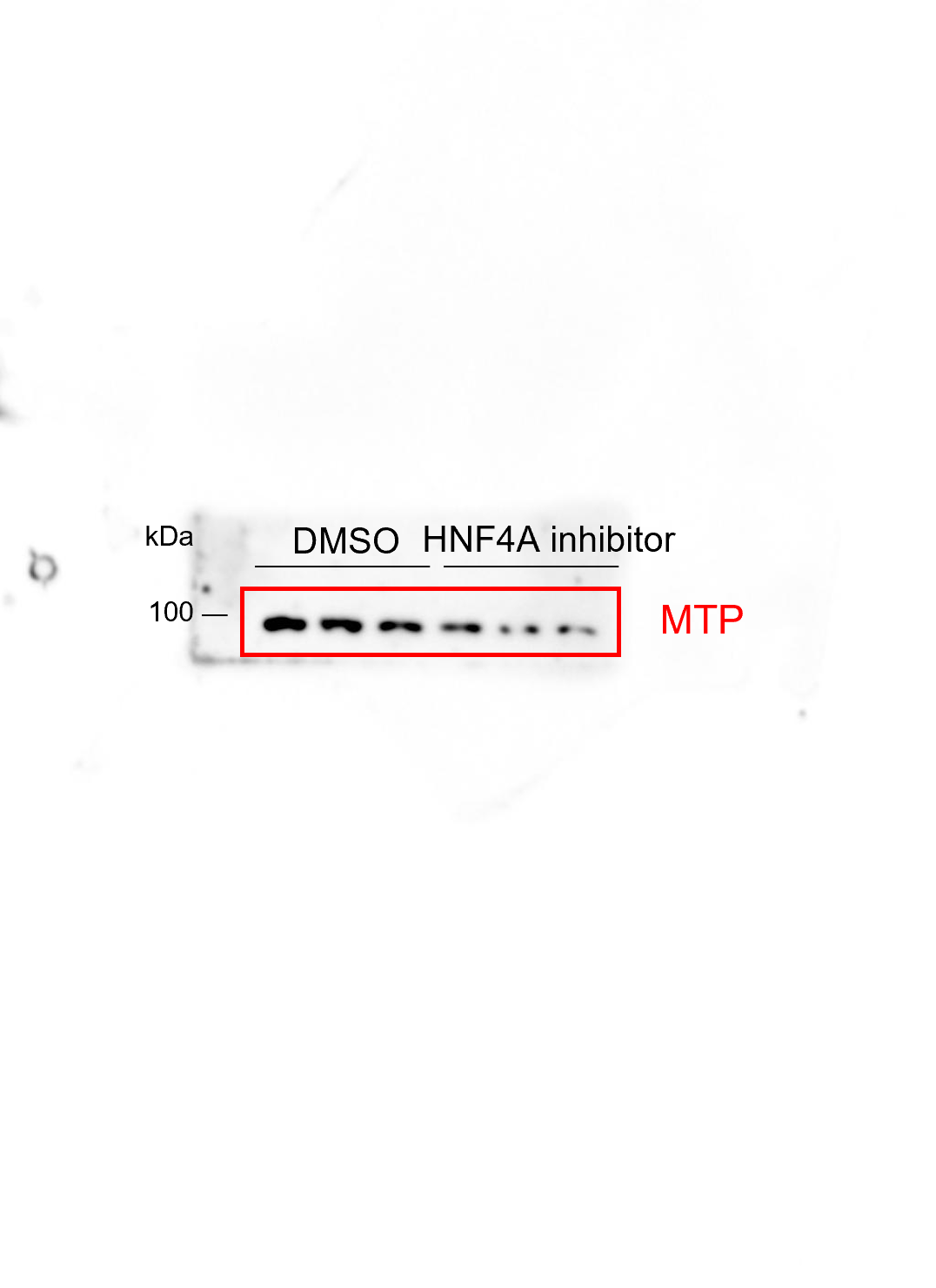

Supplement: Supplementary file 9 — Source data Fig. 6 [file 44318_2026_754_MOESM9_ESM.zip › Figure 6/6I-J/6I_western_MTP_label.tif]

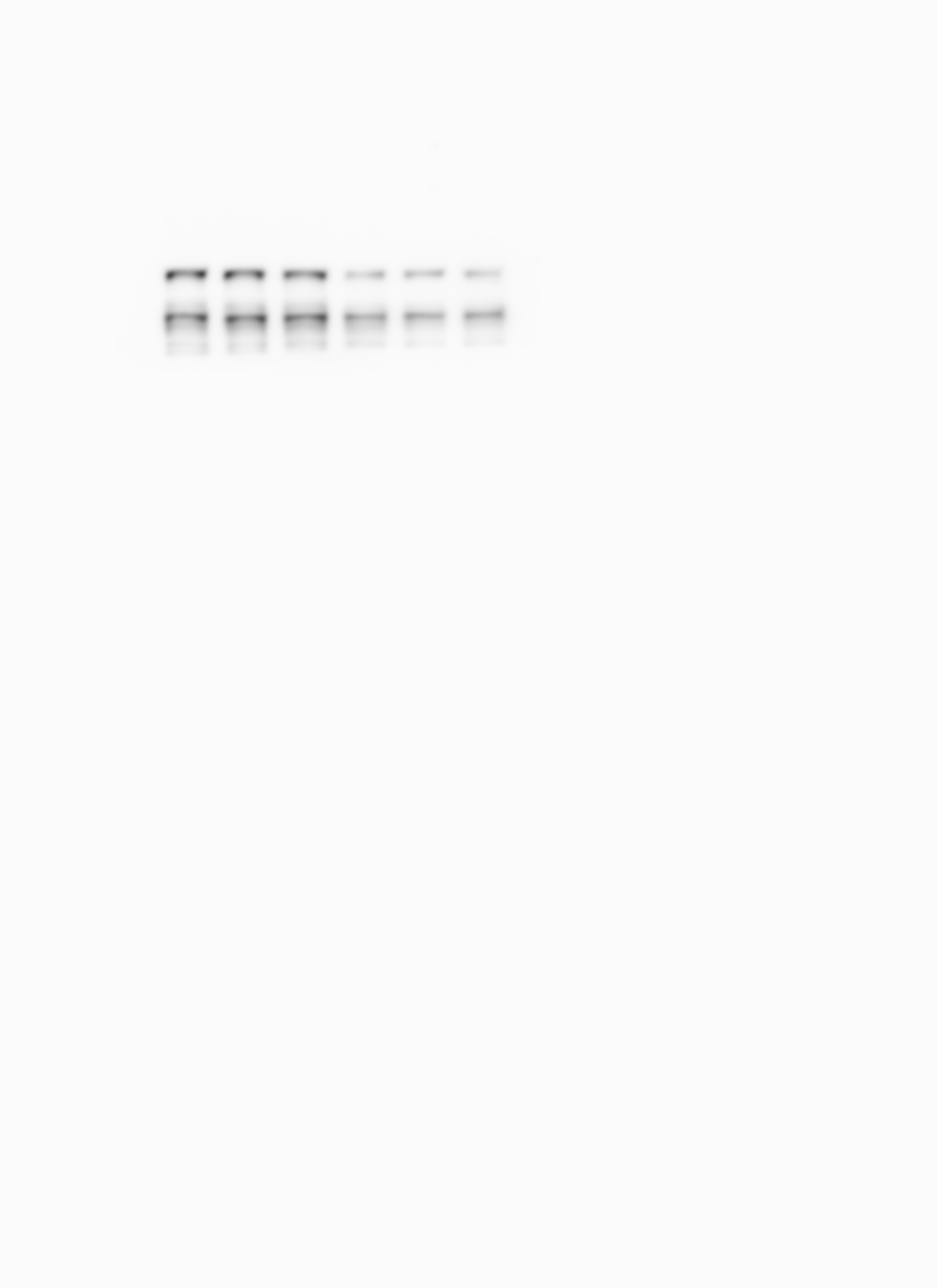

Supplement: Supplementary file 9 — Source data Fig. 6 [file 44318_2026_754_MOESM9_ESM.zip › Figure 6/6I-J/6I_western_SEC16B.tif]

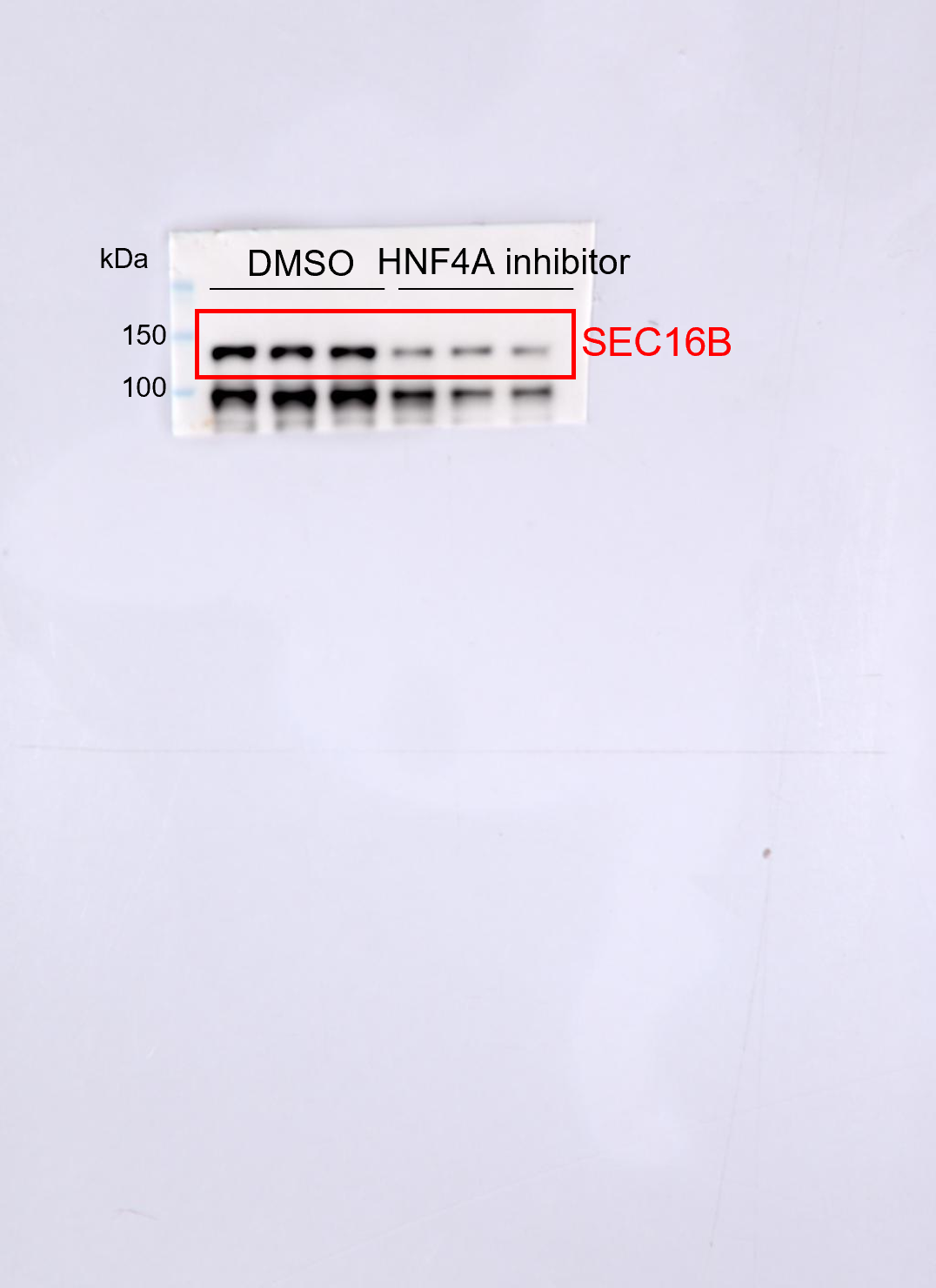

Supplement: Supplementary file 9 — Source data Fig. 6 [file 44318_2026_754_MOESM9_ESM.zip › Figure 6/6I-J/6I_western_SEC16B_label.tif]

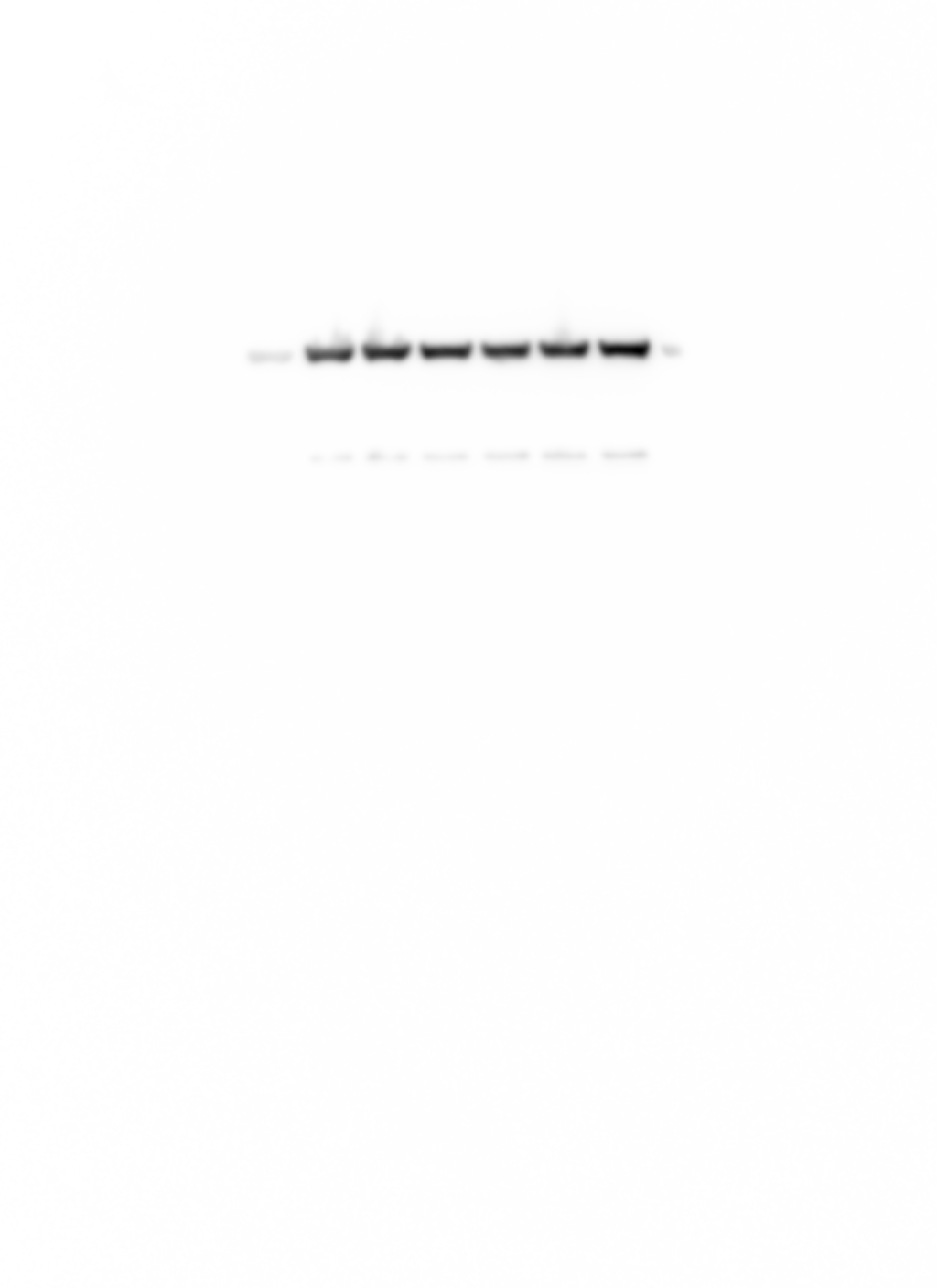

Supplement: Supplementary file 9 — Source data Fig. 6 [file 44318_2026_754_MOESM9_ESM.zip › Figure 6/6I-J/6I_western_Tub.tif]

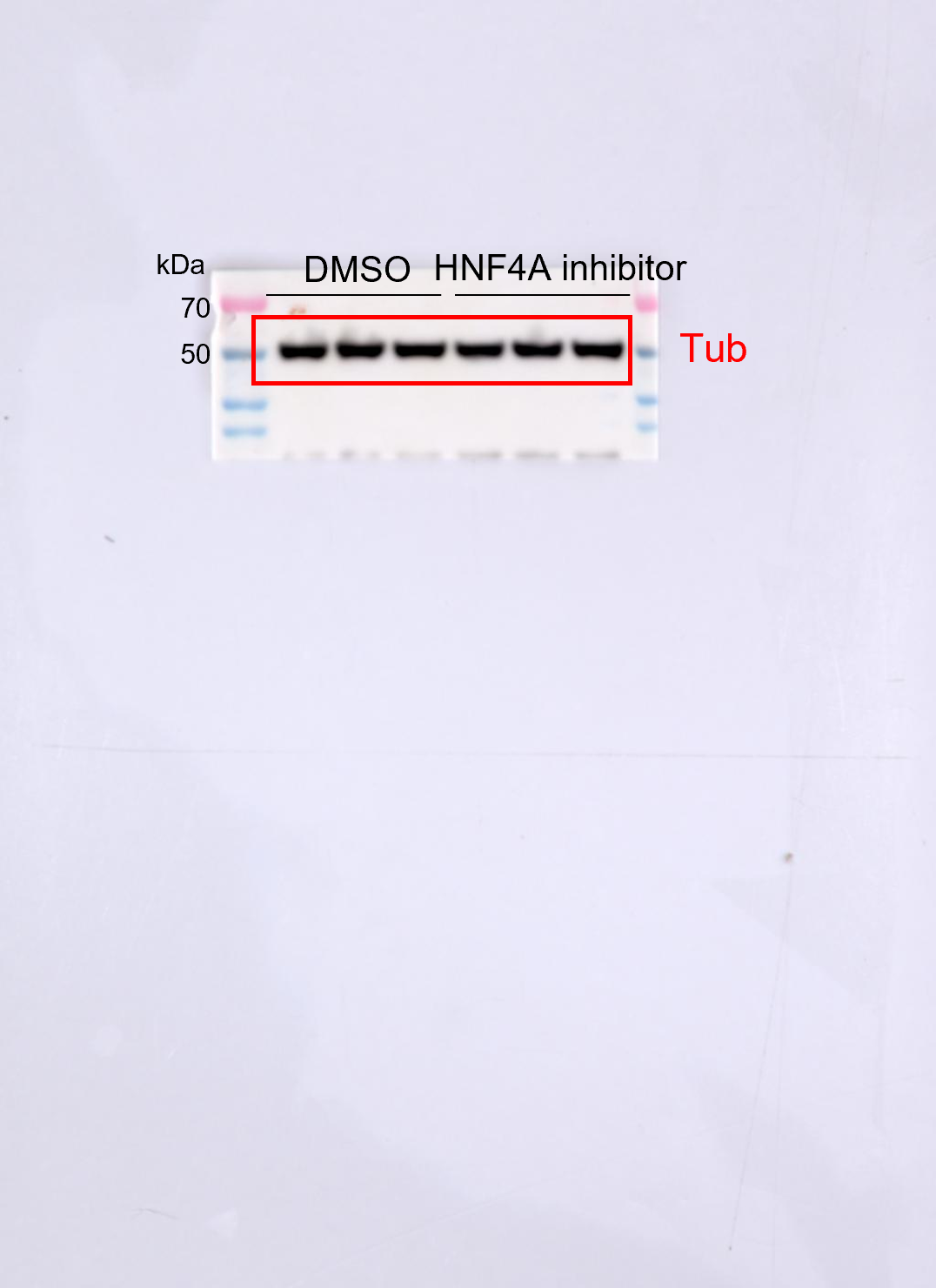

Supplement: Supplementary file 9 — Source data Fig. 6 [file 44318_2026_754_MOESM9_ESM.zip › Figure 6/6I-J/6I_western_Tub_label.tif]

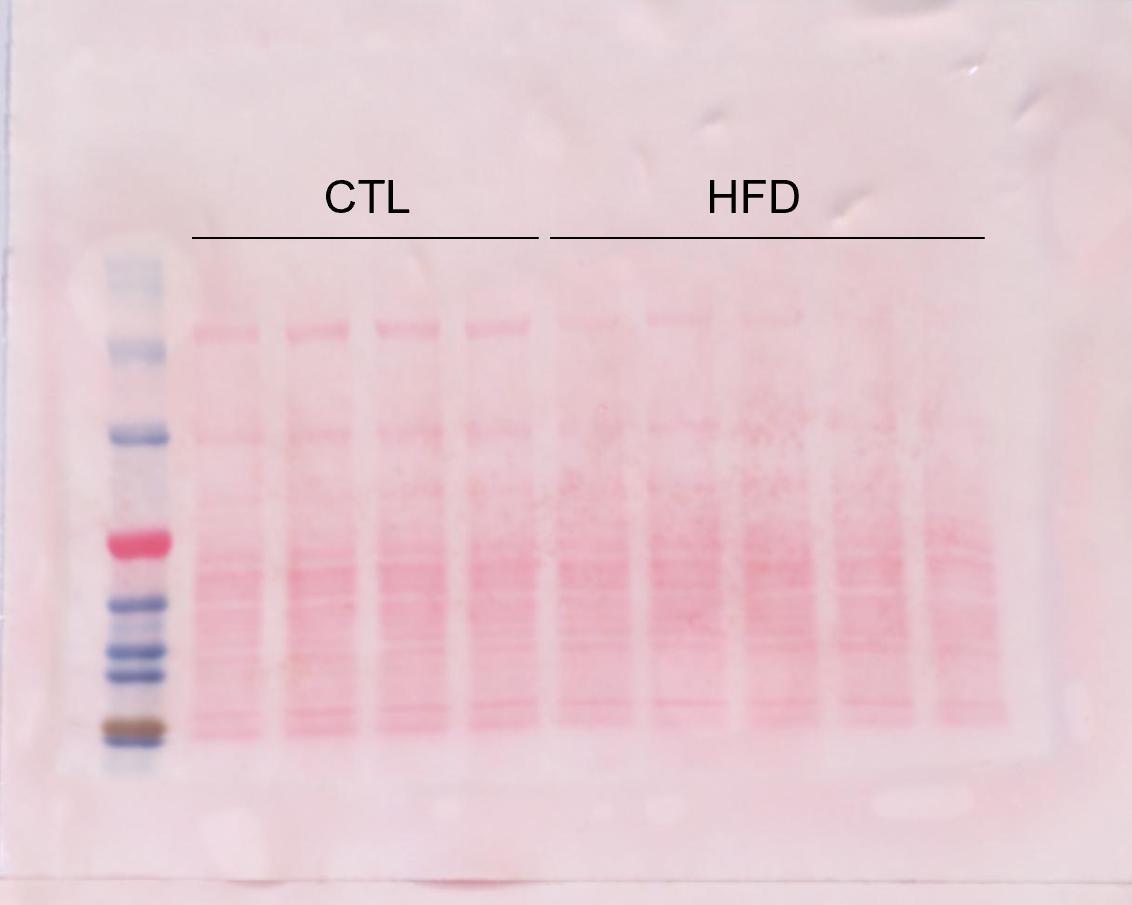

Supplement: Supplementary file 9 — Source data Fig. 6 [file 44318_2026_754_MOESM9_ESM.zip › Figure 6/6K-L/6K_western_HFD_Ponceau S.tif]

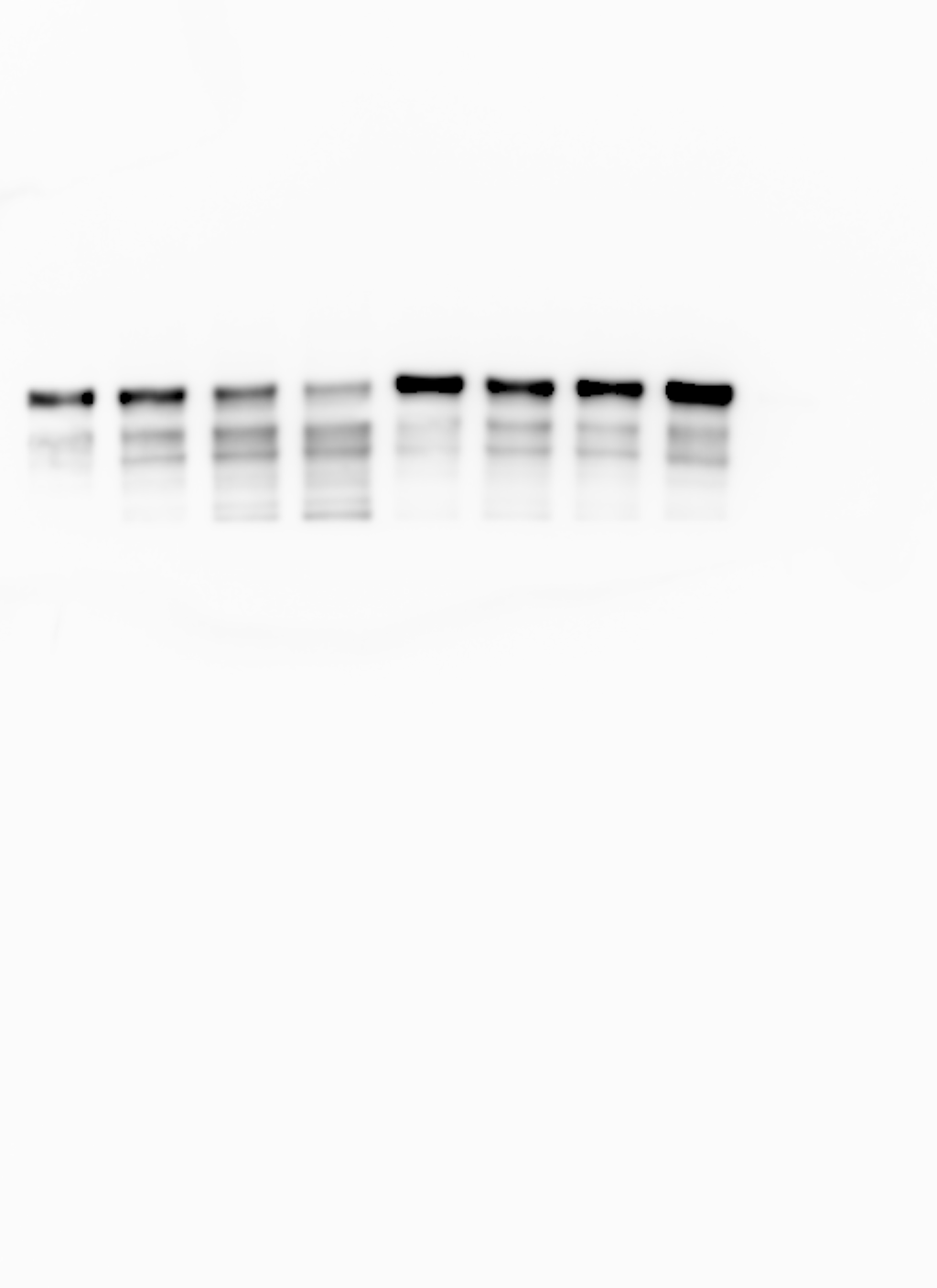

Supplement: Supplementary file 9 — Source data Fig. 6 [file 44318_2026_754_MOESM9_ESM.zip › Figure 6/6K-L/6K_western_HFD_SEC16B.tif]

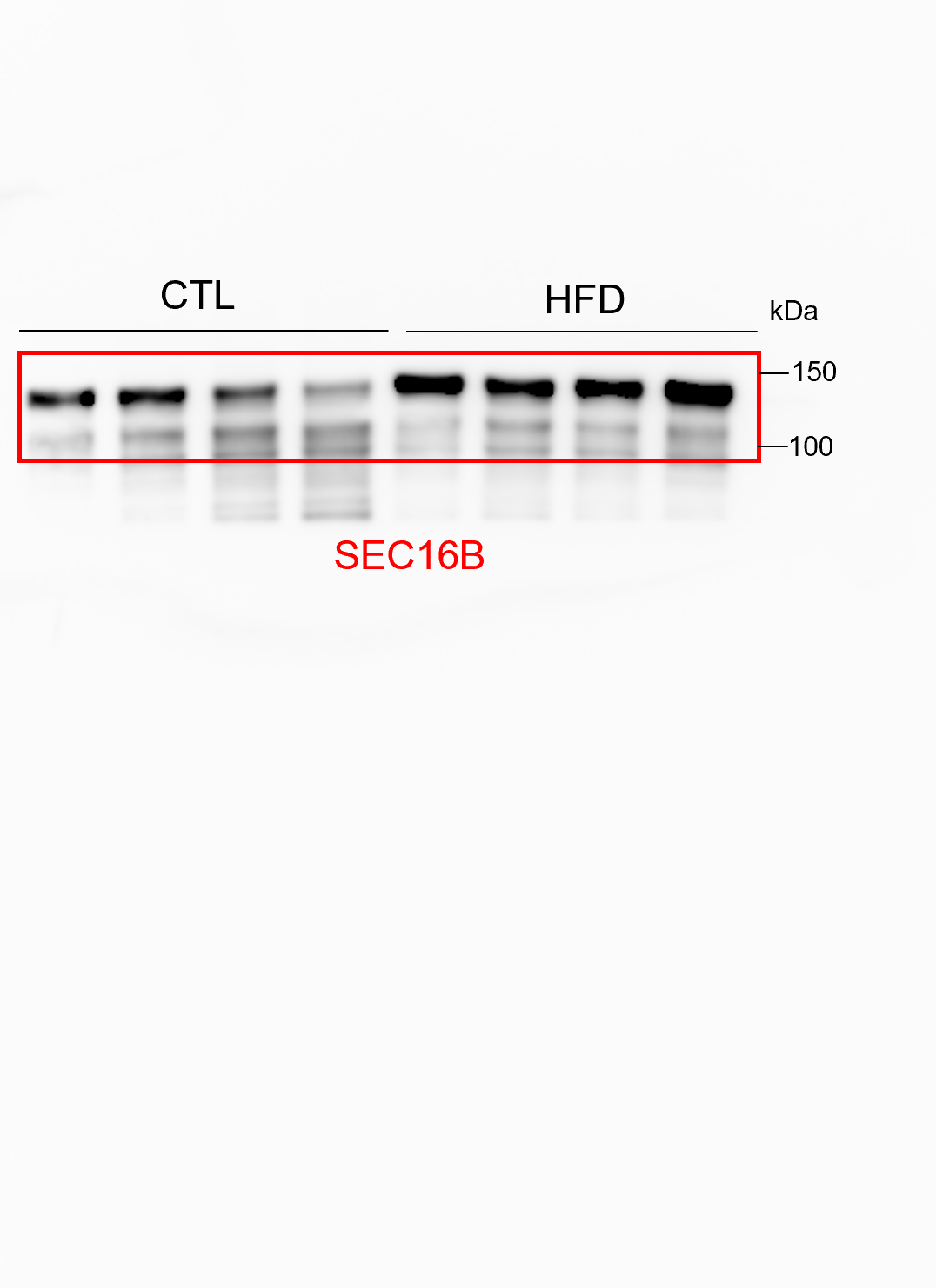

Supplement: Supplementary file 9 — Source data Fig. 6 [file 44318_2026_754_MOESM9_ESM.zip › Figure 6/6K-L/6K_western_HFD_SEC16B_label.tif]

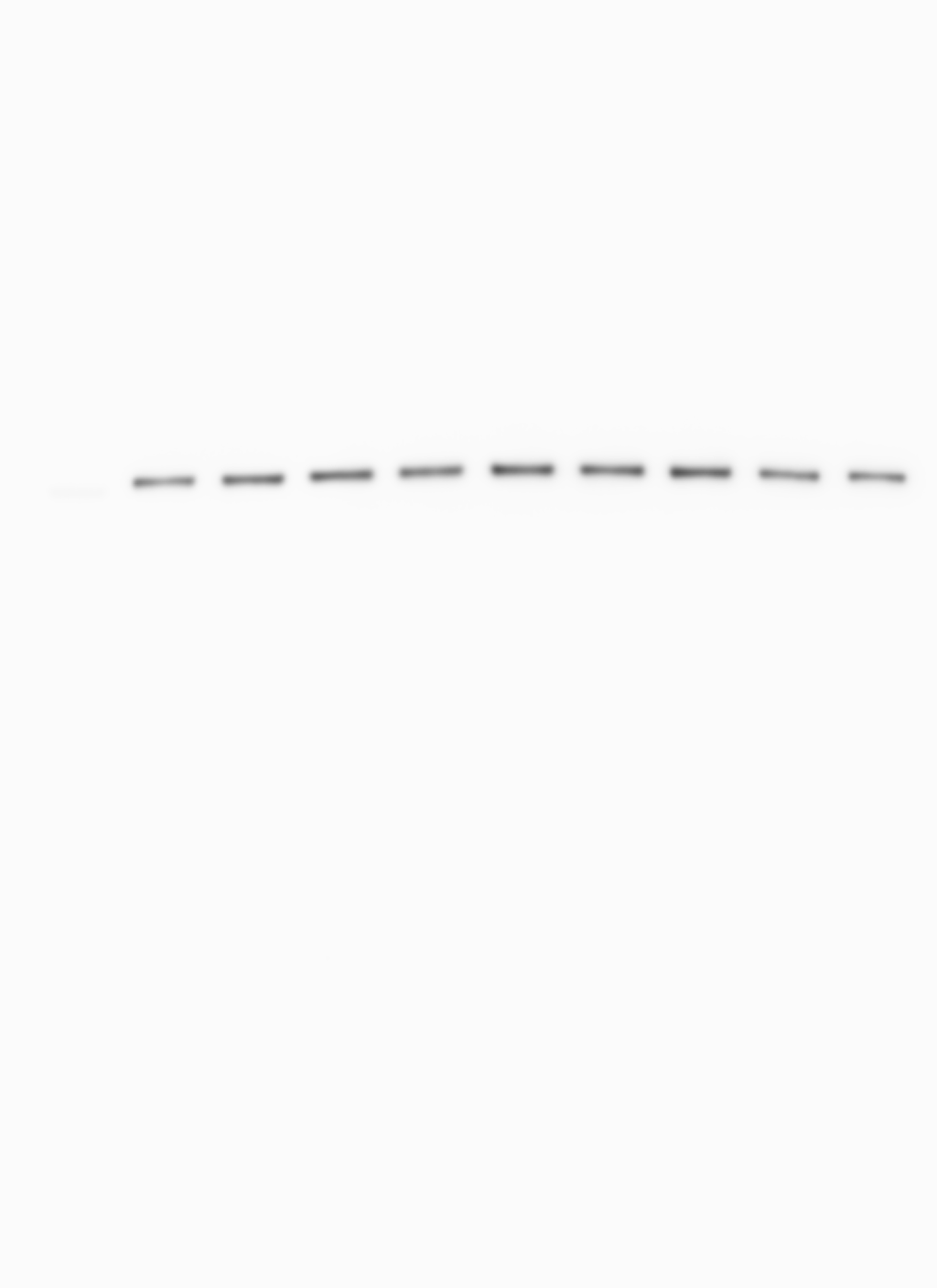

Supplement: Supplementary file 9 — Source data Fig. 6 [file 44318_2026_754_MOESM9_ESM.zip › Figure 6/6K-L/6K_western_HFD_Tub.tif]

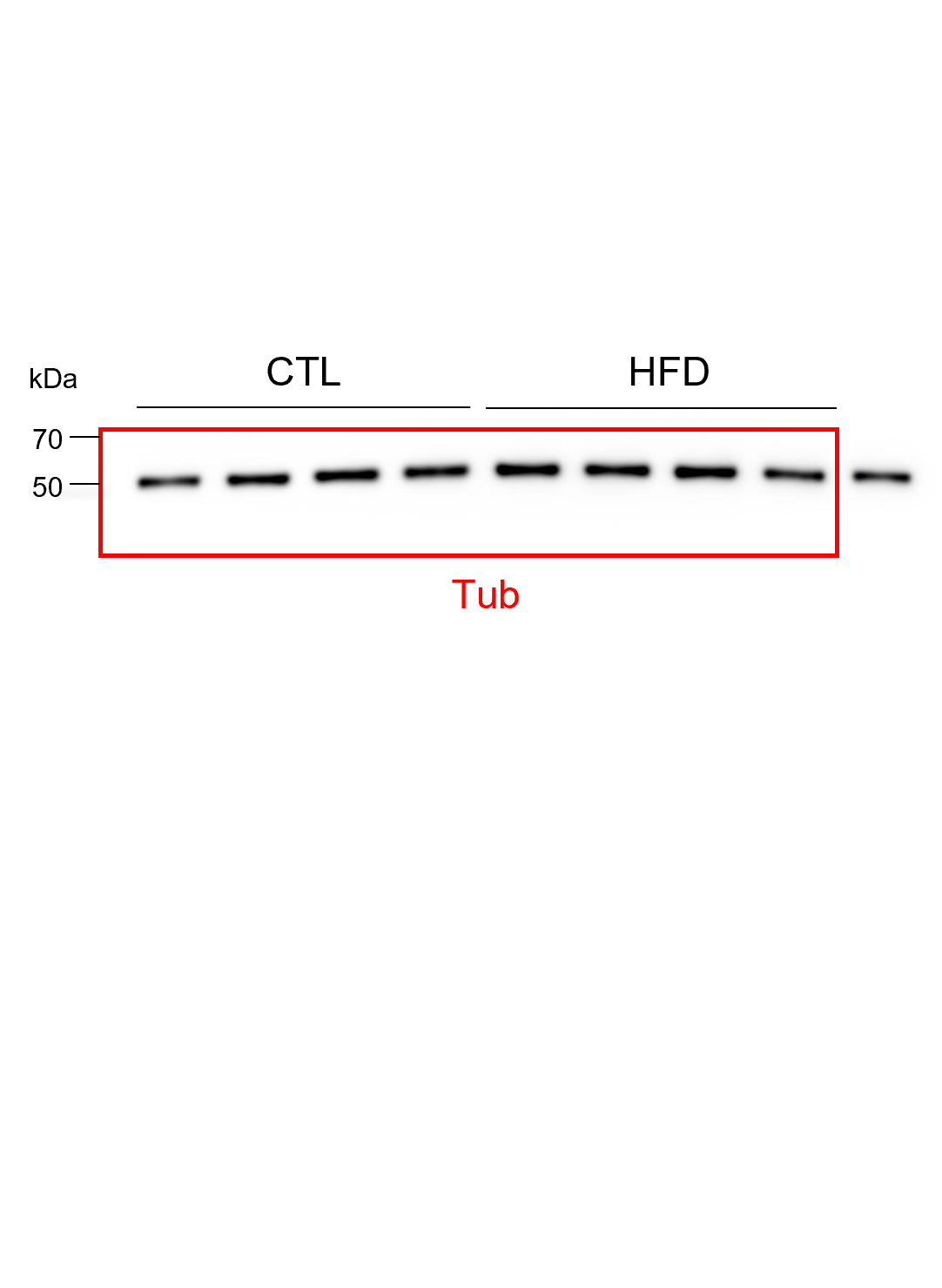

Supplement: Supplementary file 9 — Source data Fig. 6 [file 44318_2026_754_MOESM9_ESM.zip › Figure 6/6K-L/6K_western_HFD_Tub_label.tif]

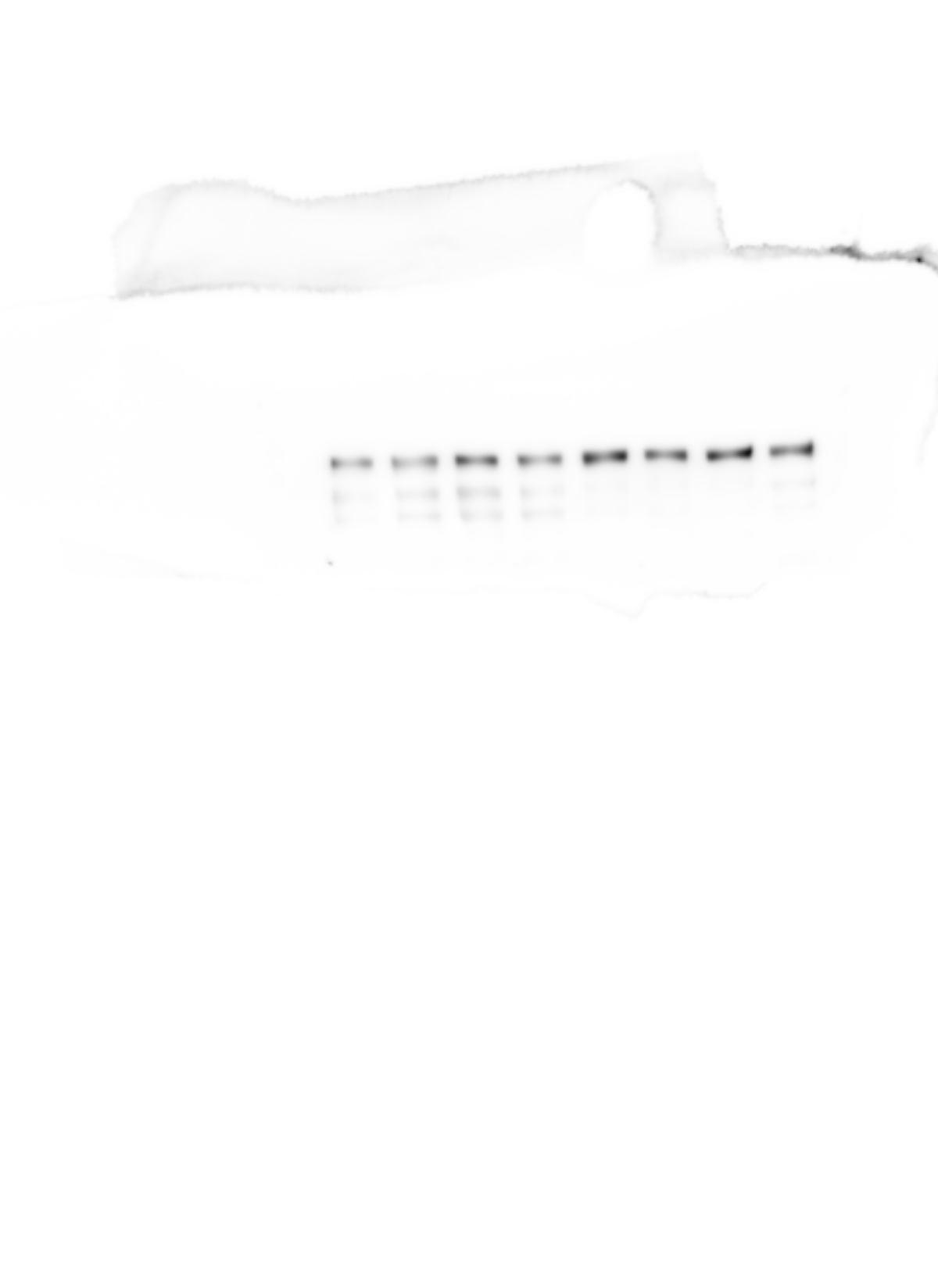

Supplement: Supplementary file 9 — Source data Fig. 6 [file 44318_2026_754_MOESM9_ESM.zip › Figure 6/6K-L/6K_western_Hyperlipidmia_SEC16B.tif]

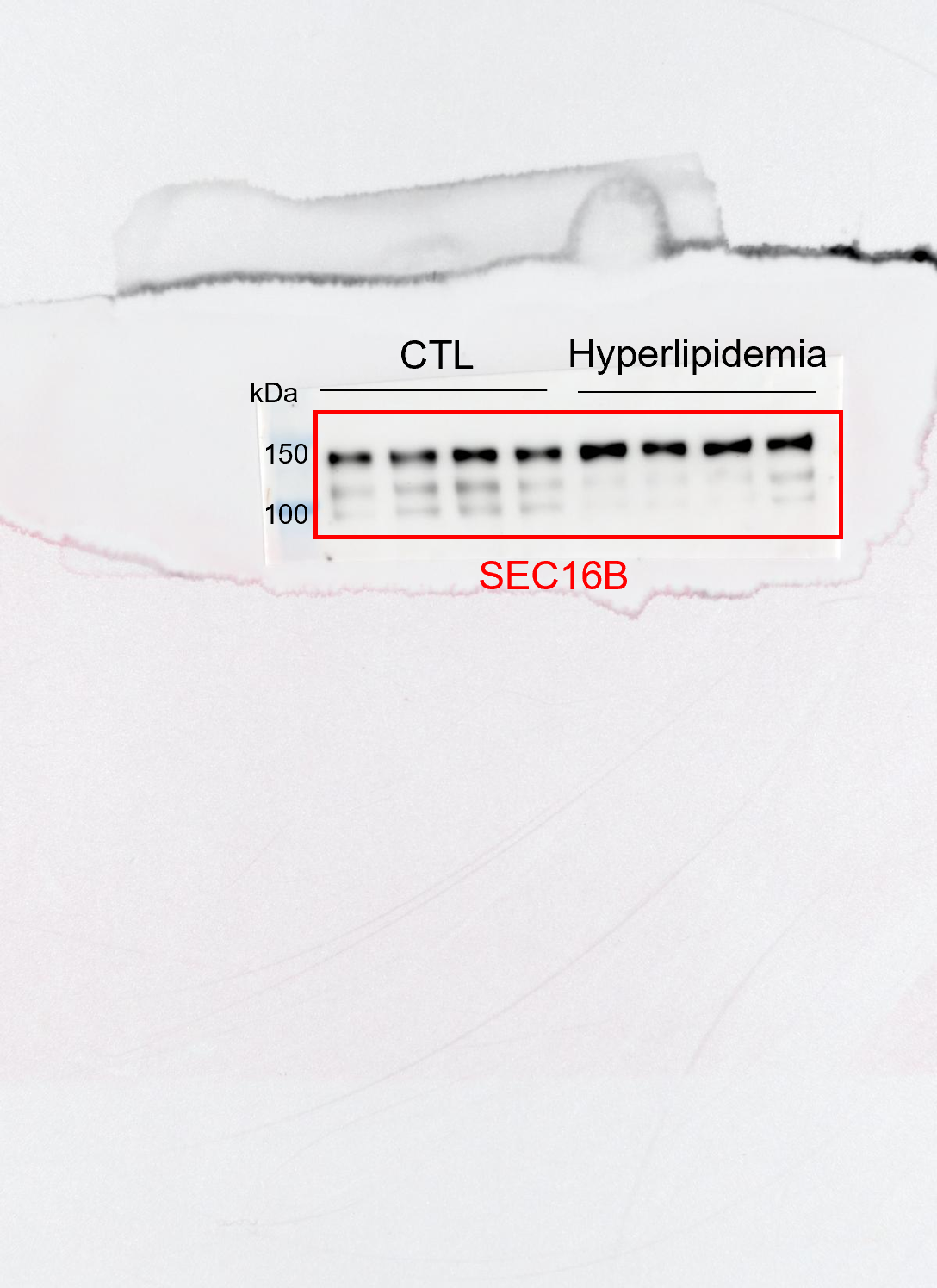

Supplement: Supplementary file 9 — Source data Fig. 6 [file 44318_2026_754_MOESM9_ESM.zip › Figure 6/6K-L/6K_western_Hyperlipidmia_SEC16B_label.tif]

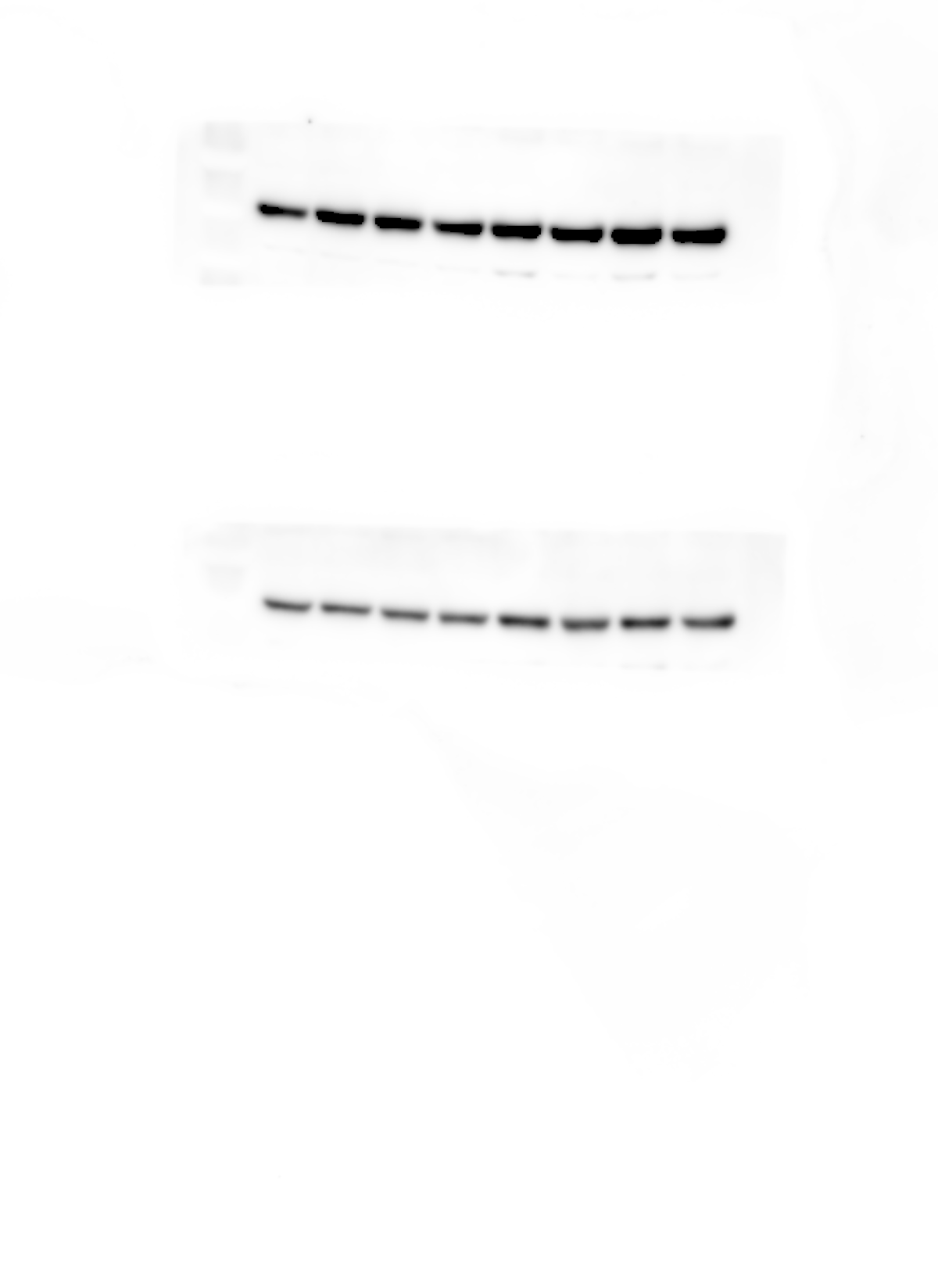

Supplement: Supplementary file 9 — Source data Fig. 6 [file 44318_2026_754_MOESM9_ESM.zip › Figure 6/6K-L/6K_western_Hyperlipidmia_Tub.tif]

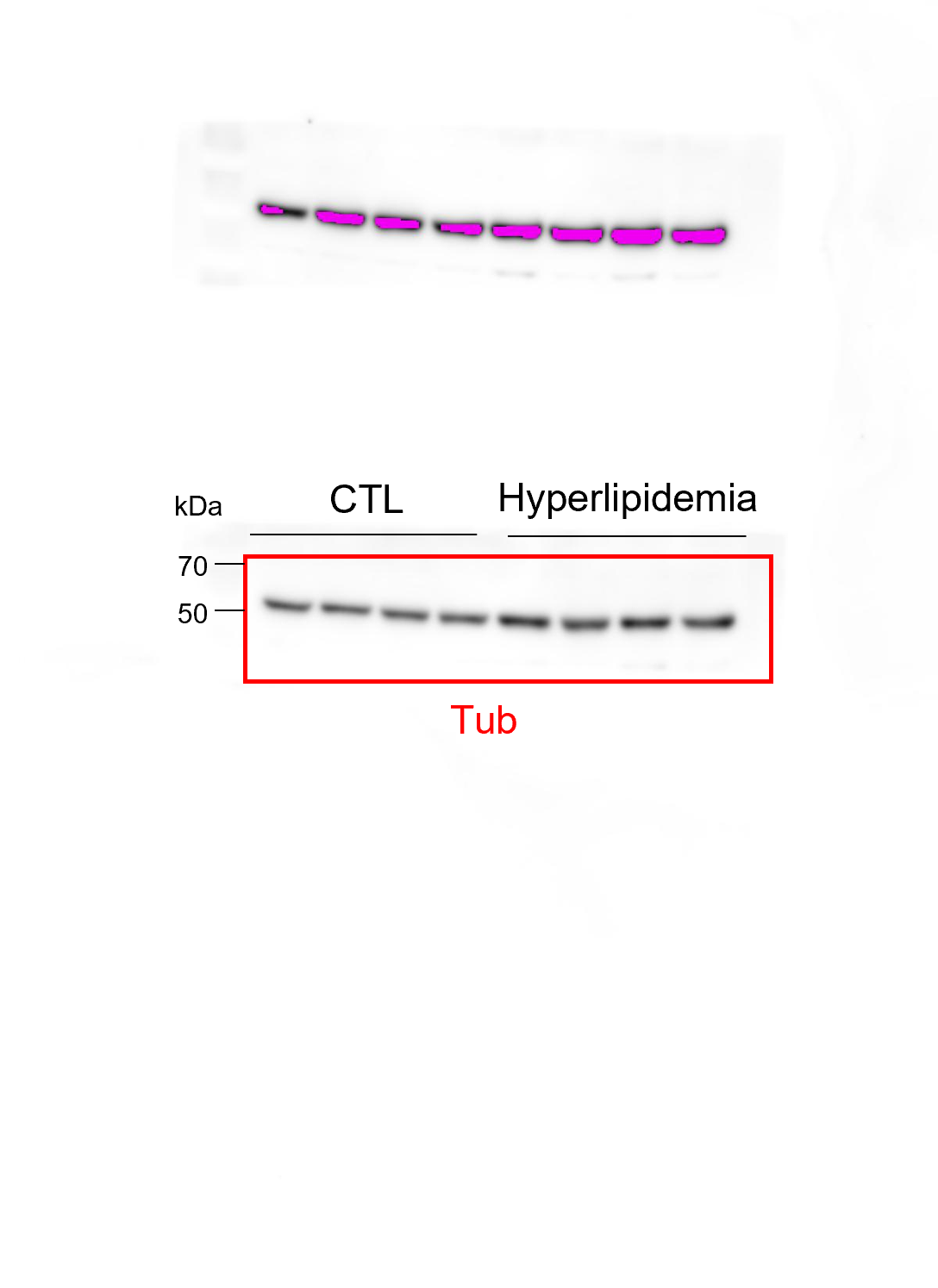

Supplement: Supplementary file 9 — Source data Fig. 6 [file 44318_2026_754_MOESM9_ESM.zip › Figure 6/6K-L/6K_western_Hyperlipidmia_Tub_label.tif]

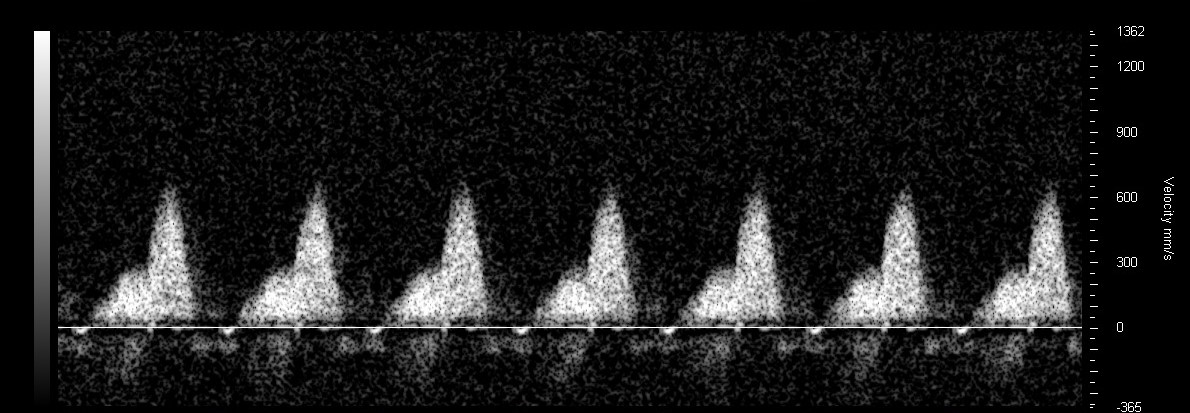

Supplement: Supplementary file 10 — Source data Fig. 7 [file 44318_2026_754_MOESM10_ESM.zip › Figure 7/7I-J/7I_image_KO_Hyperemia.tiff]

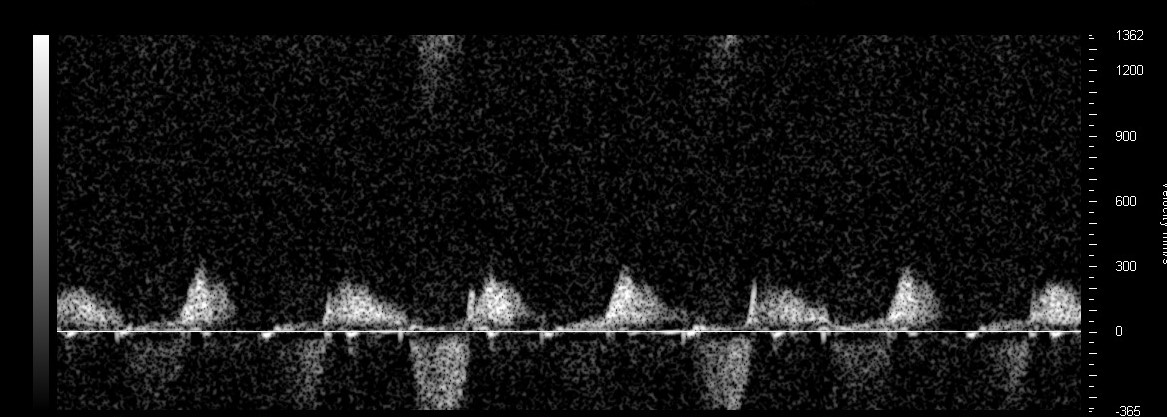

Supplement: Supplementary file 10 — Source data Fig. 7 [file 44318_2026_754_MOESM10_ESM.zip › Figure 7/7I-J/7I_image_KO_Rest.tiff]

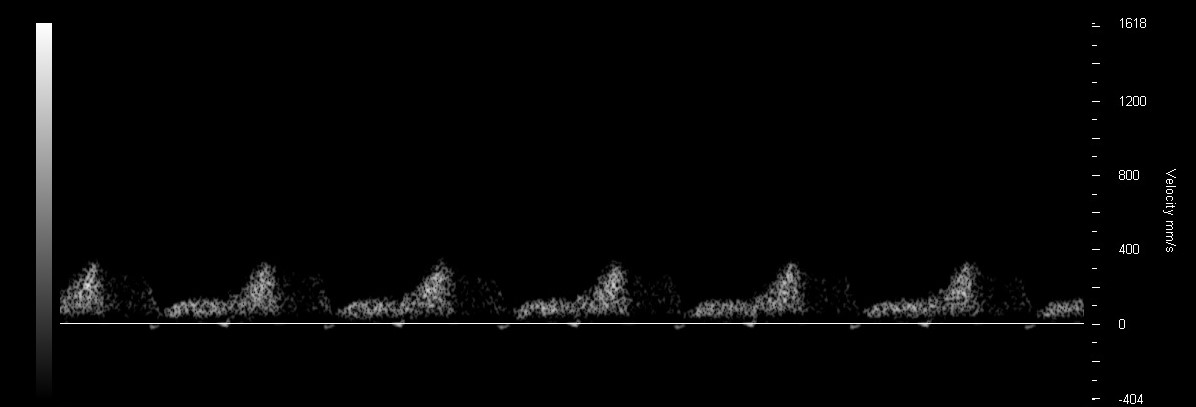

Supplement: Supplementary file 10 — Source data Fig. 7 [file 44318_2026_754_MOESM10_ESM.zip › Figure 7/7I-J/7I_image_WT_Hyperemia.tiff]

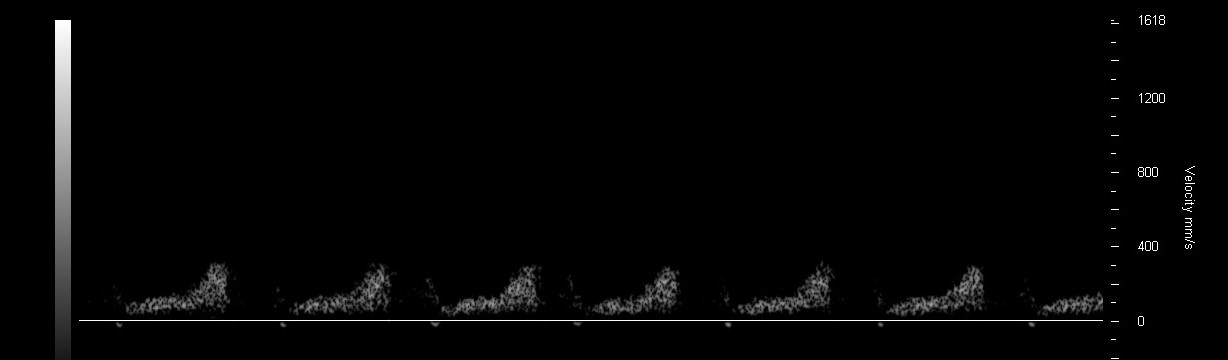

Supplement: Supplementary file 10 — Source data Fig. 7 [file 44318_2026_754_MOESM10_ESM.zip › Figure 7/7I-J/7I_image_WT_Rest.tiff]

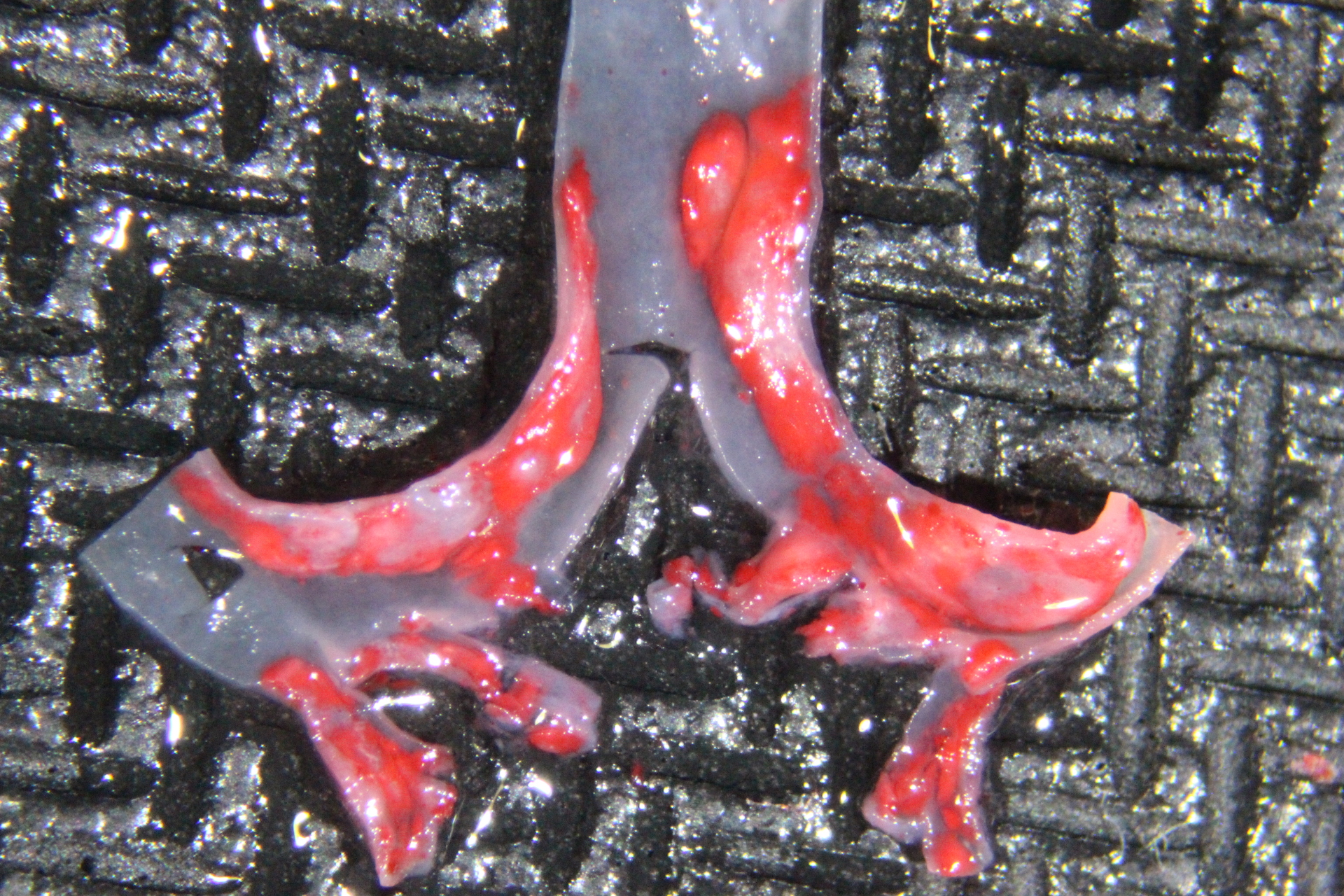

Supplement: Supplementary file 10 — Source data Fig. 7 [file 44318_2026_754_MOESM10_ESM.zip › Figure 7/7K-L/7K_image_CTL.1.tiff]

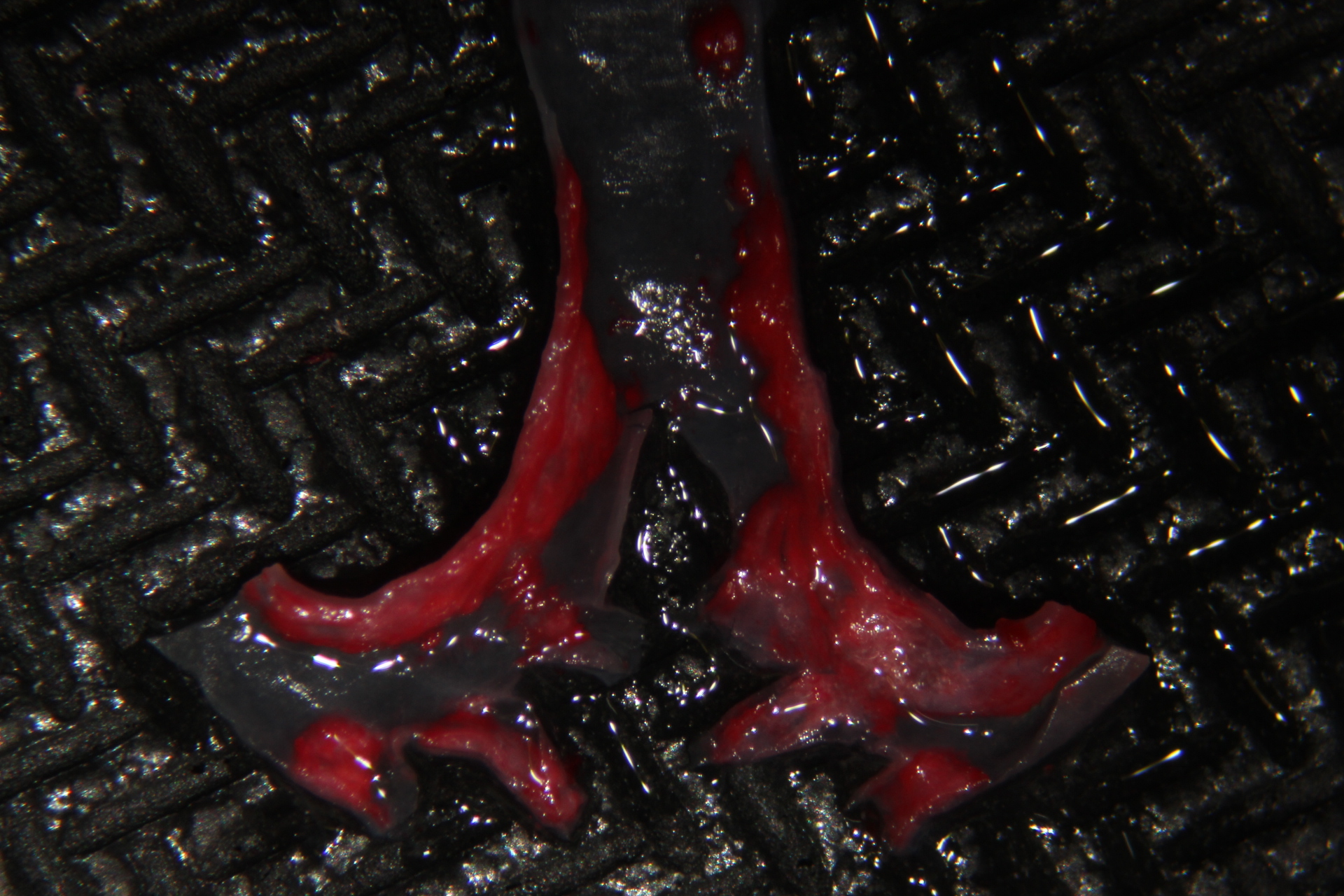

Supplement: Supplementary file 10 — Source data Fig. 7 [file 44318_2026_754_MOESM10_ESM.zip › Figure 7/7K-L/7K_image_CTL.2.tiff]

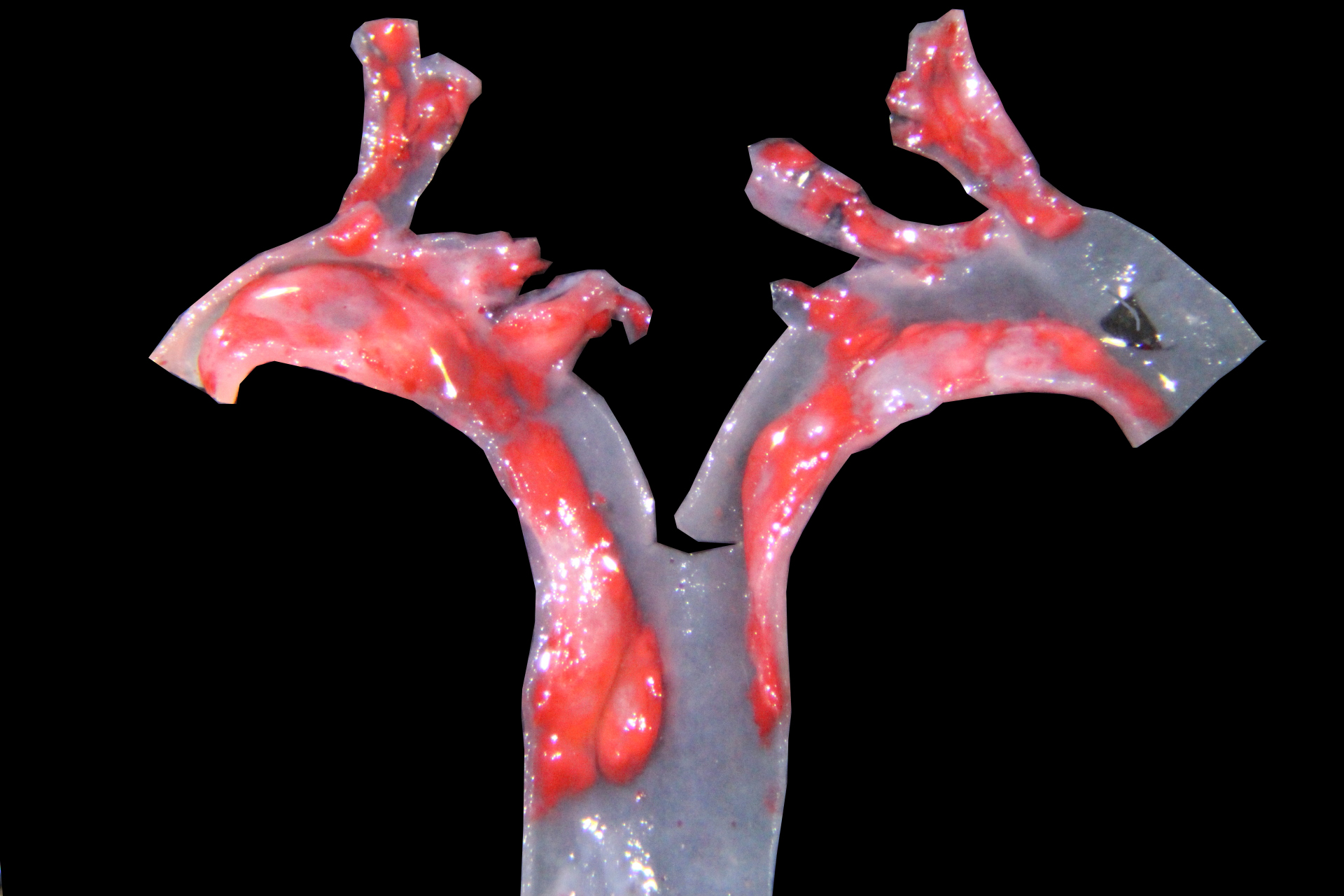

Supplement: Supplementary file 10 — Source data Fig. 7 [file 44318_2026_754_MOESM10_ESM.zip › Figure 7/7K-L/7K_image_CTL_processed.1.tiff]

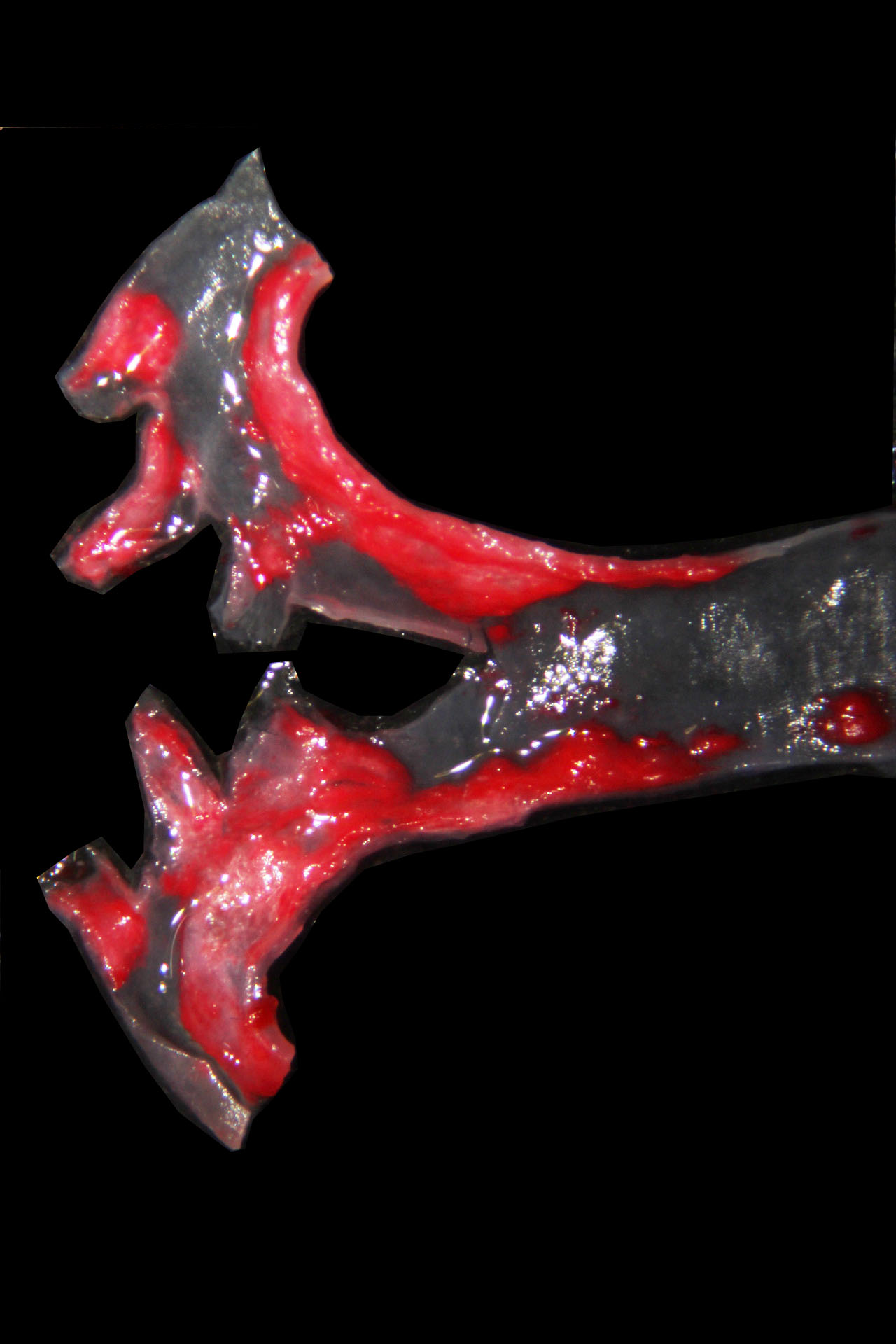

Supplement: Supplementary file 10 — Source data Fig. 7 [file 44318_2026_754_MOESM10_ESM.zip › Figure 7/7K-L/7K_image_CTL_processed.2.tiff]

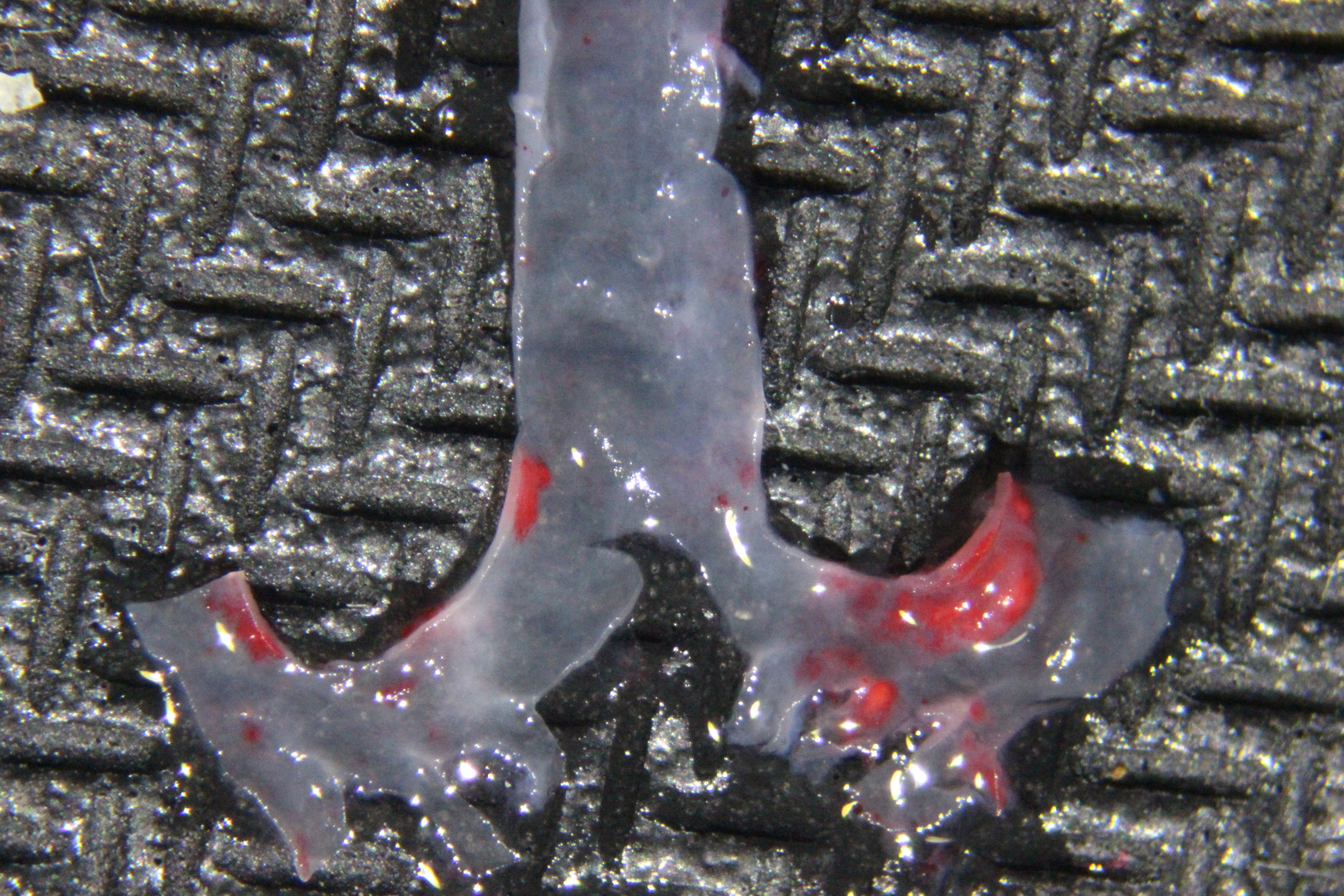

Supplement: Supplementary file 10 — Source data Fig. 7 [file 44318_2026_754_MOESM10_ESM.zip › Figure 7/7K-L/7K_image_KO.1.tiff]

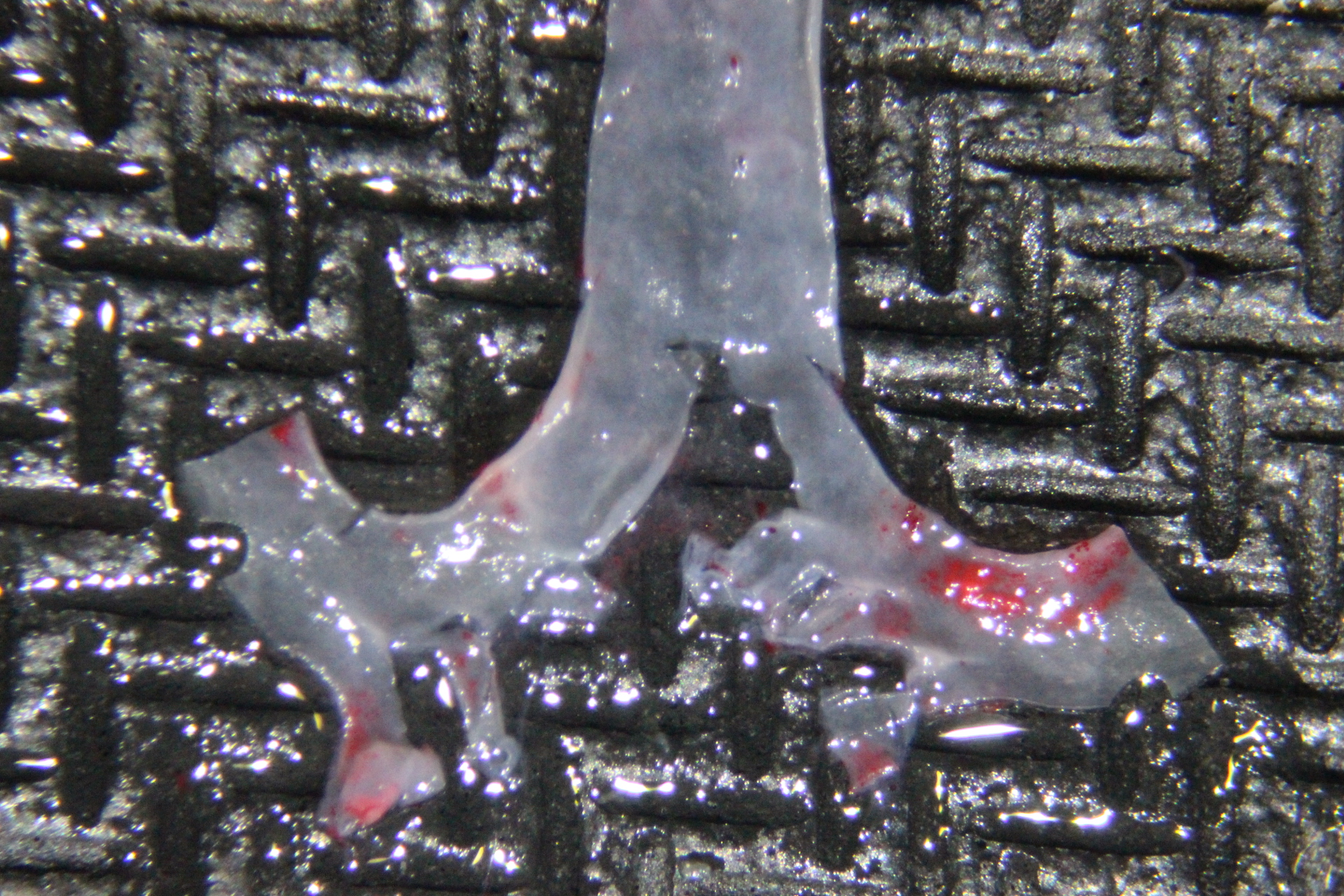

Supplement: Supplementary file 10 — Source data Fig. 7 [file 44318_2026_754_MOESM10_ESM.zip › Figure 7/7K-L/7K_image_KO.2.tiff]

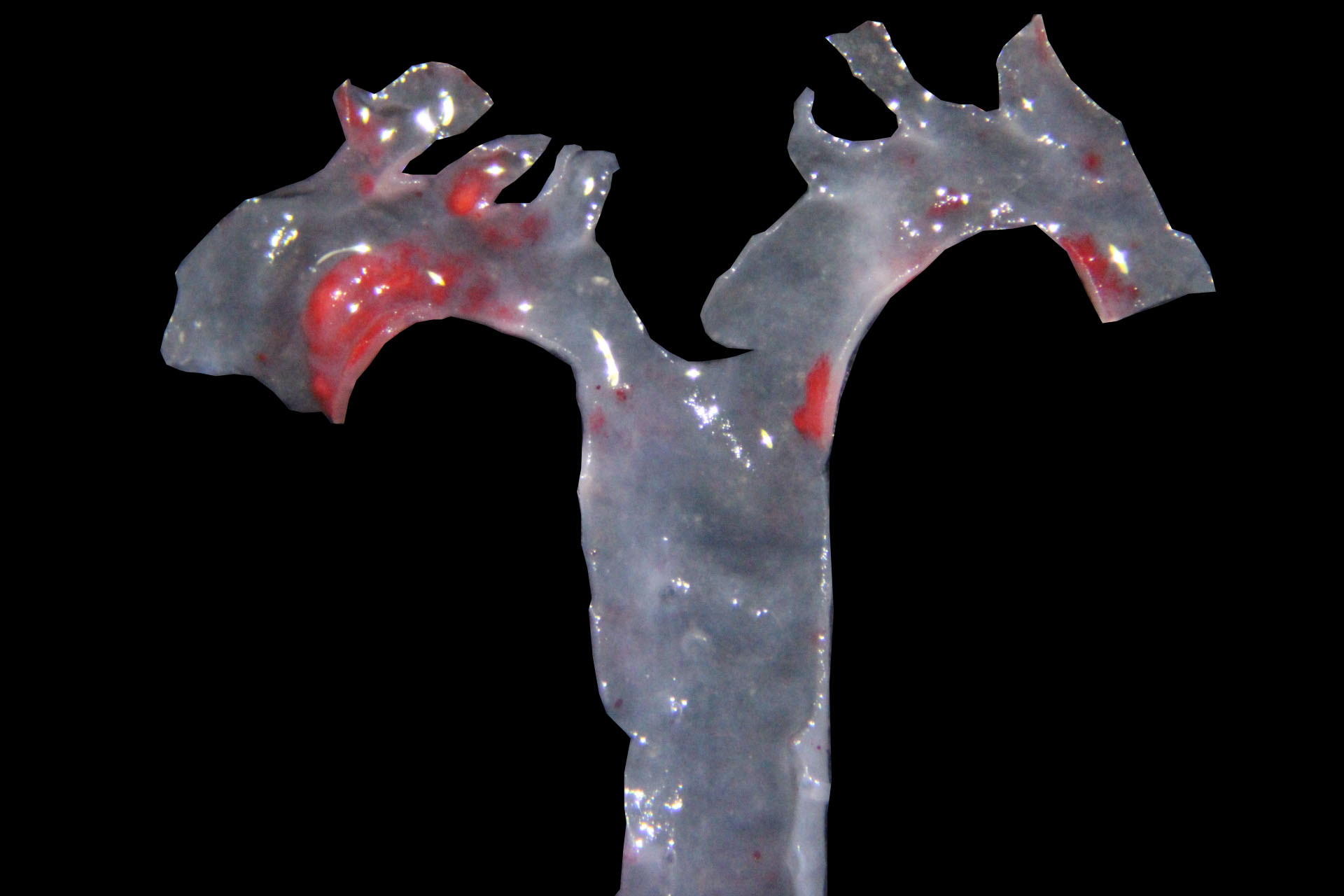

Supplement: Supplementary file 10 — Source data Fig. 7 [file 44318_2026_754_MOESM10_ESM.zip › Figure 7/7K-L/7K_image_KO_processed.1.tiff]

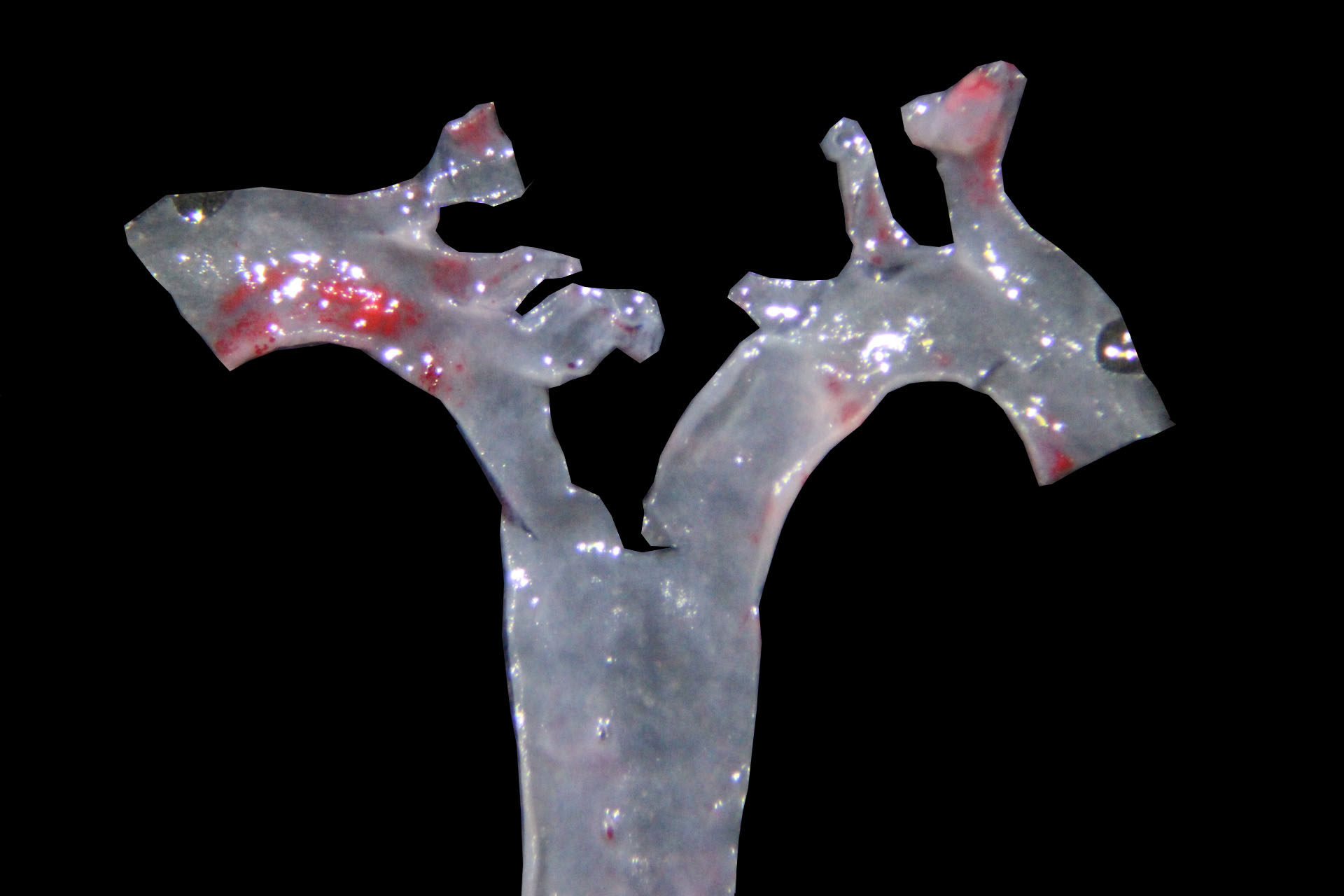

Supplement: Supplementary file 10 — Source data Fig. 7 [file 44318_2026_754_MOESM10_ESM.zip › Figure 7/7K-L/7K_image_KO_processed.2.tiff]

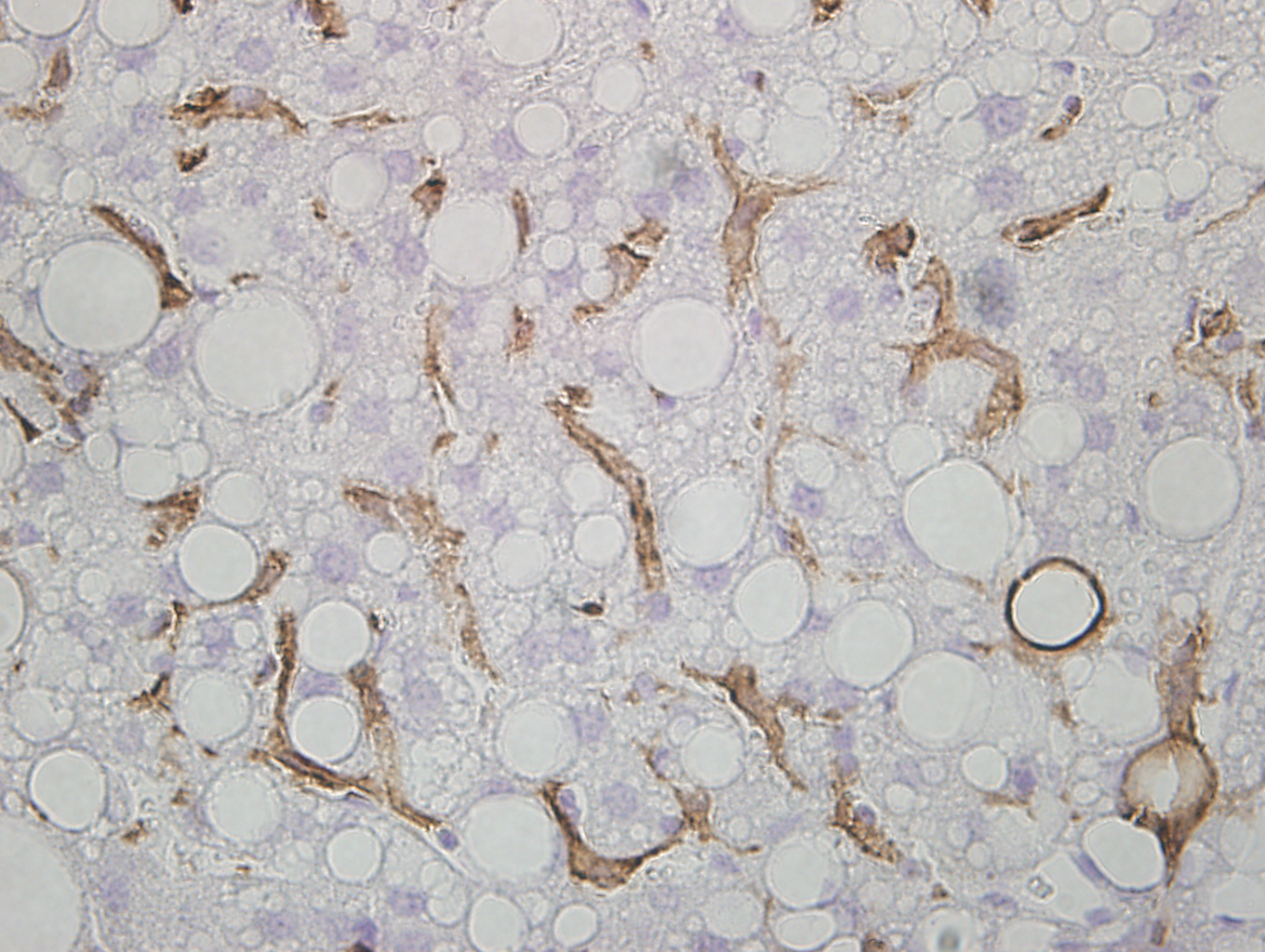

Supplement: Supplementary file 10 — Source data Fig. 7 [file 44318_2026_754_MOESM10_ESM.zip › Figure 7/7N,P/7N_image_CTL_F480.tif]

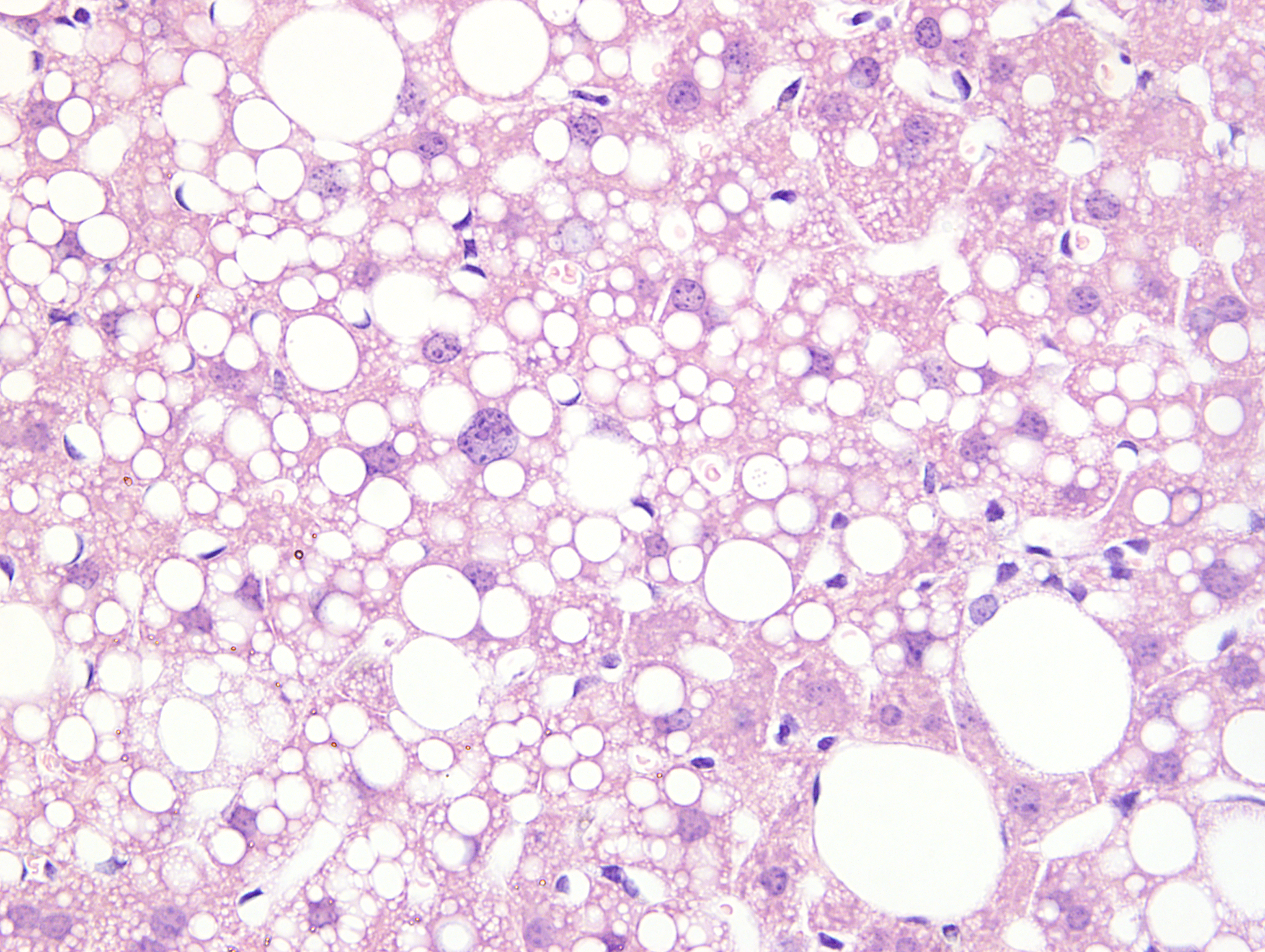

Supplement: Supplementary file 10 — Source data Fig. 7 [file 44318_2026_754_MOESM10_ESM.zip › Figure 7/7N,P/7N_image_CTL_HE.tif]

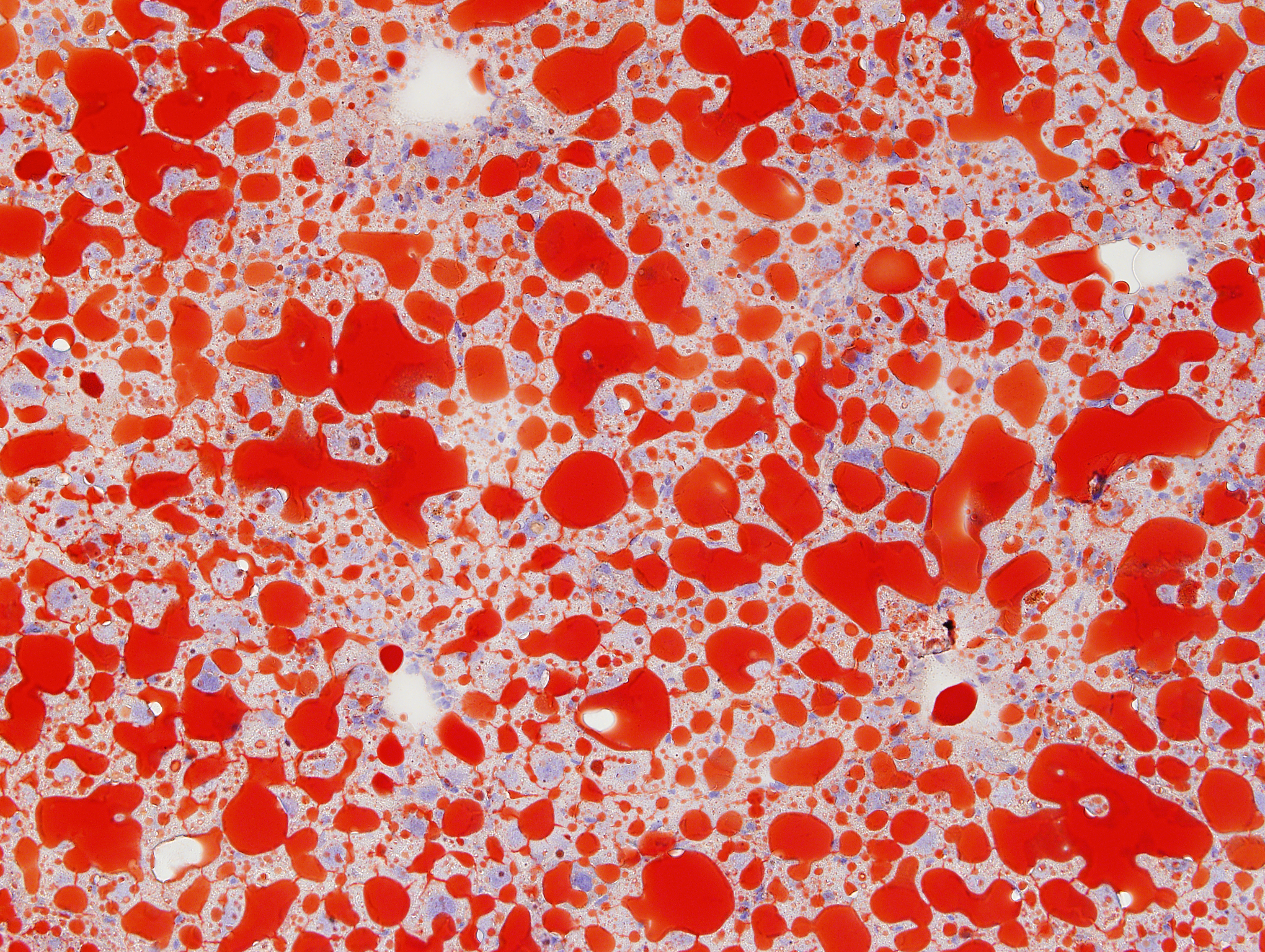

Supplement: Supplementary file 10 — Source data Fig. 7 [file 44318_2026_754_MOESM10_ESM.zip › Figure 7/7N,P/7N_image_CTL_ORO.tif]

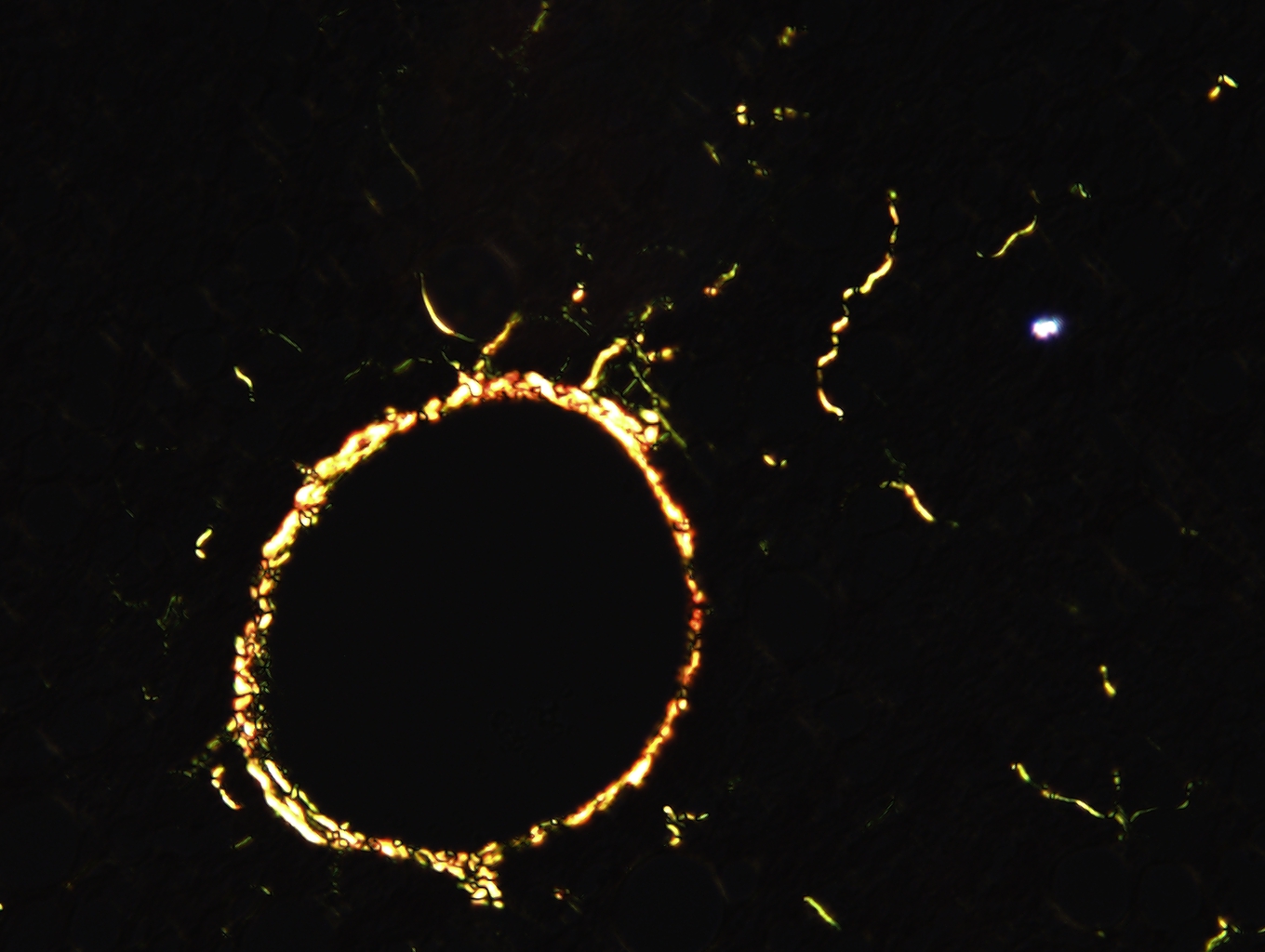

Supplement: Supplementary file 10 — Source data Fig. 7 [file 44318_2026_754_MOESM10_ESM.zip › Figure 7/7N,P/7N_image_CTL_Sirius Red Pol..jpg]

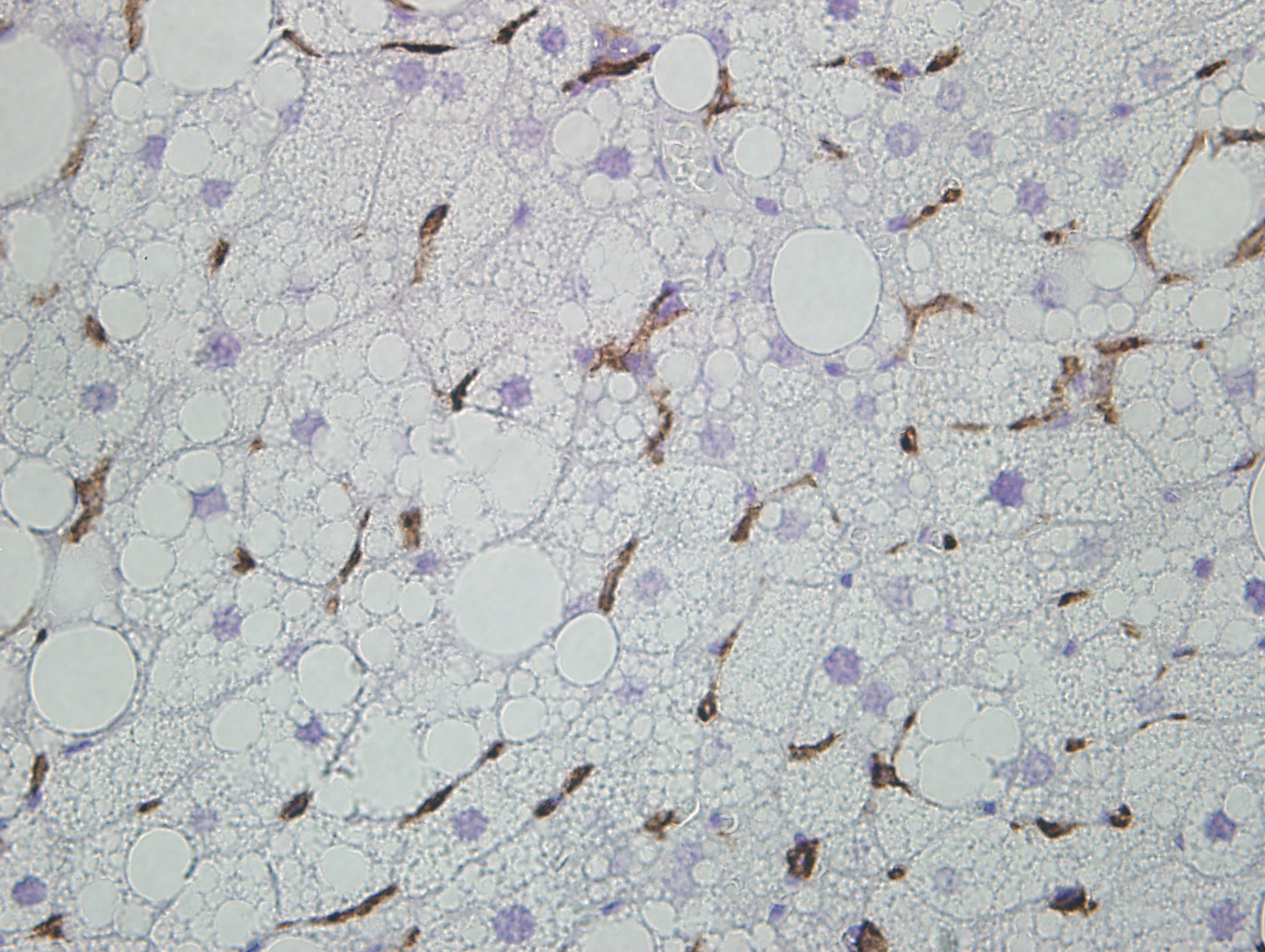

Supplement: Supplementary file 10 — Source data Fig. 7 [file 44318_2026_754_MOESM10_ESM.zip › Figure 7/7N,P/7N_image_KO_F480.tif]

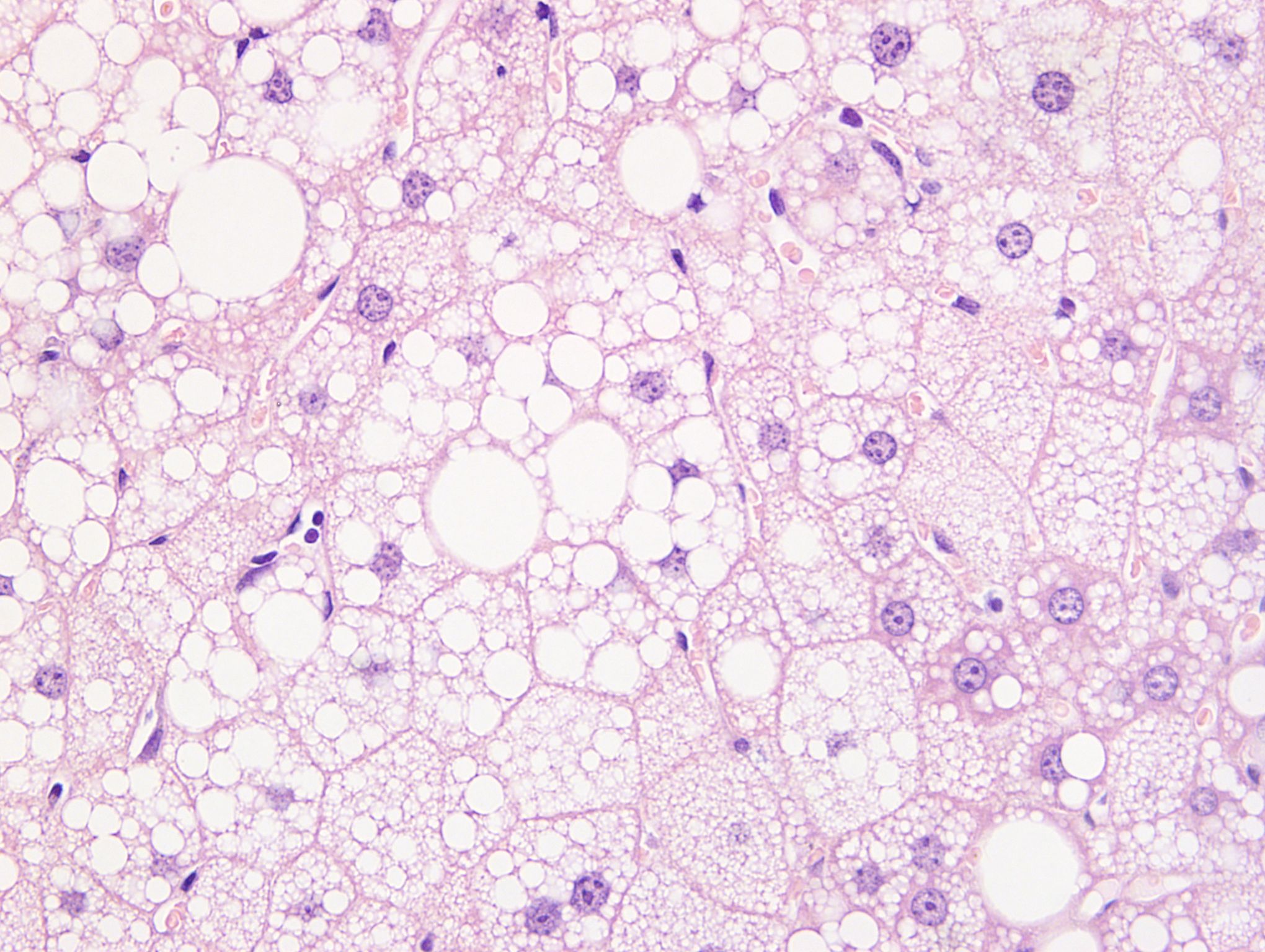

Supplement: Supplementary file 10 — Source data Fig. 7 [file 44318_2026_754_MOESM10_ESM.zip › Figure 7/7N,P/7N_image_KO_HE.tif]

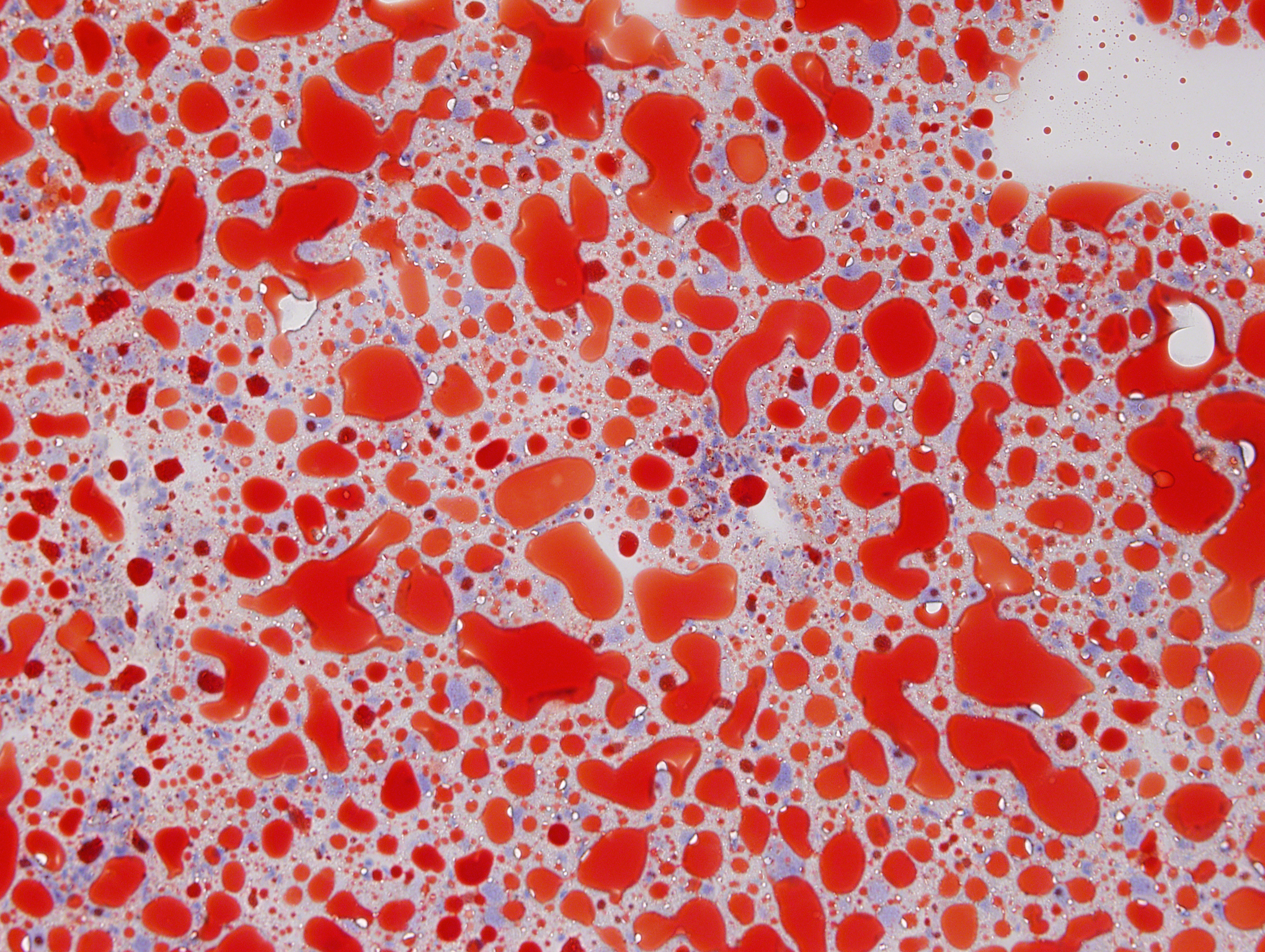

Supplement: Supplementary file 10 — Source data Fig. 7 [file 44318_2026_754_MOESM10_ESM.zip › Figure 7/7N,P/7N_image_KO_ORO.tif]

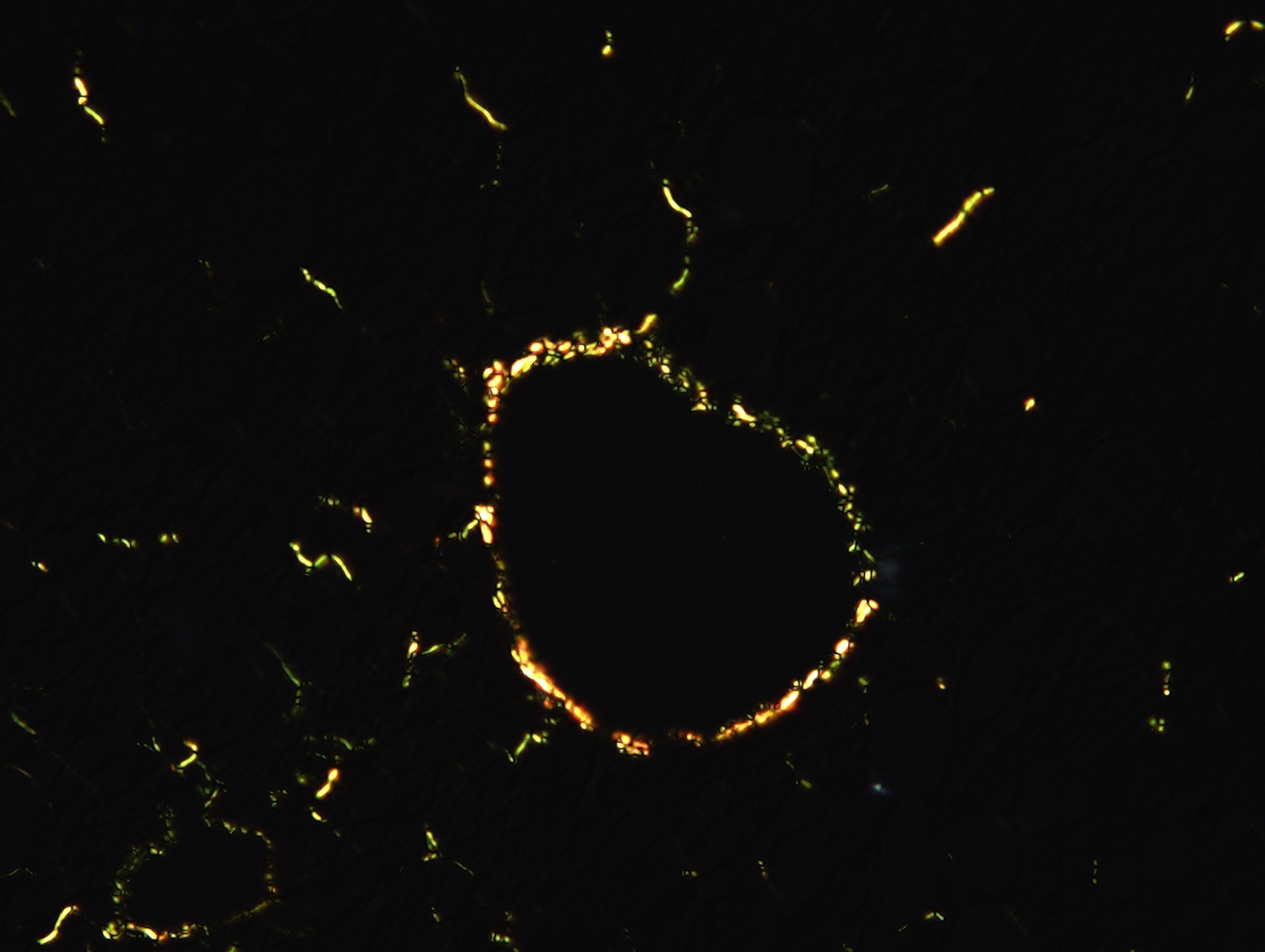

Supplement: Supplementary file 10 — Source data Fig. 7 [file 44318_2026_754_MOESM10_ESM.zip › Figure 7/7N,P/7N_image_KO_Sirius Red Pol..jpg]

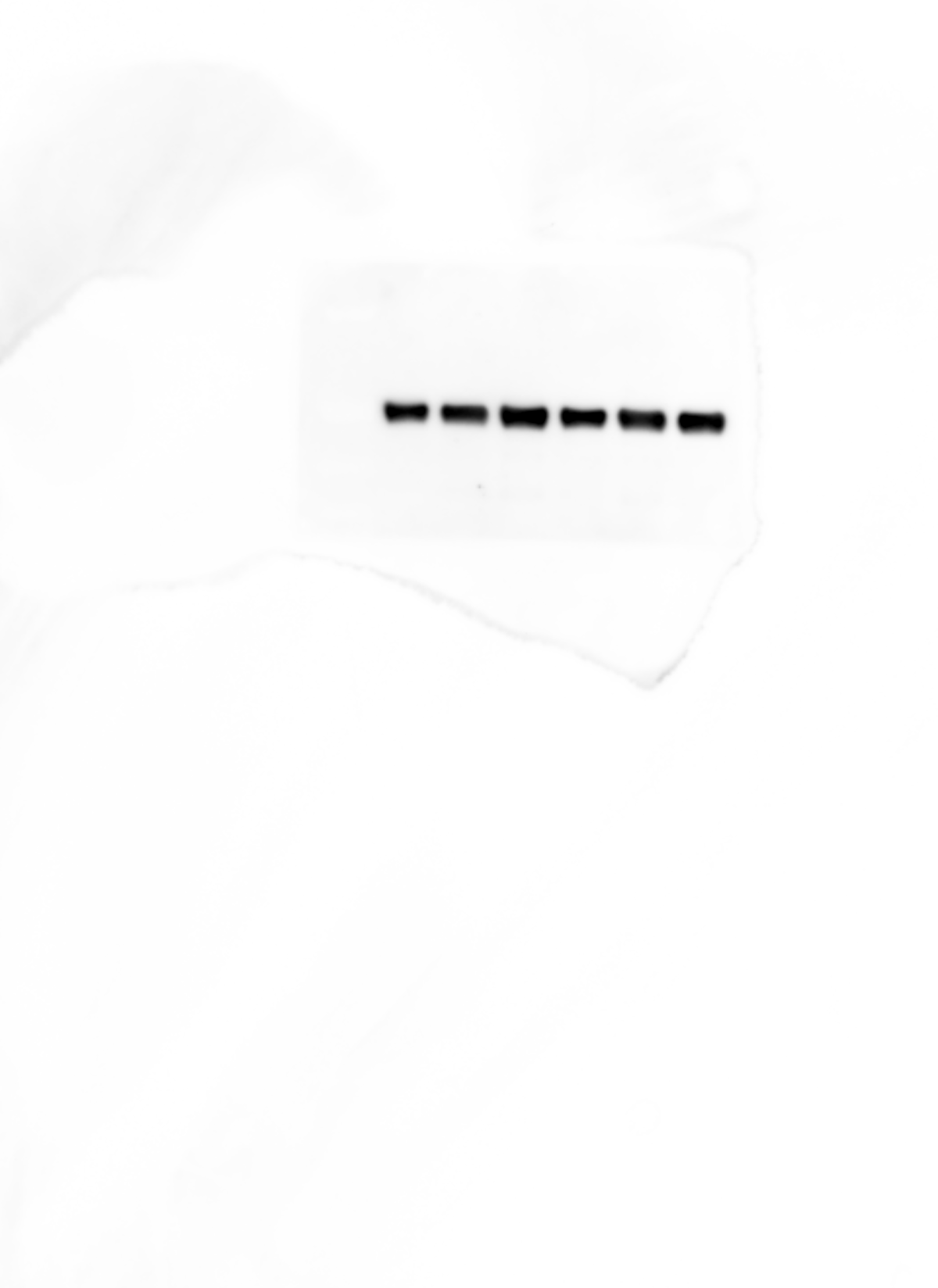

Supplement: Supplementary file 11 — Figure EV2 Source Data [file 44318_2026_754_MOESM11_ESM.zip › EV Figure2/EV 2A/EV2A_western_PCSK9.tif]

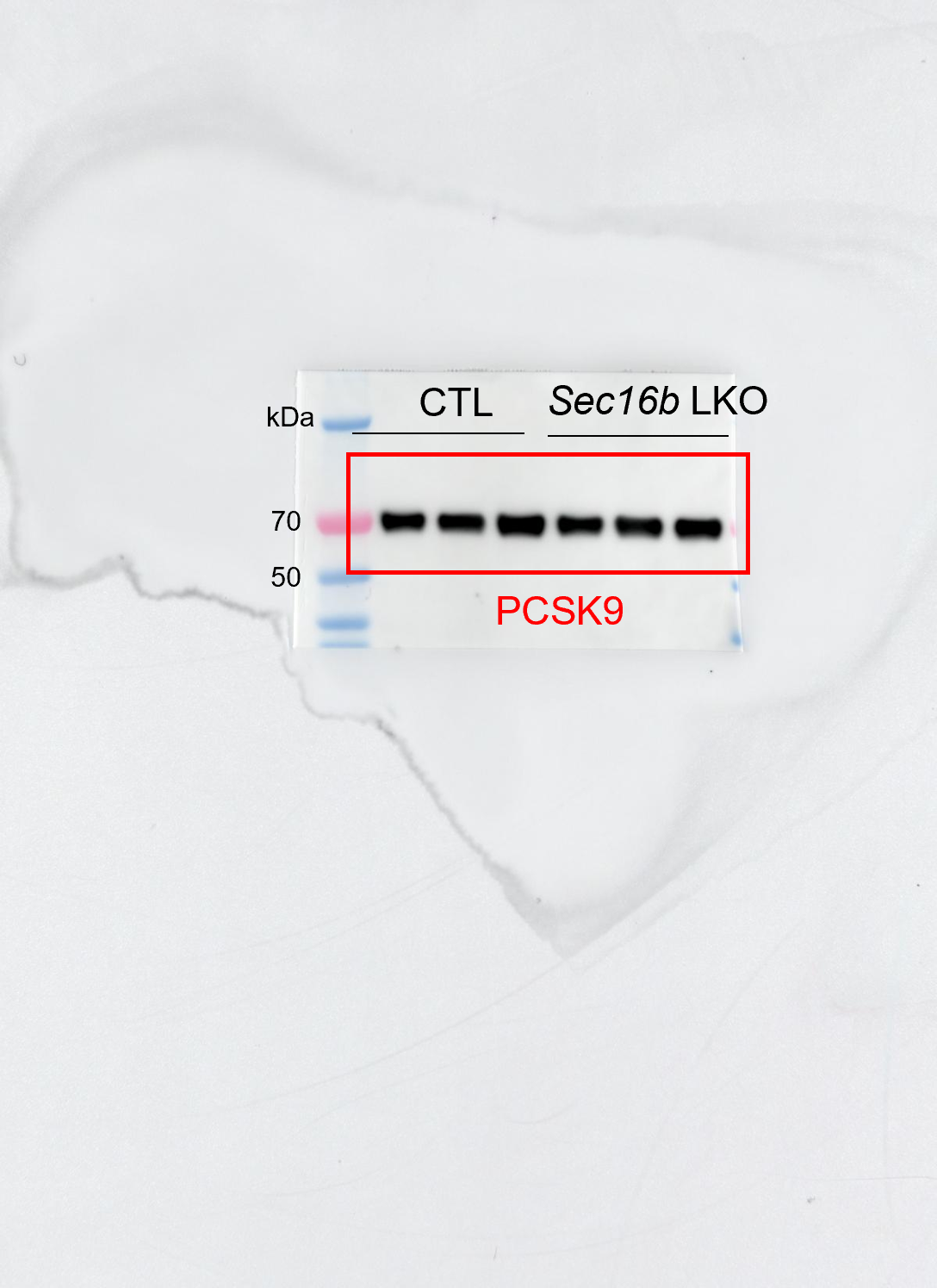

Supplement: Supplementary file 11 — Figure EV2 Source Data [file 44318_2026_754_MOESM11_ESM.zip › EV Figure2/EV 2A/EV2A_western_PCSK9_label.tif]

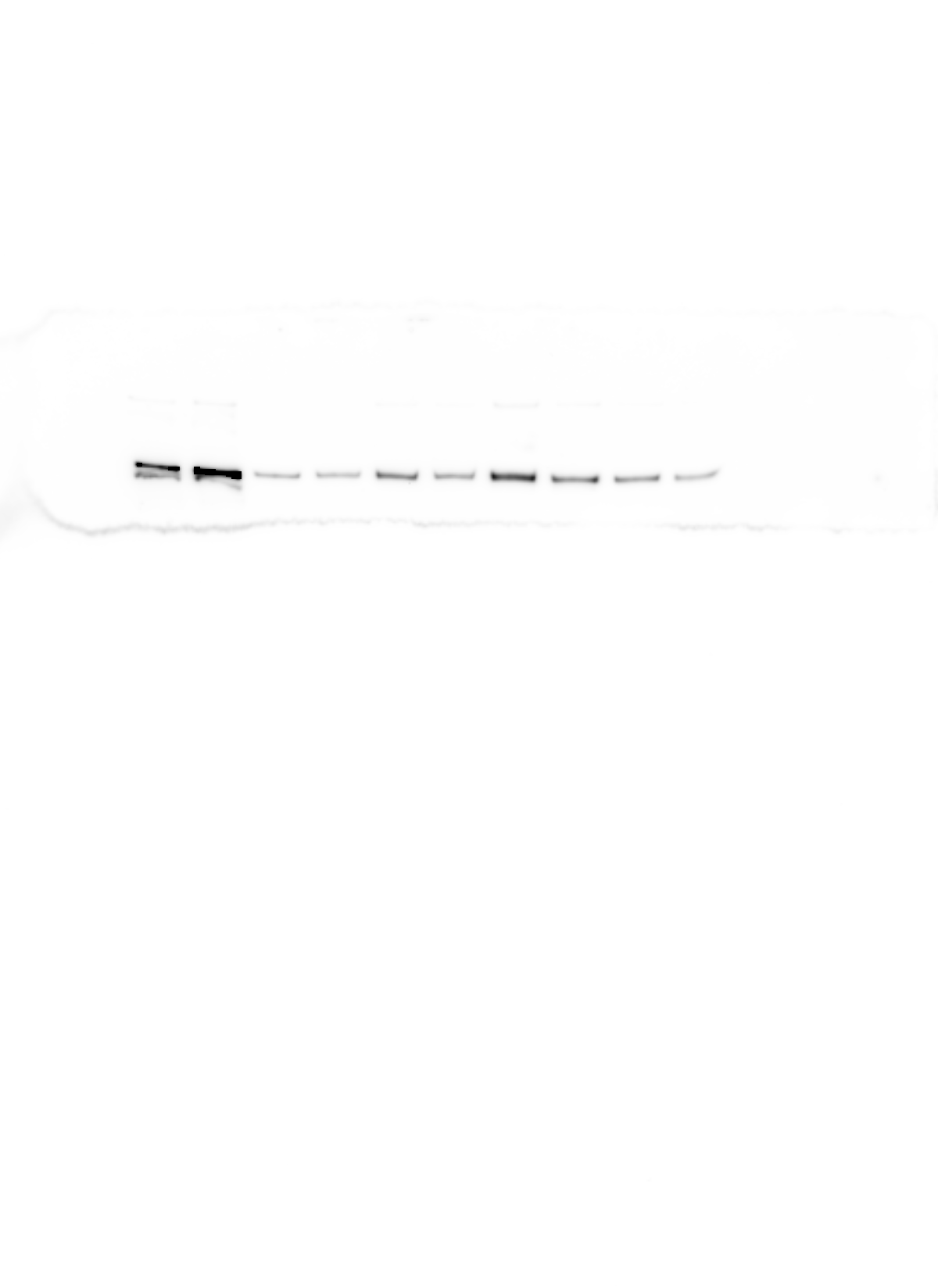

Supplement: Supplementary file 11 — Figure EV2 Source Data [file 44318_2026_754_MOESM11_ESM.zip › EV Figure2/EV 2C/EV2C_western_APOB.tif]

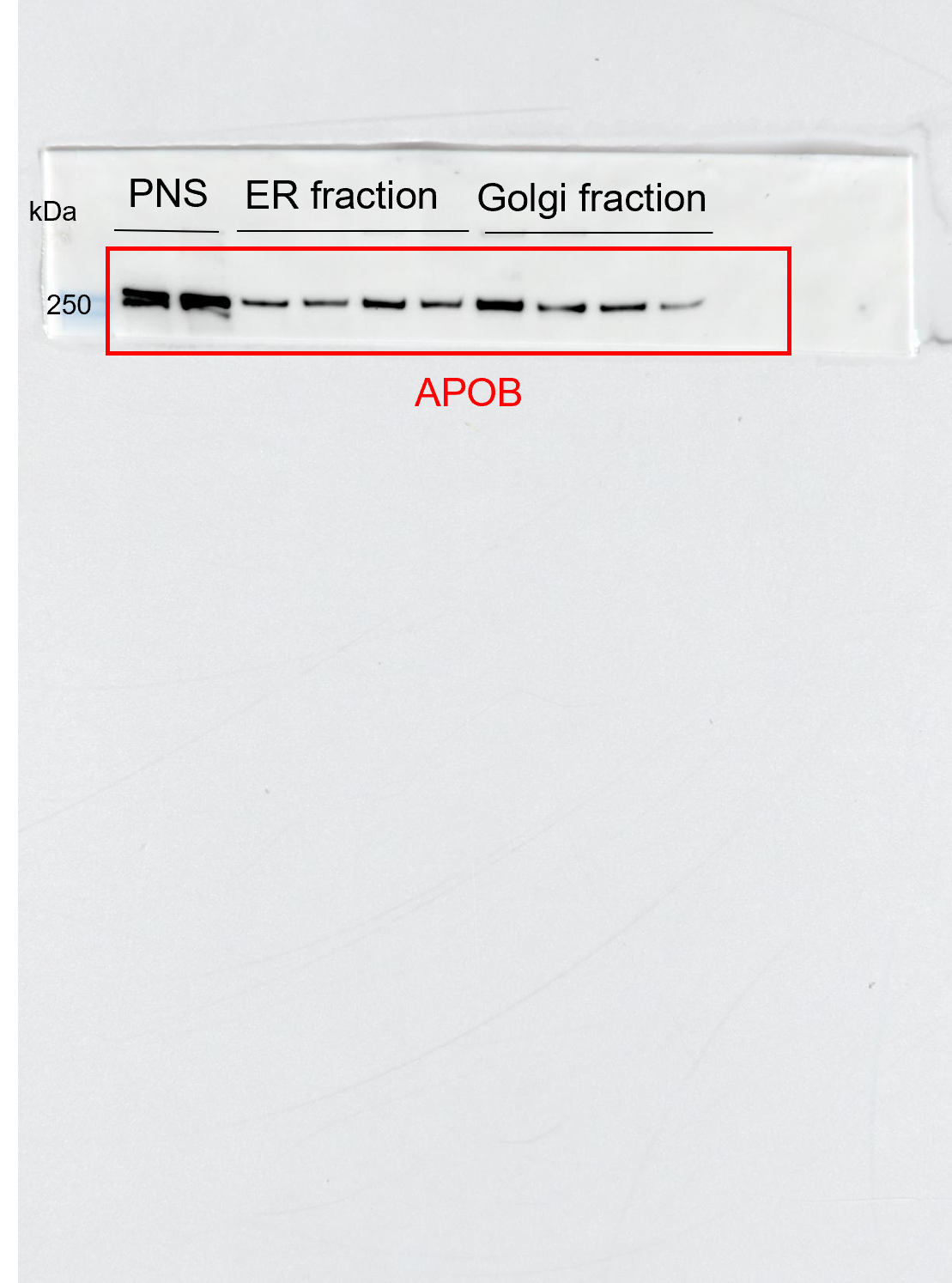

Supplement: Supplementary file 11 — Figure EV2 Source Data [file 44318_2026_754_MOESM11_ESM.zip › EV Figure2/EV 2C/EV2C_western_APOB_label.tif]

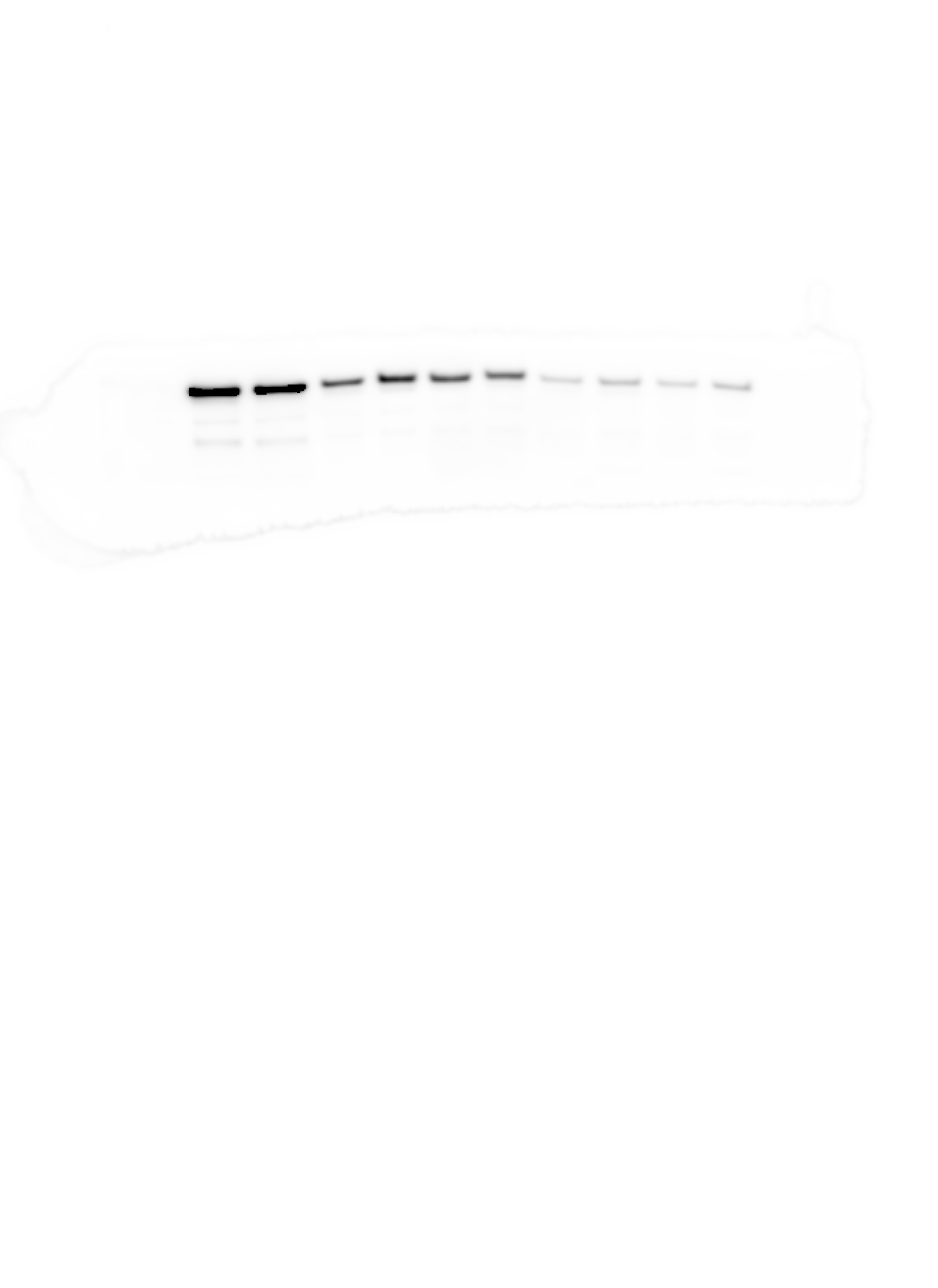

Supplement: Supplementary file 11 — Figure EV2 Source Data [file 44318_2026_754_MOESM11_ESM.zip › EV Figure2/EV 2C/EV2C_western_CNX.tif]

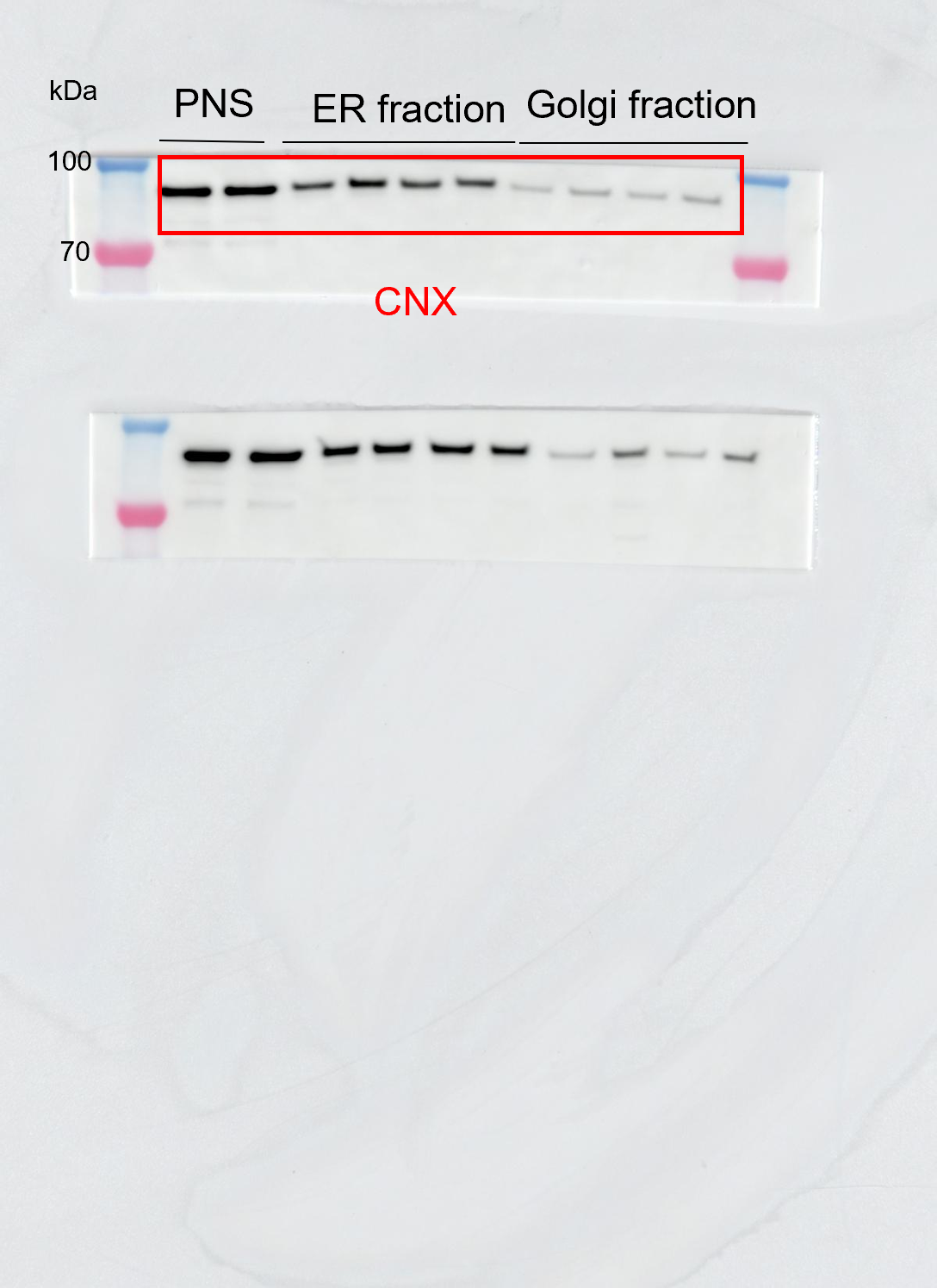

Supplement: Supplementary file 11 — Figure EV2 Source Data [file 44318_2026_754_MOESM11_ESM.zip › EV Figure2/EV 2C/EV2C_western_CNX_label.tif]

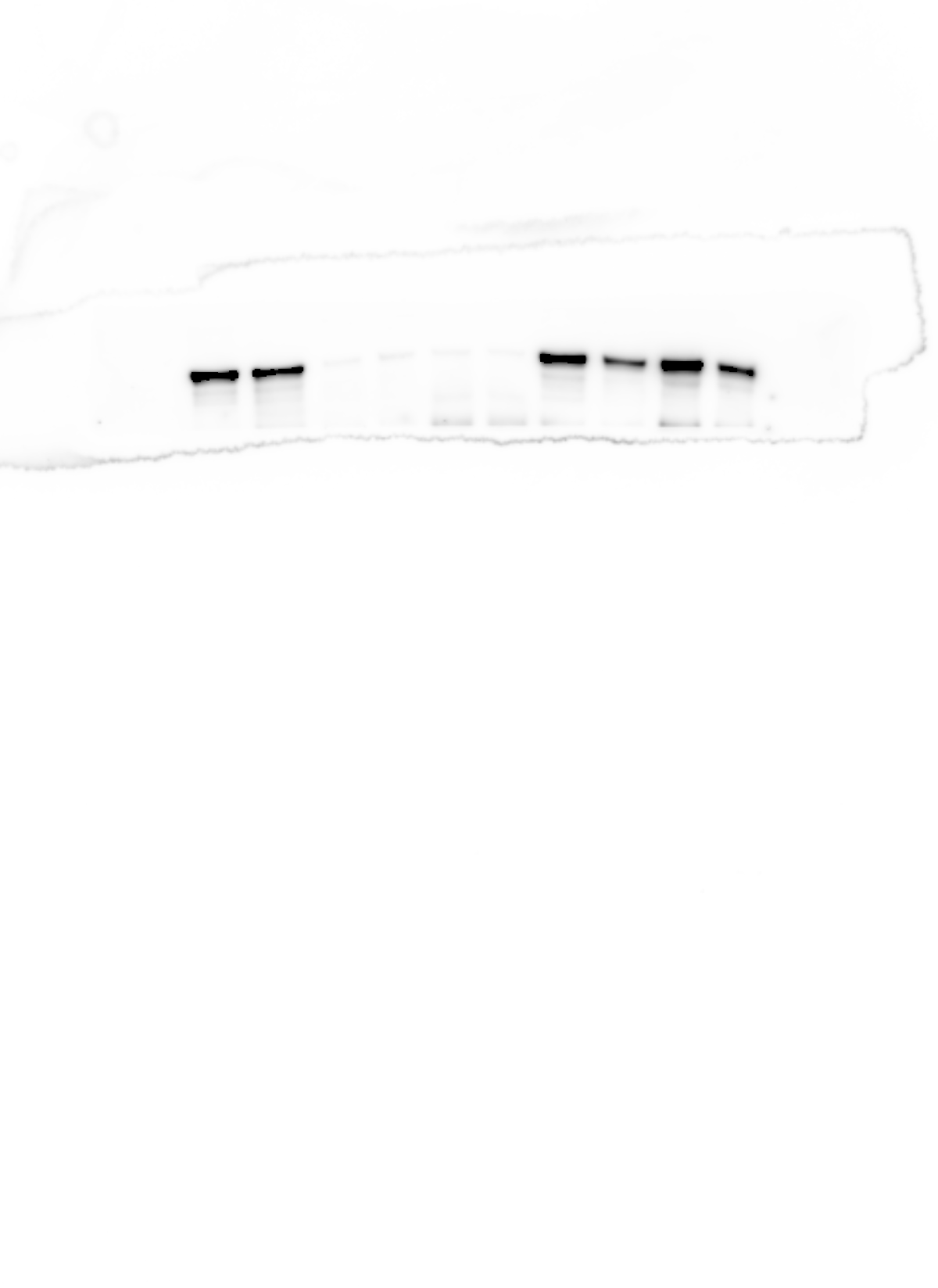

Supplement: Supplementary file 11 — Figure EV2 Source Data [file 44318_2026_754_MOESM11_ESM.zip › EV Figure2/EV 2C/EV2C_western_GM130.tif]

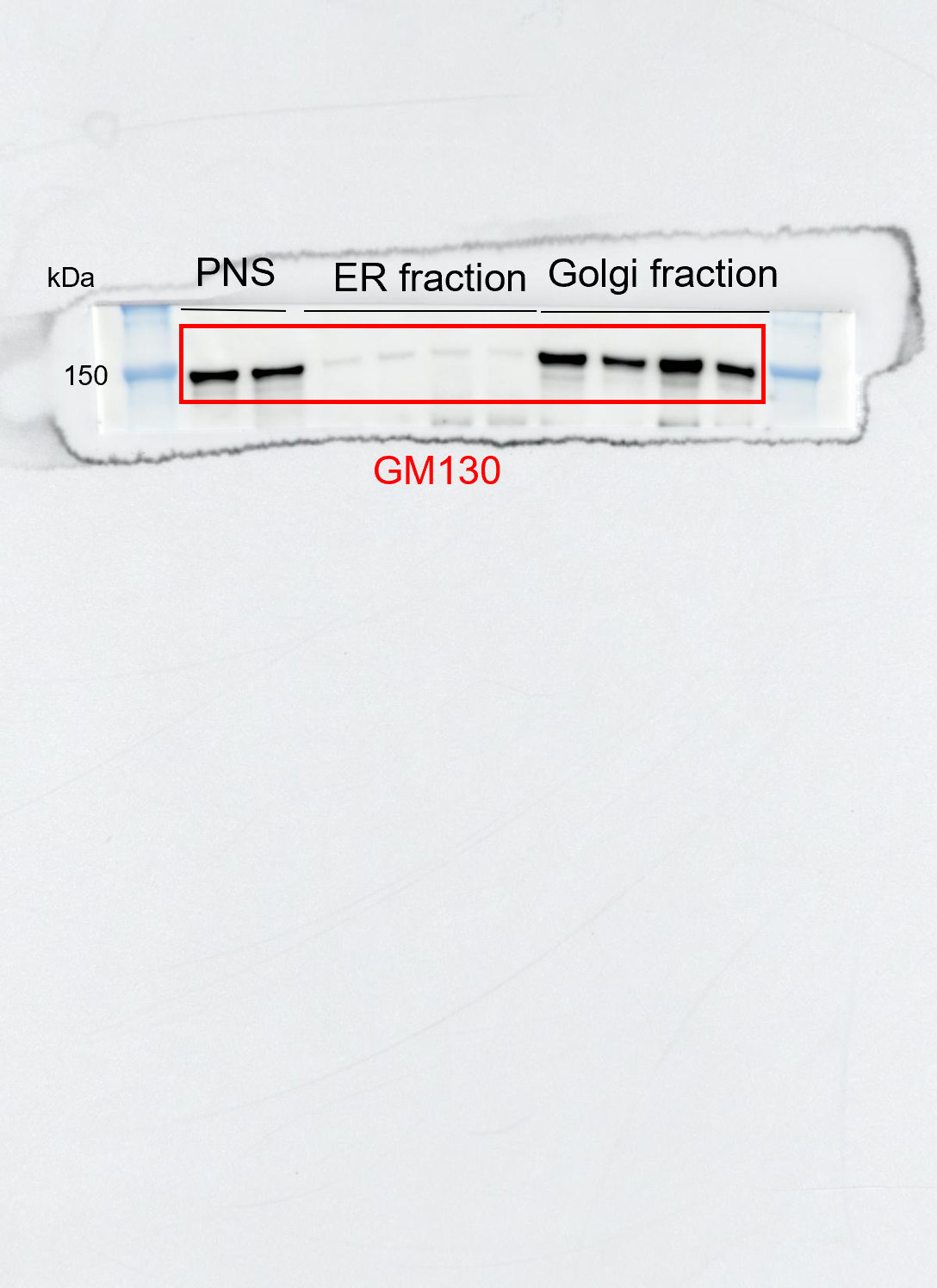

Supplement: Supplementary file 11 — Figure EV2 Source Data [file 44318_2026_754_MOESM11_ESM.zip › EV Figure2/EV 2C/EV2C_western_GM130_label.tif]

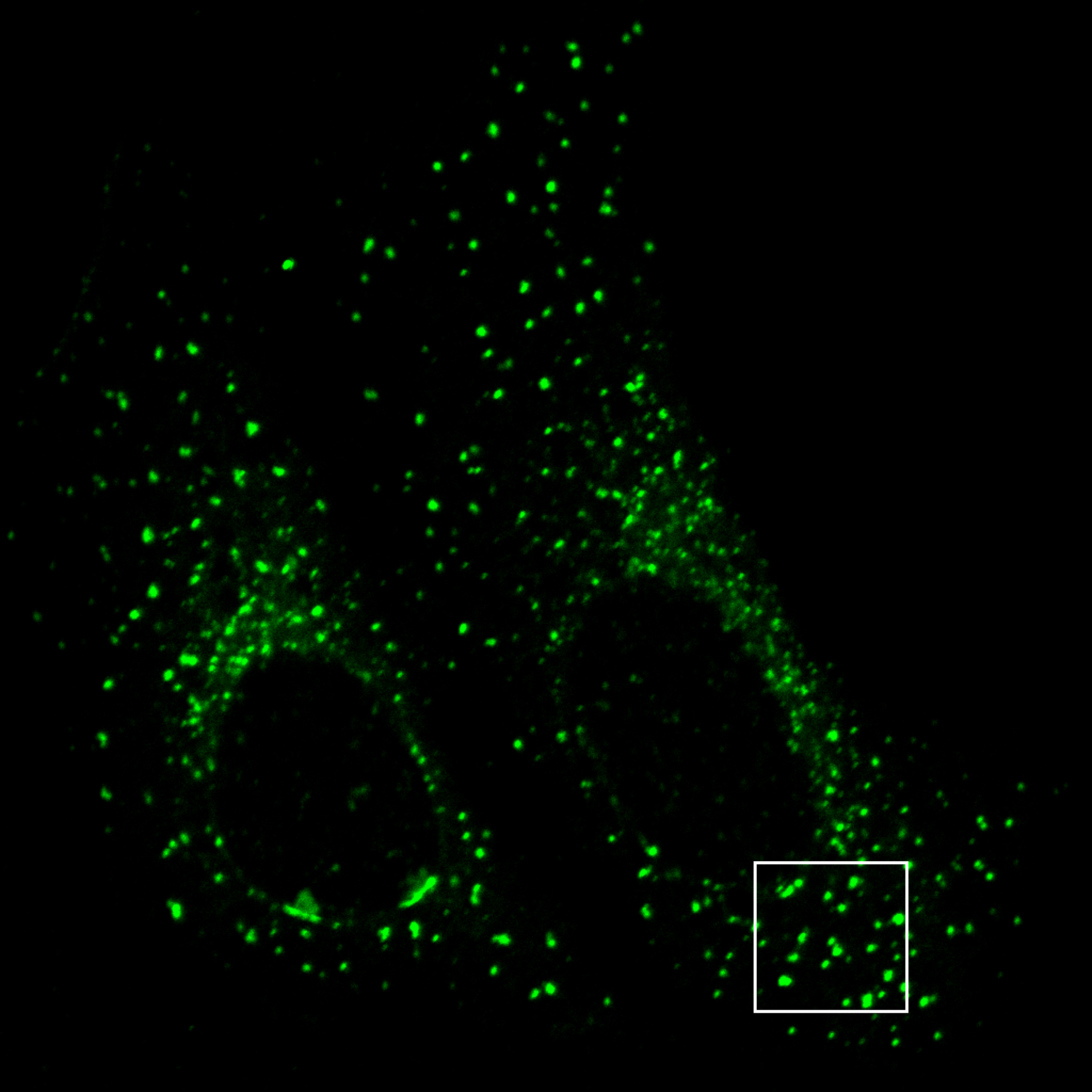

Supplement: Supplementary file 11 — Figure EV2 Source Data [file 44318_2026_754_MOESM11_ESM.zip › EV Figure2/EV 2F/EV2F_image_GFP-SEC16B_label.tif]

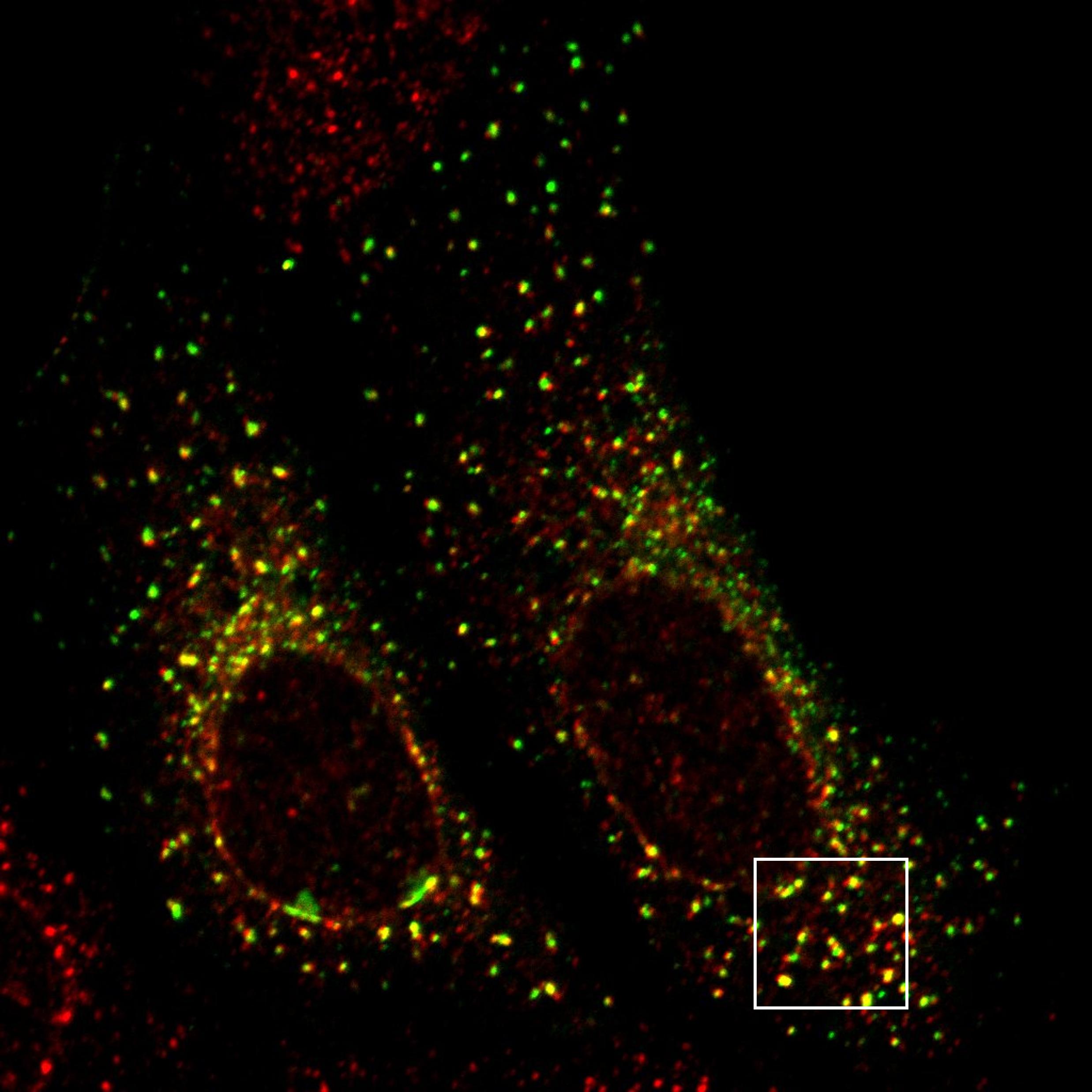

Supplement: Supplementary file 11 — Figure EV2 Source Data [file 44318_2026_754_MOESM11_ESM.zip › EV Figure2/EV 2F/EV2F_image_Merge_label.tif]

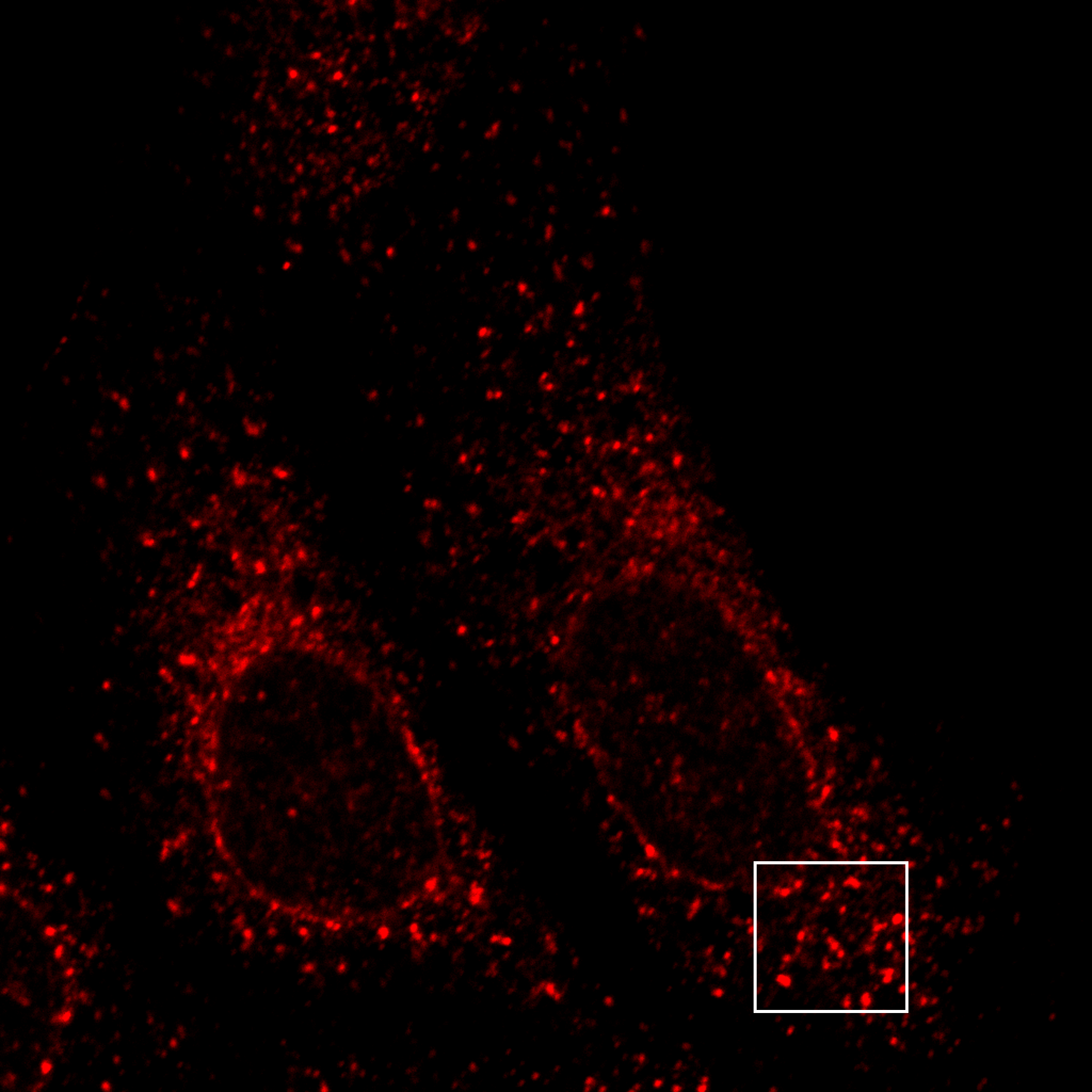

Supplement: Supplementary file 11 — Figure EV2 Source Data [file 44318_2026_754_MOESM11_ESM.zip › EV Figure2/EV 2F/EV2F_image_SURF4_label.tif]

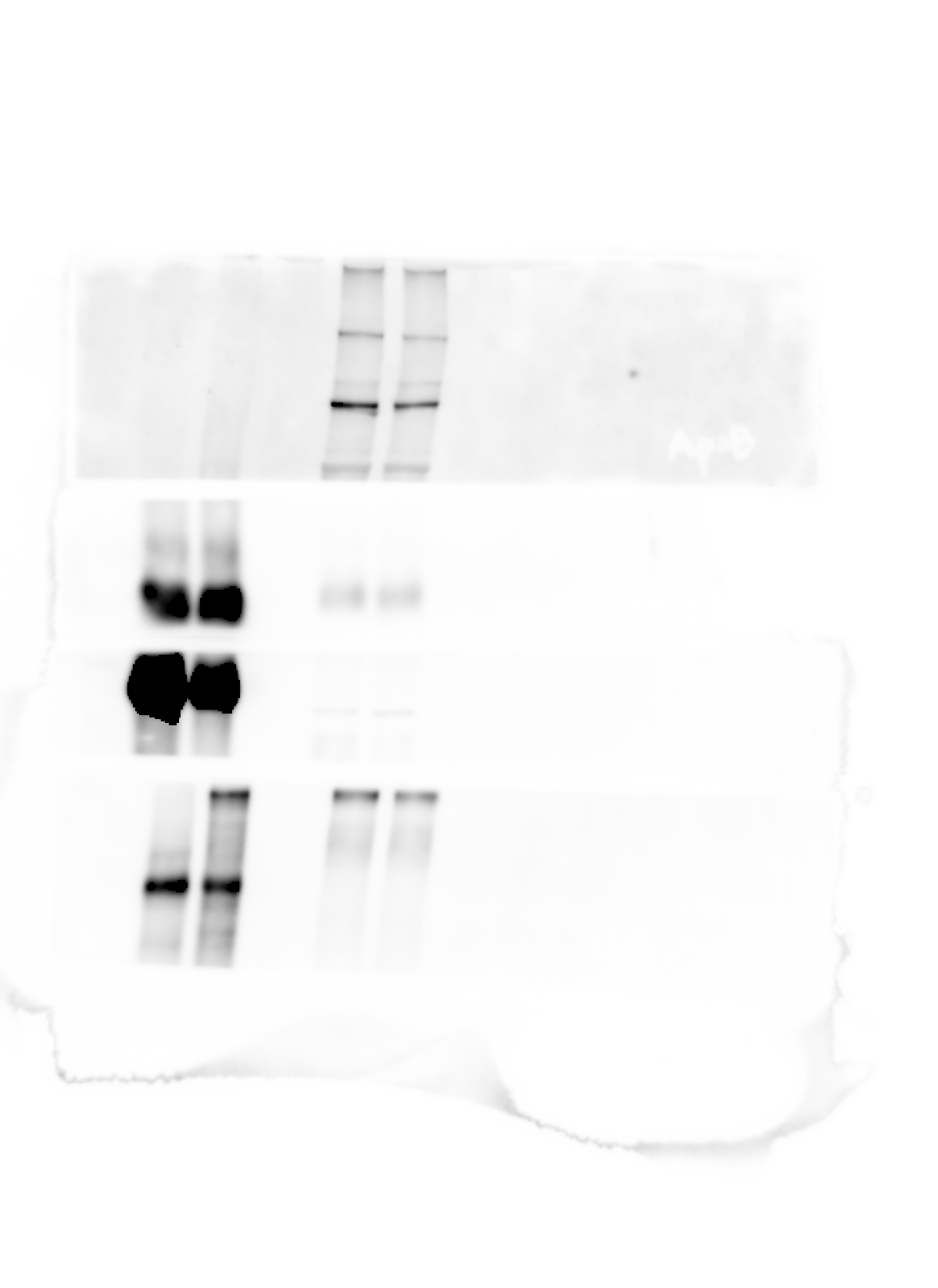

Supplement: Supplementary file 11 — Figure EV2 Source Data [file 44318_2026_754_MOESM11_ESM.zip › EV Figure2/EV 2G/EV2G_western_APOB_SEC16B.tif]

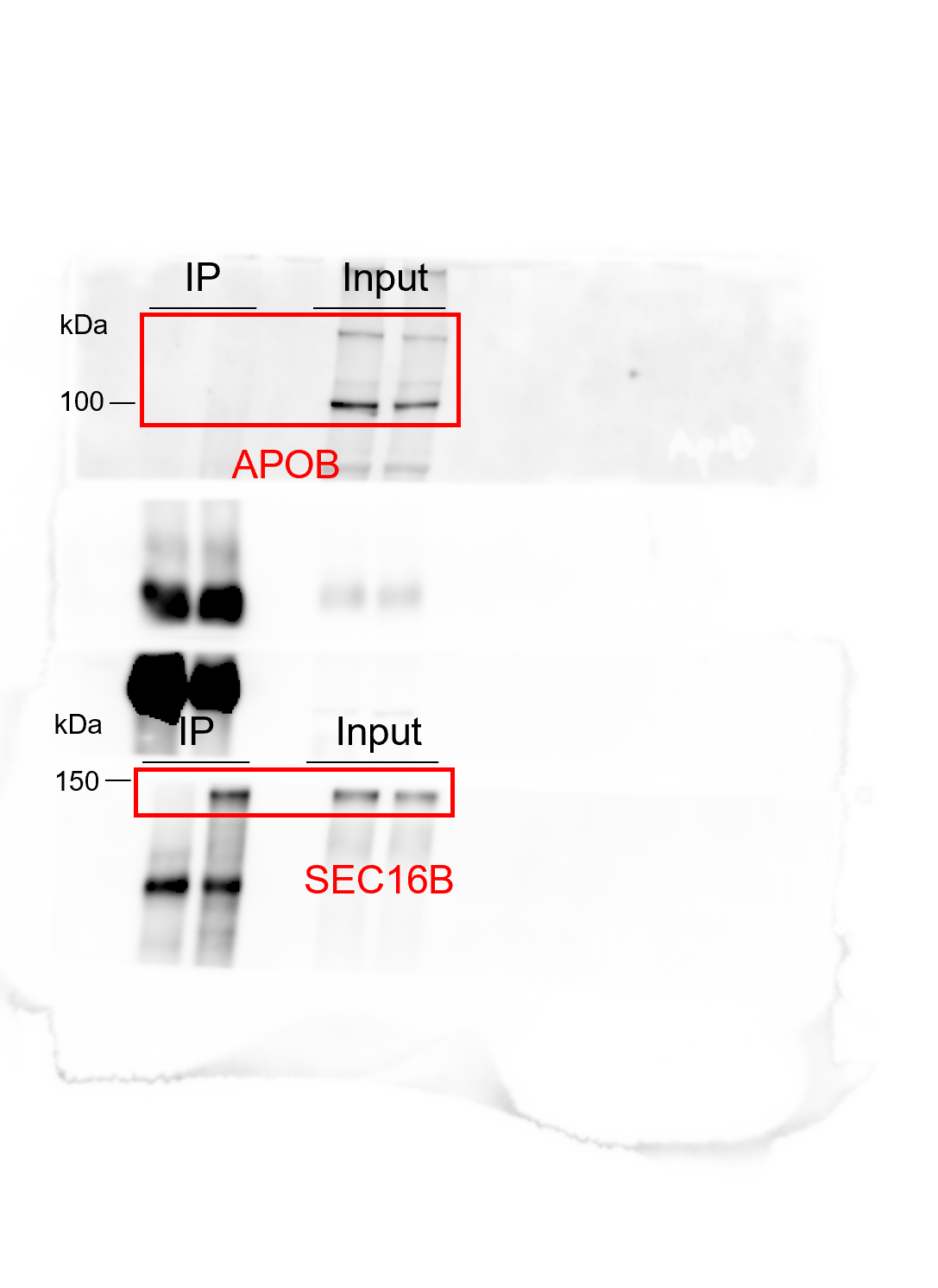

Supplement: Supplementary file 11 — Figure EV2 Source Data [file 44318_2026_754_MOESM11_ESM.zip › EV Figure2/EV 2G/EV2G_western_APOB_SEC16B_label.tif]

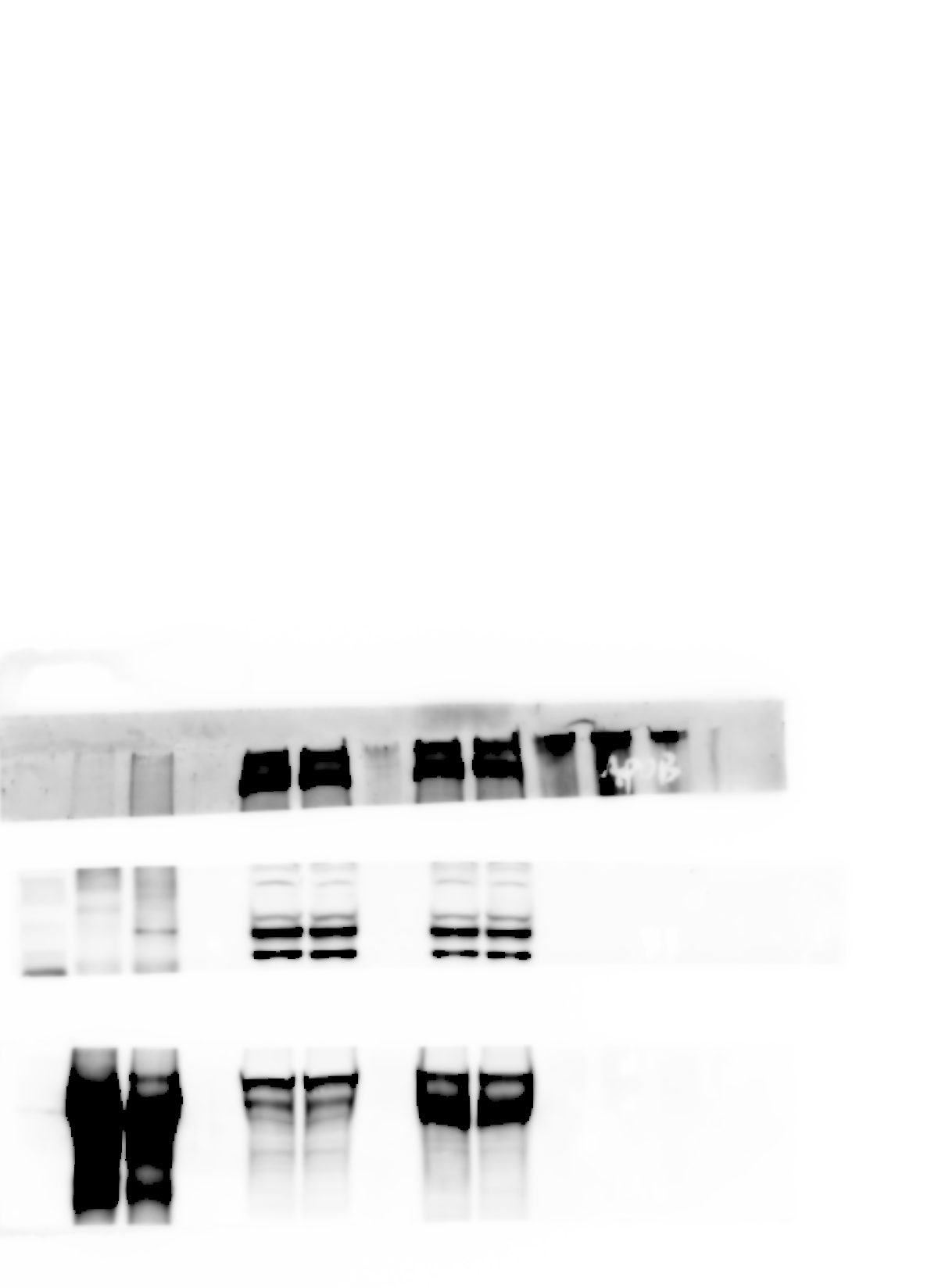

Supplement: Supplementary file 11 — Figure EV2 Source Data [file 44318_2026_754_MOESM11_ESM.zip › EV Figure2/EV 2G/EV2G_western_SEC13.tif]

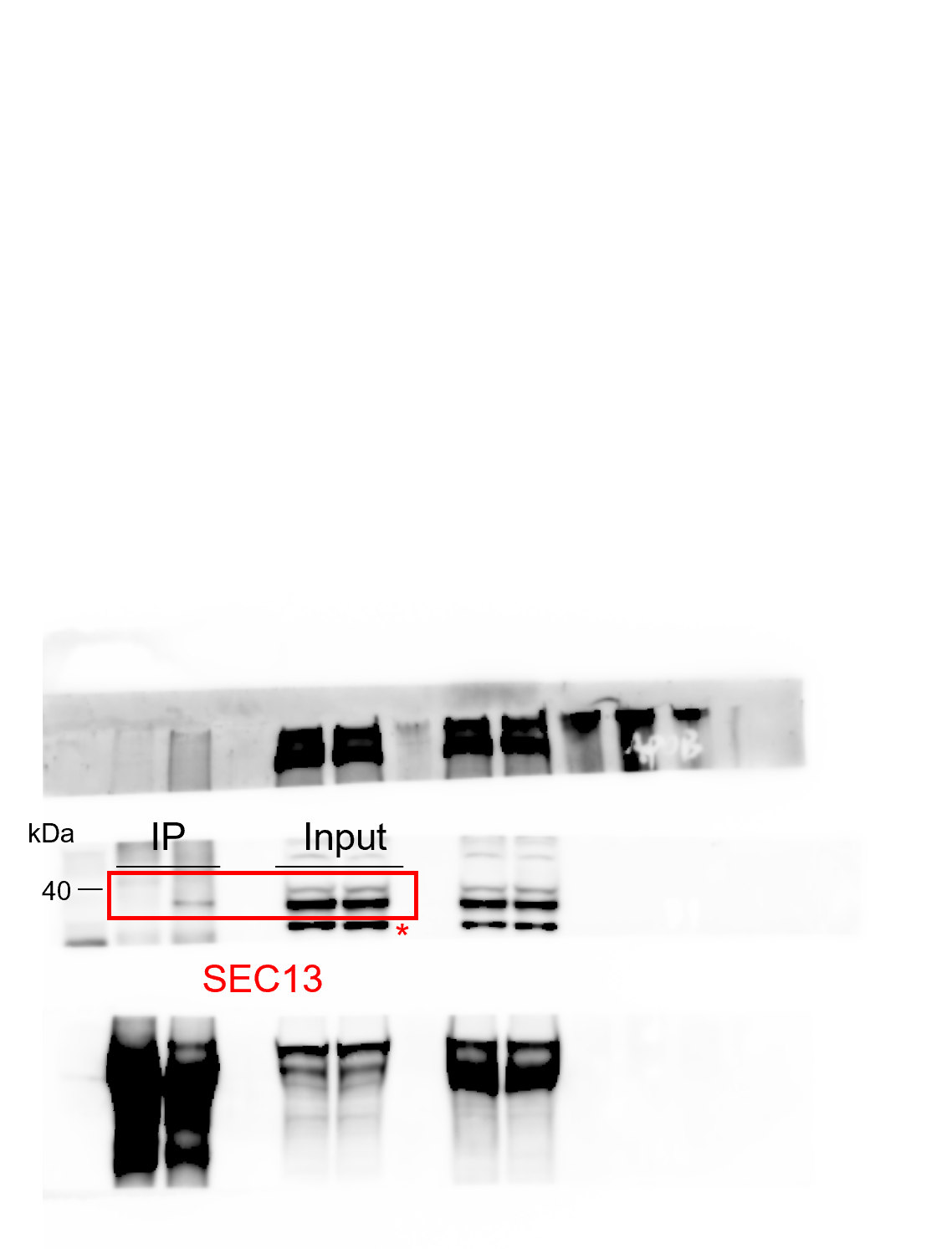

Supplement: Supplementary file 11 — Figure EV2 Source Data [file 44318_2026_754_MOESM11_ESM.zip › EV Figure2/EV 2G/EV2G_western_SEC13_label.tif]

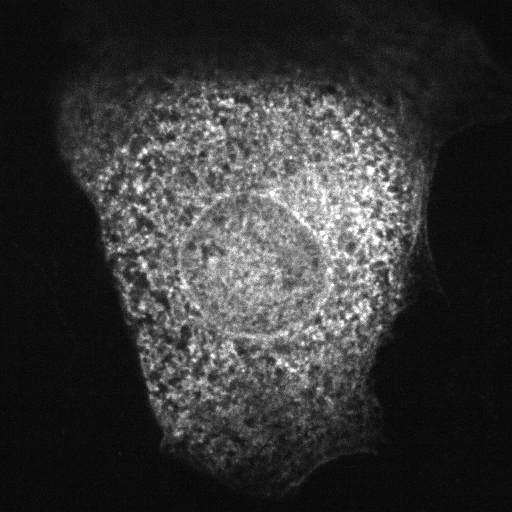

Supplement: Supplementary file 11 — Figure EV2 Source Data [file 44318_2026_754_MOESM11_ESM.zip › EV Figure2/EV 2H/EV2H_image_CTL_RFP-SEC13.tif]

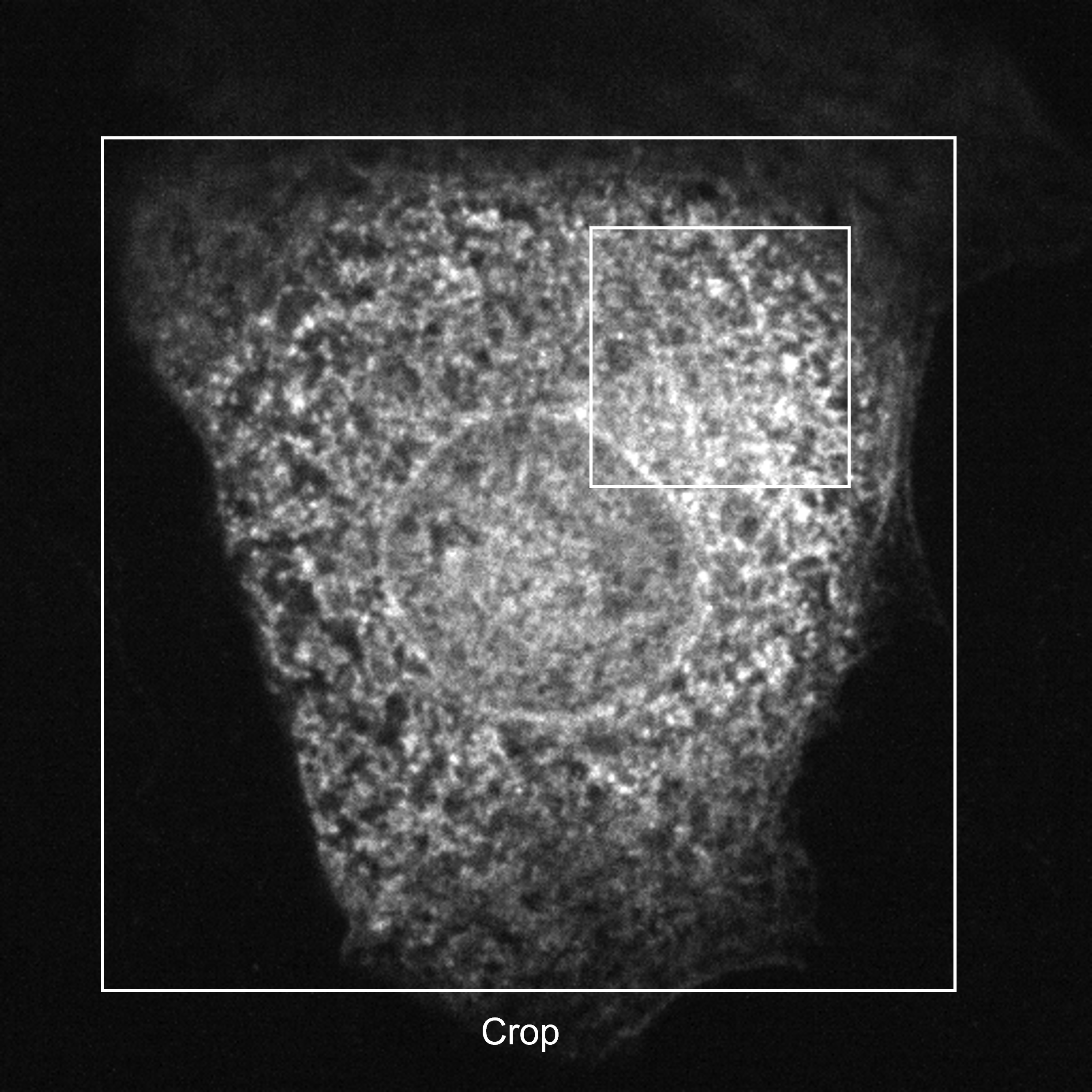

Supplement: Supplementary file 11 — Figure EV2 Source Data [file 44318_2026_754_MOESM11_ESM.zip › EV Figure2/EV 2H/EV2H_image_CTL_RFP-SEC13_label.tif]

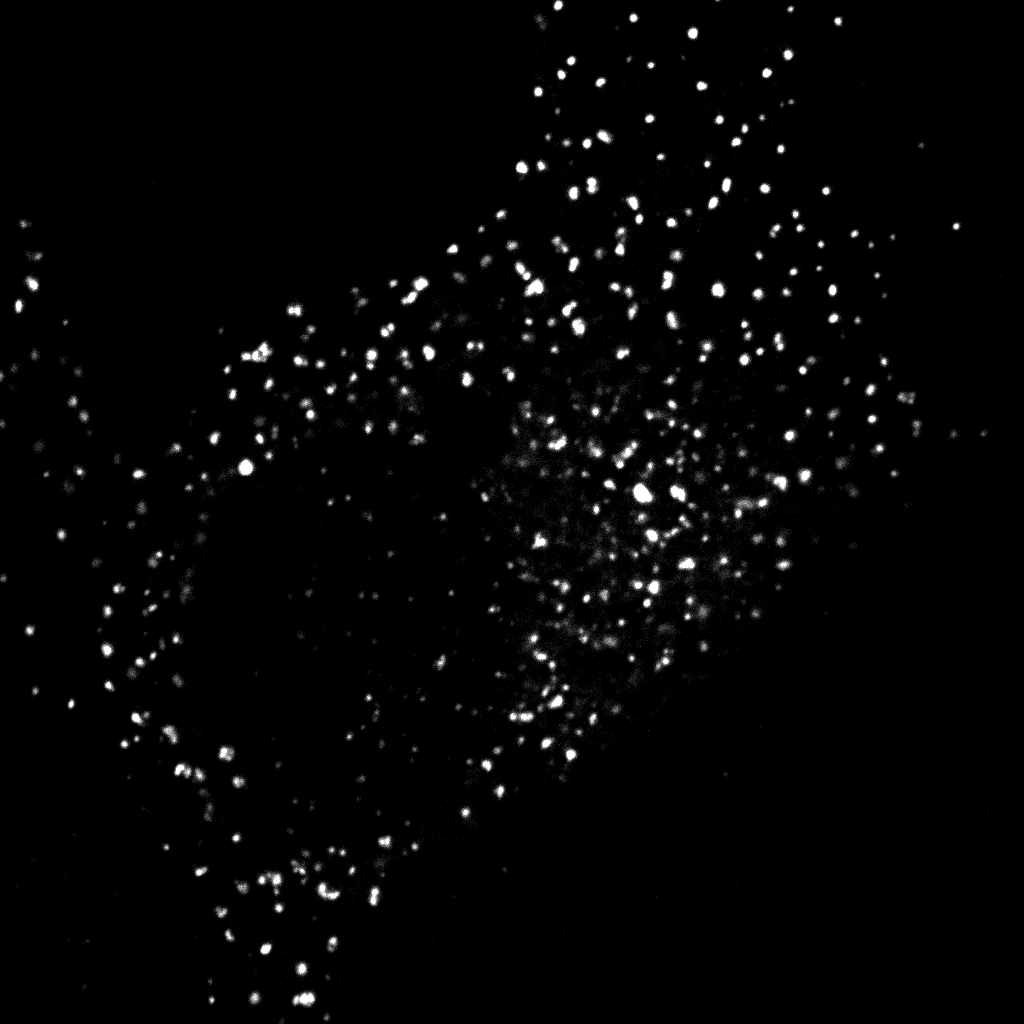

Supplement: Supplementary file 11 — Figure EV2 Source Data [file 44318_2026_754_MOESM11_ESM.zip › EV Figure2/EV 2H/EV2H_image_SEC16B OE_GFP-SEC16B.tif]

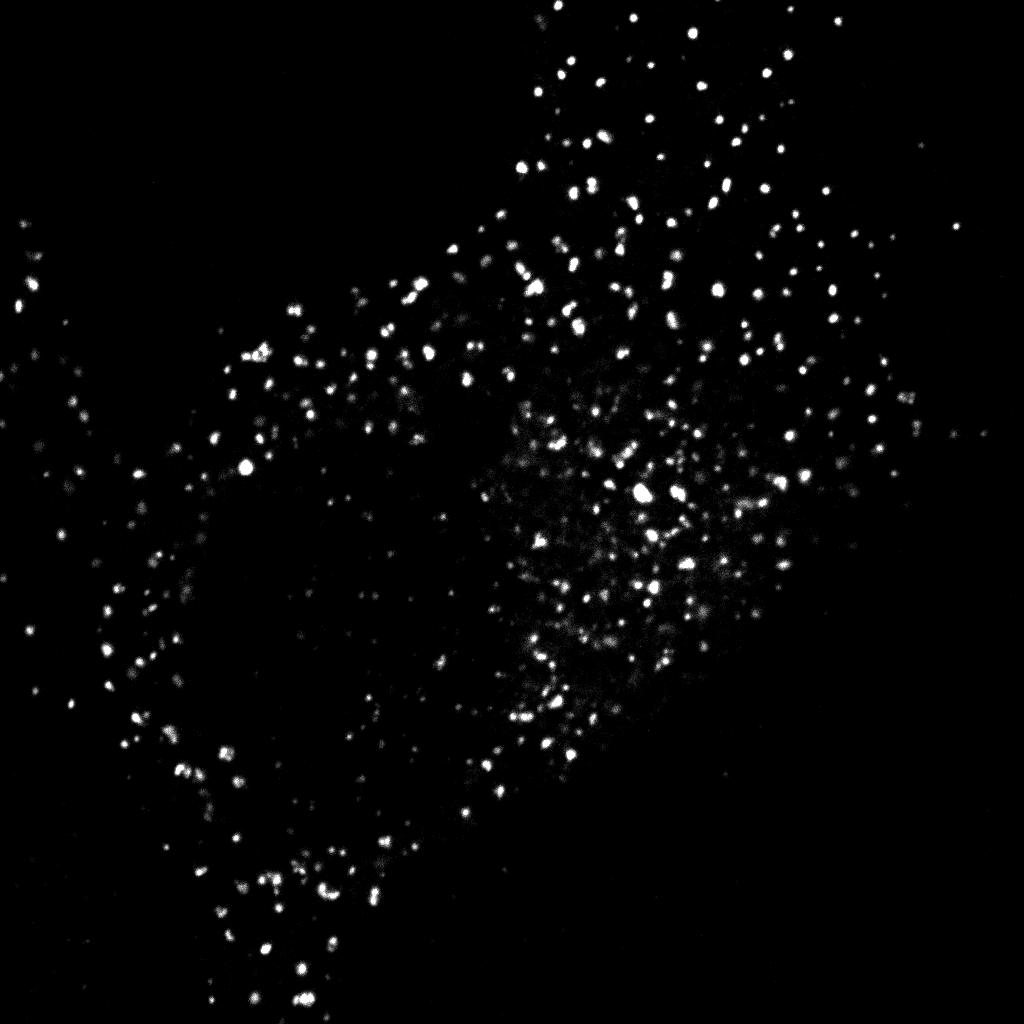

Supplement: Supplementary file 11 — Figure EV2 Source Data [file 44318_2026_754_MOESM11_ESM.zip › EV Figure2/EV 2H/EV2H_image_SEC16B OE_Merge.tif]

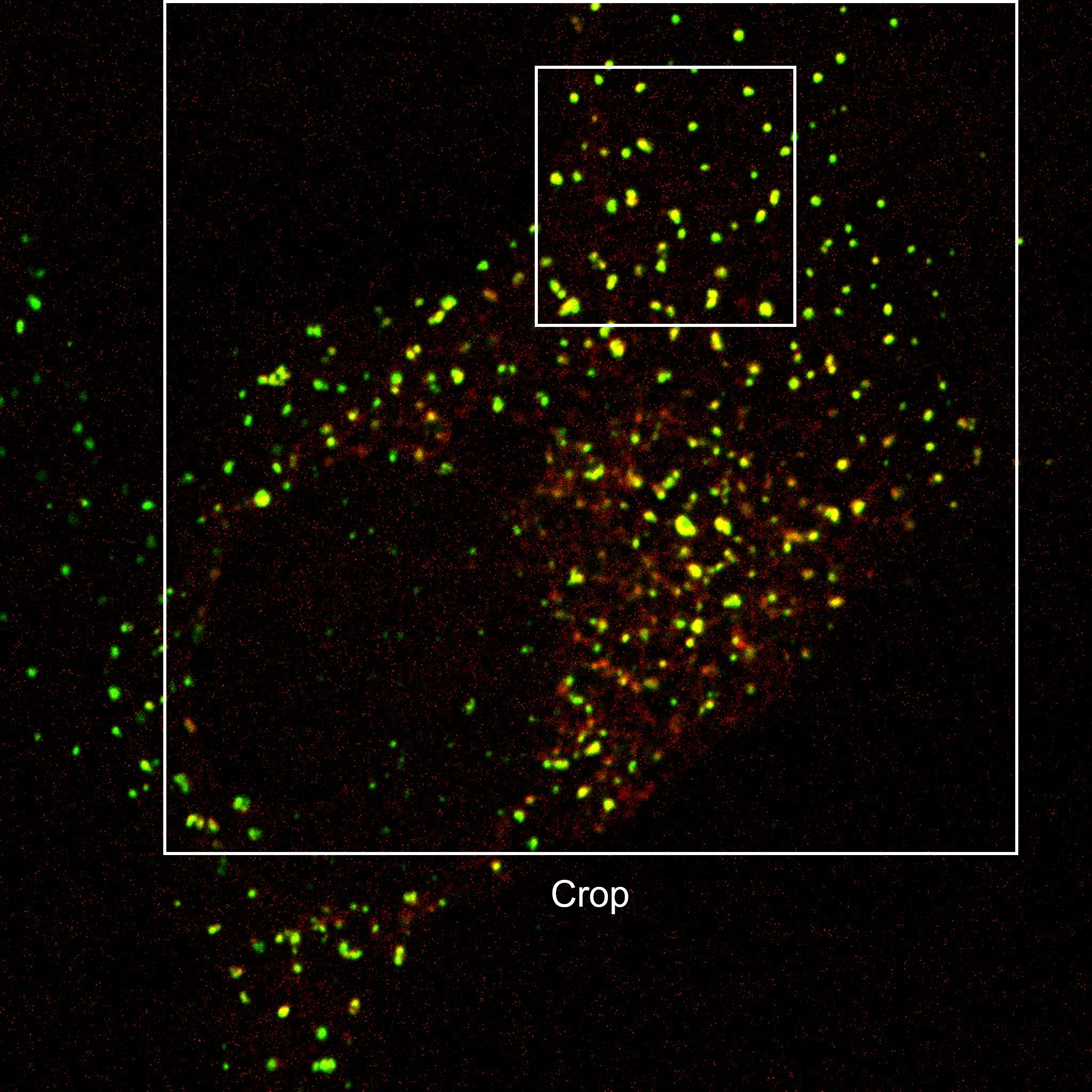

Supplement: Supplementary file 11 — Figure EV2 Source Data [file 44318_2026_754_MOESM11_ESM.zip › EV Figure2/EV 2H/EV2H_image_SEC16B OE_Merge_label.tif]

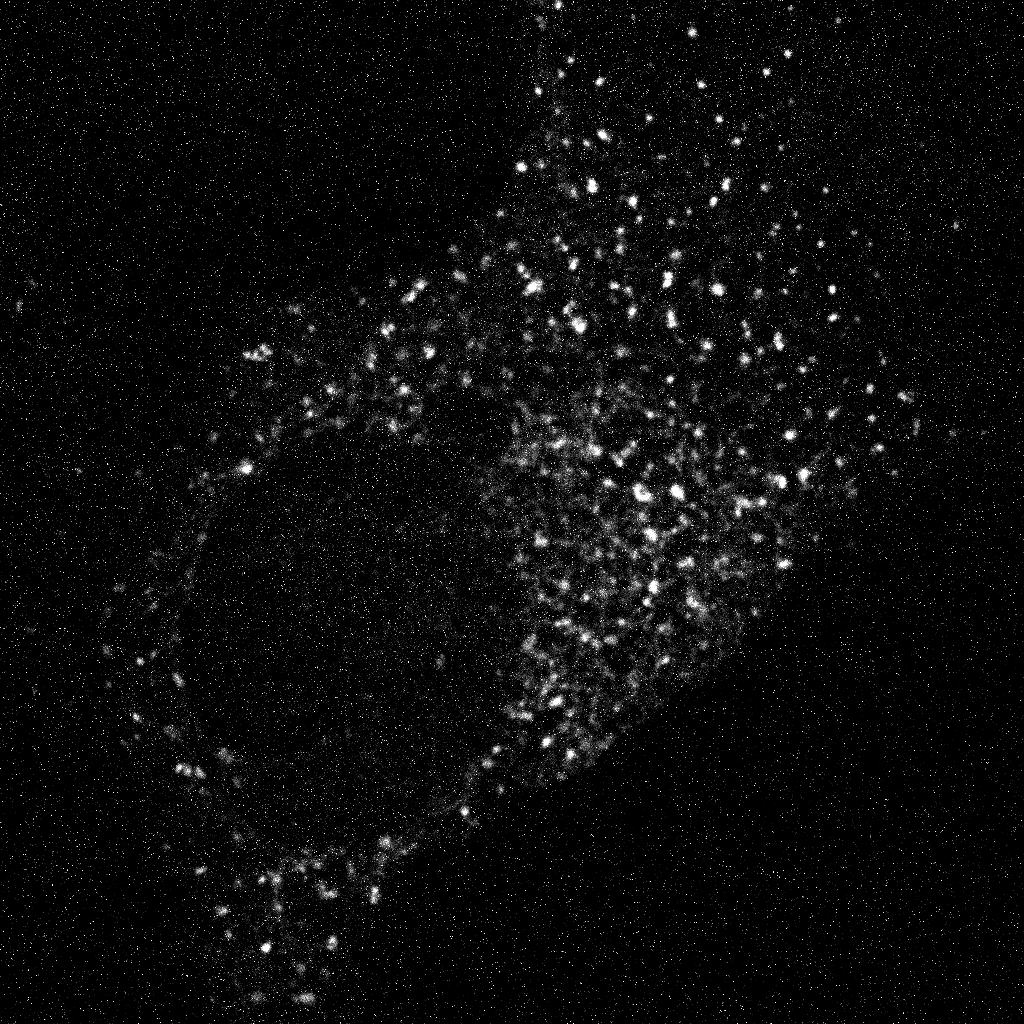

Supplement: Supplementary file 11 — Figure EV2 Source Data [file 44318_2026_754_MOESM11_ESM.zip › EV Figure2/EV 2H/EV2H_image_SEC16B OE_RFP-SEC13.tif]

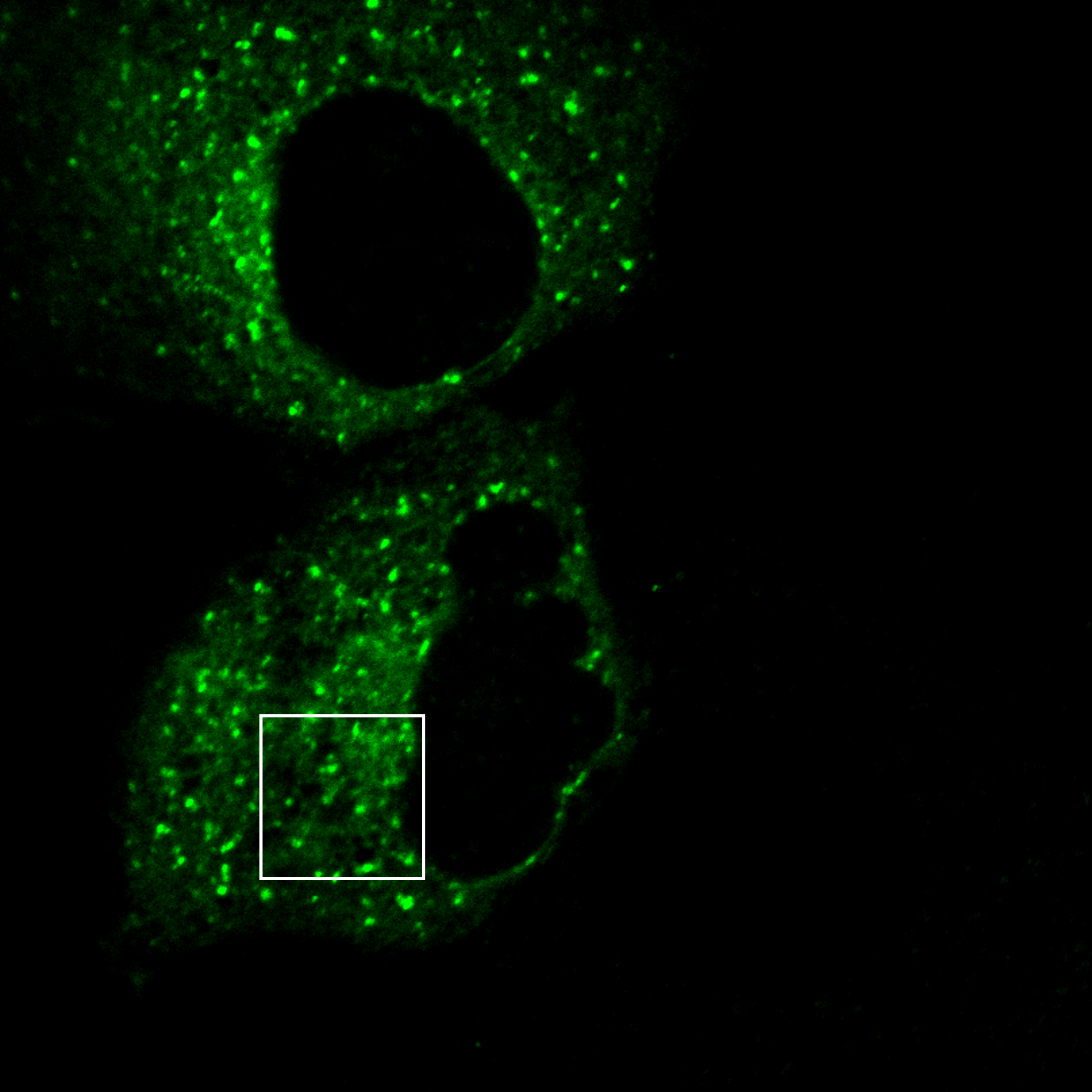

Supplement: Supplementary file 11 — Figure EV2 Source Data [file 44318_2026_754_MOESM11_ESM.zip › EV Figure2/EV 2I/EV2I_image_SEC16B OE_GFP-SEC16B_label.tif]

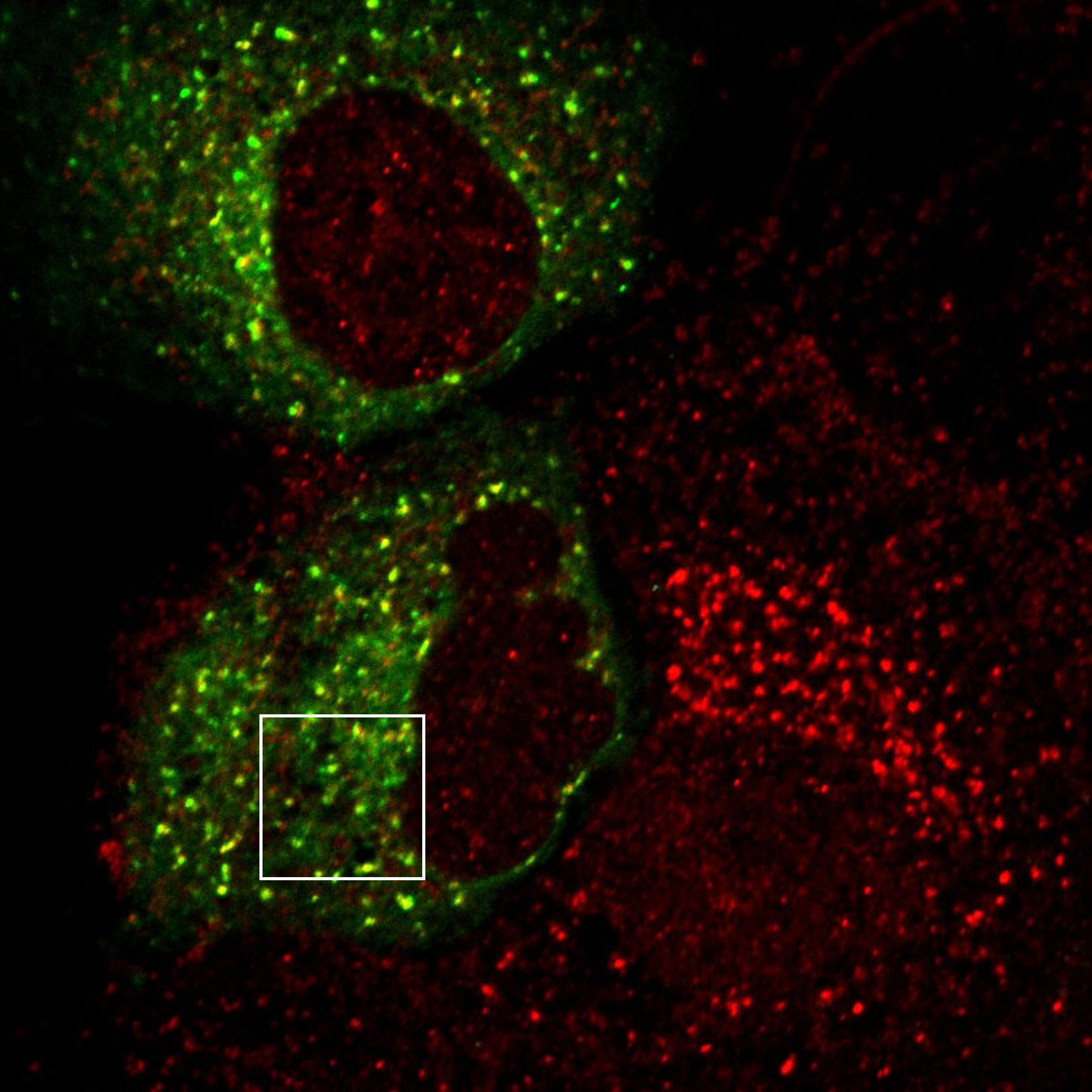

Supplement: Supplementary file 11 — Figure EV2 Source Data [file 44318_2026_754_MOESM11_ESM.zip › EV Figure2/EV 2I/EV2I_image_SEC16B OE_Merge_label.tif]

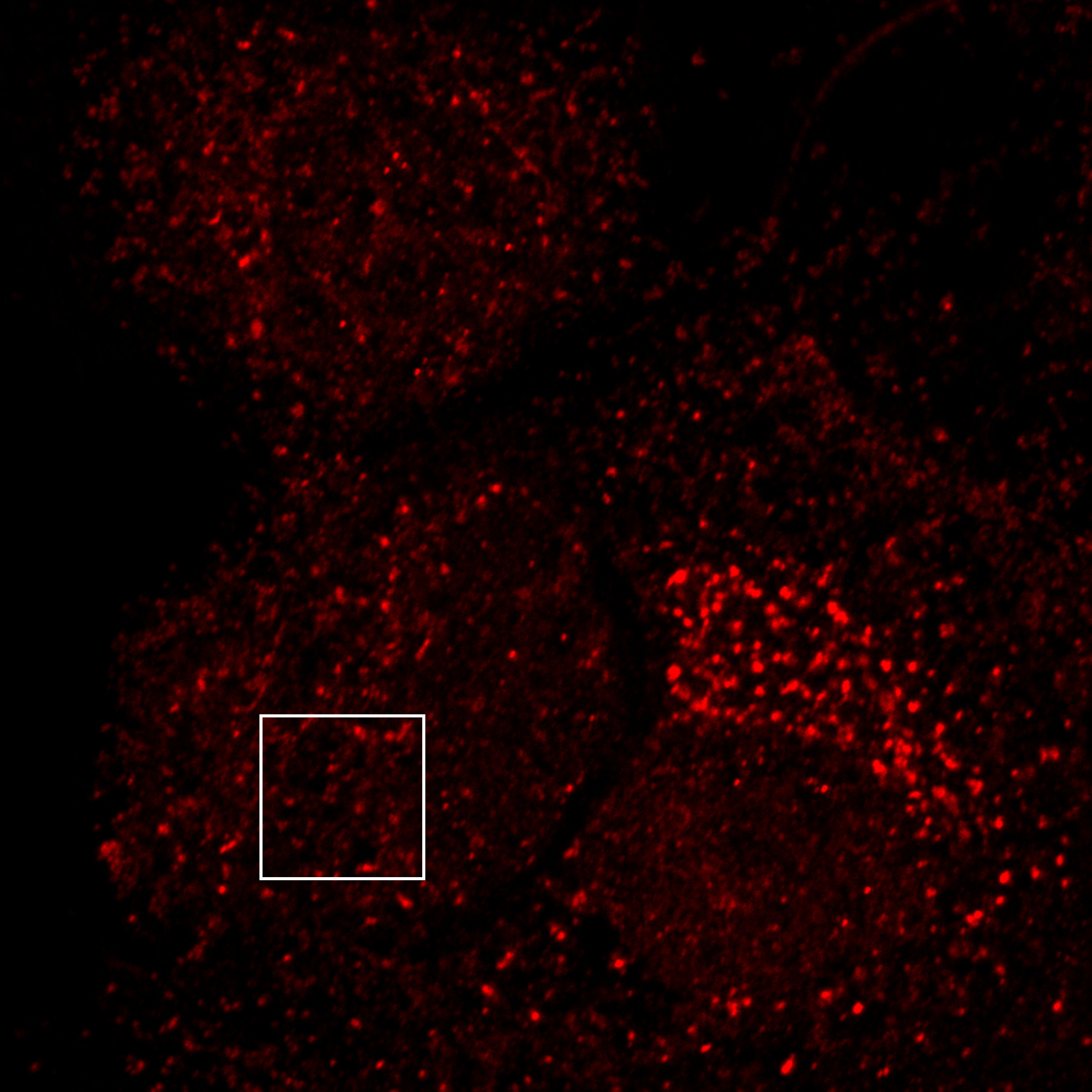

Supplement: Supplementary file 11 — Figure EV2 Source Data [file 44318_2026_754_MOESM11_ESM.zip › EV Figure2/EV 2I/EV2I_image_SEC16B OE_SEC24A_label.tif]

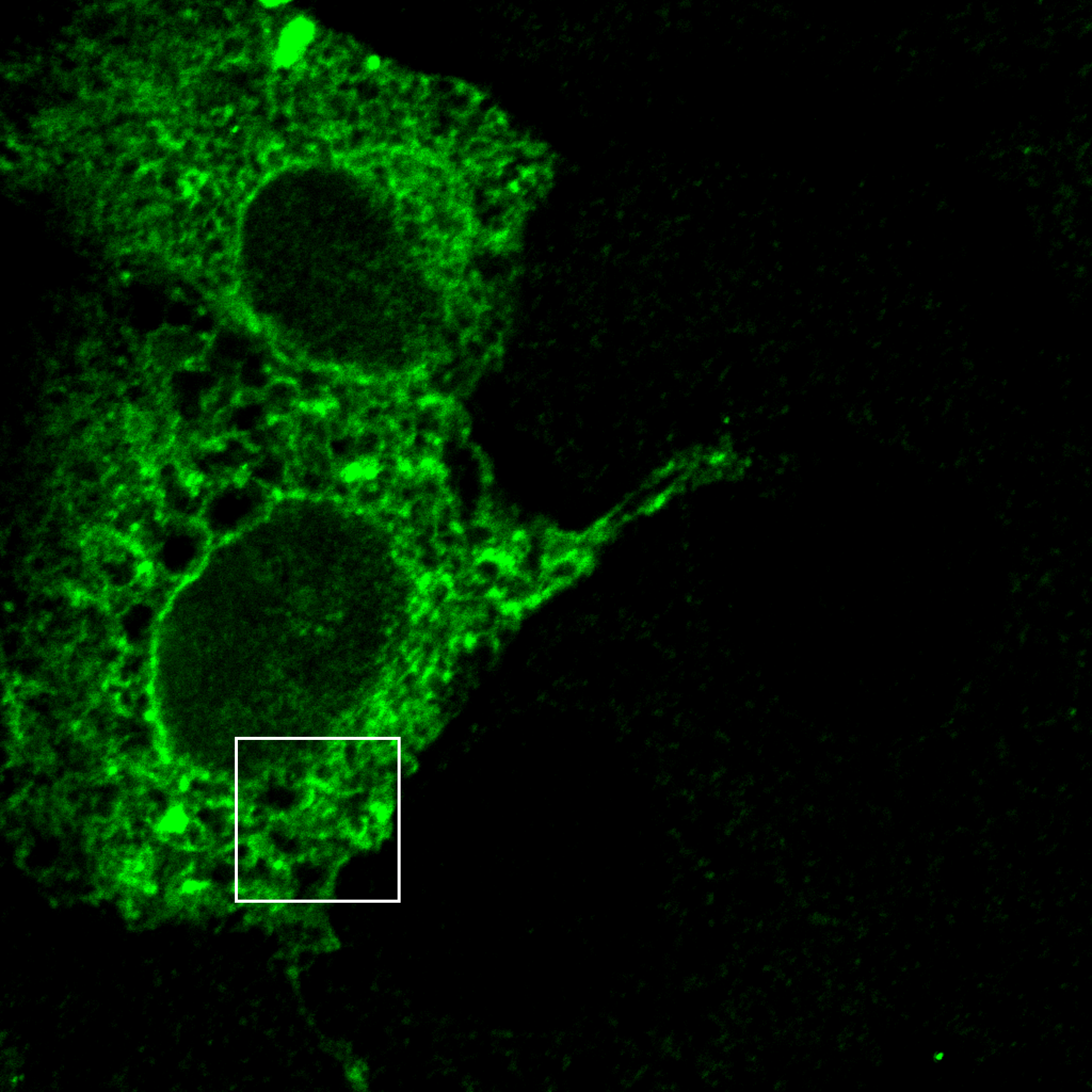

Supplement: Supplementary file 11 — Figure EV2 Source Data [file 44318_2026_754_MOESM11_ESM.zip › EV Figure2/EV 2I/EV2I_image_SEC61B OE_GFP-SEC61B_label.tif]

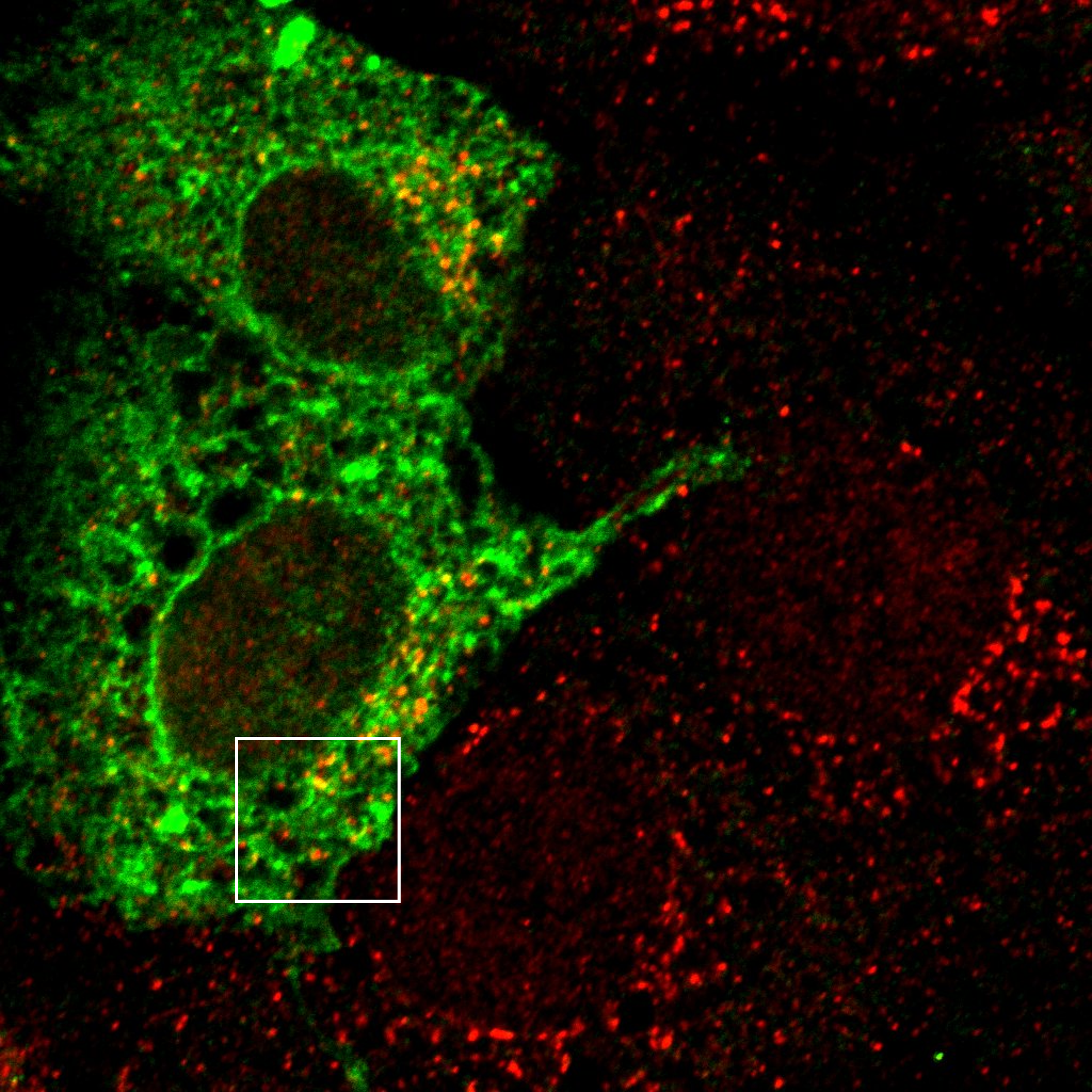

Supplement: Supplementary file 11 — Figure EV2 Source Data [file 44318_2026_754_MOESM11_ESM.zip › EV Figure2/EV 2I/EV2I_image_SEC61B OE_Merge_label.tif]

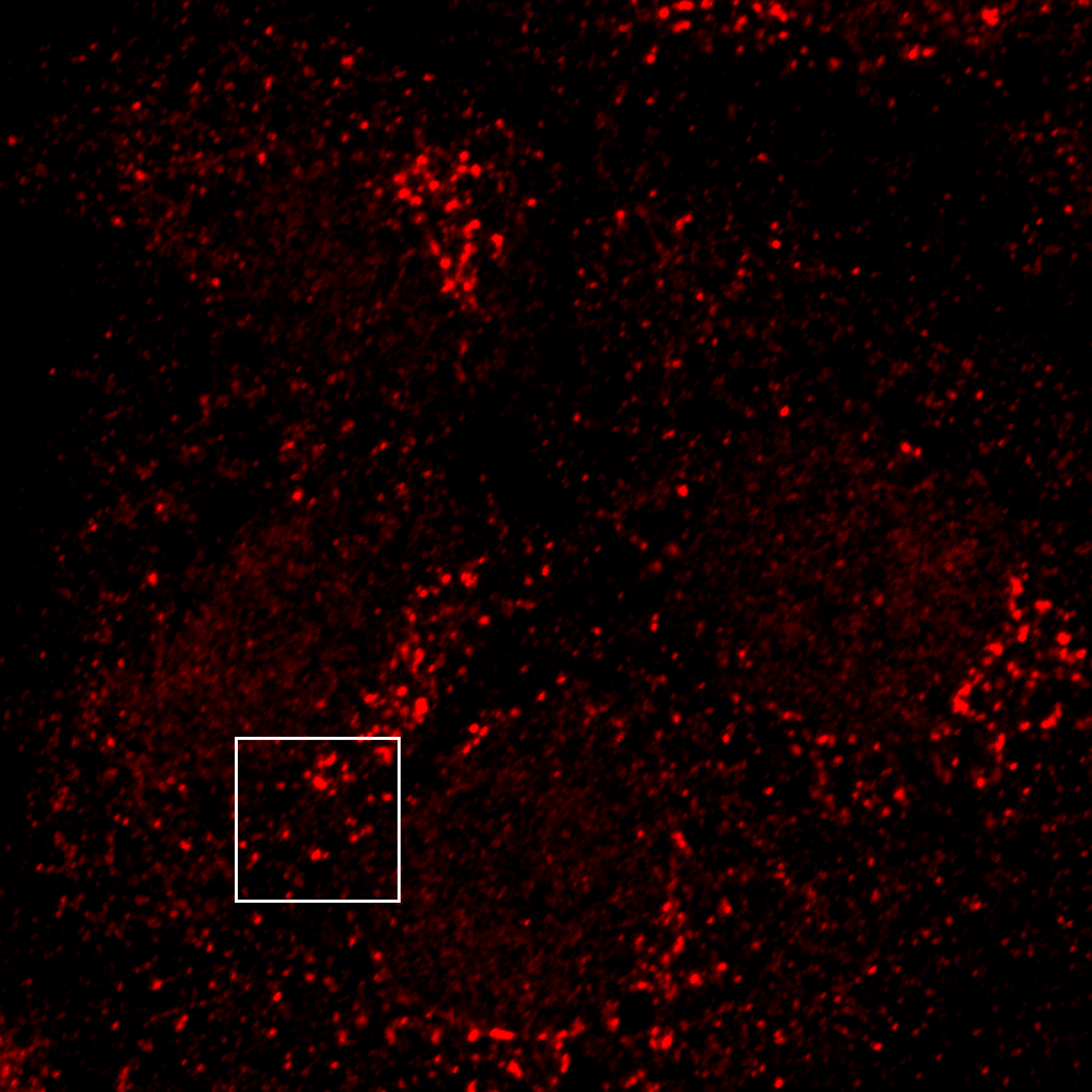

Supplement: Supplementary file 11 — Figure EV2 Source Data [file 44318_2026_754_MOESM11_ESM.zip › EV Figure2/EV 2I/EV2I_image_SEC61B OE_SEC24A_label.tif]

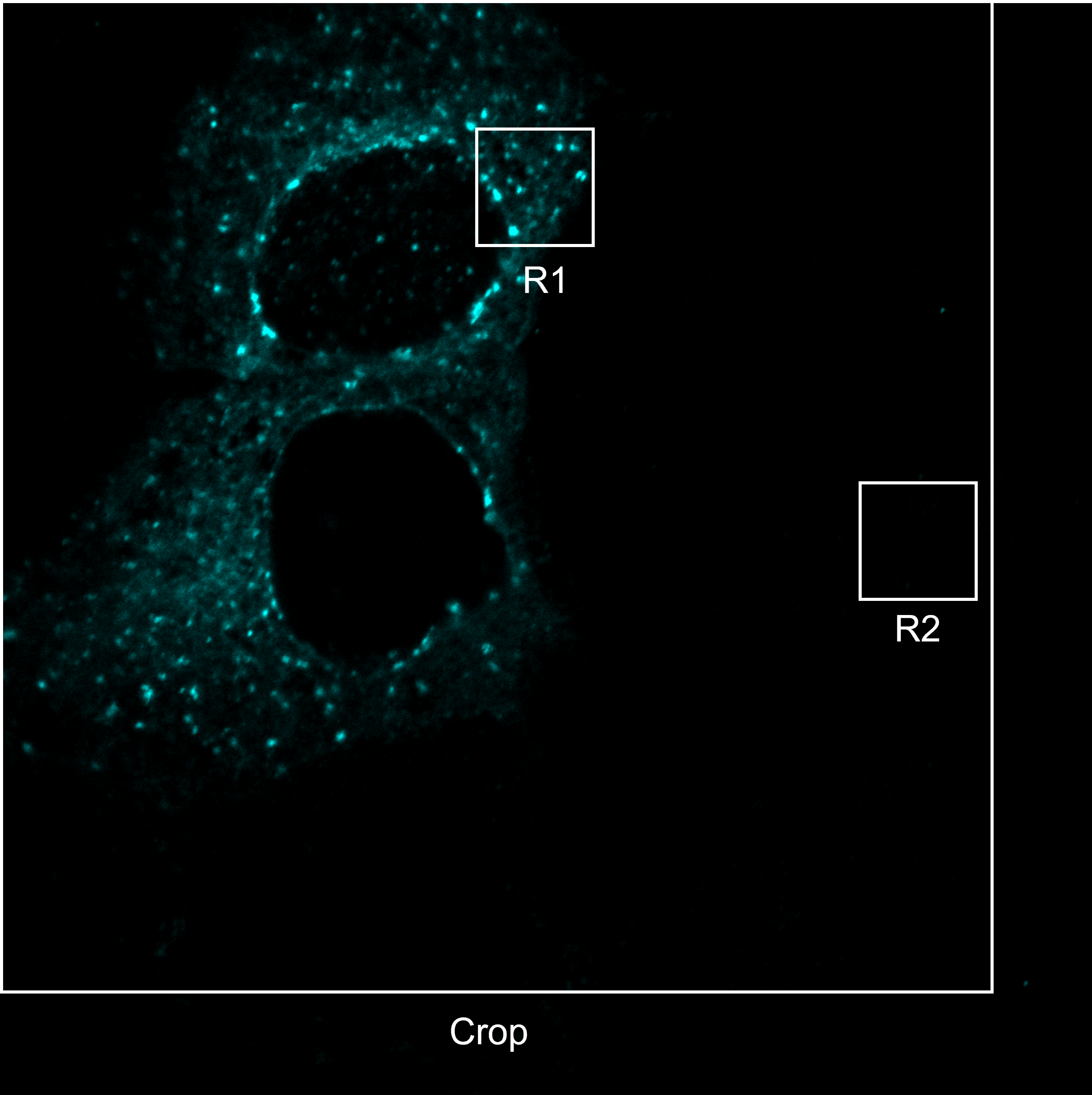

Supplement: Supplementary file 11 — Figure EV2 Source Data [file 44318_2026_754_MOESM11_ESM.zip › EV Figure2/EV 2J/EV2J_image_GFP-SEC16B_label.tif]

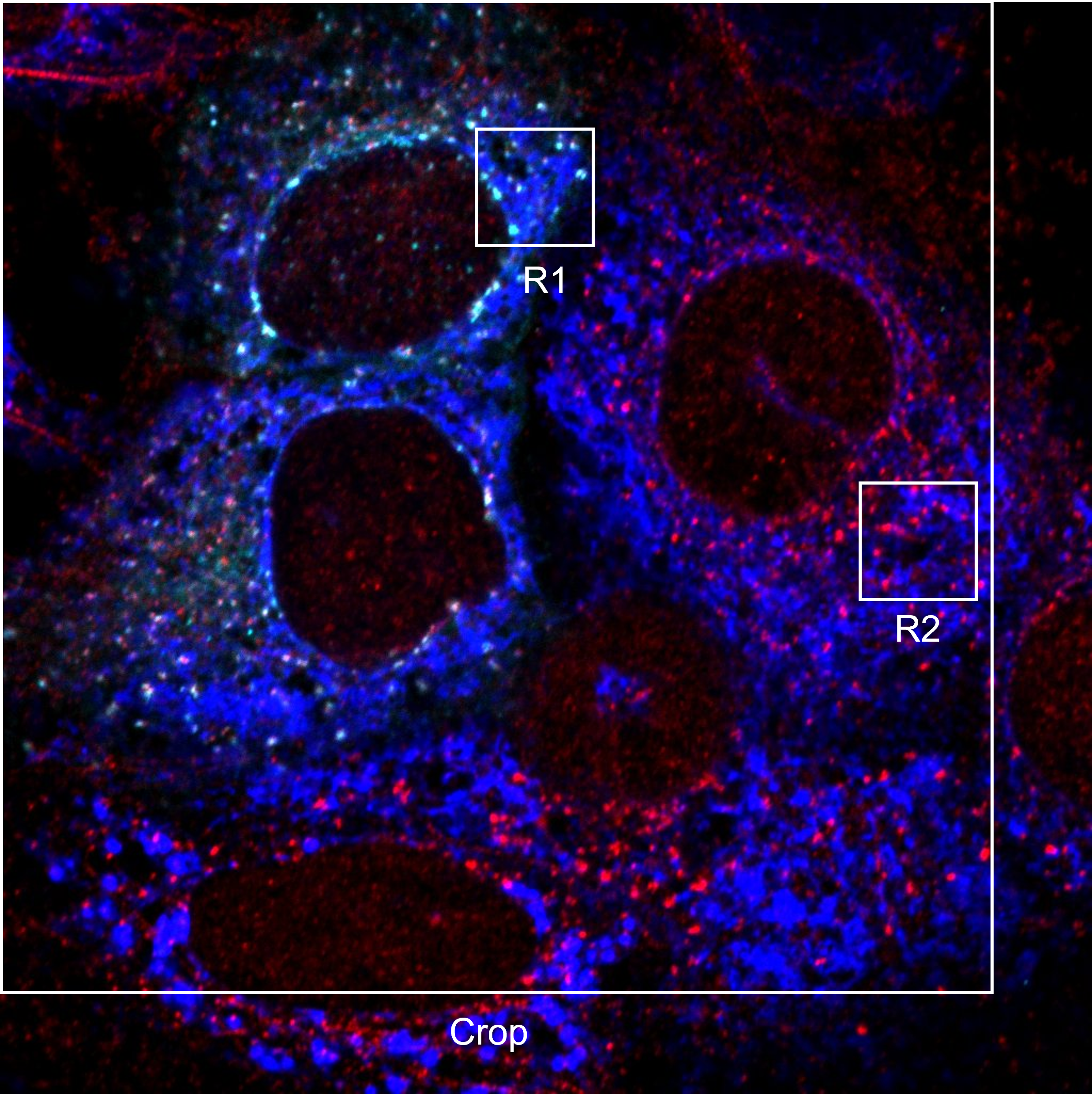

Supplement: Supplementary file 11 — Figure EV2 Source Data [file 44318_2026_754_MOESM11_ESM.zip › EV Figure2/EV 2J/EV2J_image_Merge_label.tif]

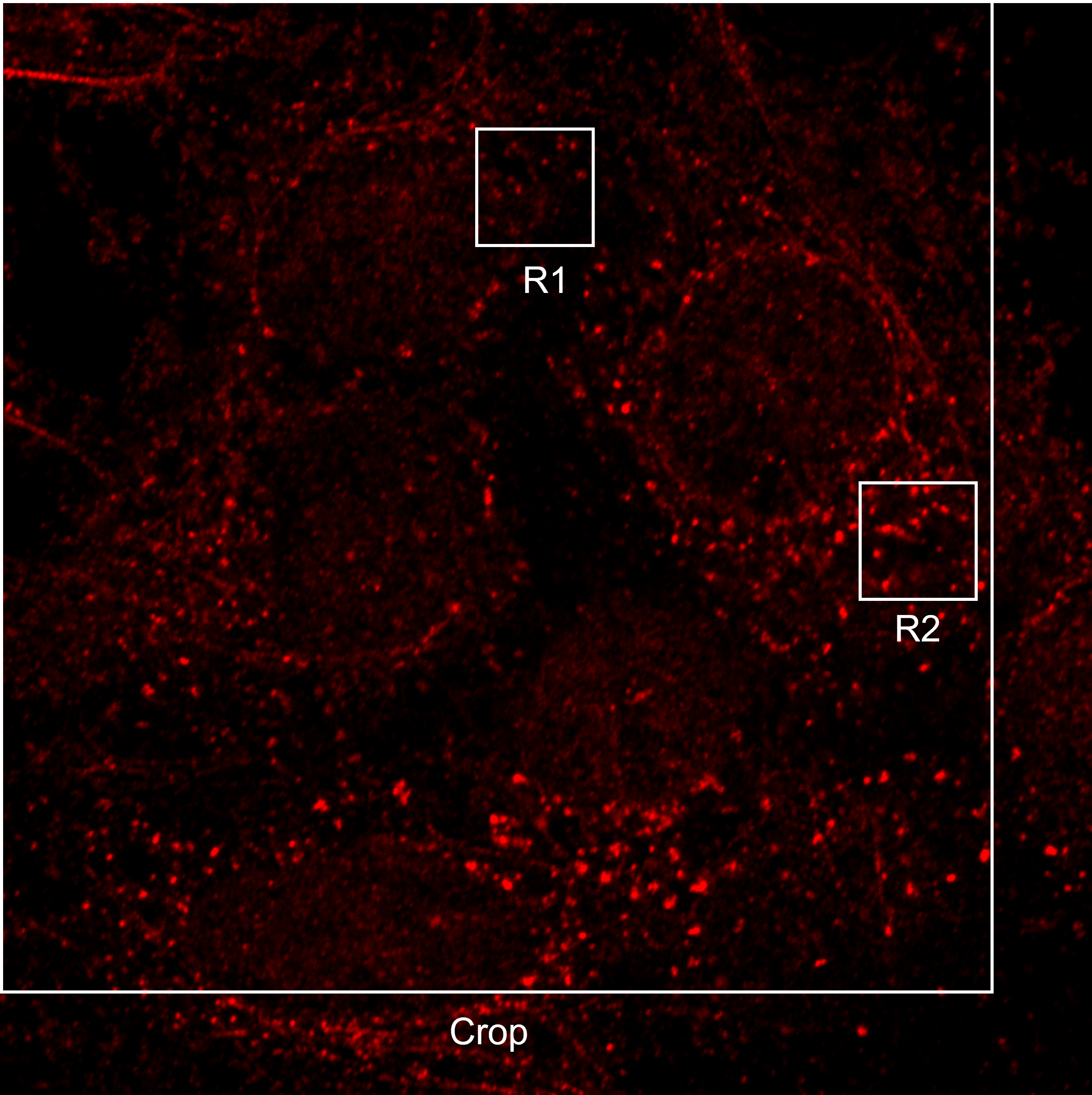

Supplement: Supplementary file 11 — Figure EV2 Source Data [file 44318_2026_754_MOESM11_ESM.zip › EV Figure2/EV 2J/EV2J_image_SEC24A_label.tif]

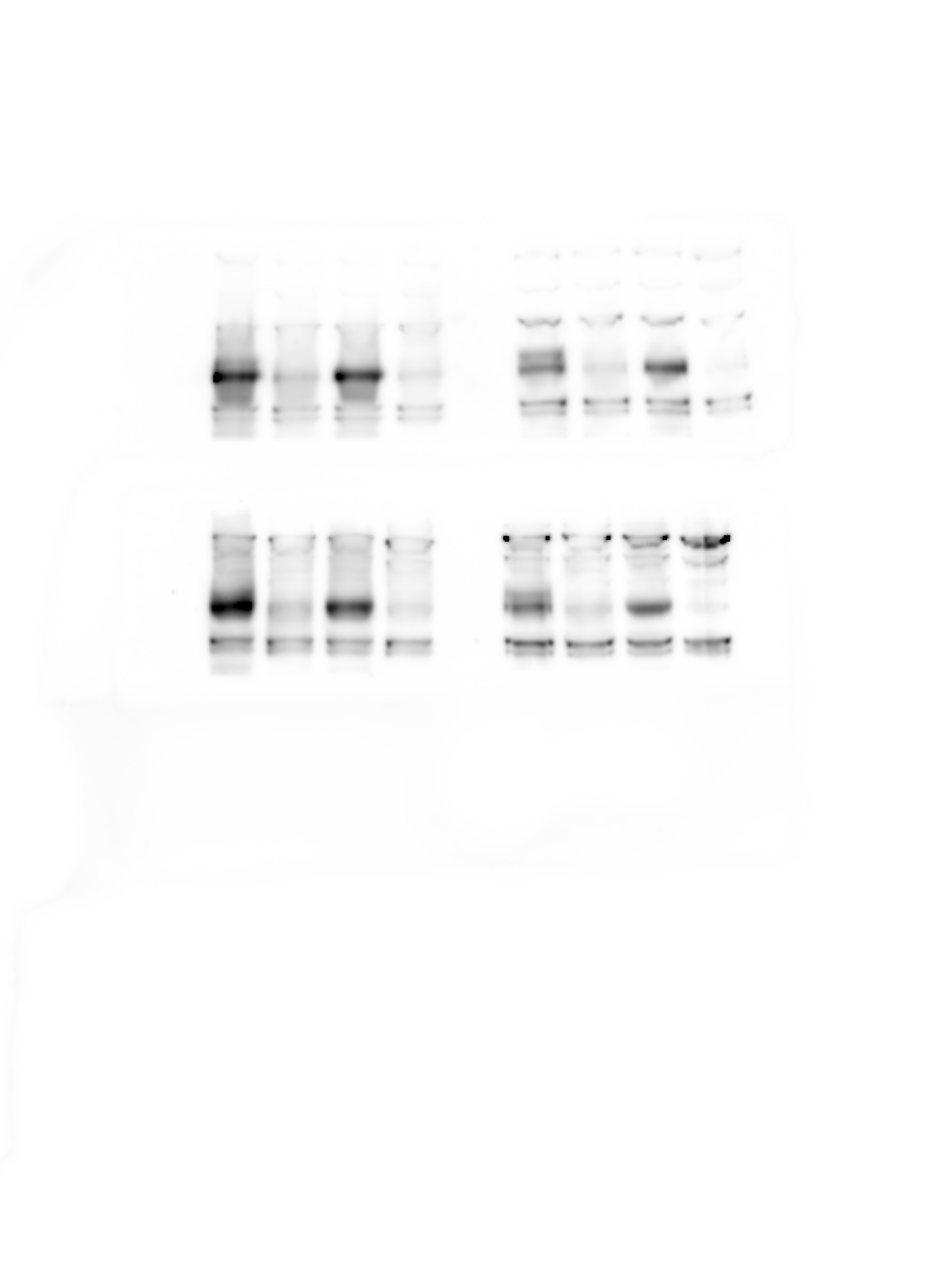

Supplement: Supplementary file 12 — Figure EV3 Source Data [file 44318_2026_754_MOESM12_ESM.zip › EV Figure3/EV 3A/EV3A_western_SEC16B.tif]

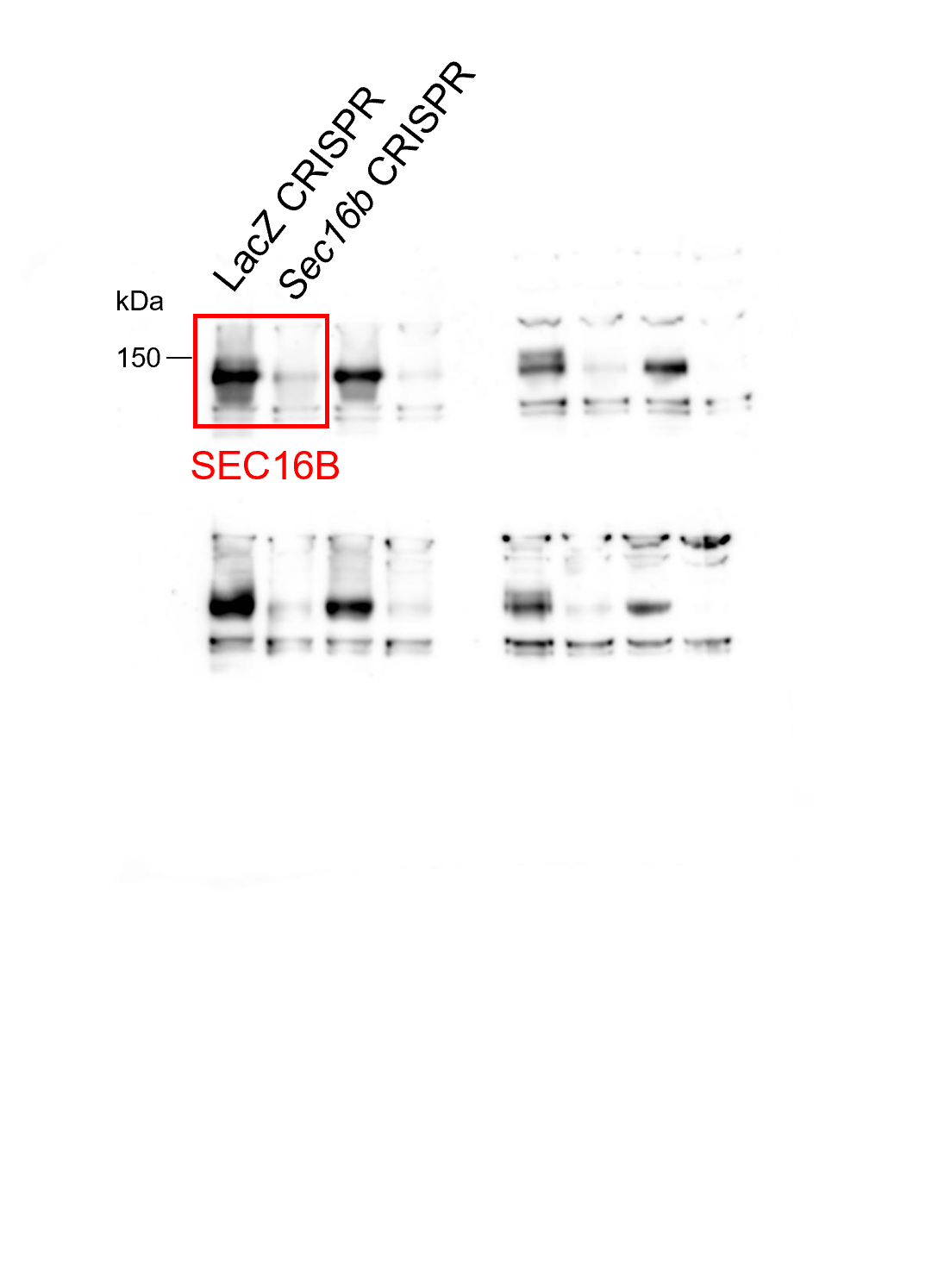

Supplement: Supplementary file 12 — Figure EV3 Source Data [file 44318_2026_754_MOESM12_ESM.zip › EV Figure3/EV 3A/EV3A_western_SEC16B_label.tif]

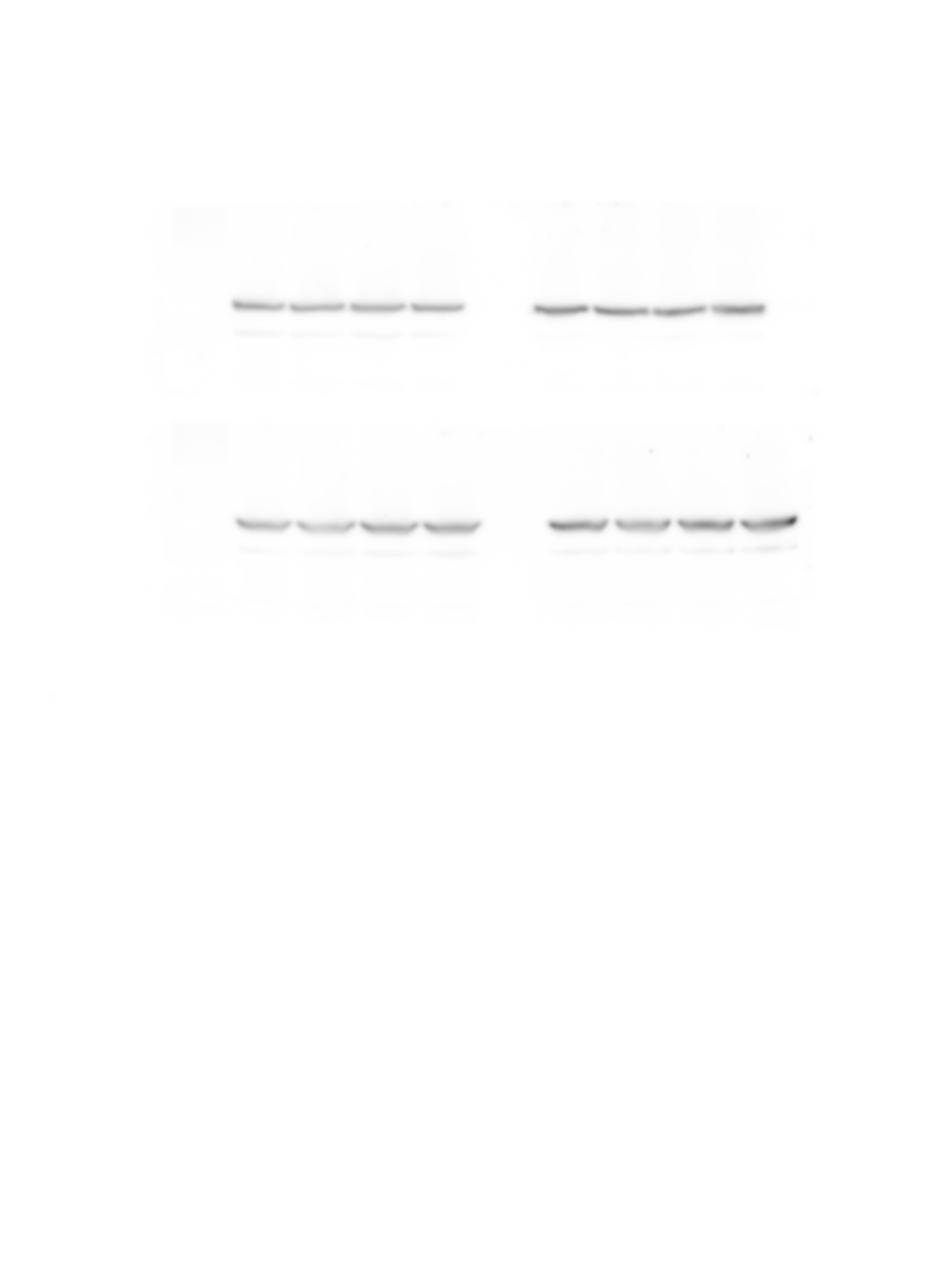

Supplement: Supplementary file 12 — Figure EV3 Source Data [file 44318_2026_754_MOESM12_ESM.zip › EV Figure3/EV 3A/EV3A_western_Tub.tif]

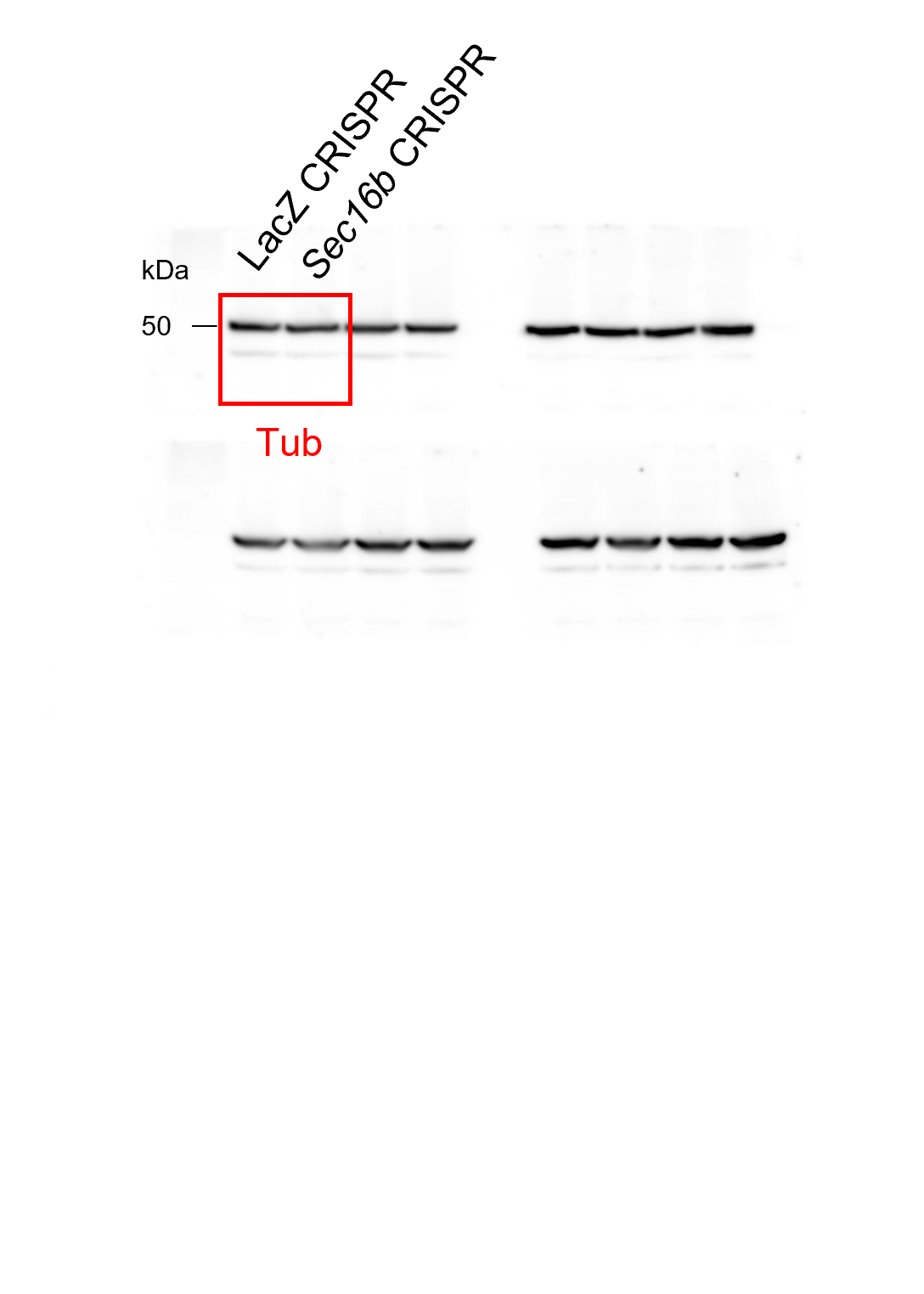

Supplement: Supplementary file 12 — Figure EV3 Source Data [file 44318_2026_754_MOESM12_ESM.zip › EV Figure3/EV 3A/EV3A_western_Tub_label.tif]

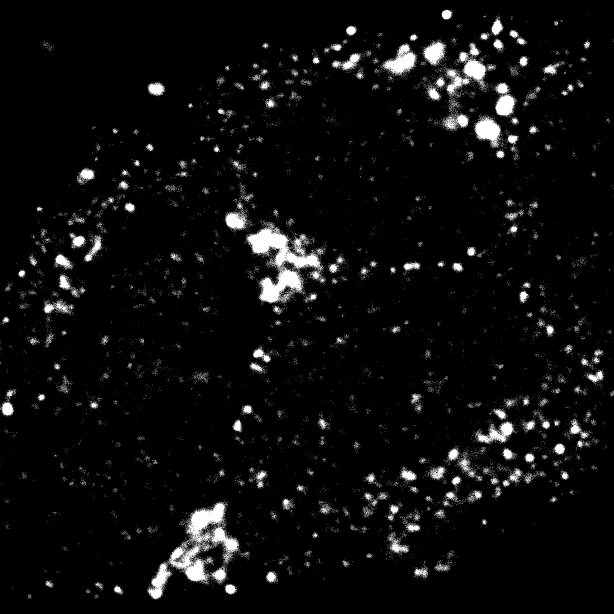

Supplement: Supplementary file 12 — Figure EV3 Source Data [file 44318_2026_754_MOESM12_ESM.zip › EV Figure3/EV 3B/EV3B_image_KO_SEC24A.tif]

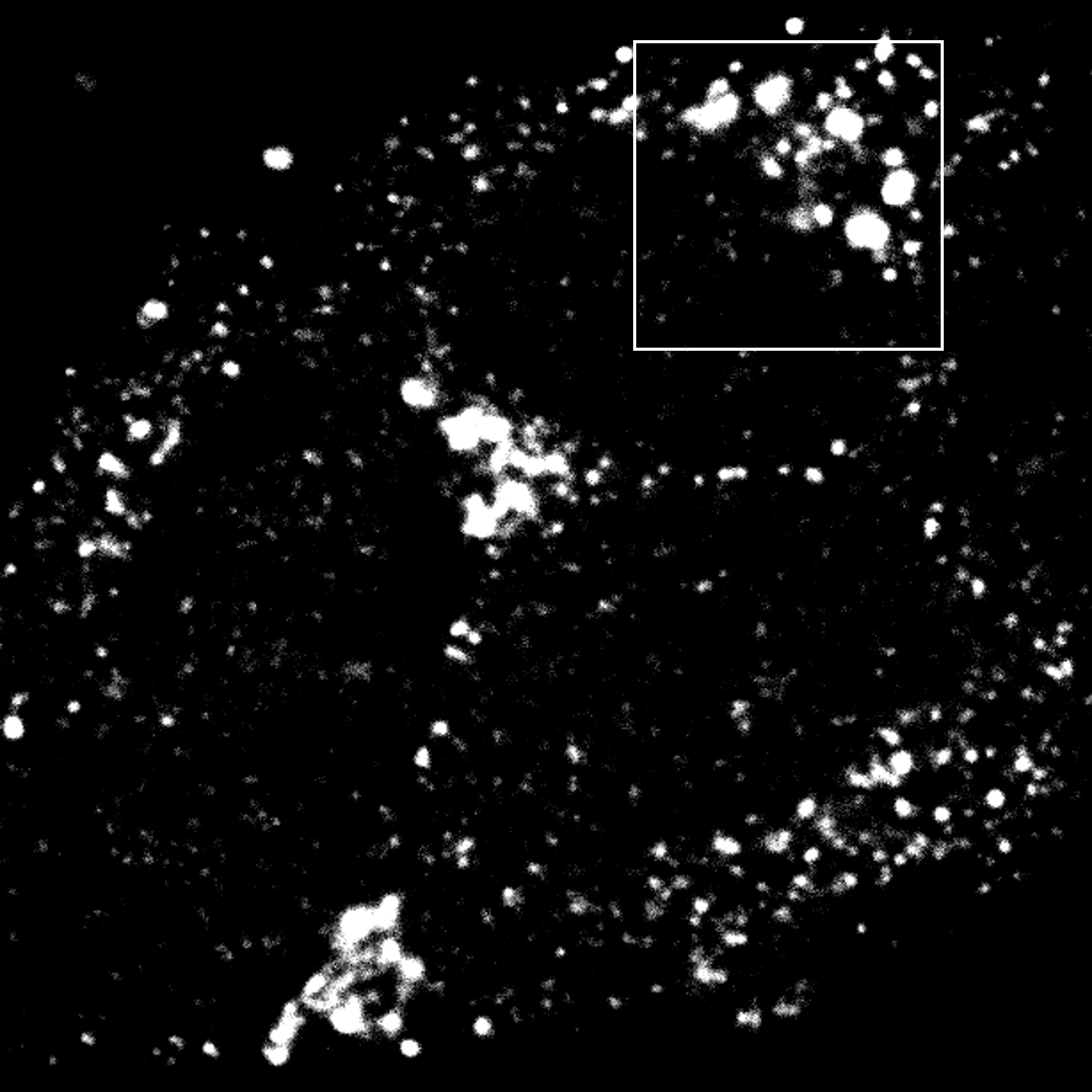

Supplement: Supplementary file 12 — Figure EV3 Source Data [file 44318_2026_754_MOESM12_ESM.zip › EV Figure3/EV 3B/EV3B_image_KO_SEC24A_label.tif]

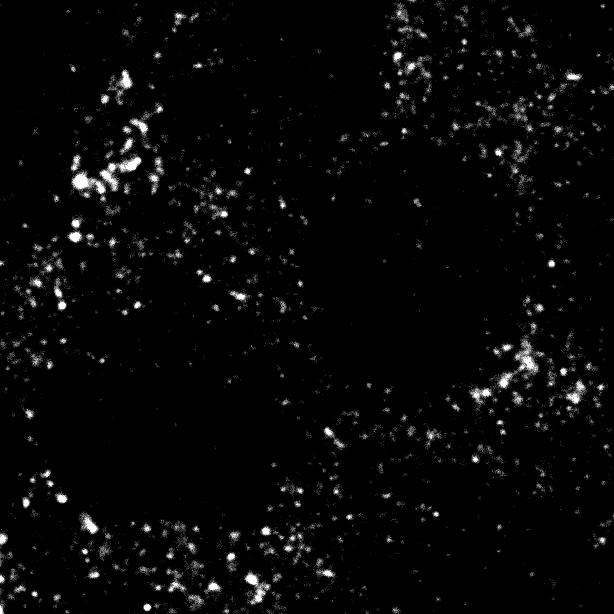

Supplement: Supplementary file 12 — Figure EV3 Source Data [file 44318_2026_754_MOESM12_ESM.zip › EV Figure3/EV 3B/EV3B_image_LacZ_SEC24A.tif]

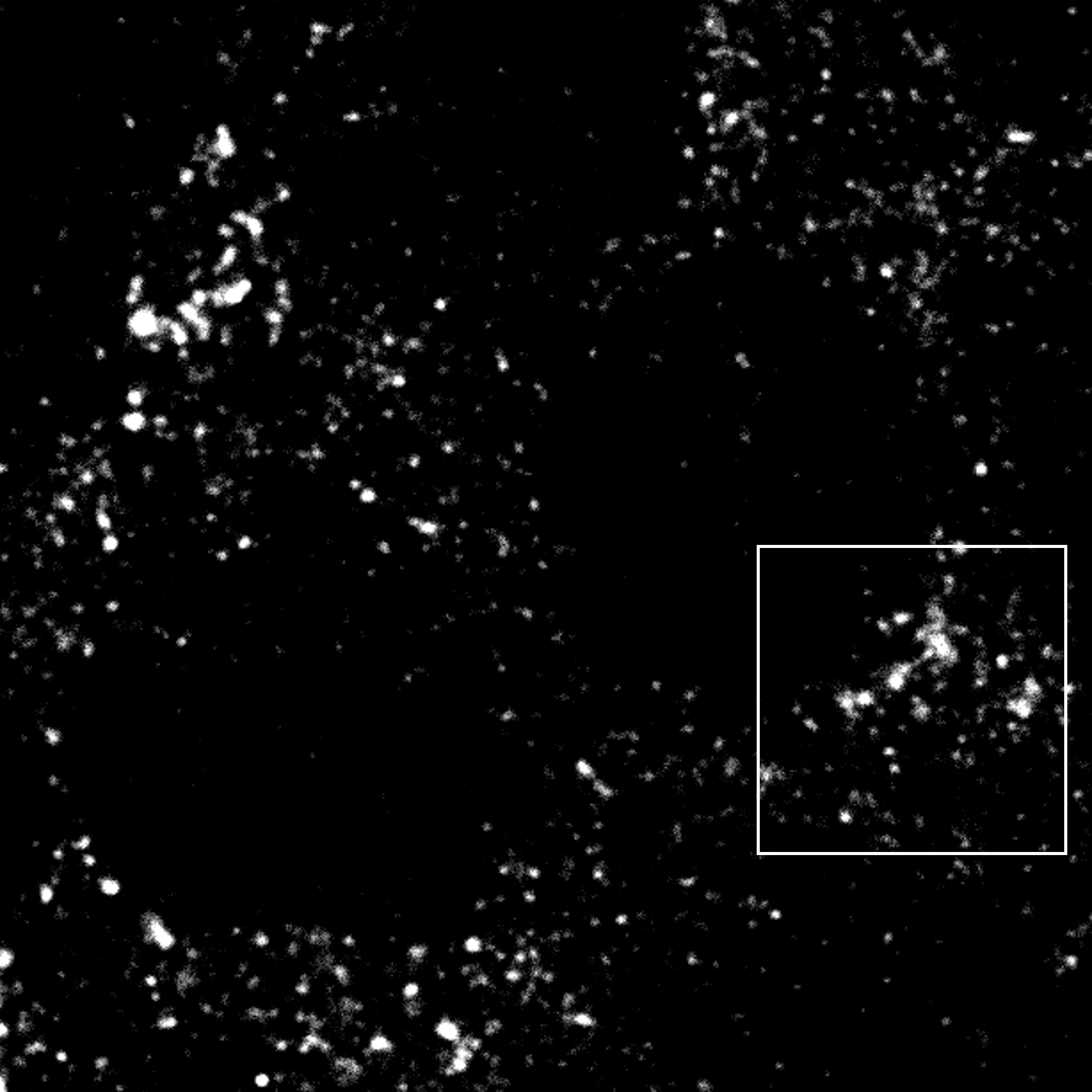

Supplement: Supplementary file 12 — Figure EV3 Source Data [file 44318_2026_754_MOESM12_ESM.zip › EV Figure3/EV 3B/EV3B_image_LacZ_SEC24A_label.tif]

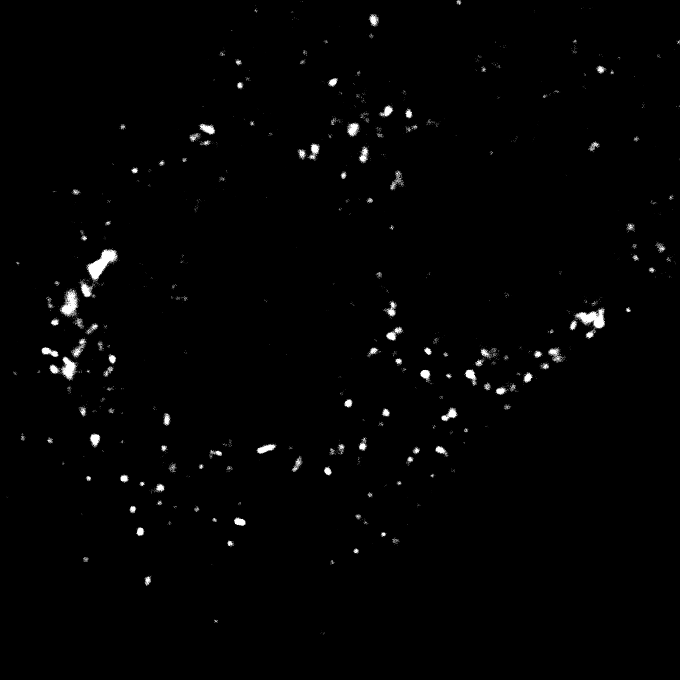

Supplement: Supplementary file 12 — Figure EV3 Source Data [file 44318_2026_754_MOESM12_ESM.zip › EV Figure3/EV 3C/EV3C_image_KO_SEC31A.tif]

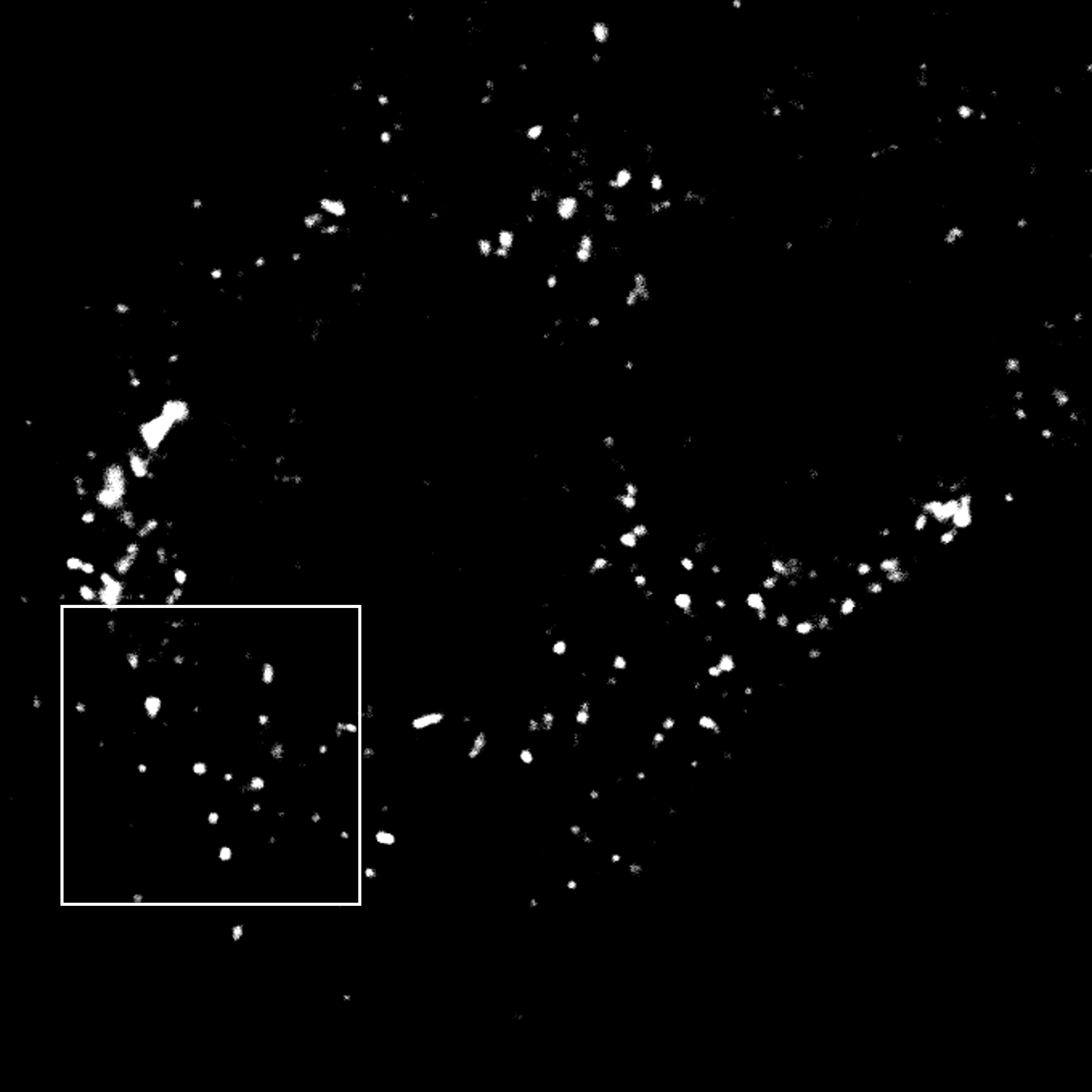

Supplement: Supplementary file 12 — Figure EV3 Source Data [file 44318_2026_754_MOESM12_ESM.zip › EV Figure3/EV 3C/EV3C_image_KO_SEC31A_label.tif]

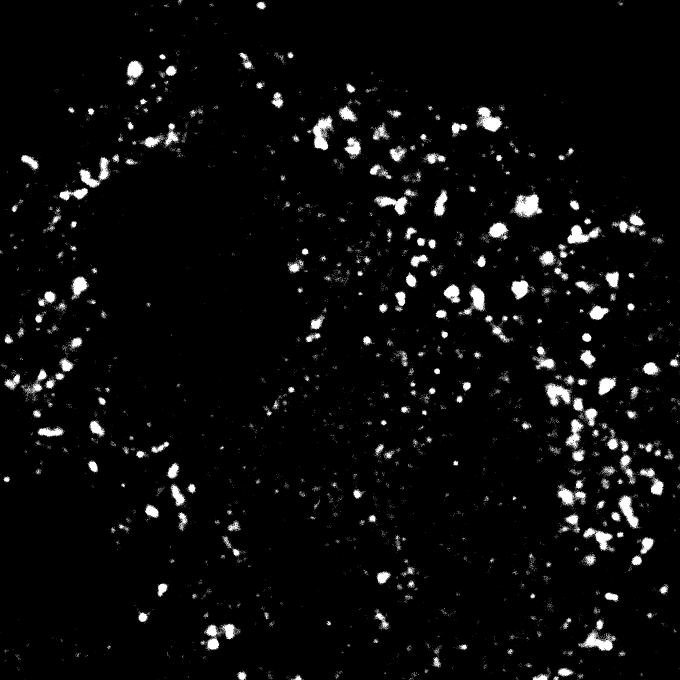

Supplement: Supplementary file 12 — Figure EV3 Source Data [file 44318_2026_754_MOESM12_ESM.zip › EV Figure3/EV 3C/EV3C_image_Lacz_SEC31A.tif]

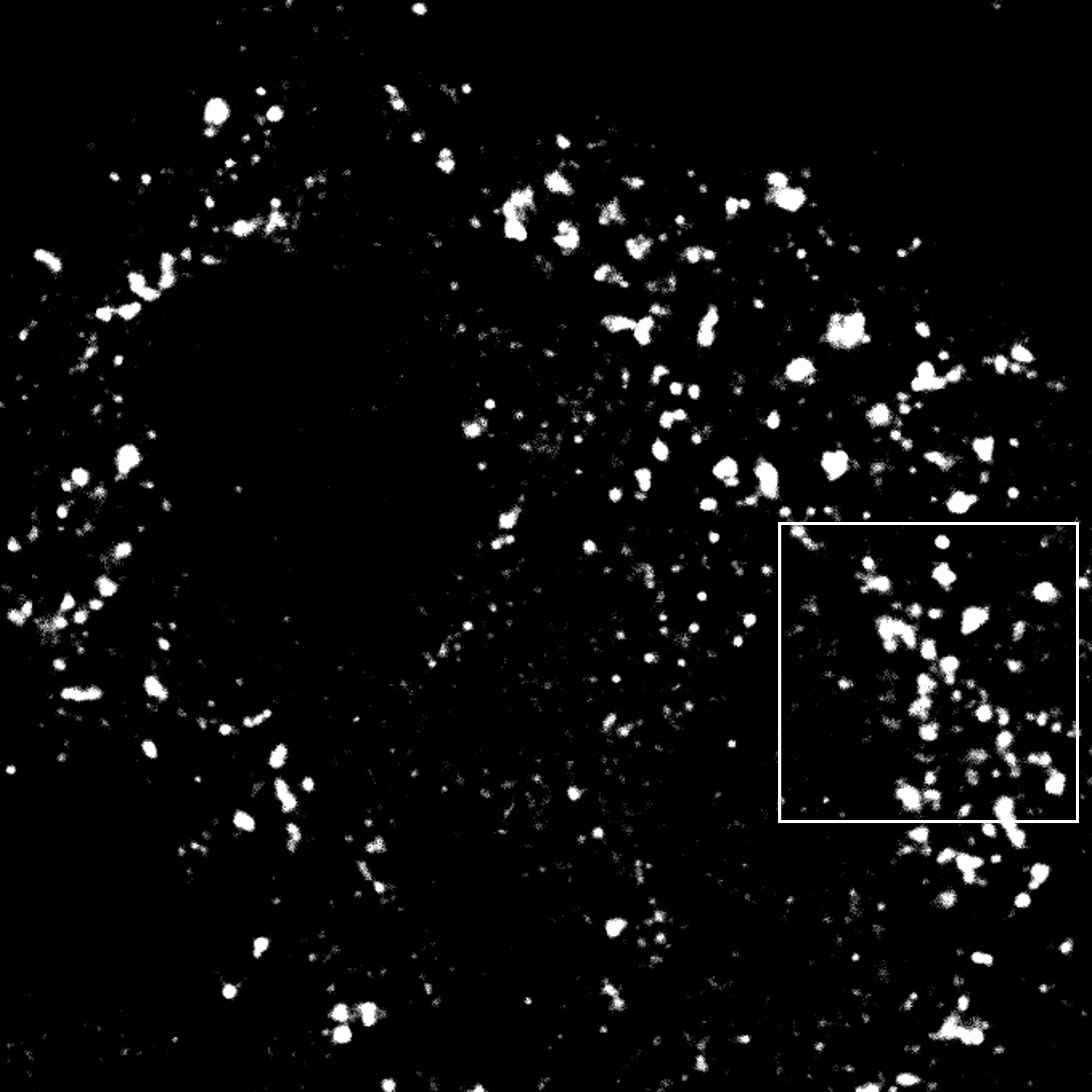

Supplement: Supplementary file 12 — Figure EV3 Source Data [file 44318_2026_754_MOESM12_ESM.zip › EV Figure3/EV 3C/EV3C_image_Lacz_SEC31A_label.tif]

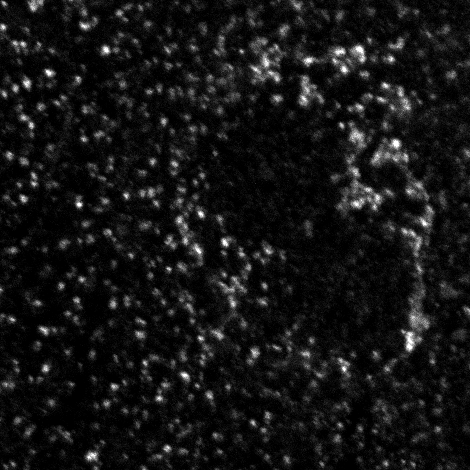

Supplement: Supplementary file 12 — Figure EV3 Source Data [file 44318_2026_754_MOESM12_ESM.zip › EV Figure3/EV 3D/EV3D_image_KO_0 μM.tif]

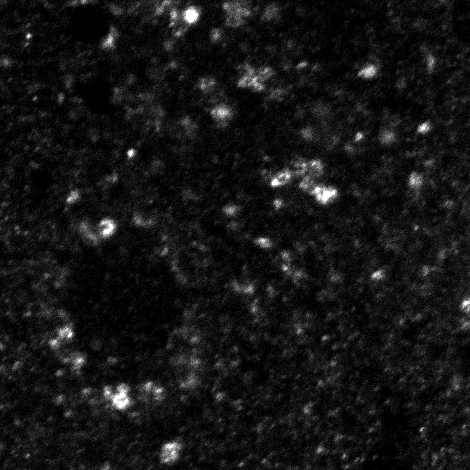

Supplement: Supplementary file 12 — Figure EV3 Source Data [file 44318_2026_754_MOESM12_ESM.zip › EV Figure3/EV 3D/EV3D_image_KO_100 μM.tif]

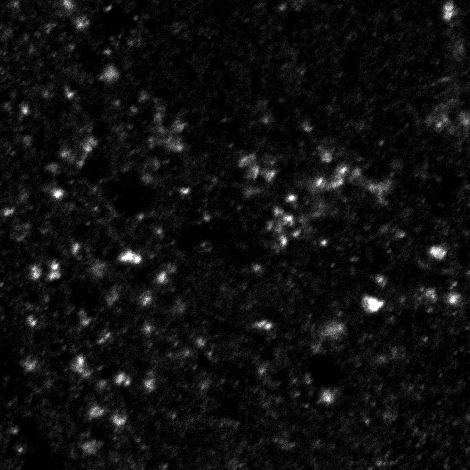

Supplement: Supplementary file 12 — Figure EV3 Source Data [file 44318_2026_754_MOESM12_ESM.zip › EV Figure3/EV 3D/EV3D_image_KO_25 μM.tif]

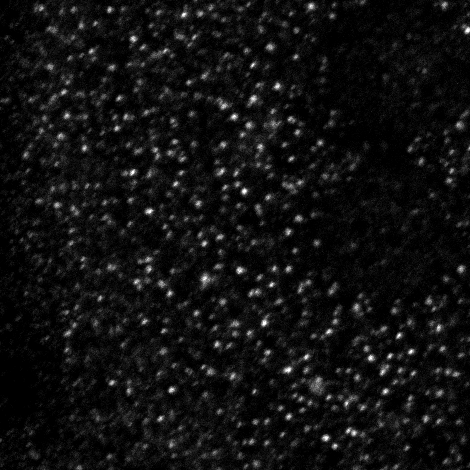

Supplement: Supplementary file 12 — Figure EV3 Source Data [file 44318_2026_754_MOESM12_ESM.zip › EV Figure3/EV 3D/EV3D_image_WT_0 μM.tif]
