# Supplementary figures and images for: Tissue-selective COPII modulator SEC16B aggravates cardiovascular disease by promoting lipid export (part 3 of 3)
Source: EMBO J. 2026 Apr 24;45(11):3731–62. doi: 10.1038/s44318-026-00754-8 (PMC13226660; doi:10.1038/s44318-026-00754-8)

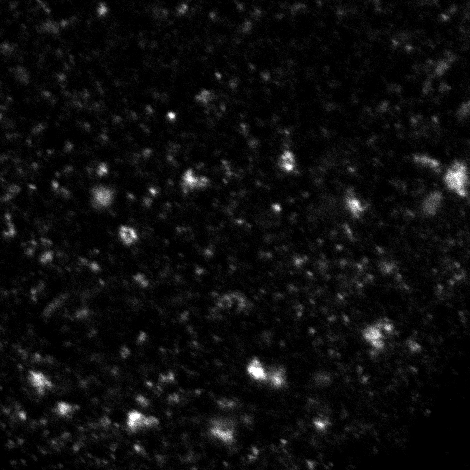

Supplement: Supplementary file 12 — Figure EV3 Source Data [file 44318_2026_754_MOESM12_ESM.zip › EV Figure3/EV 3D/EV3D_image_WT_100 μM.tif]

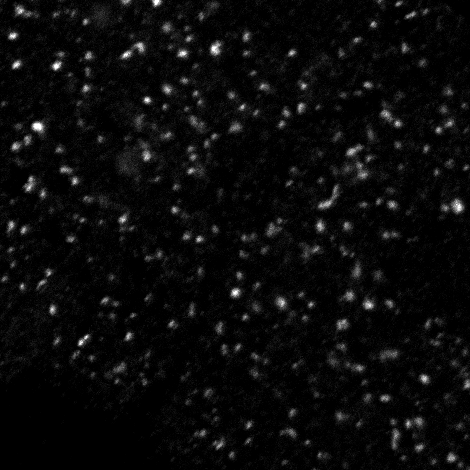

Supplement: Supplementary file 12 — Figure EV3 Source Data [file 44318_2026_754_MOESM12_ESM.zip › EV Figure3/EV 3D/EV3D_image_WT_25 μM.tif]

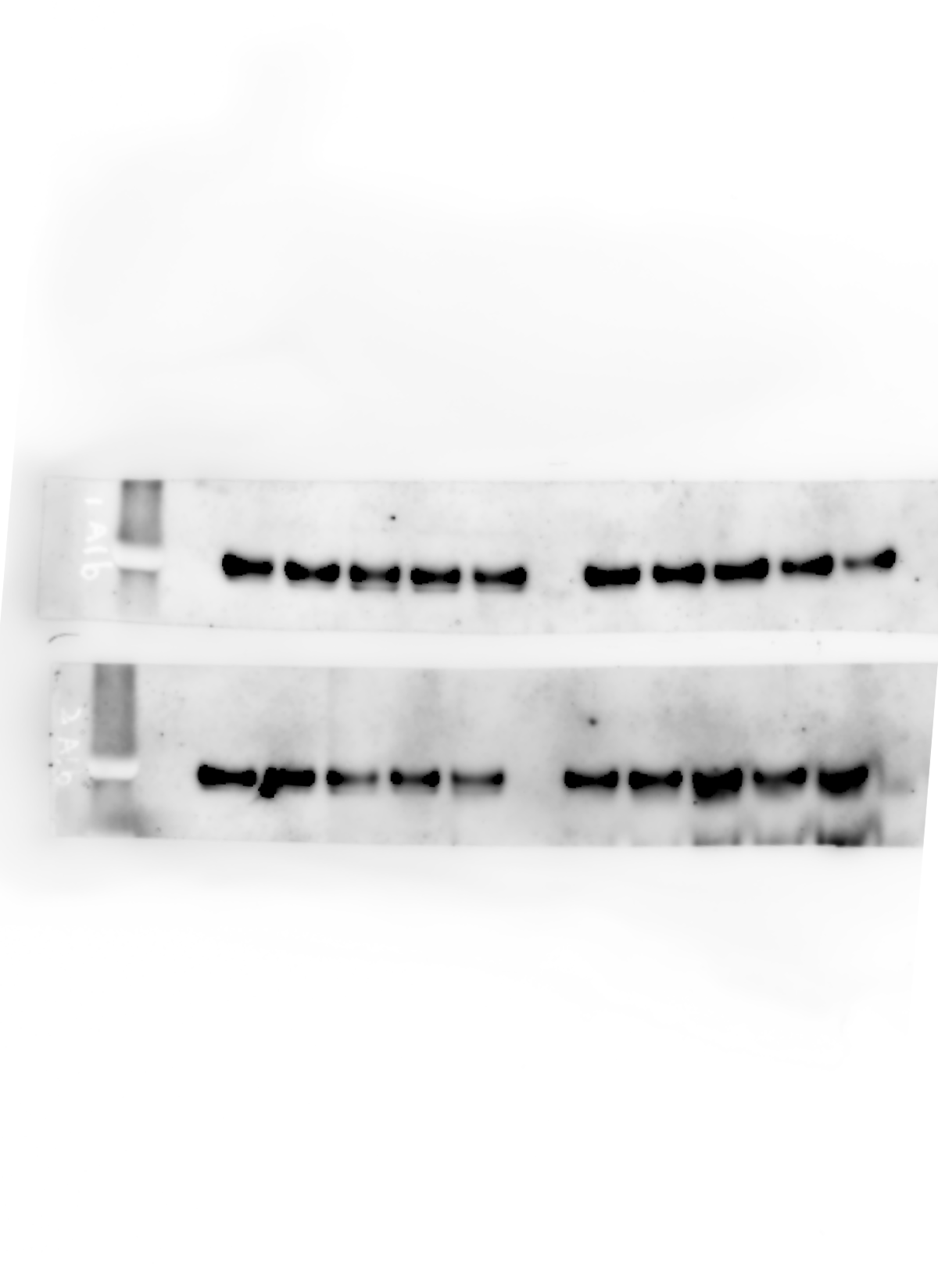

Supplement: Supplementary file 12 — Figure EV3 Source Data [file 44318_2026_754_MOESM12_ESM.zip › EV Figure3/EV 3F/EV3F_western_ALB.tif]

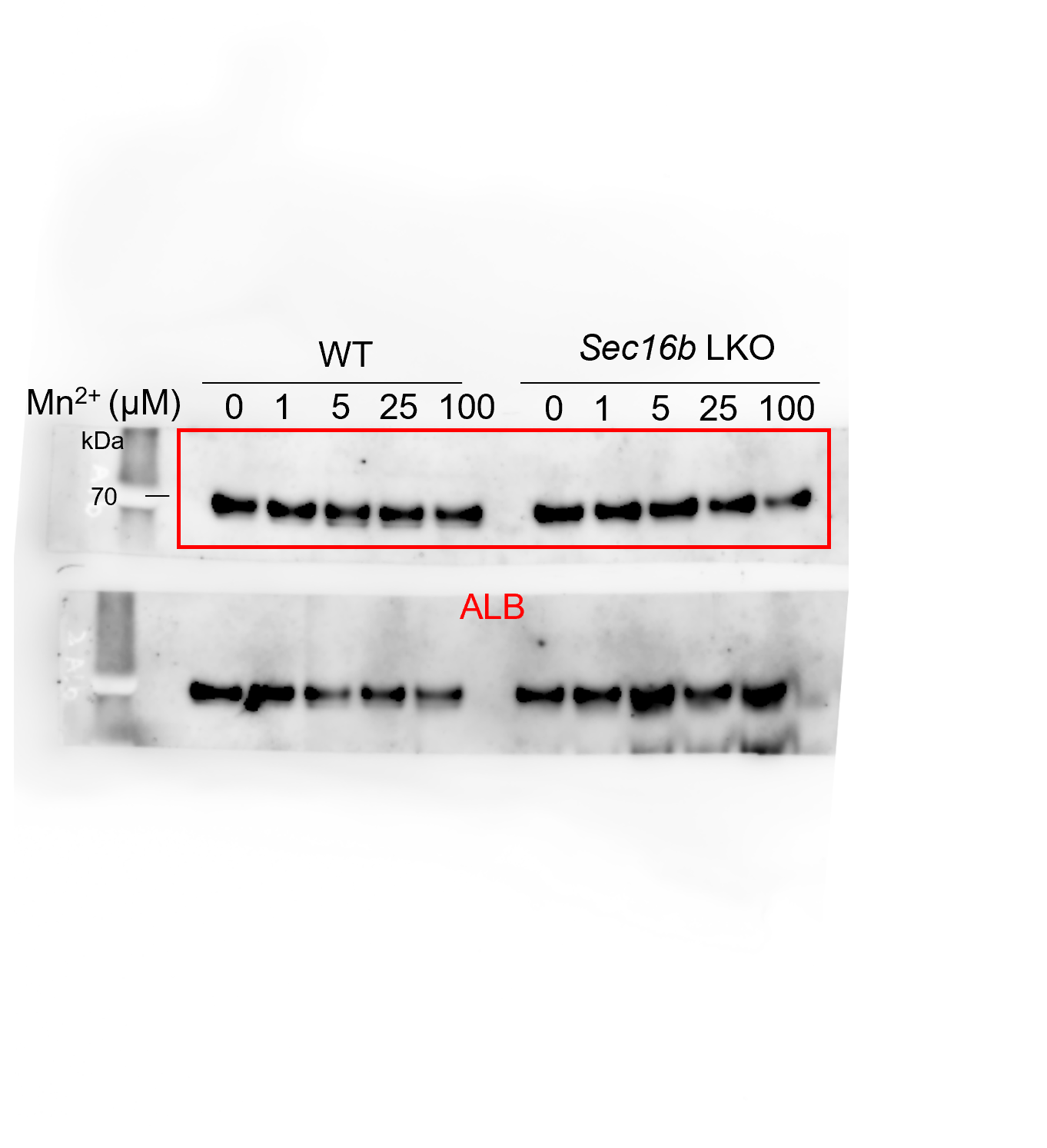

Supplement: Supplementary file 12 — Figure EV3 Source Data [file 44318_2026_754_MOESM12_ESM.zip › EV Figure3/EV 3F/EV3F_western_ALB_label.tif]

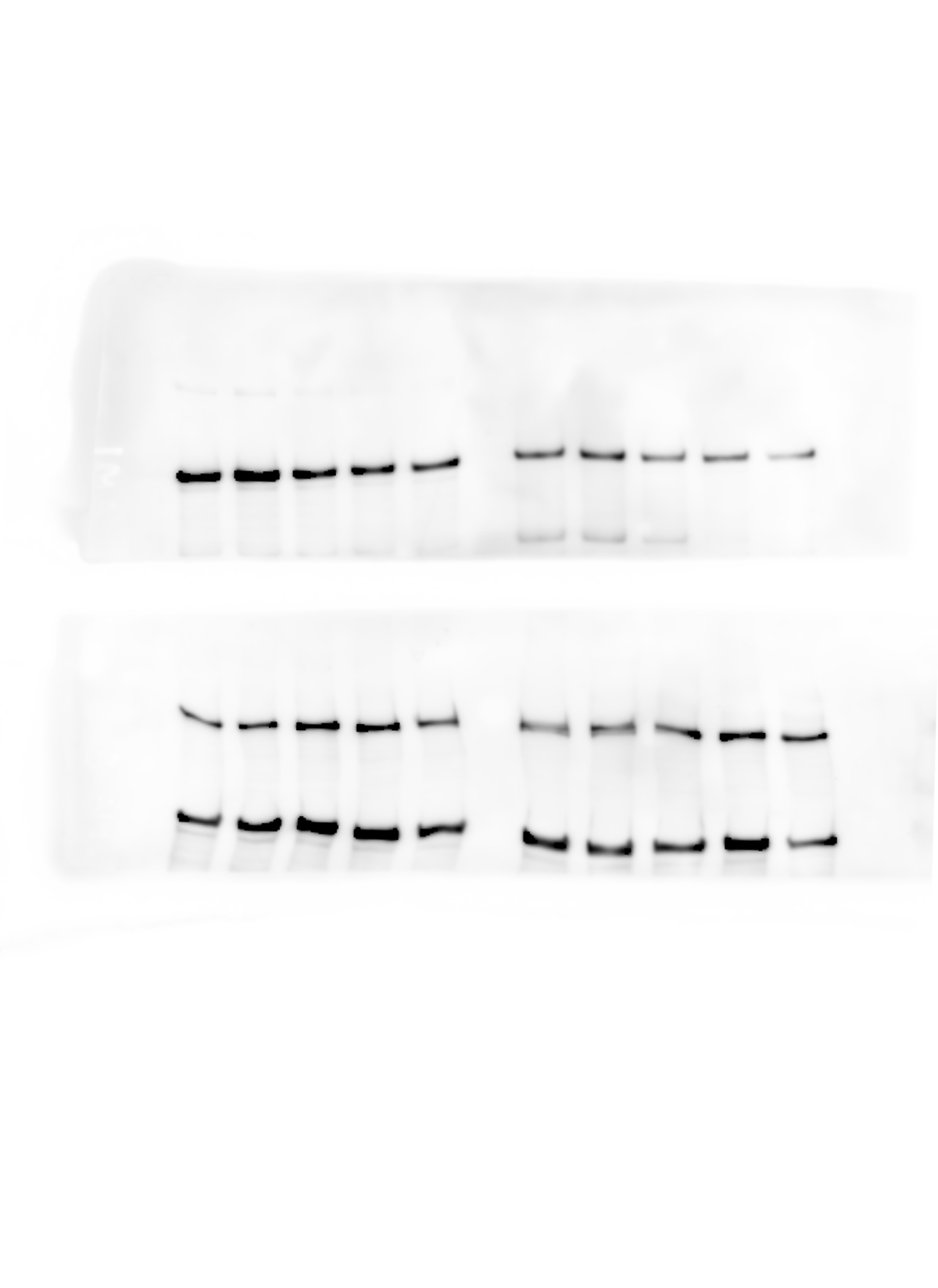

Supplement: Supplementary file 12 — Figure EV3 Source Data [file 44318_2026_754_MOESM12_ESM.zip › EV Figure3/EV 3F/EV3F_western_APOB.tif]

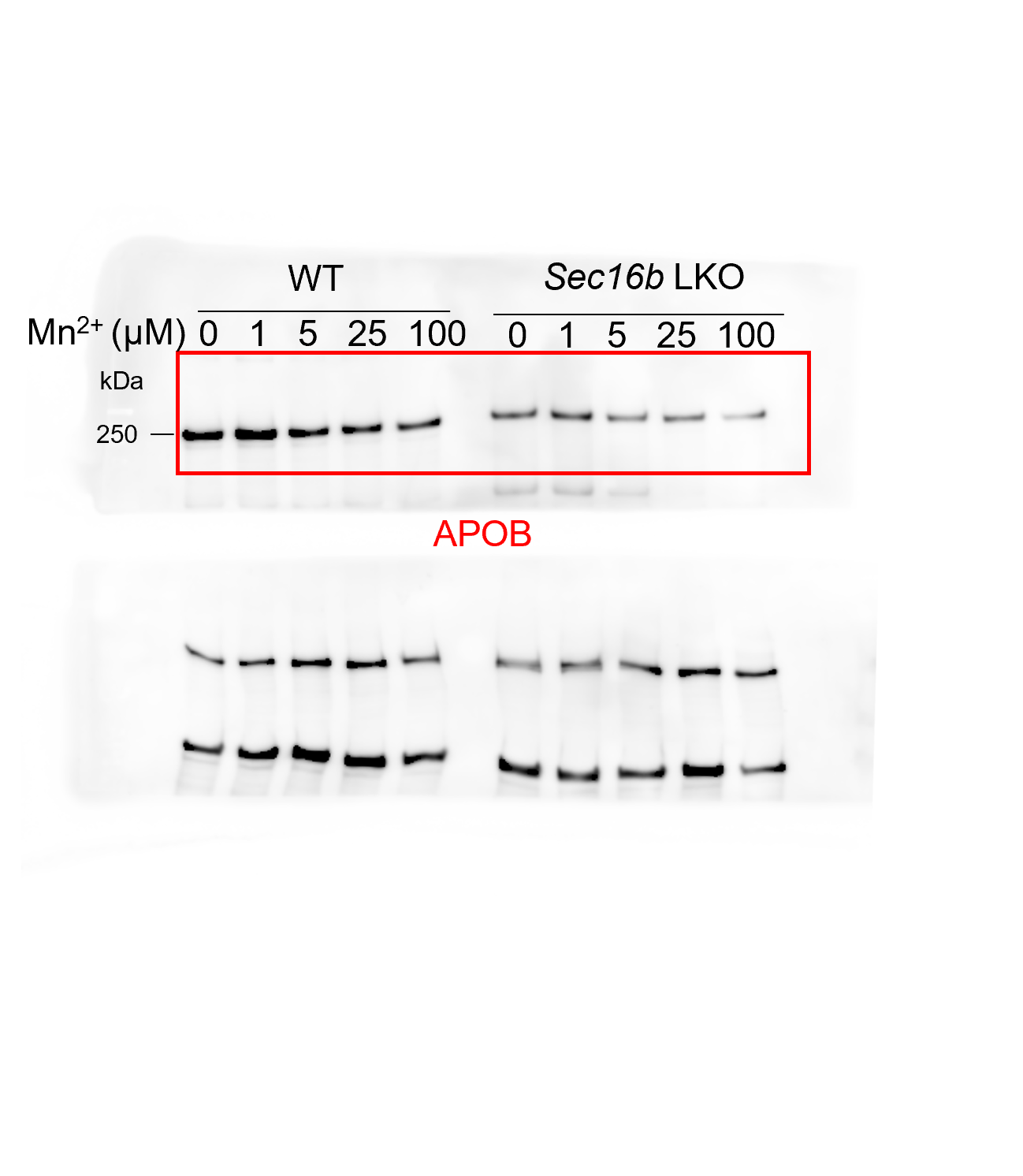

Supplement: Supplementary file 12 — Figure EV3 Source Data [file 44318_2026_754_MOESM12_ESM.zip › EV Figure3/EV 3F/EV3F_western_APOB_label.tif]

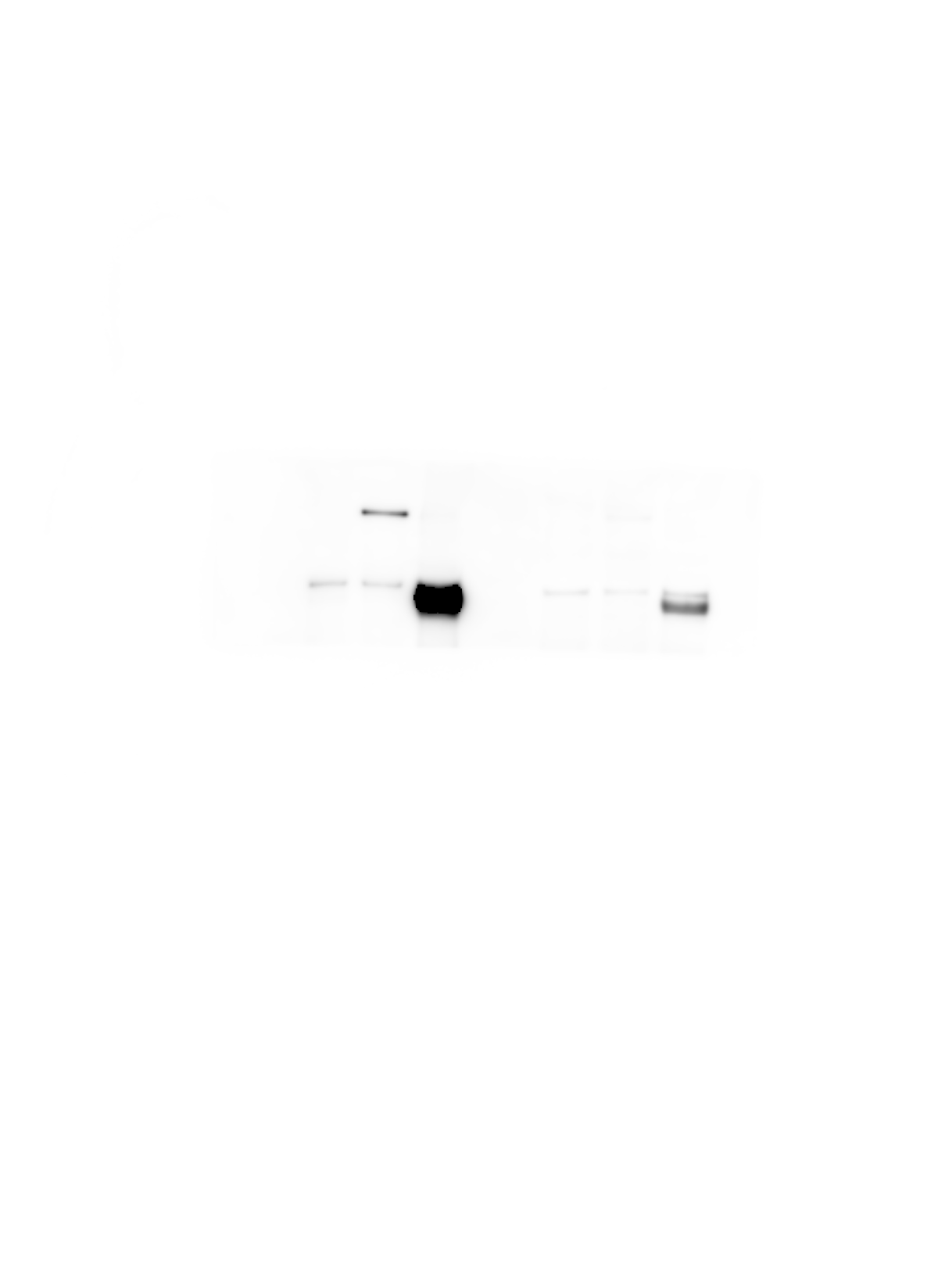

Supplement: Supplementary file 13 — Figure EV4 Source Data [file 44318_2026_754_MOESM13_ESM.zip › EV Figure4/EV 4A/EV4A_western_FLAG.tif]

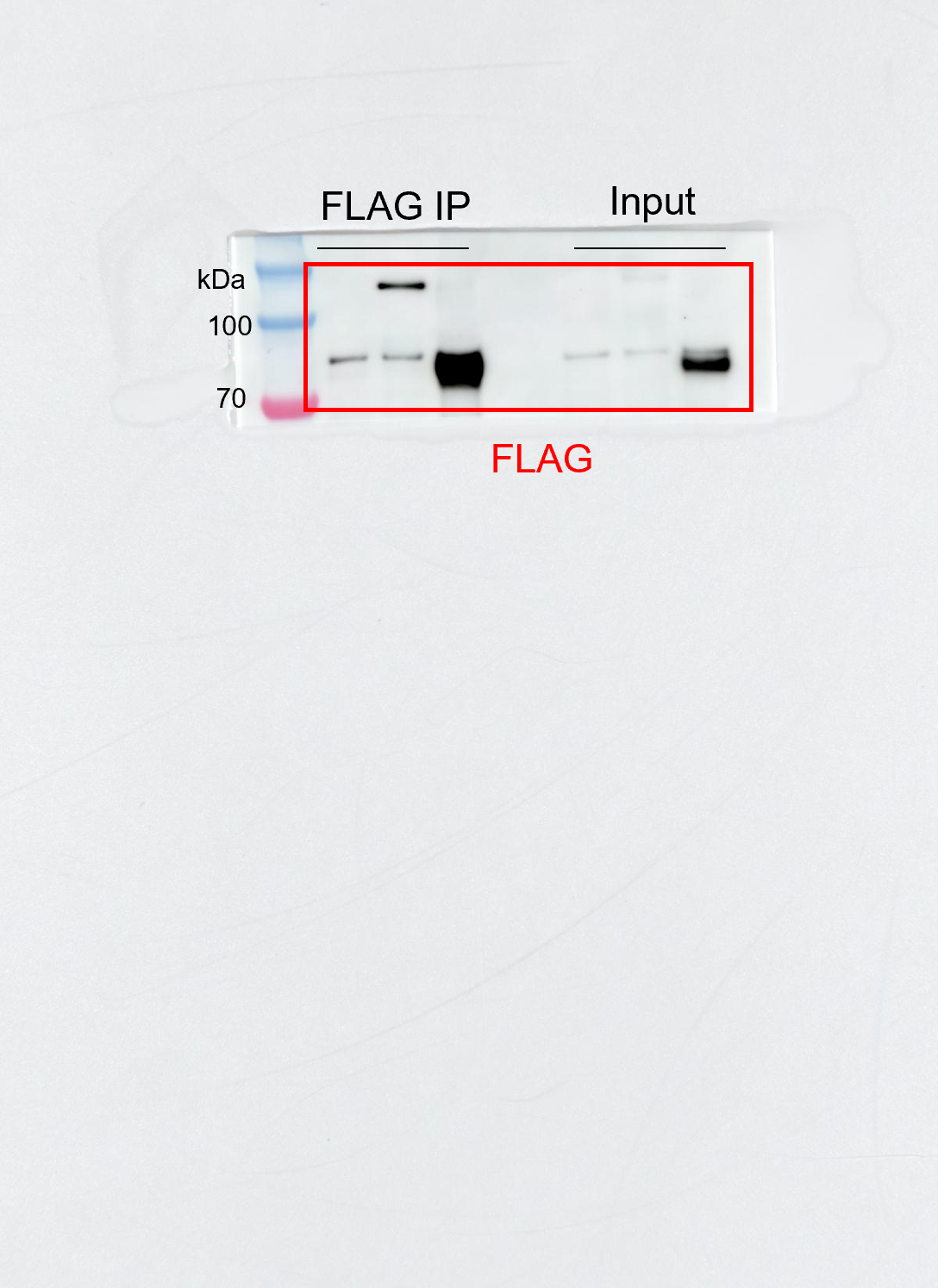

Supplement: Supplementary file 13 — Figure EV4 Source Data [file 44318_2026_754_MOESM13_ESM.zip › EV Figure4/EV 4A/EV4A_western_FLAG_label.tif]

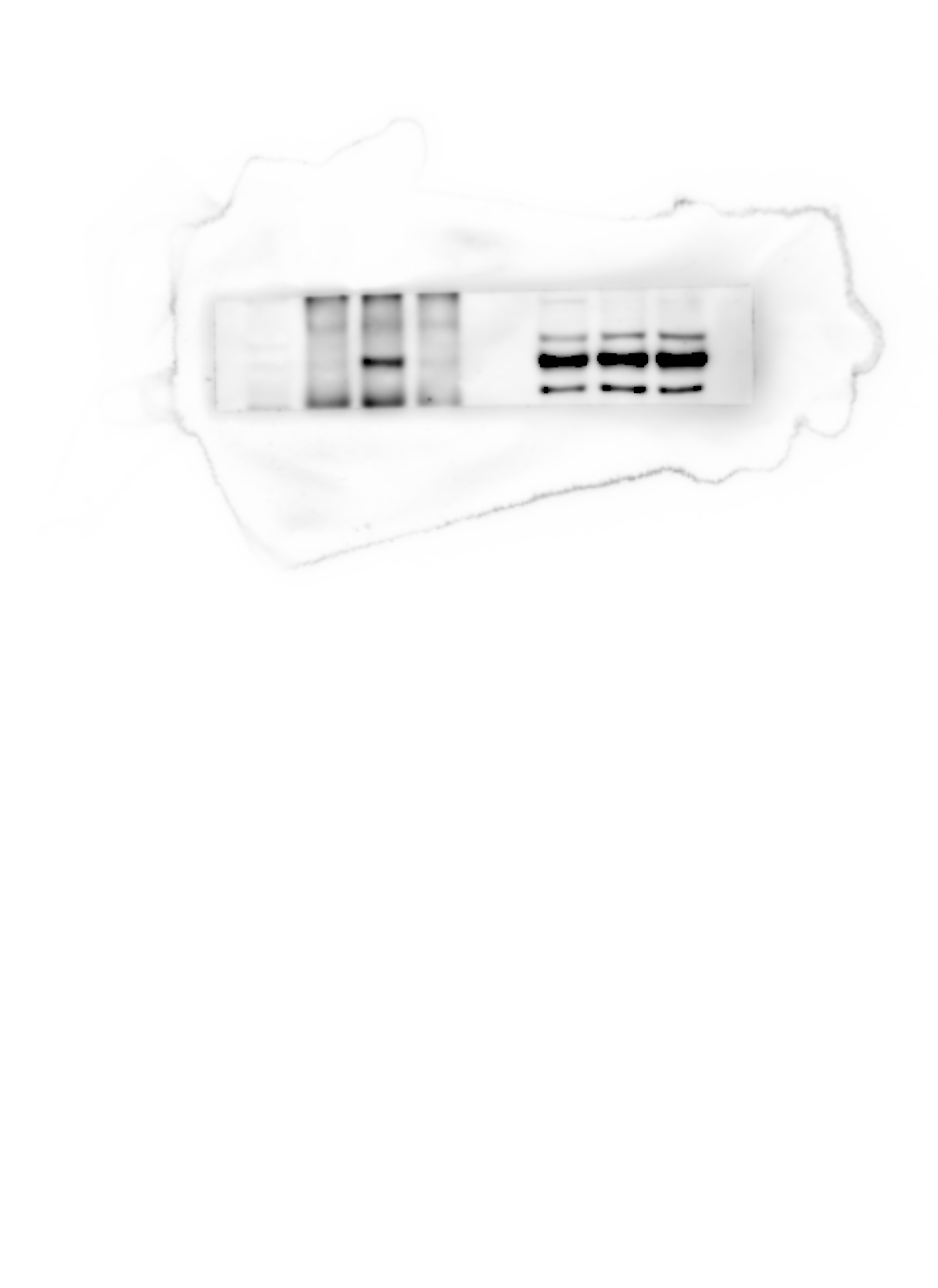

Supplement: Supplementary file 13 — Figure EV4 Source Data [file 44318_2026_754_MOESM13_ESM.zip › EV Figure4/EV 4A/EV4A_western_SEC13.tif]

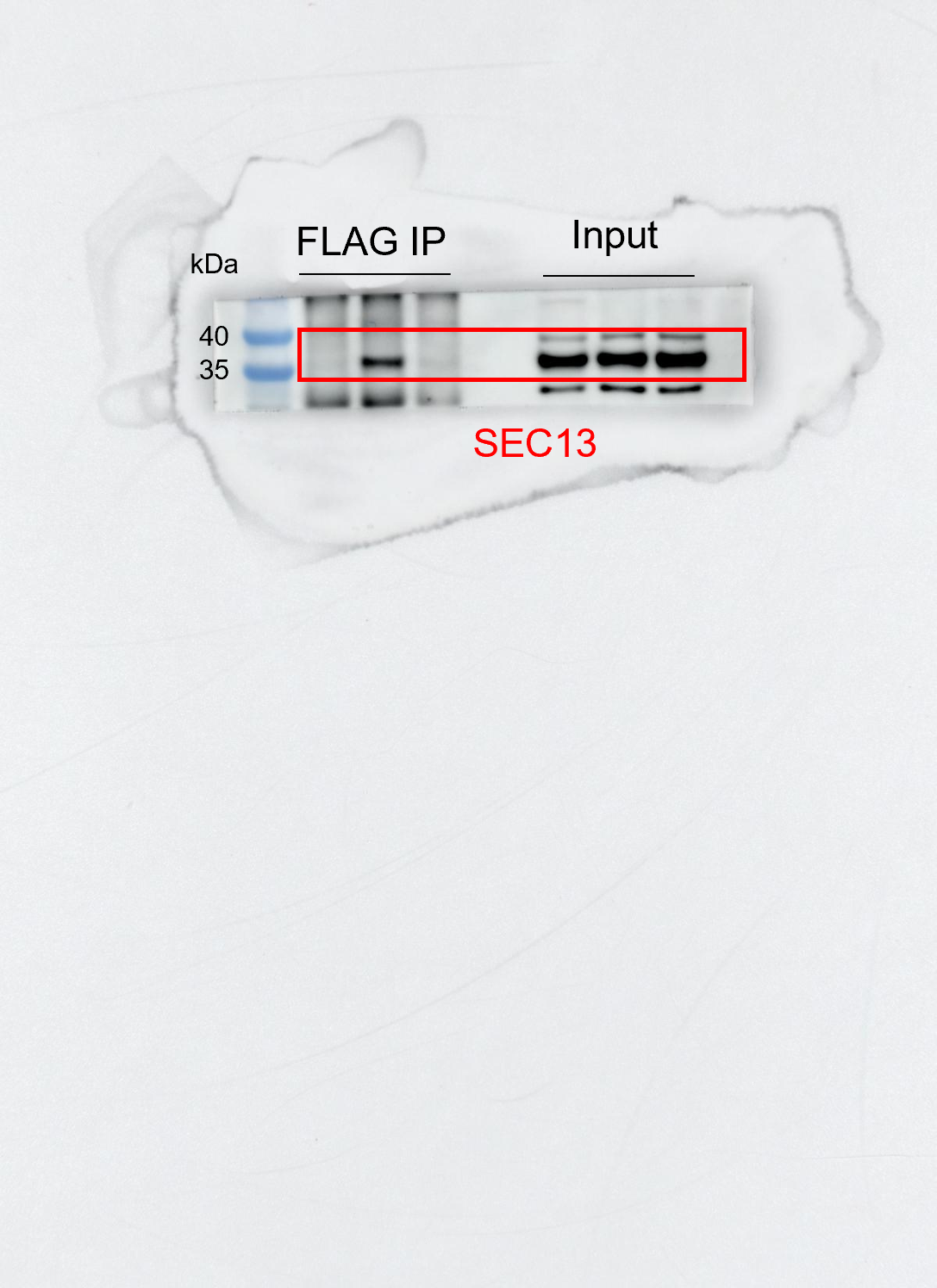

Supplement: Supplementary file 13 — Figure EV4 Source Data [file 44318_2026_754_MOESM13_ESM.zip › EV Figure4/EV 4A/EV4A_western_SEC13_label.tif]

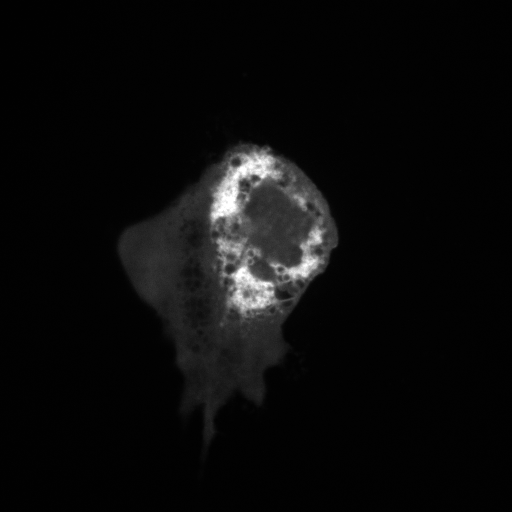

Supplement: Supplementary file 13 — Figure EV4 Source Data [file 44318_2026_754_MOESM13_ESM.zip › EV Figure4/EV 4B/EV4B_image_SEC16B dCCD+1,6-Hex.tif]

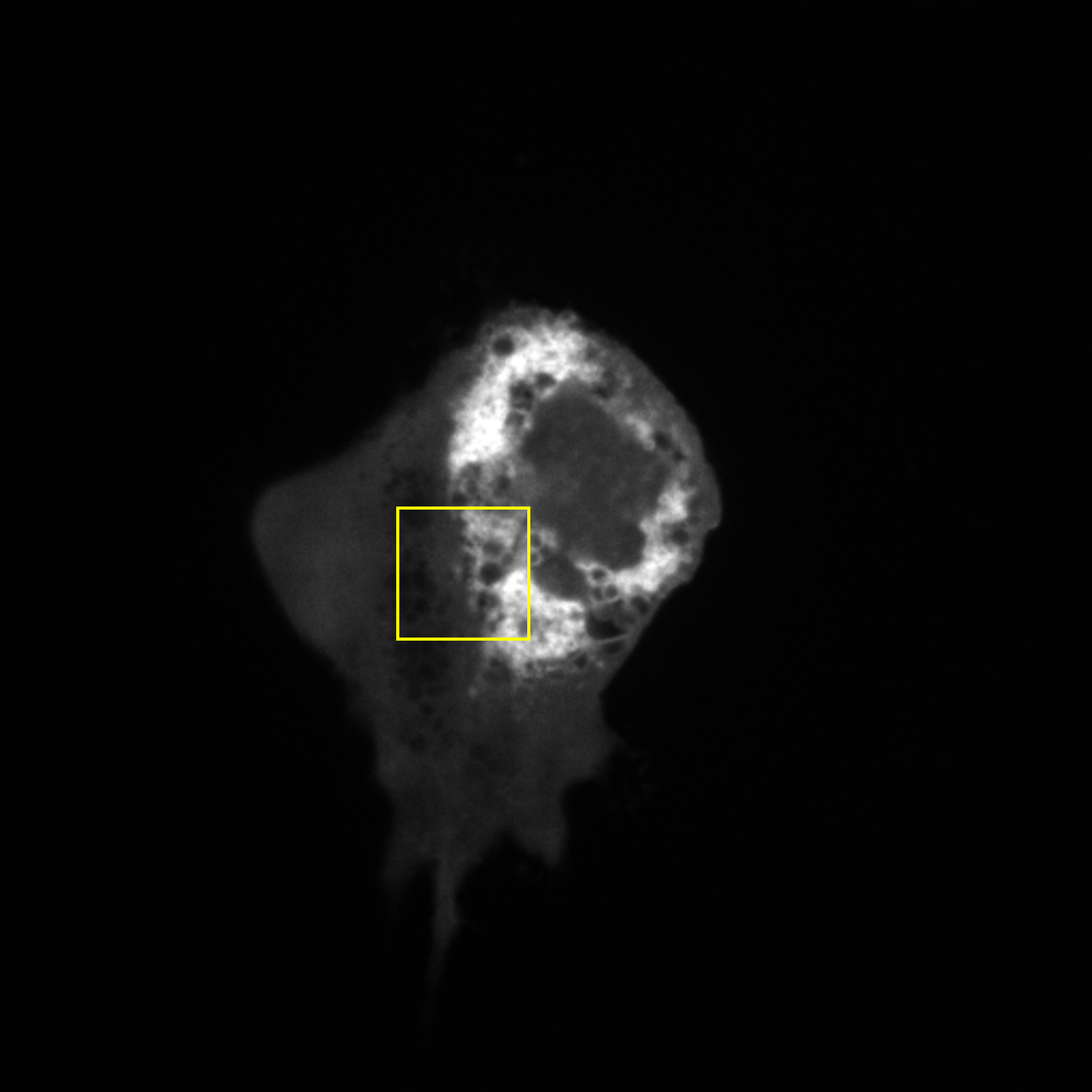

Supplement: Supplementary file 13 — Figure EV4 Source Data [file 44318_2026_754_MOESM13_ESM.zip › EV Figure4/EV 4B/EV4B_image_SEC16B dCCD+1,6-Hex_label.tif]

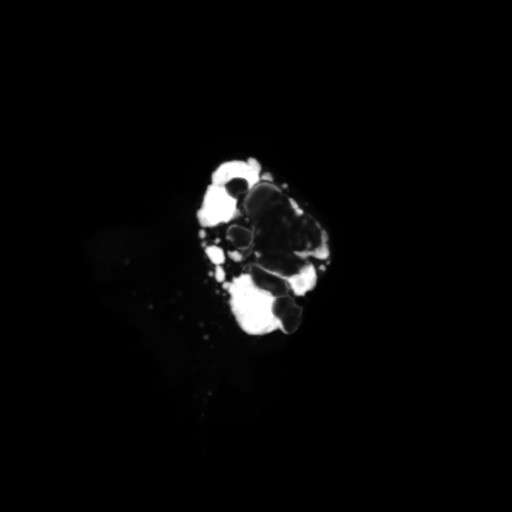

Supplement: Supplementary file 13 — Figure EV4 Source Data [file 44318_2026_754_MOESM13_ESM.zip › EV Figure4/EV 4B/EV4B_image_SEC16B dCCD.tif]

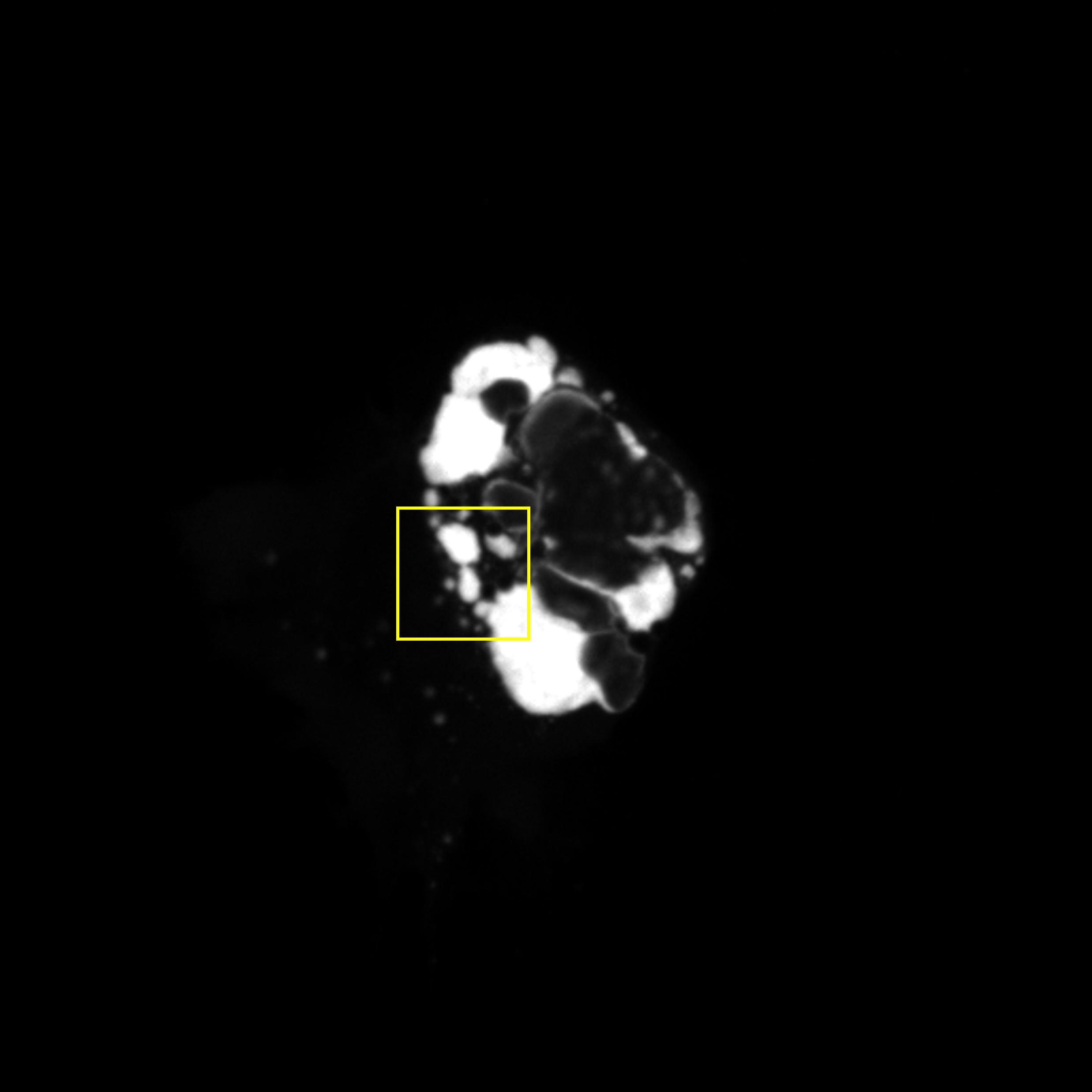

Supplement: Supplementary file 13 — Figure EV4 Source Data [file 44318_2026_754_MOESM13_ESM.zip › EV Figure4/EV 4B/EV4B_image_SEC16B dCCD_label.tif]

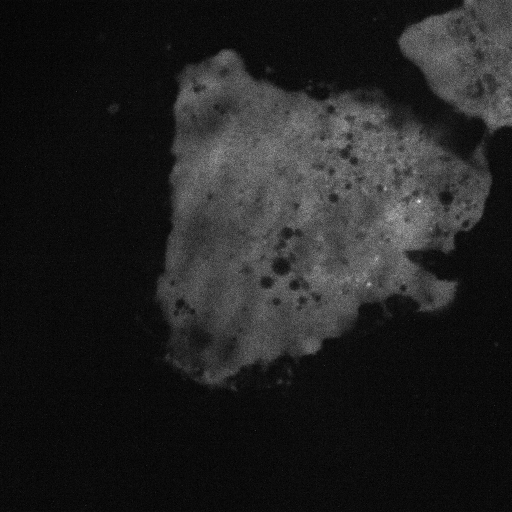

Supplement: Supplementary file 13 — Figure EV4 Source Data [file 44318_2026_754_MOESM13_ESM.zip › EV Figure4/EV 4B/EV4B_image_SEC16B+1,6-Hex.tif]

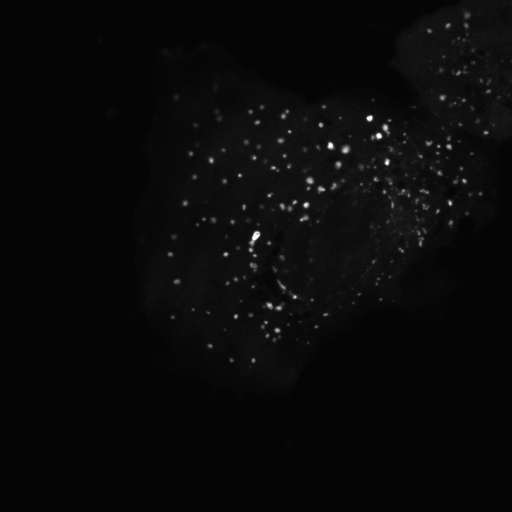

Supplement: Supplementary file 13 — Figure EV4 Source Data [file 44318_2026_754_MOESM13_ESM.zip › EV Figure4/EV 4B/EV4B_image_SEC16B.tif]

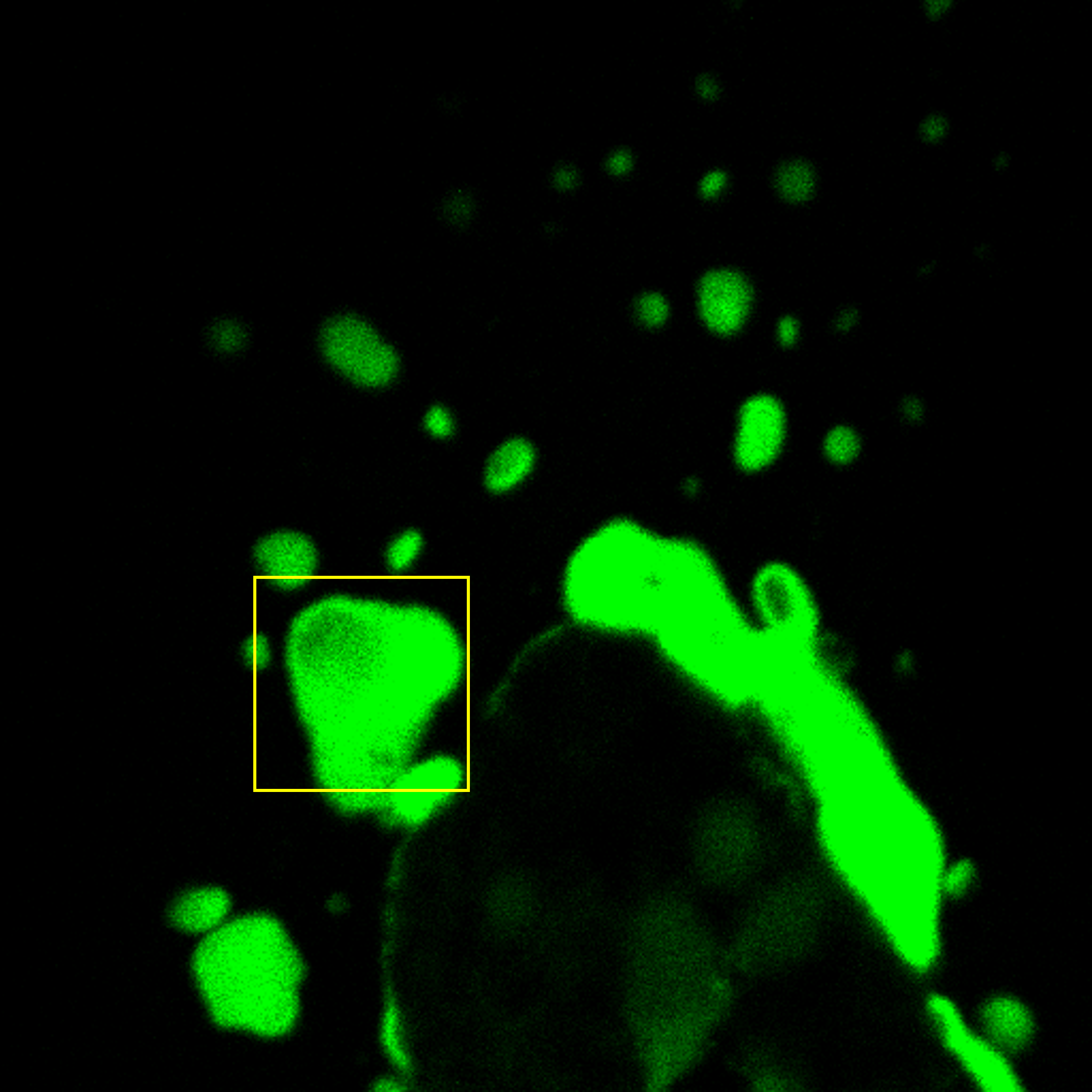

Supplement: Supplementary file 13 — Figure EV4 Source Data [file 44318_2026_754_MOESM13_ESM.zip › EV Figure4/EV 4C/EV4C_image_SEC16B dCCD_50s_label.tif]

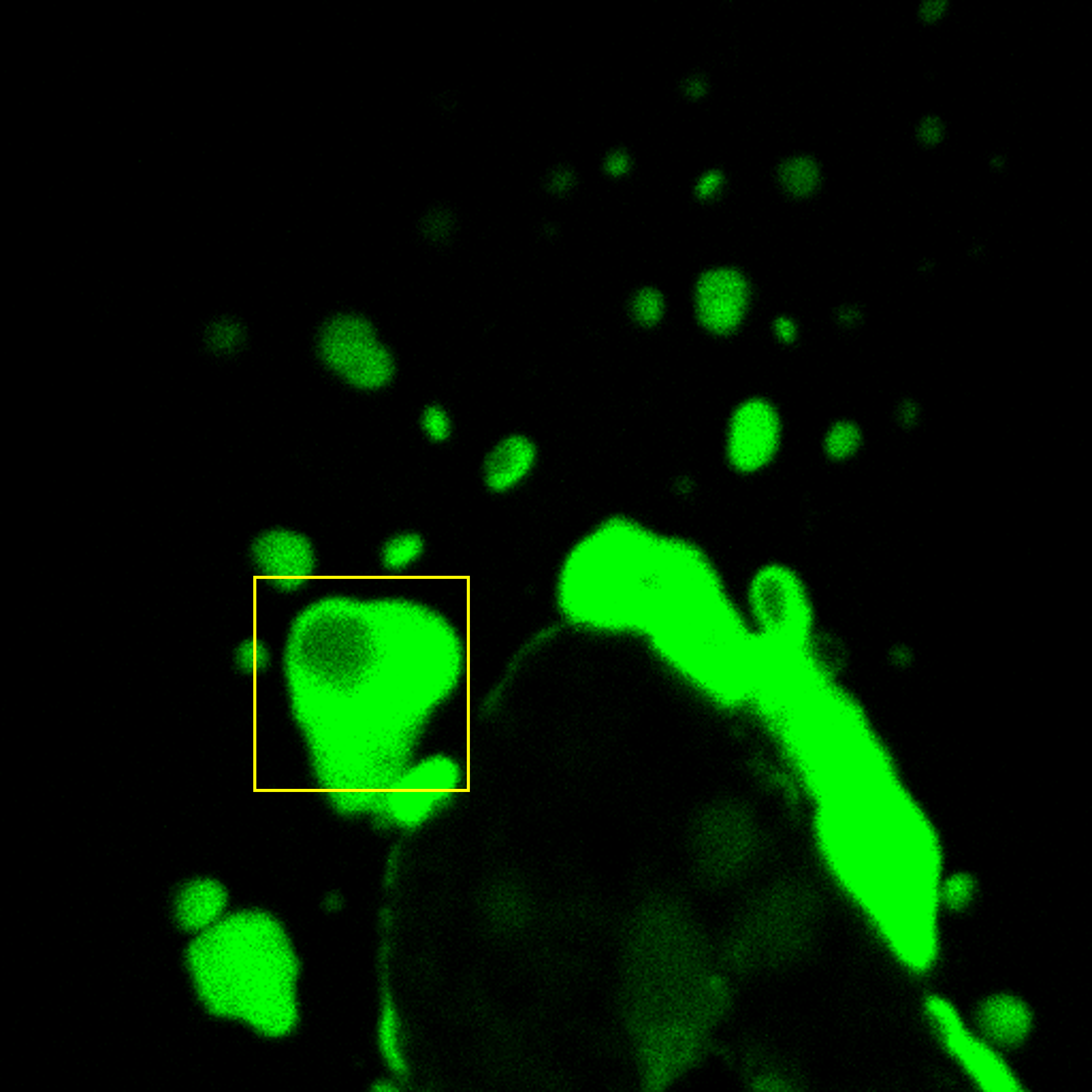

Supplement: Supplementary file 13 — Figure EV4 Source Data [file 44318_2026_754_MOESM13_ESM.zip › EV Figure4/EV 4C/EV4C_image_SEC16B dCCD_5s_label.tif]

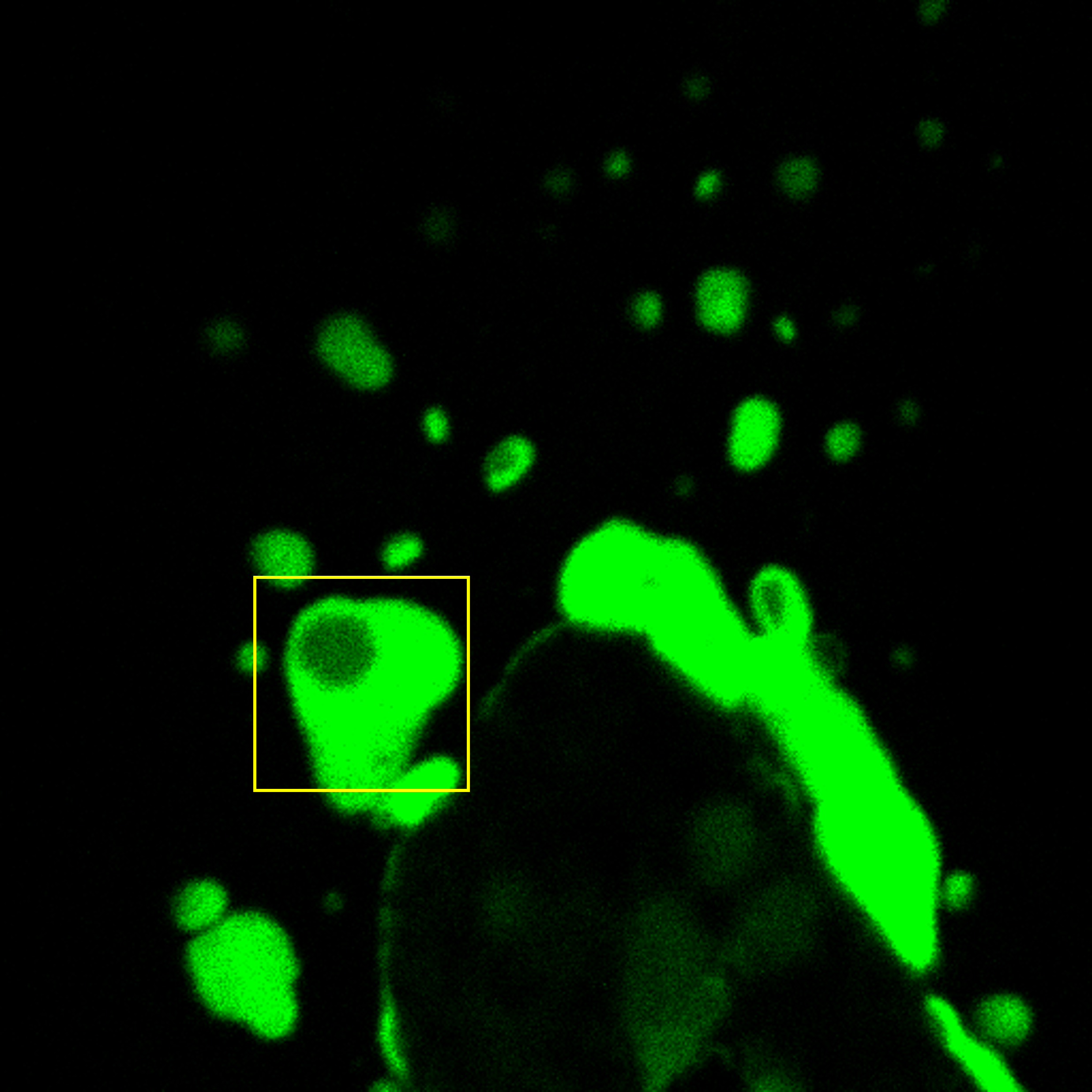

Supplement: Supplementary file 13 — Figure EV4 Source Data [file 44318_2026_754_MOESM13_ESM.zip › EV Figure4/EV 4C/EV4C_image_SEC16B dCCD_Bleach_label.tif]

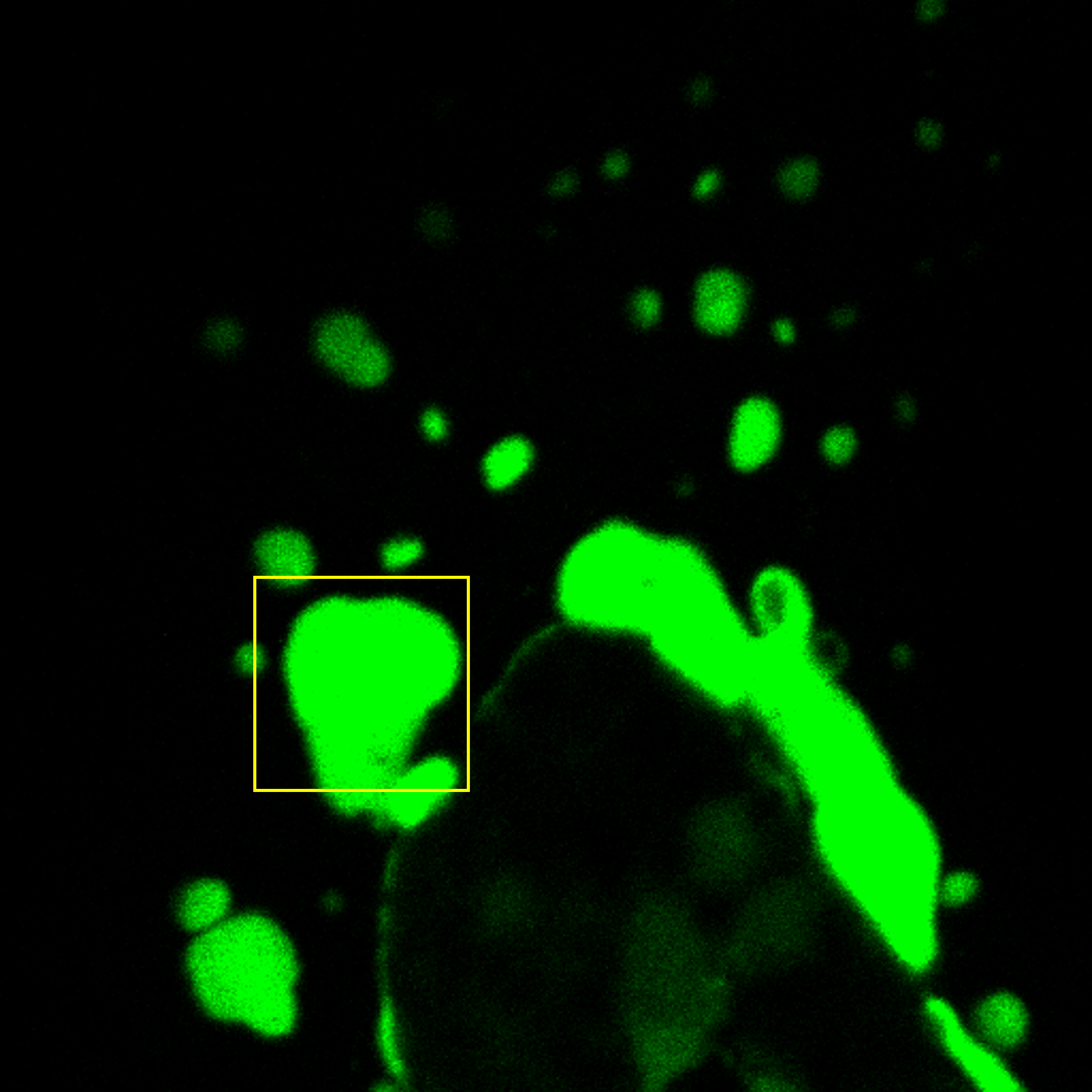

Supplement: Supplementary file 13 — Figure EV4 Source Data [file 44318_2026_754_MOESM13_ESM.zip › EV Figure4/EV 4C/EV4C_image_SEC16B dCCD_pre_label.tif]

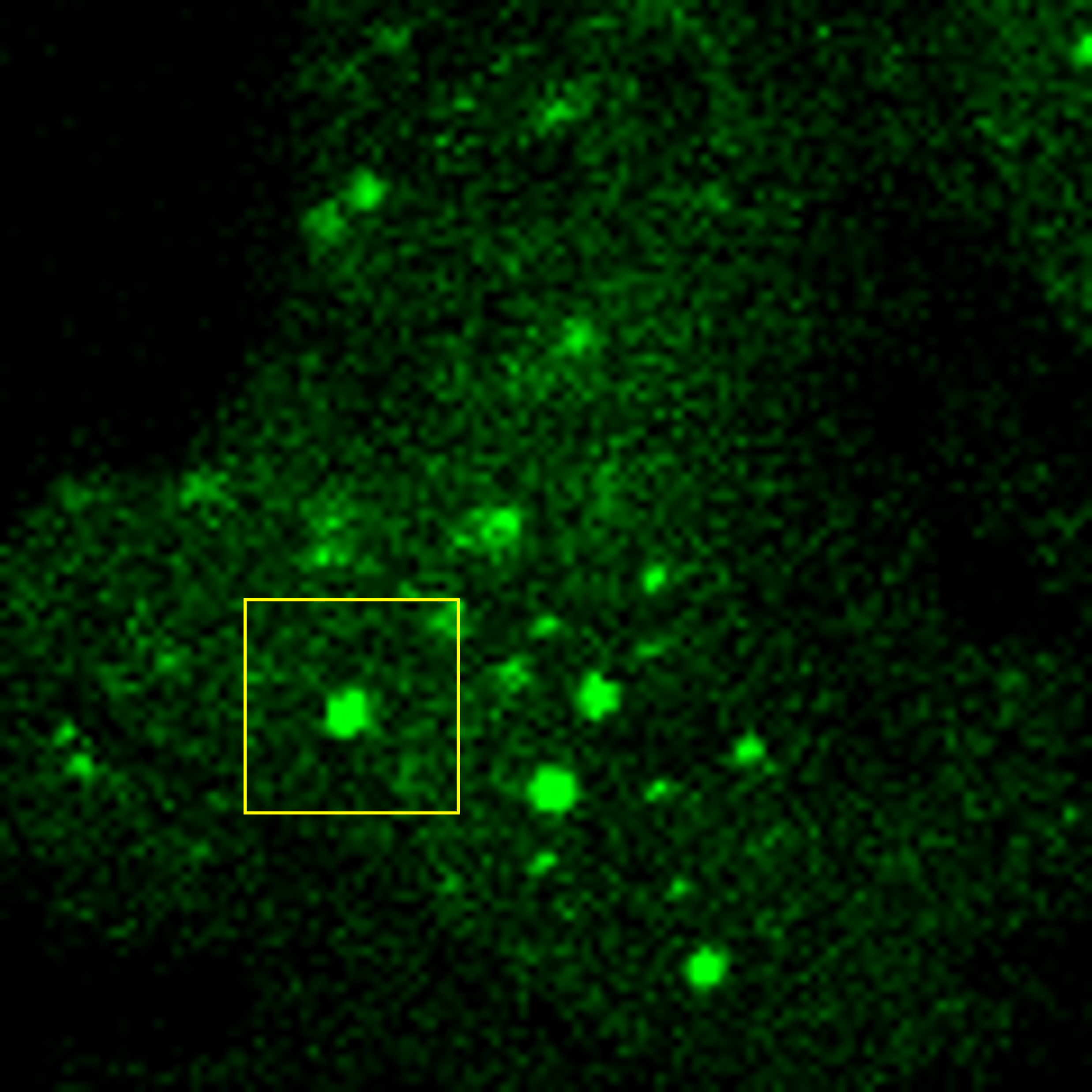

Supplement: Supplementary file 13 — Figure EV4 Source Data [file 44318_2026_754_MOESM13_ESM.zip › EV Figure4/EV 4C/EV4C_image_SEC16B FL_50s_label.tif]

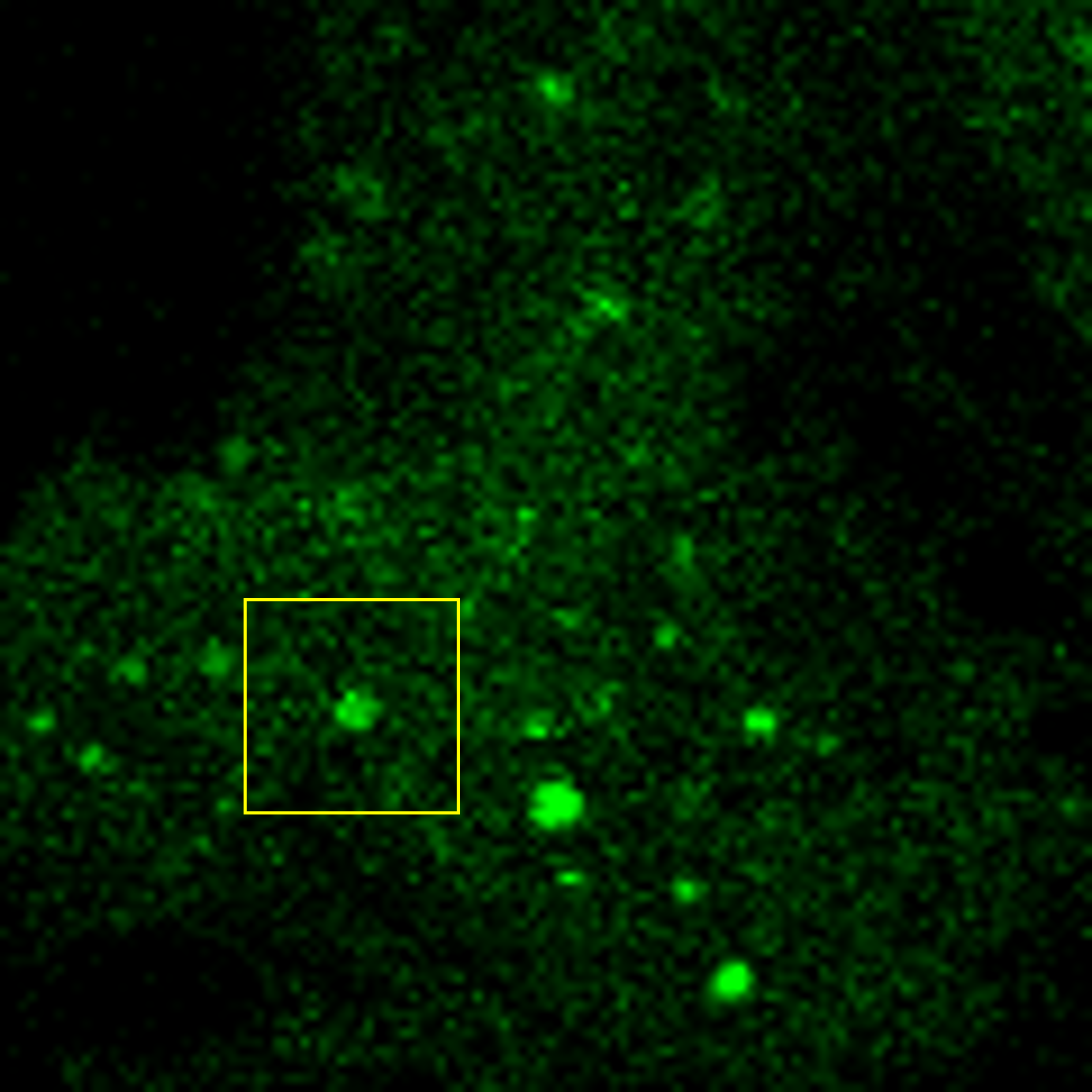

Supplement: Supplementary file 13 — Figure EV4 Source Data [file 44318_2026_754_MOESM13_ESM.zip › EV Figure4/EV 4C/EV4C_image_SEC16B FL_5s_label.tif]

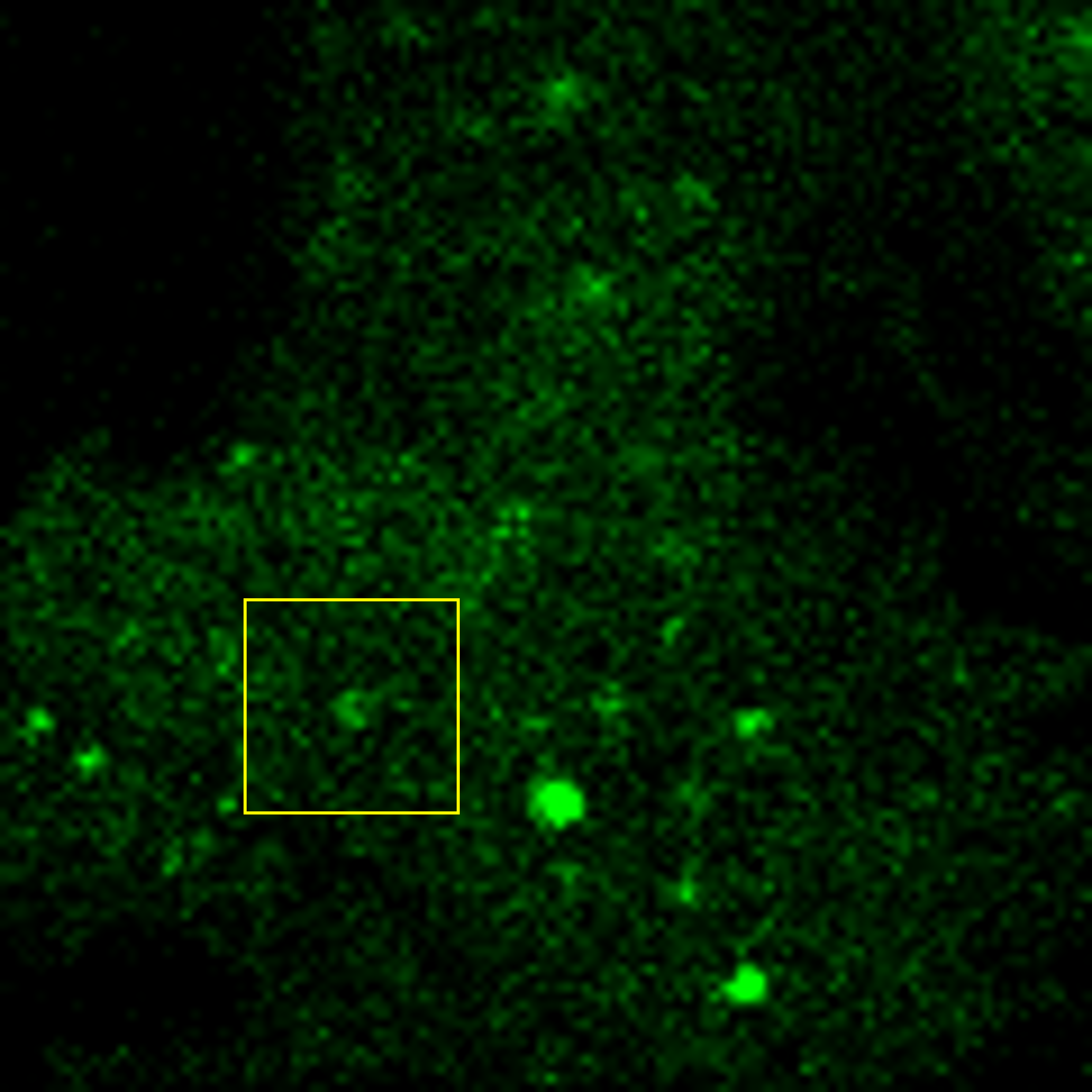

Supplement: Supplementary file 13 — Figure EV4 Source Data [file 44318_2026_754_MOESM13_ESM.zip › EV Figure4/EV 4C/EV4C_image_SEC16B FL_Bleach_label.tif]

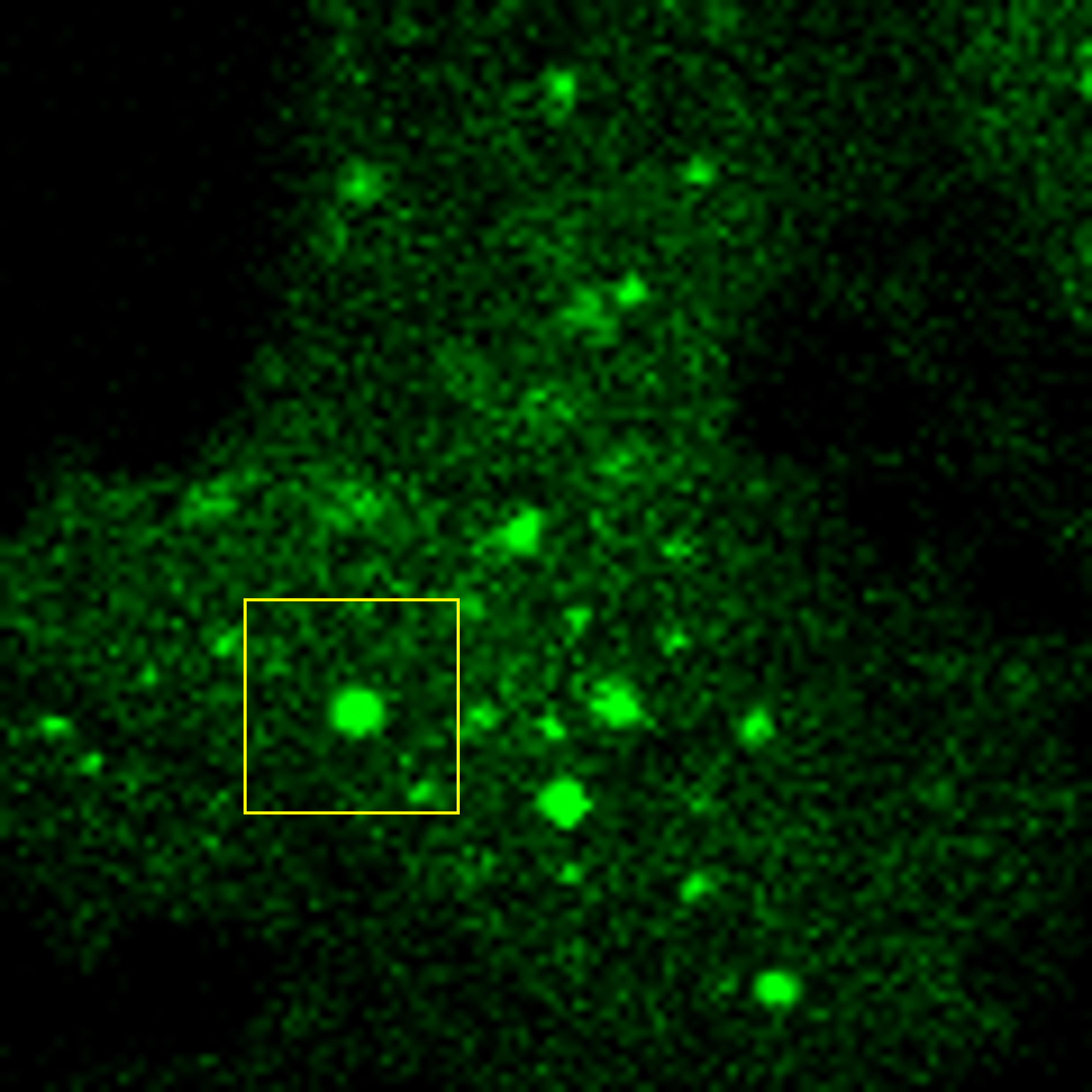

Supplement: Supplementary file 13 — Figure EV4 Source Data [file 44318_2026_754_MOESM13_ESM.zip › EV Figure4/EV 4C/EV4C_image_SEC16B FL_pre_label.tif]

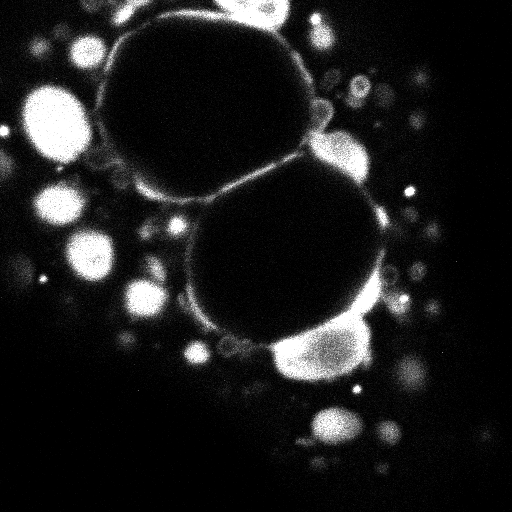

Supplement: Supplementary file 13 — Figure EV4 Source Data [file 44318_2026_754_MOESM13_ESM.zip › EV Figure4/EV 4D/EV4D_image_SEC16B dCCD_SEC24_50s.tif]

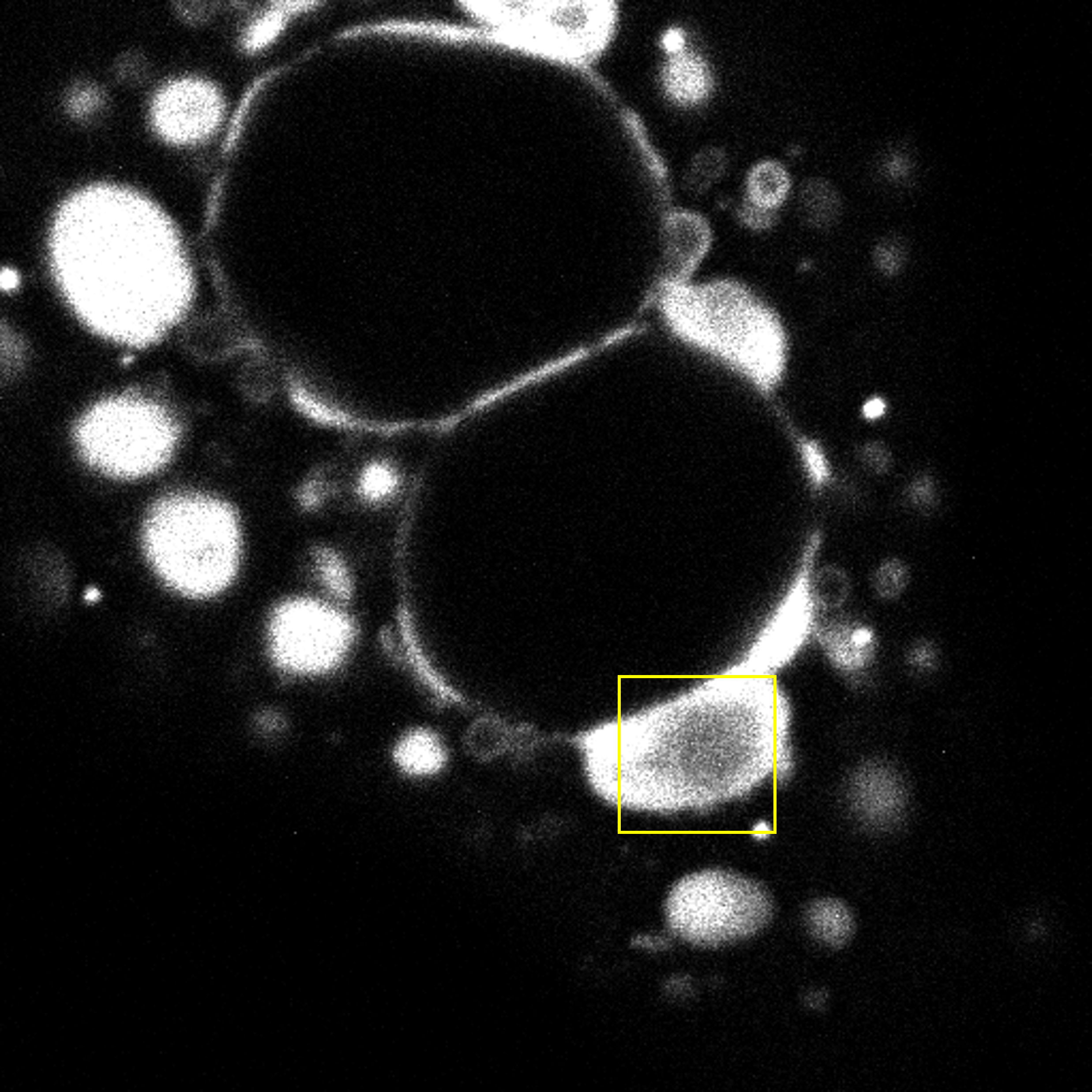

Supplement: Supplementary file 13 — Figure EV4 Source Data [file 44318_2026_754_MOESM13_ESM.zip › EV Figure4/EV 4D/EV4D_image_SEC16B dCCD_SEC24_50s_label.tif]

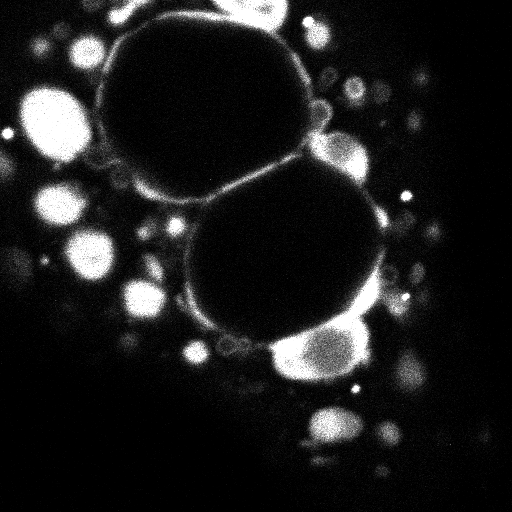

Supplement: Supplementary file 13 — Figure EV4 Source Data [file 44318_2026_754_MOESM13_ESM.zip › EV Figure4/EV 4D/EV4D_image_SEC16B dCCD_SEC24_5s.tif]

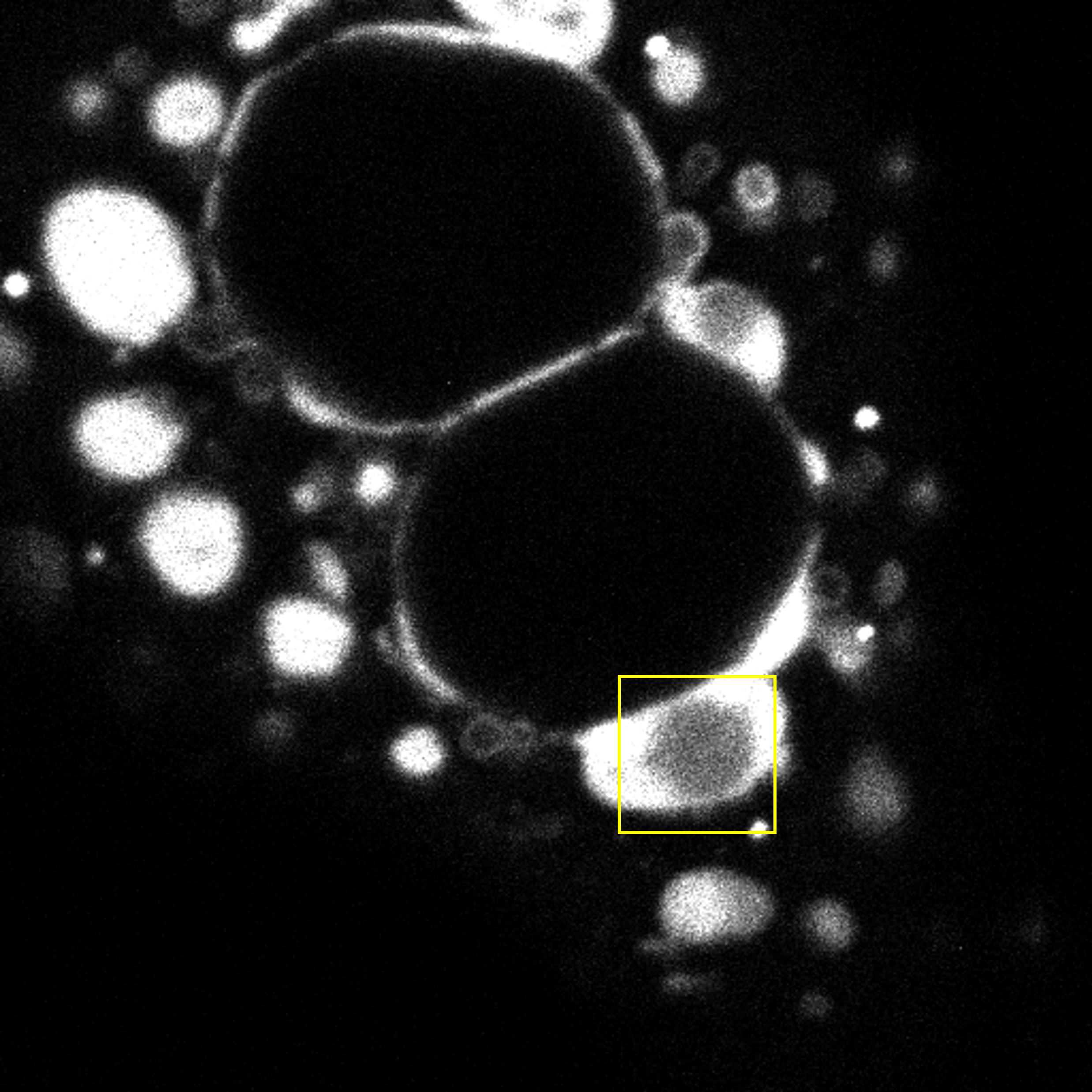

Supplement: Supplementary file 13 — Figure EV4 Source Data [file 44318_2026_754_MOESM13_ESM.zip › EV Figure4/EV 4D/EV4D_image_SEC16B dCCD_SEC24_5s_label.tif]

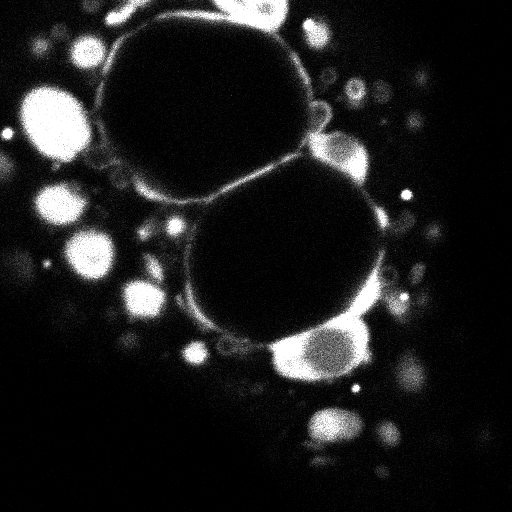

Supplement: Supplementary file 13 — Figure EV4 Source Data [file 44318_2026_754_MOESM13_ESM.zip › EV Figure4/EV 4D/EV4D_image_SEC16B dCCD_SEC24_Bleach.tif]

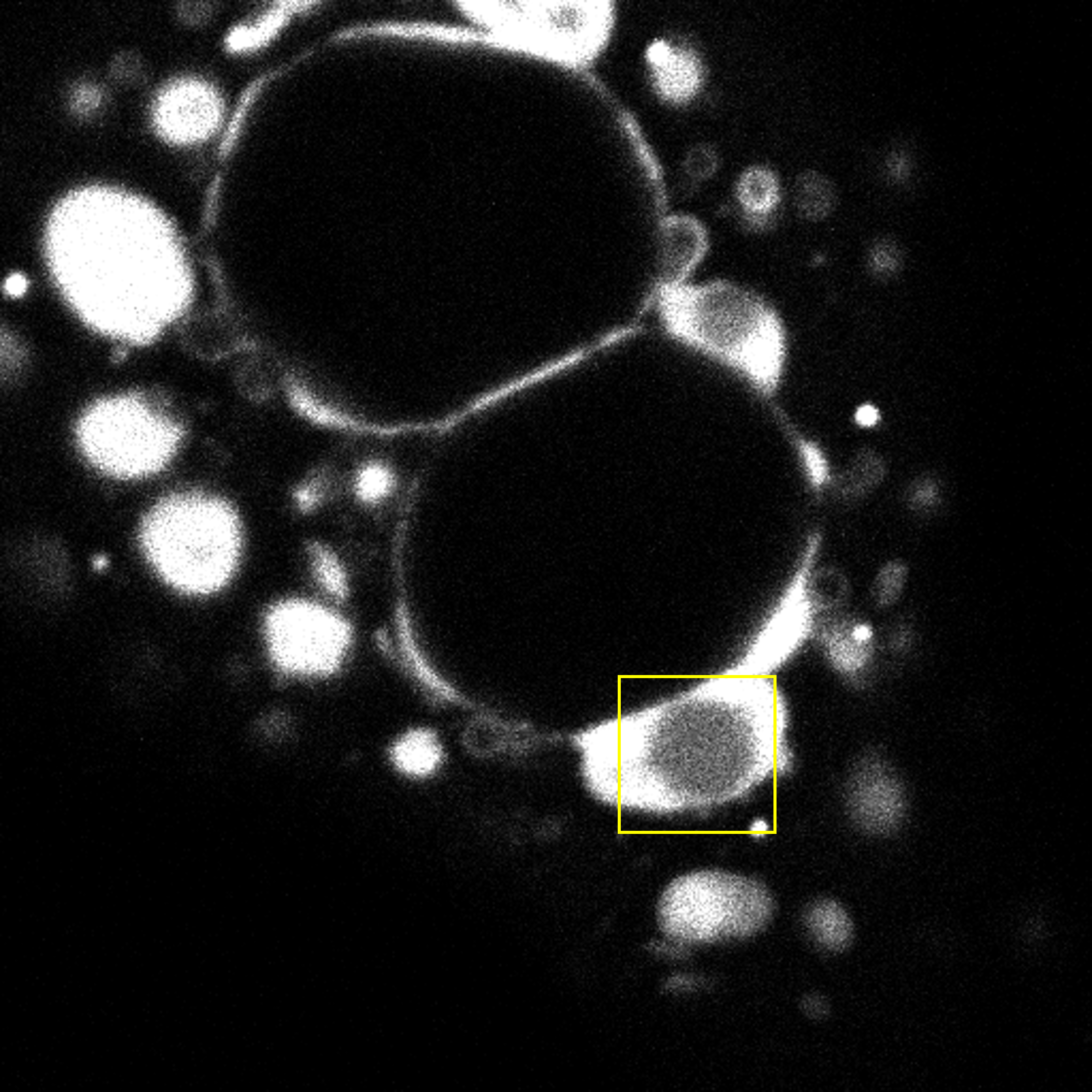

Supplement: Supplementary file 13 — Figure EV4 Source Data [file 44318_2026_754_MOESM13_ESM.zip › EV Figure4/EV 4D/EV4D_image_SEC16B dCCD_SEC24_Bleach_label.tif]

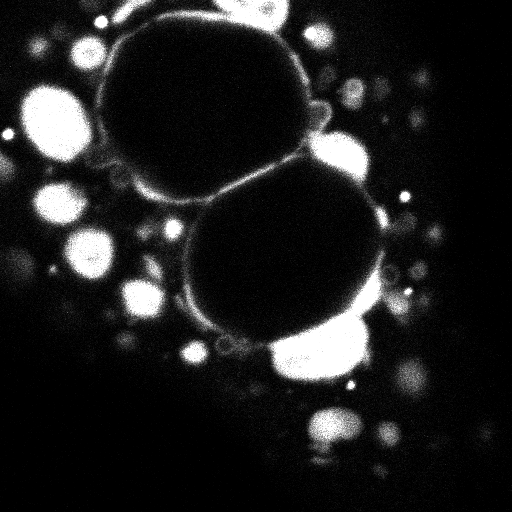

Supplement: Supplementary file 13 — Figure EV4 Source Data [file 44318_2026_754_MOESM13_ESM.zip › EV Figure4/EV 4D/EV4D_image_SEC16B dCCD_SEC24_pre.tif]

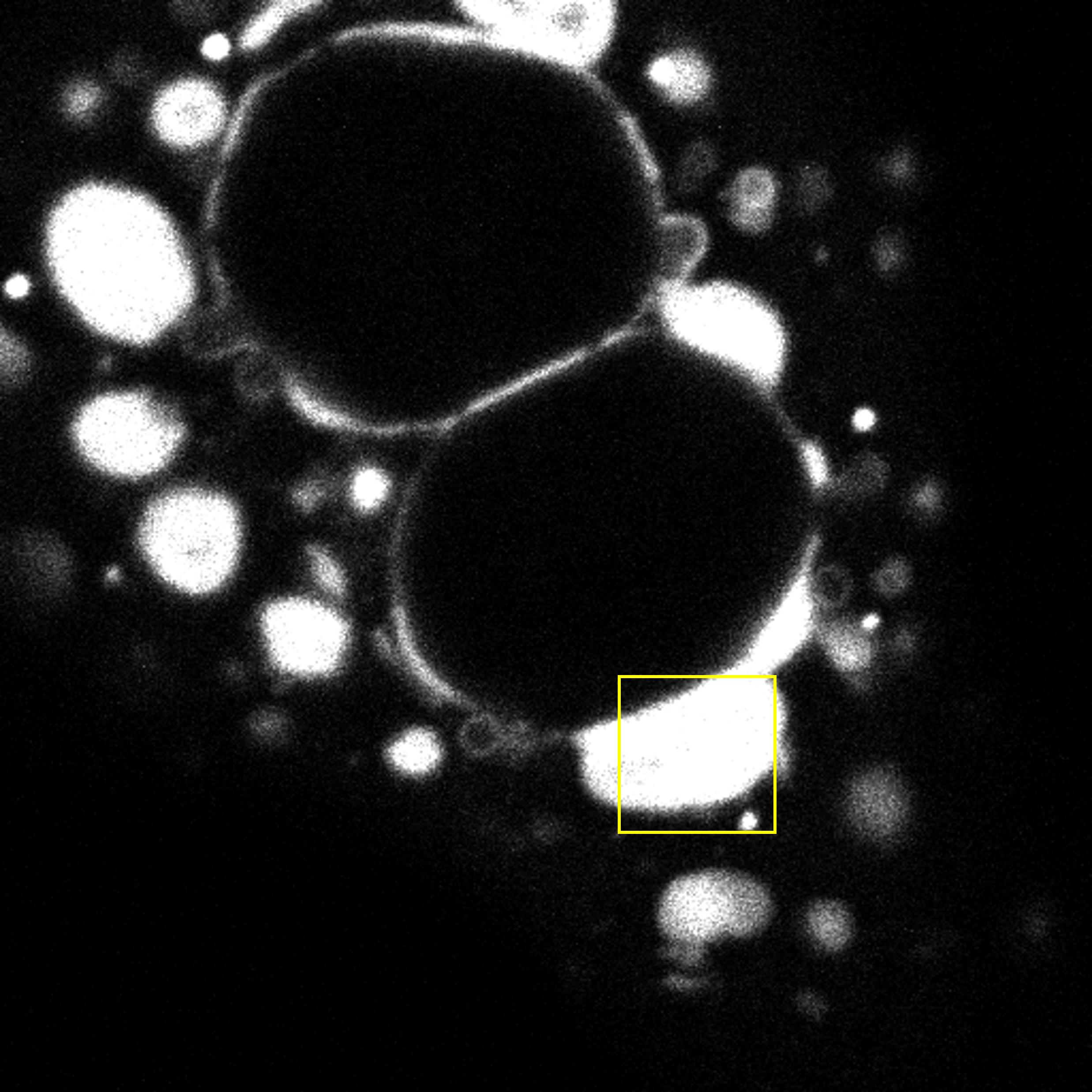

Supplement: Supplementary file 13 — Figure EV4 Source Data [file 44318_2026_754_MOESM13_ESM.zip › EV Figure4/EV 4D/EV4D_image_SEC16B dCCD_SEC24_pre_label.tif]

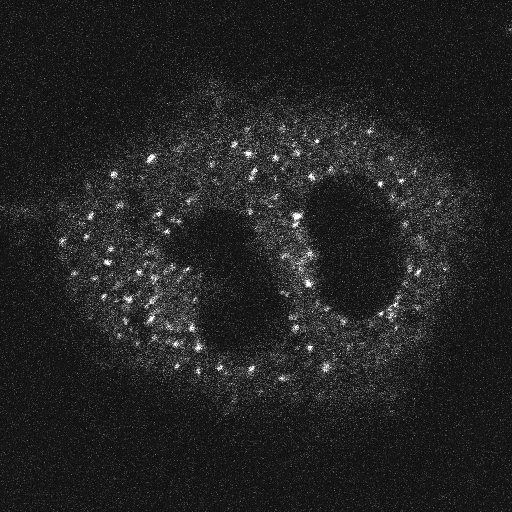

Supplement: Supplementary file 13 — Figure EV4 Source Data [file 44318_2026_754_MOESM13_ESM.zip › EV Figure4/EV 4D/EV4D_image_SEC16B FL_SEC24_50s.tif]

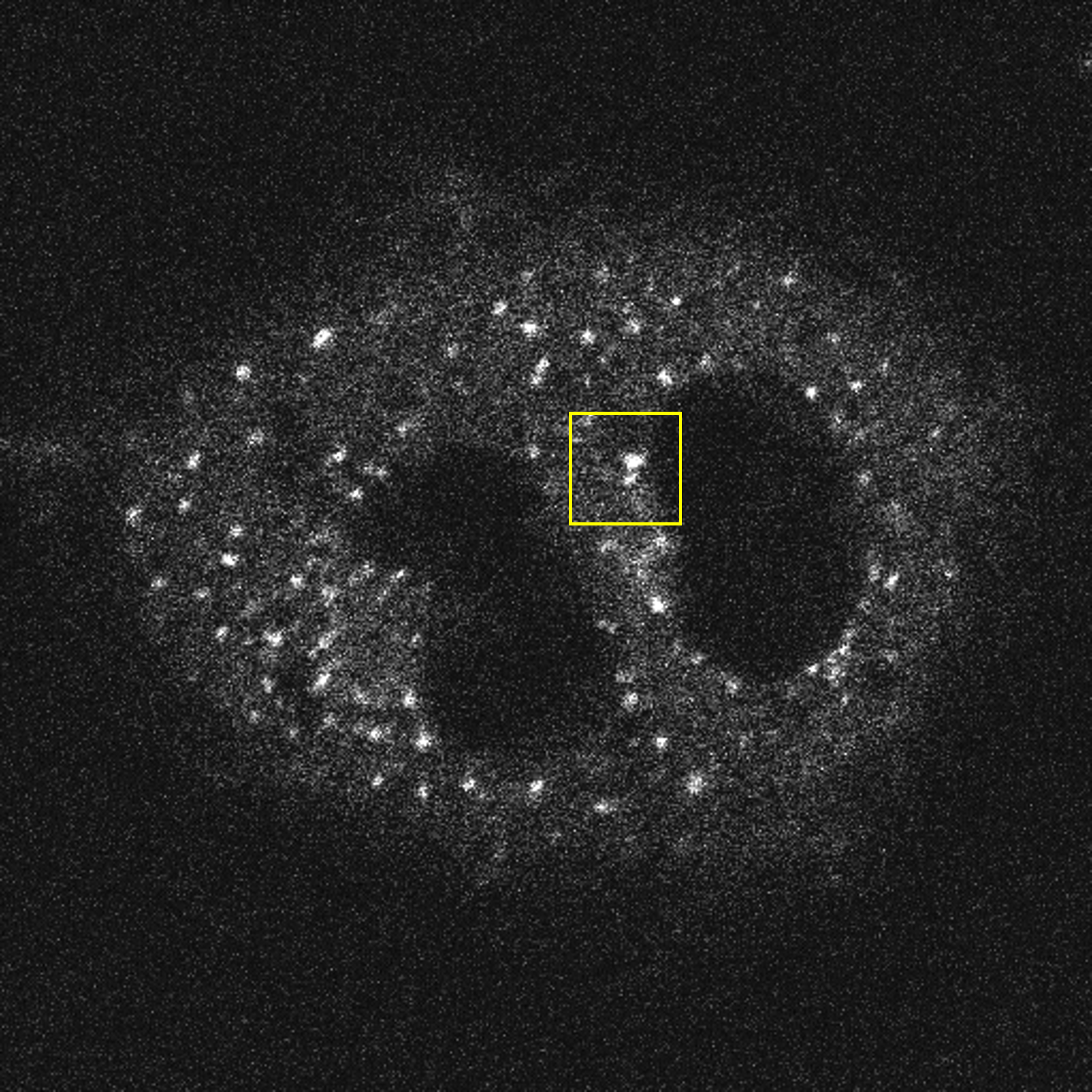

Supplement: Supplementary file 13 — Figure EV4 Source Data [file 44318_2026_754_MOESM13_ESM.zip › EV Figure4/EV 4D/EV4D_image_SEC16B FL_SEC24_50s_label.tif]

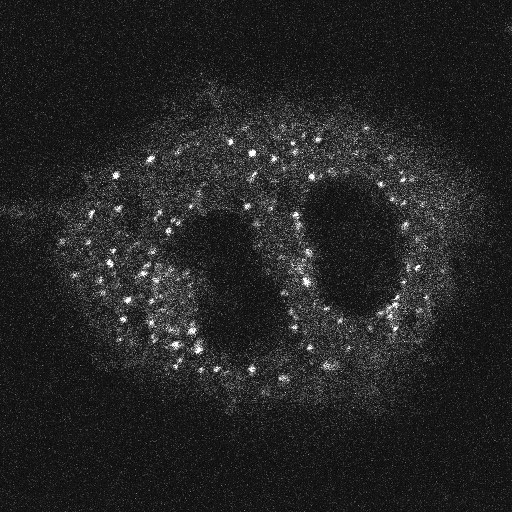

Supplement: Supplementary file 13 — Figure EV4 Source Data [file 44318_2026_754_MOESM13_ESM.zip › EV Figure4/EV 4D/EV4D_image_SEC16B FL_SEC24_5s.tif]

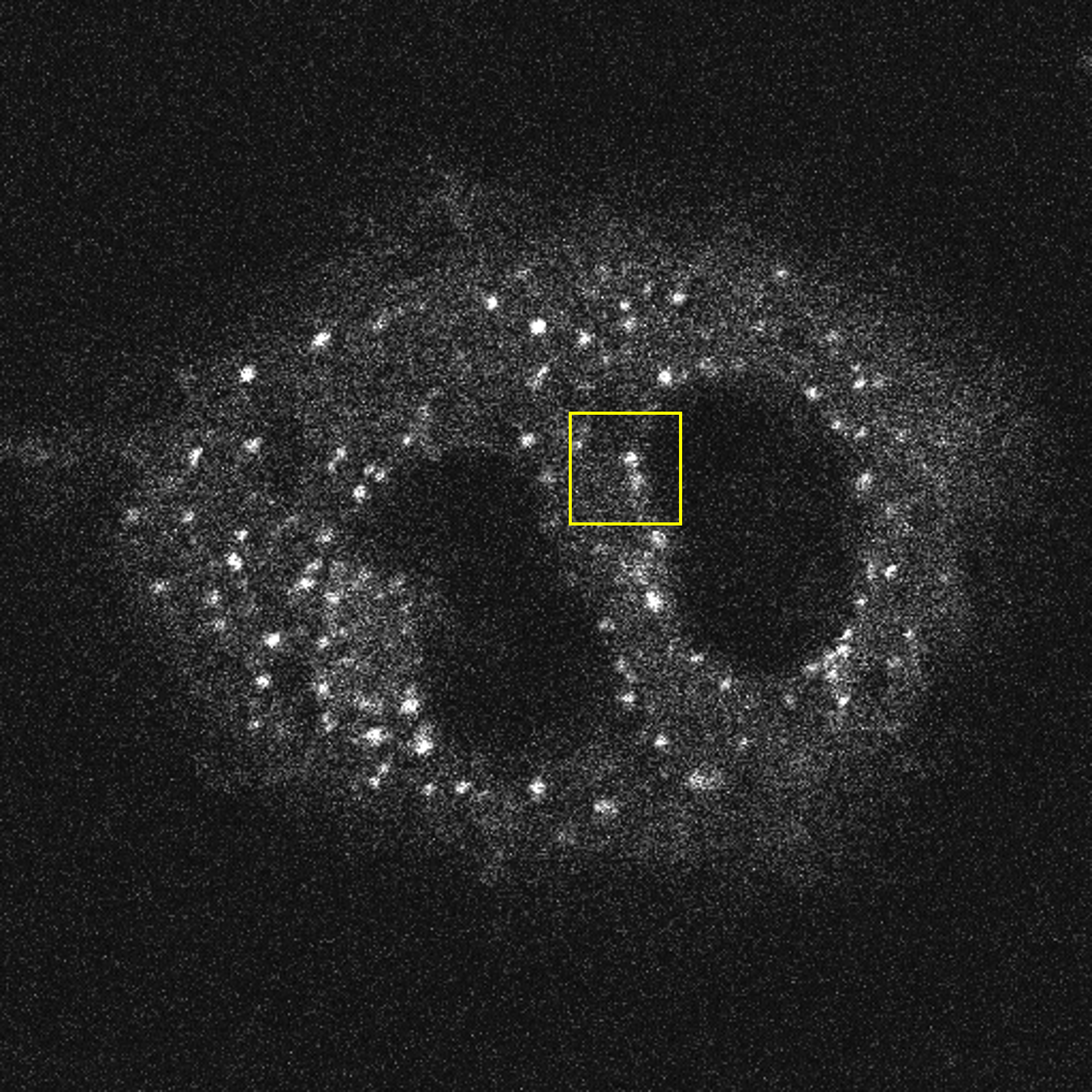

Supplement: Supplementary file 13 — Figure EV4 Source Data [file 44318_2026_754_MOESM13_ESM.zip › EV Figure4/EV 4D/EV4D_image_SEC16B FL_SEC24_5s_label.tif]

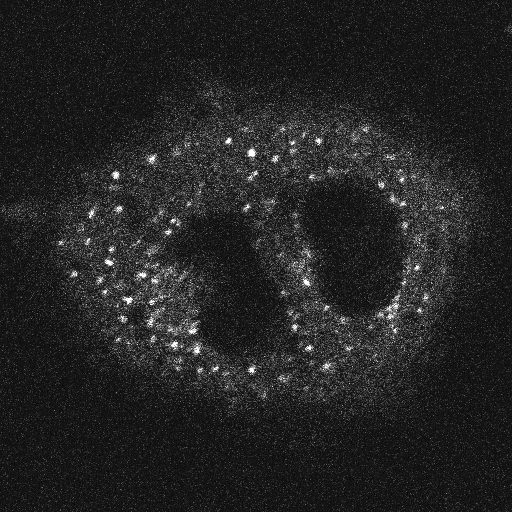

Supplement: Supplementary file 13 — Figure EV4 Source Data [file 44318_2026_754_MOESM13_ESM.zip › EV Figure4/EV 4D/EV4D_image_SEC16B FL_SEC24_Bleach.tif]

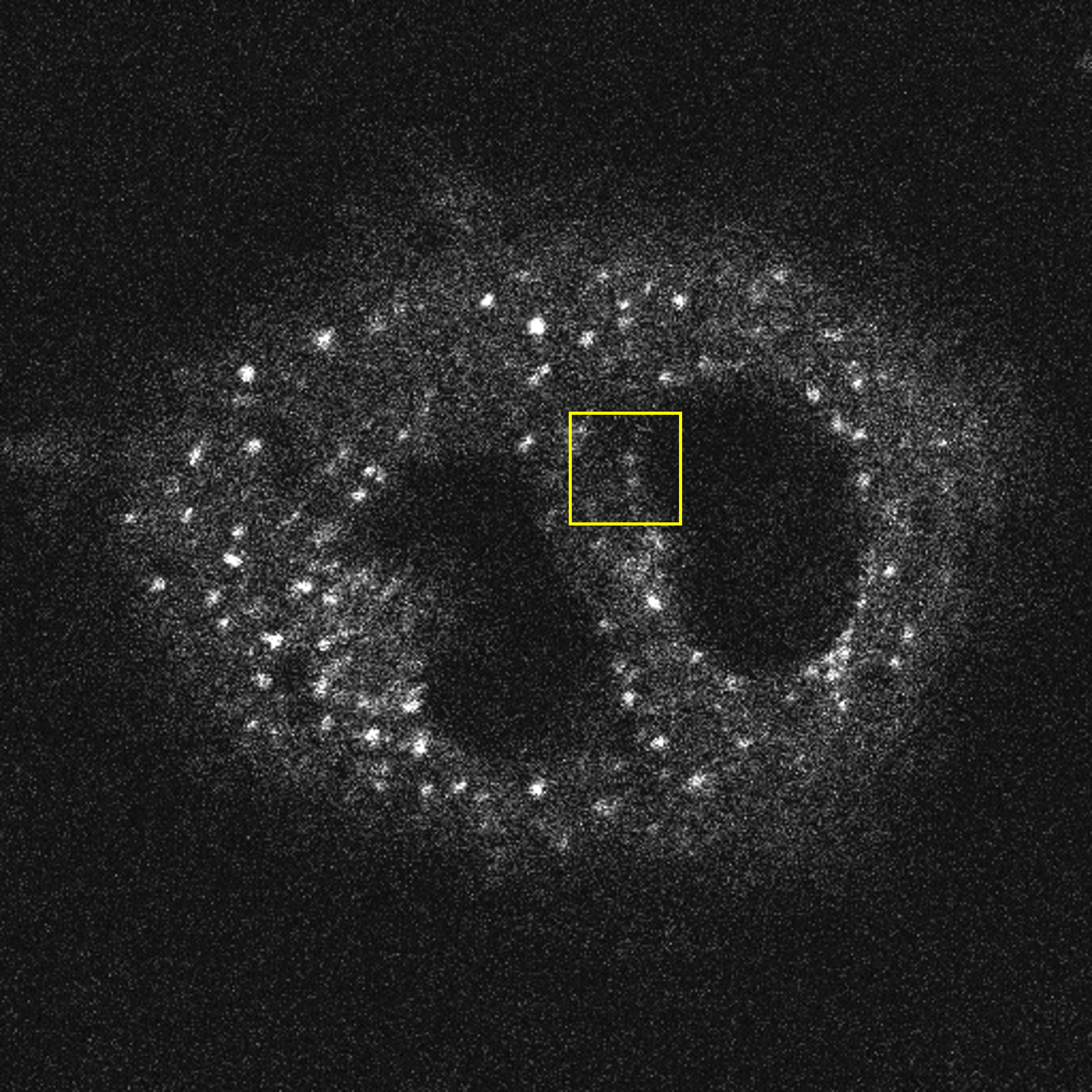

Supplement: Supplementary file 13 — Figure EV4 Source Data [file 44318_2026_754_MOESM13_ESM.zip › EV Figure4/EV 4D/EV4D_image_SEC16B FL_SEC24_Bleach_label.tif]

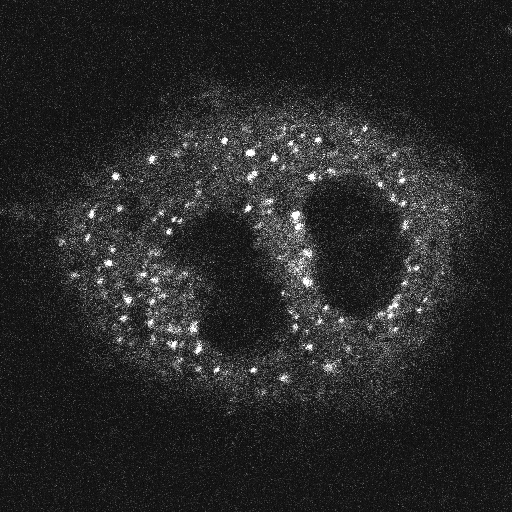

Supplement: Supplementary file 13 — Figure EV4 Source Data [file 44318_2026_754_MOESM13_ESM.zip › EV Figure4/EV 4D/EV4D_image_SEC16B FL_SEC24_pre.tif]

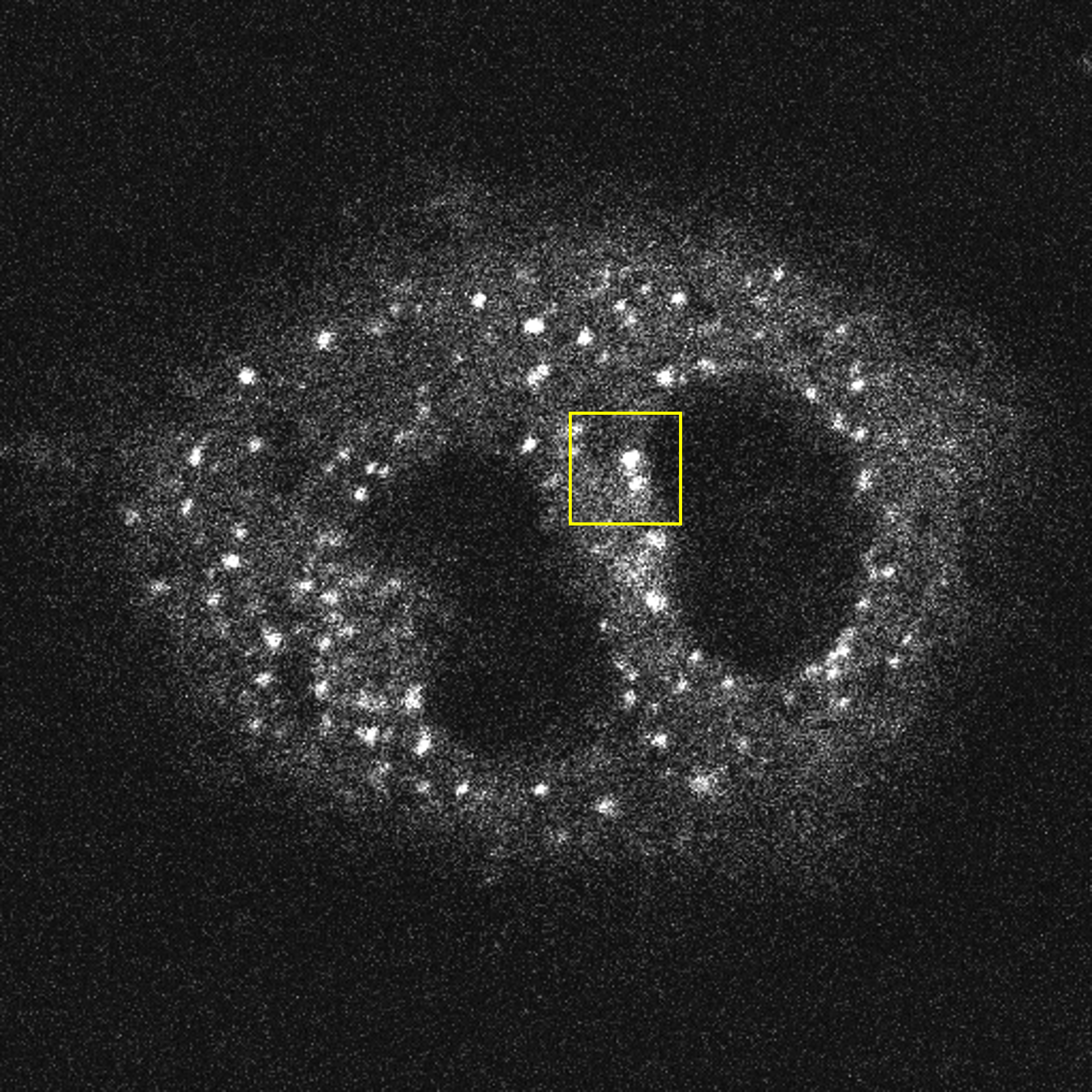

Supplement: Supplementary file 13 — Figure EV4 Source Data [file 44318_2026_754_MOESM13_ESM.zip › EV Figure4/EV 4D/EV4D_image_SEC16B FL_SEC24_pre_label.tif]

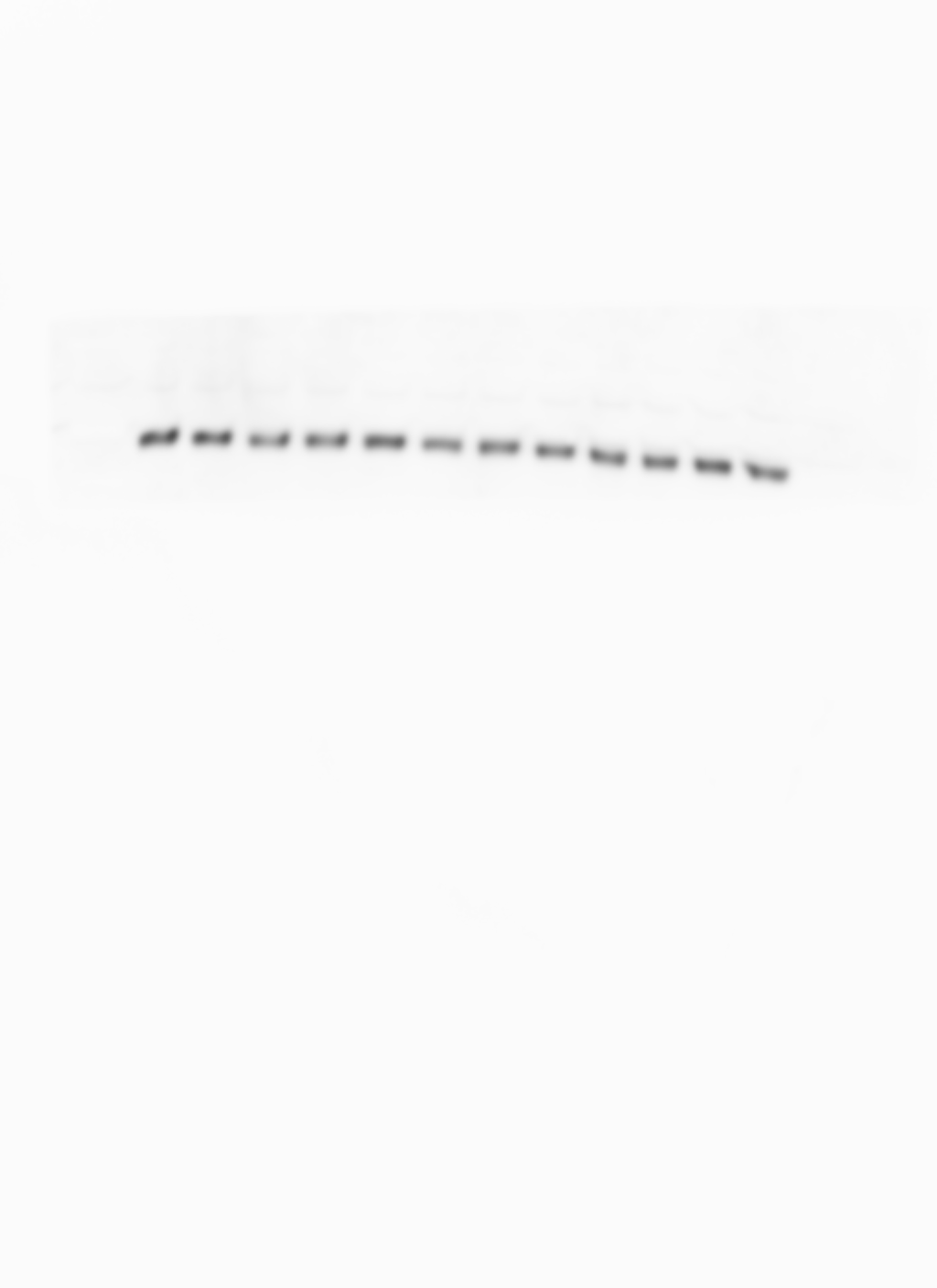

Supplement: Supplementary file 13 — Figure EV4 Source Data [file 44318_2026_754_MOESM13_ESM.zip › EV Figure4/EV 4G/EV4G_western_ALB.tif]

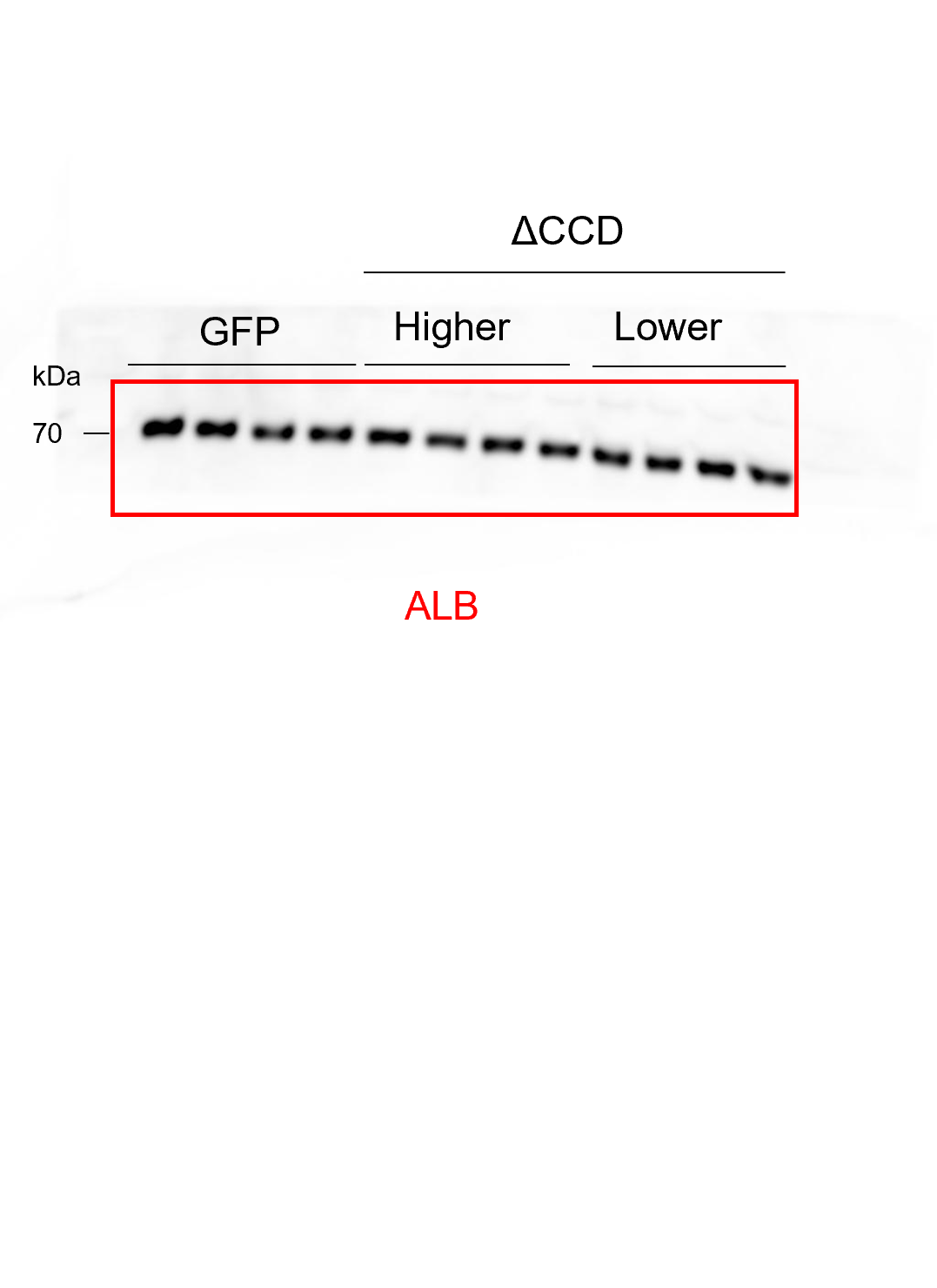

Supplement: Supplementary file 13 — Figure EV4 Source Data [file 44318_2026_754_MOESM13_ESM.zip › EV Figure4/EV 4G/EV4G_western_ALB_label.tif]

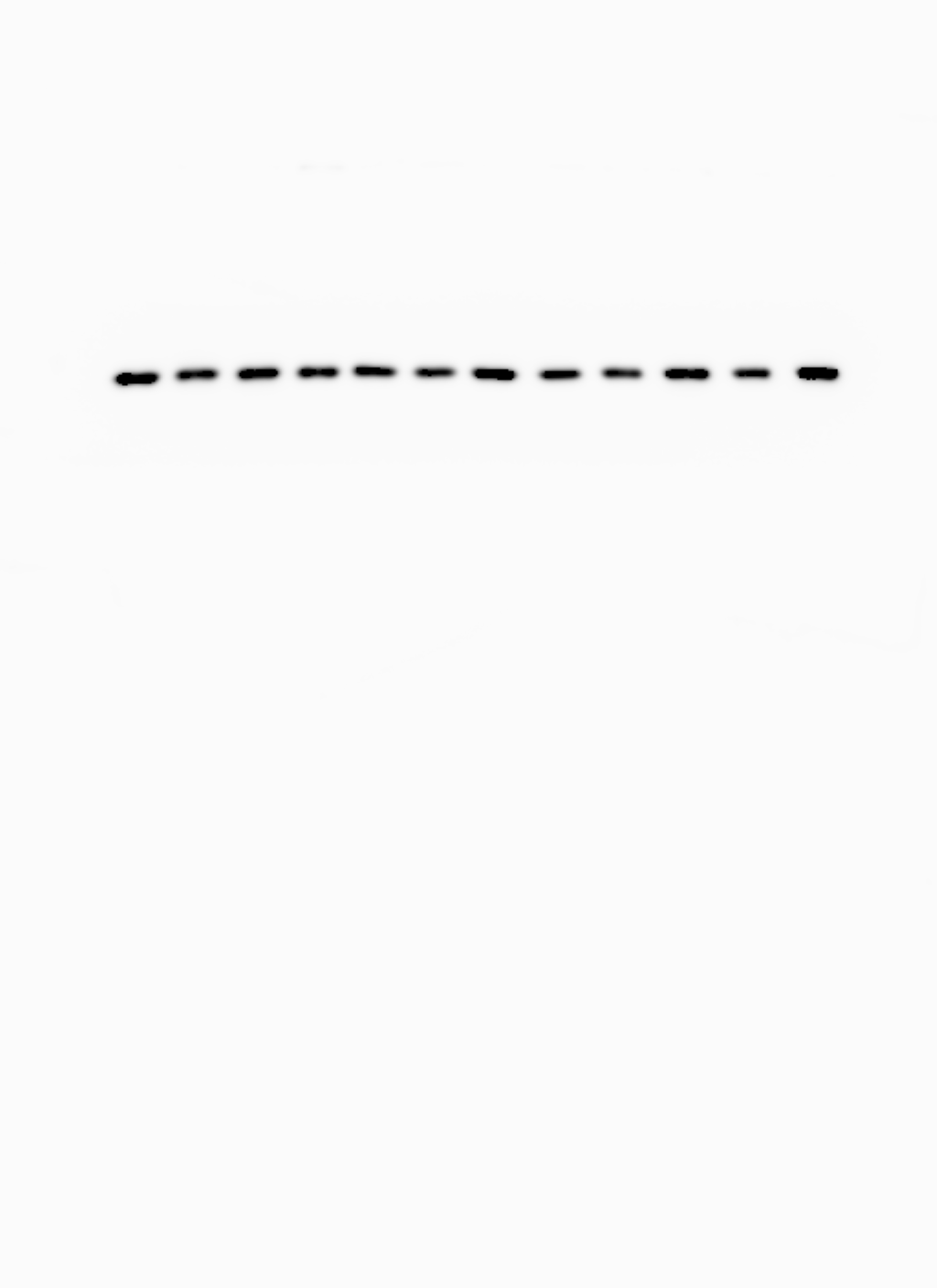

Supplement: Supplementary file 13 — Figure EV4 Source Data [file 44318_2026_754_MOESM13_ESM.zip › EV Figure4/EV 4G/EV4G_western_APOA1.tif]

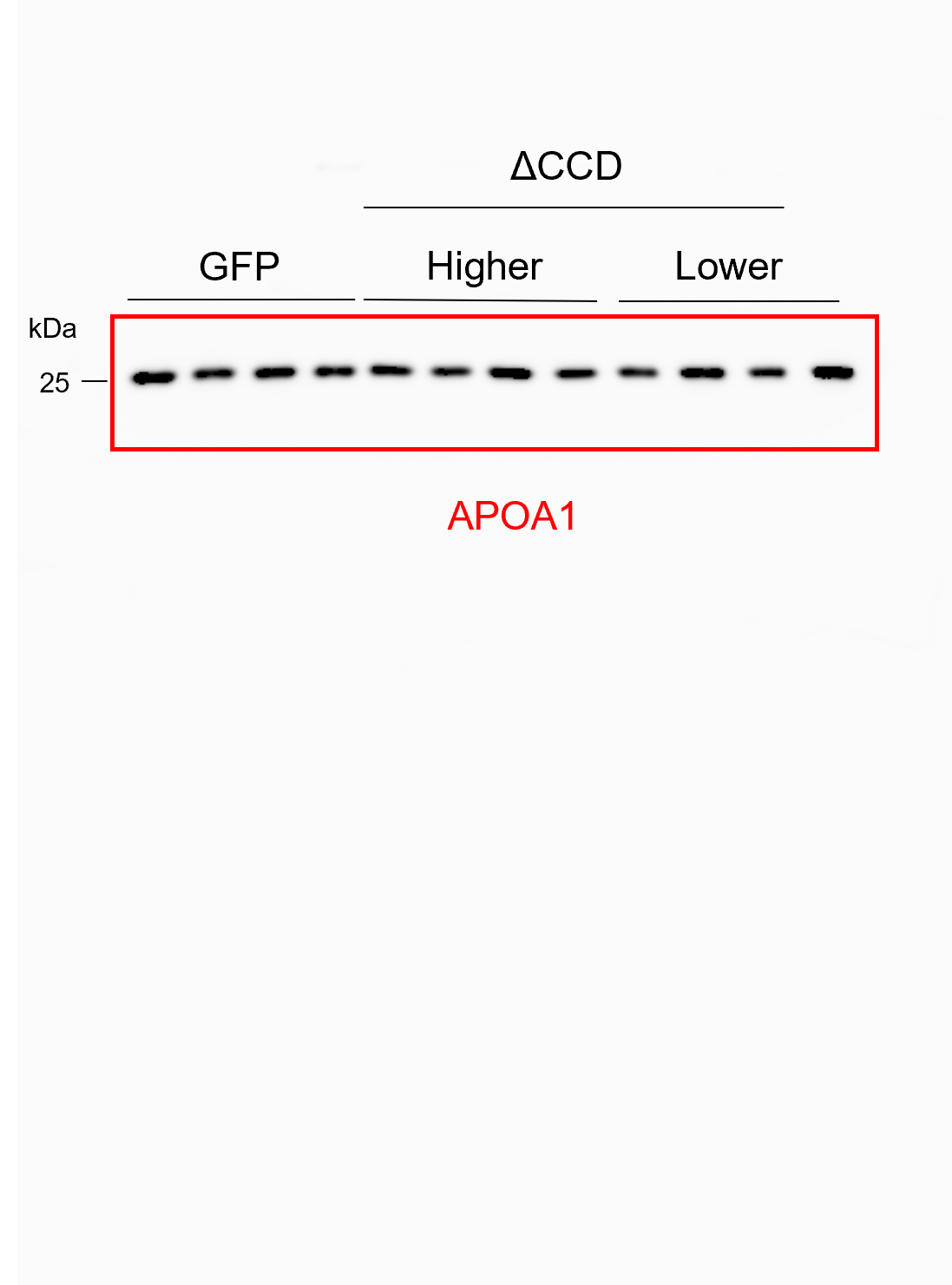

Supplement: Supplementary file 13 — Figure EV4 Source Data [file 44318_2026_754_MOESM13_ESM.zip › EV Figure4/EV 4G/EV4G_western_APOA1_label.tif]

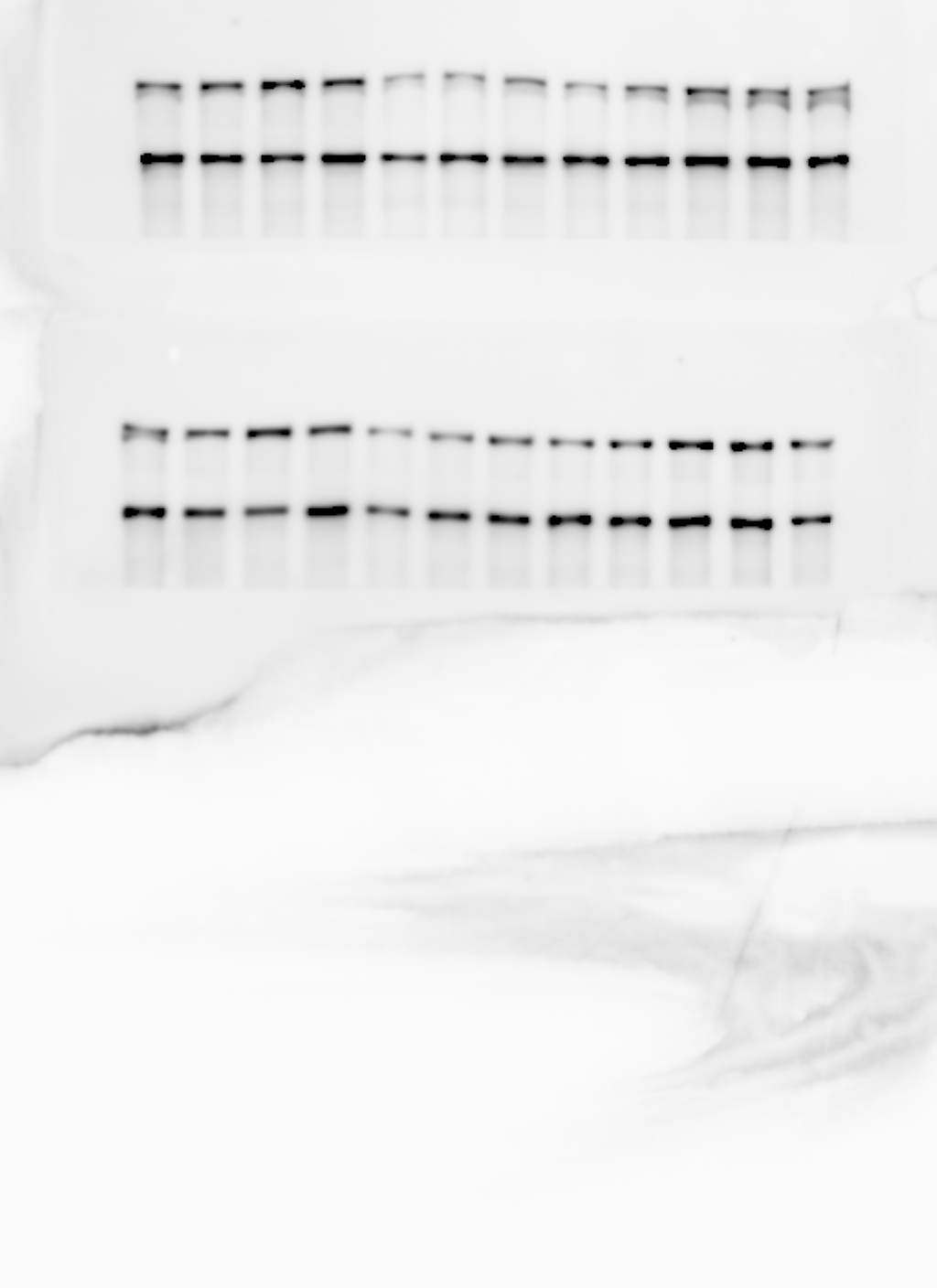

Supplement: Supplementary file 13 — Figure EV4 Source Data [file 44318_2026_754_MOESM13_ESM.zip › EV Figure4/EV 4G/EV4G_western_APOB.tif]

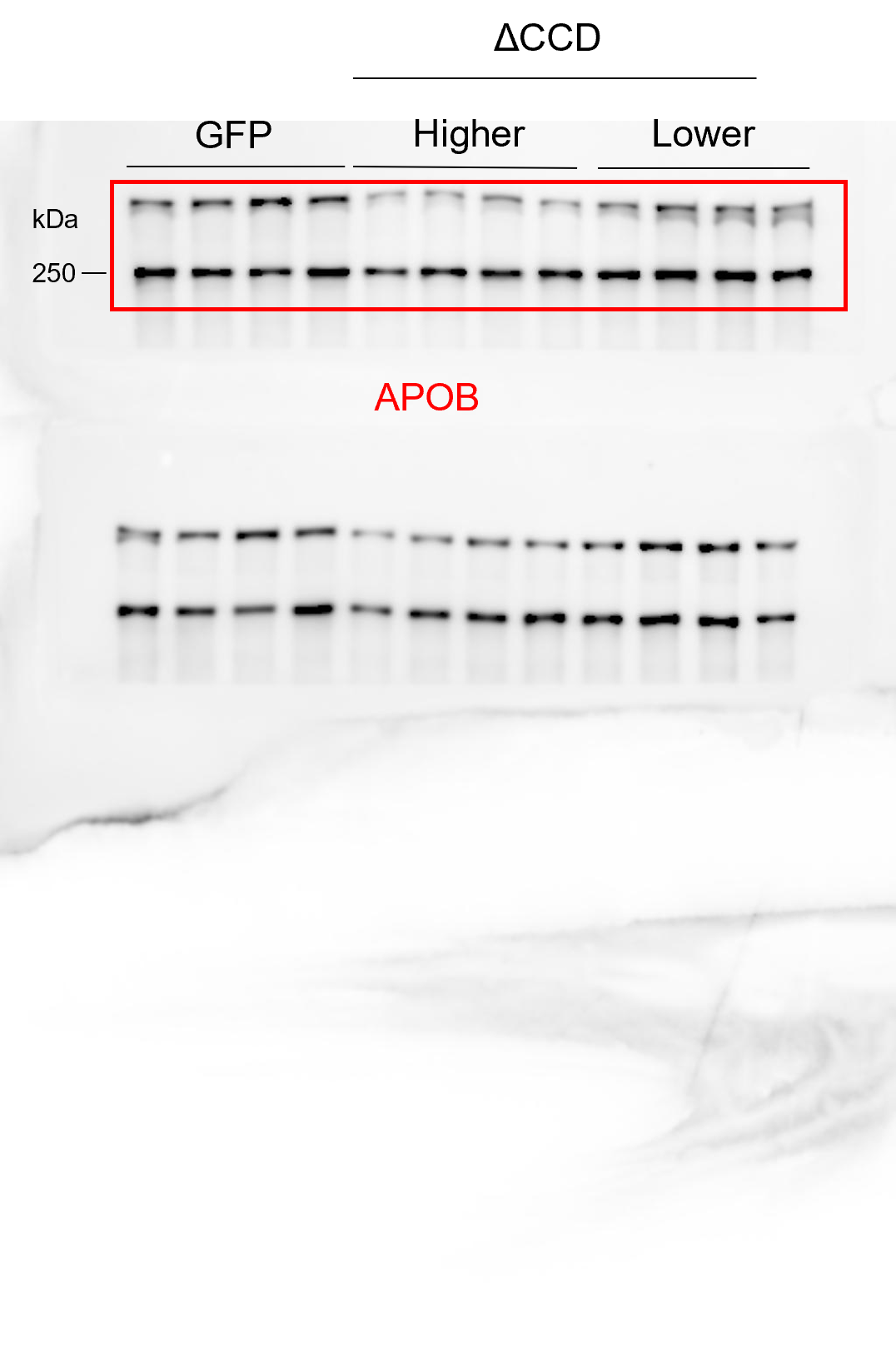

Supplement: Supplementary file 13 — Figure EV4 Source Data [file 44318_2026_754_MOESM13_ESM.zip › EV Figure4/EV 4G/EV4G_western_APOB_label.tif]

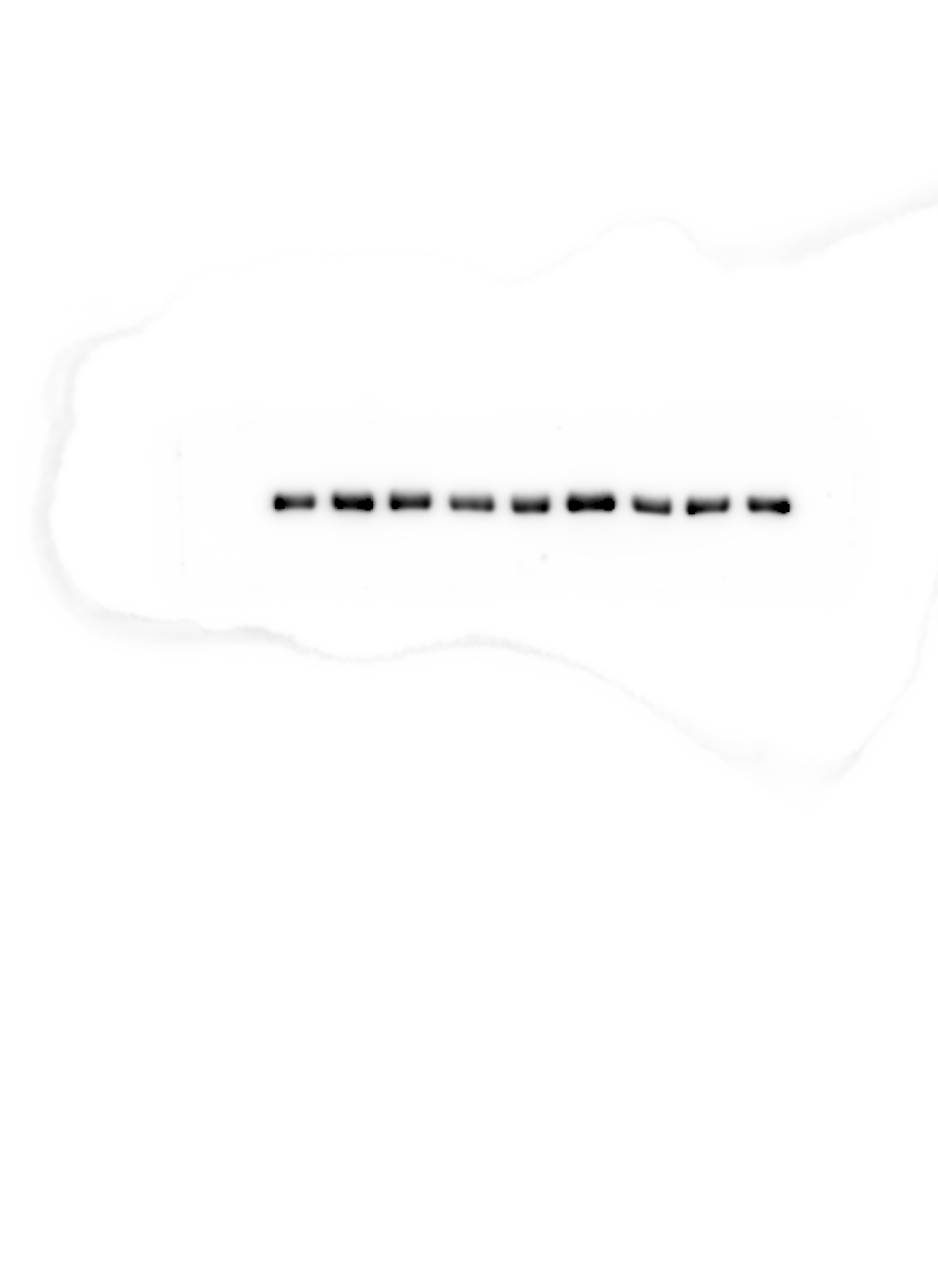

Supplement: Supplementary file 14 — Figure EV5 Source Data [file 44318_2026_754_MOESM14_ESM.zip › EV Figure5/EV 5D/EV5D_western_ALB.tif]

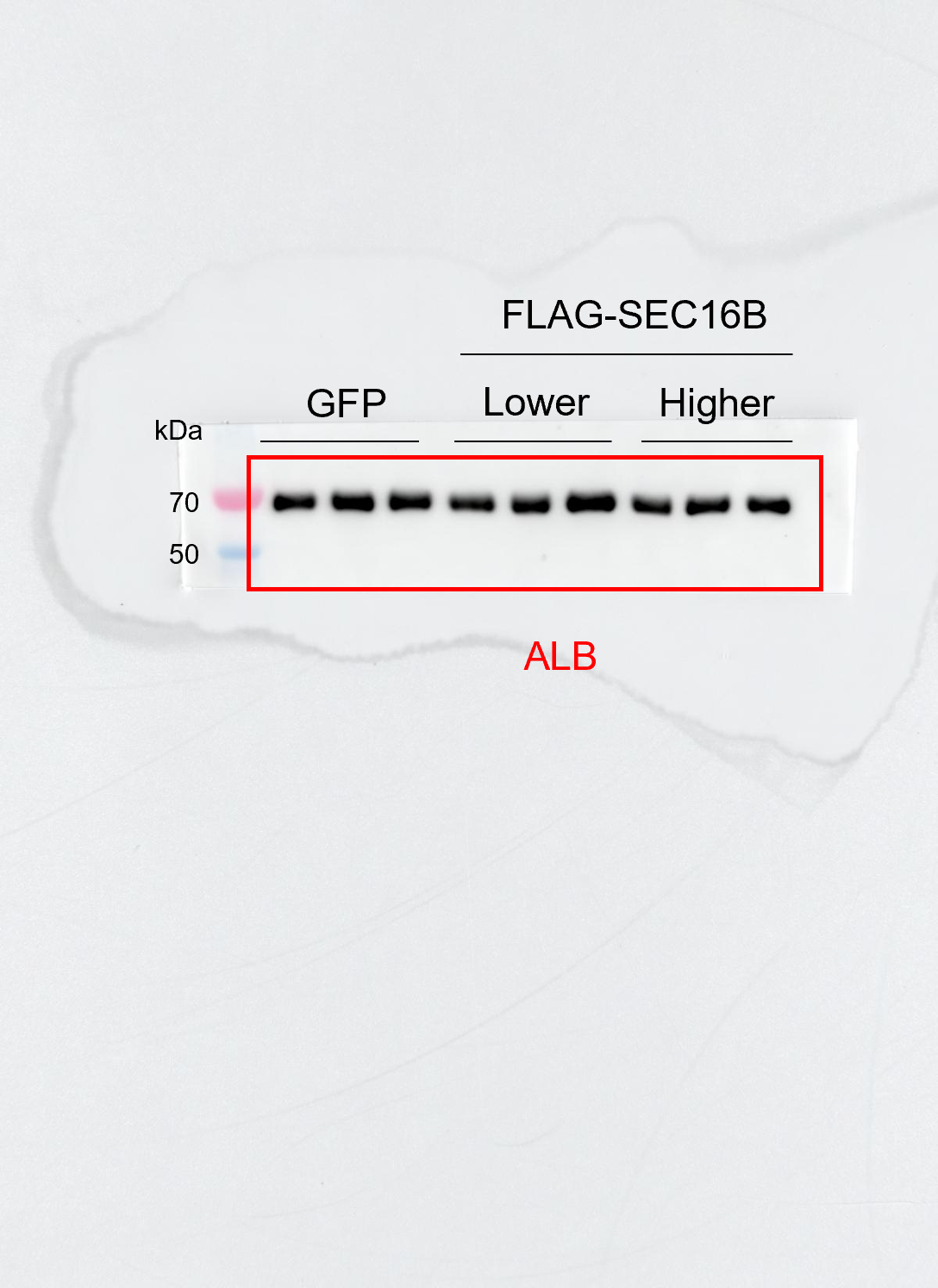

Supplement: Supplementary file 14 — Figure EV5 Source Data [file 44318_2026_754_MOESM14_ESM.zip › EV Figure5/EV 5D/EV5D_western_ALB_label.tif]

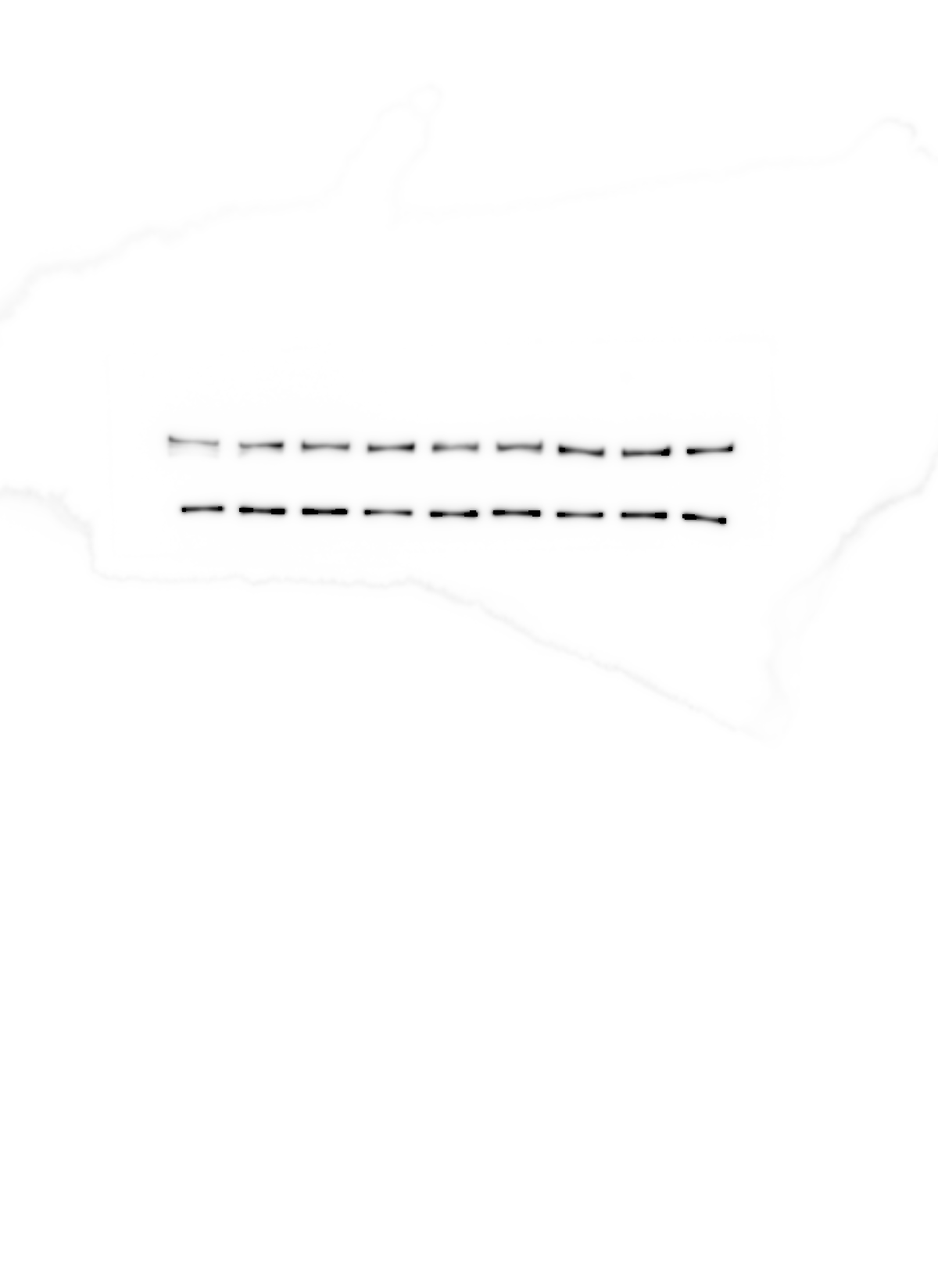

Supplement: Supplementary file 14 — Figure EV5 Source Data [file 44318_2026_754_MOESM14_ESM.zip › EV Figure5/EV 5D/EV5D_western_APOB.tif]

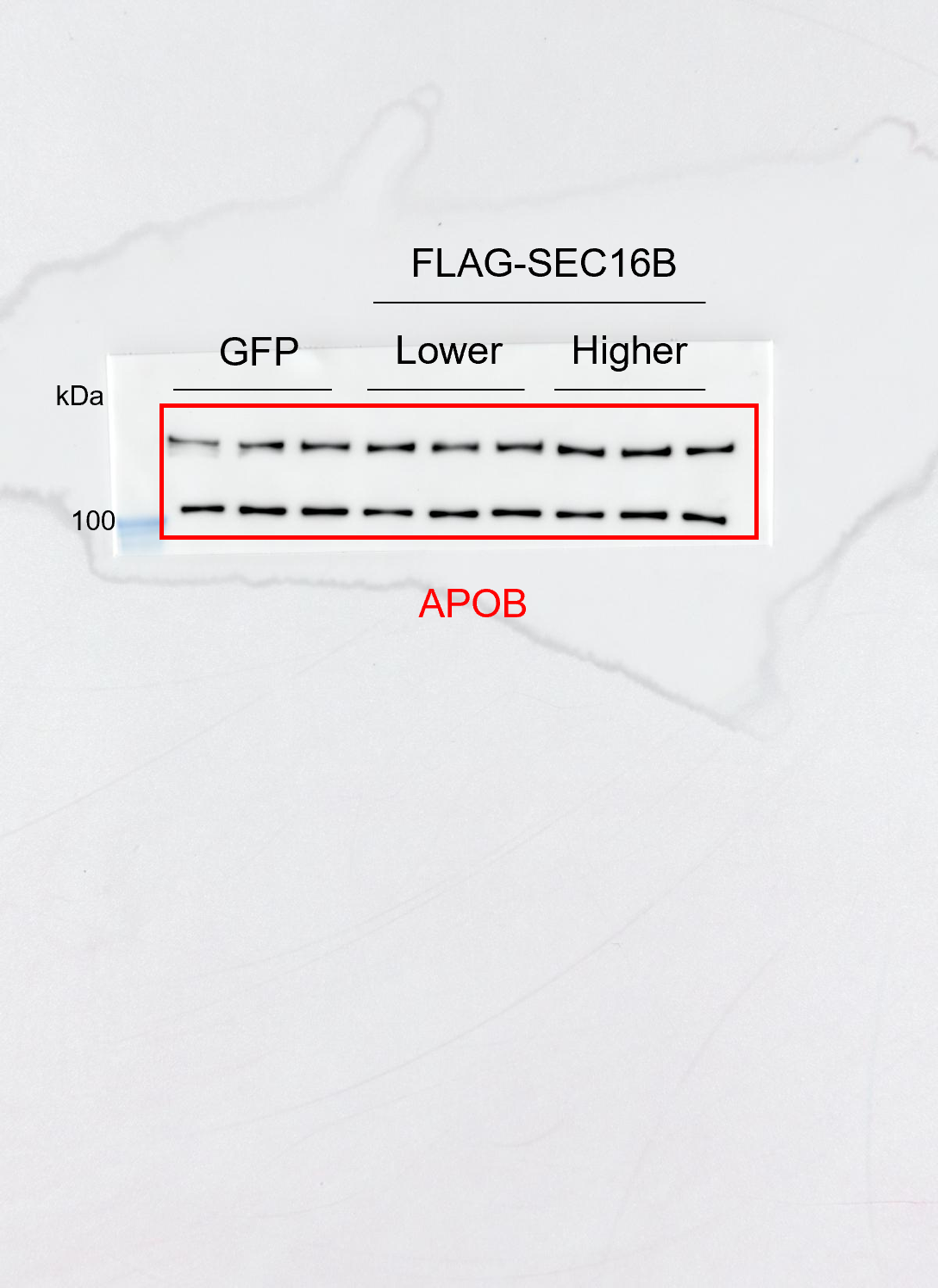

Supplement: Supplementary file 14 — Figure EV5 Source Data [file 44318_2026_754_MOESM14_ESM.zip › EV Figure5/EV 5D/EV5D_western_APOB_label.tif]

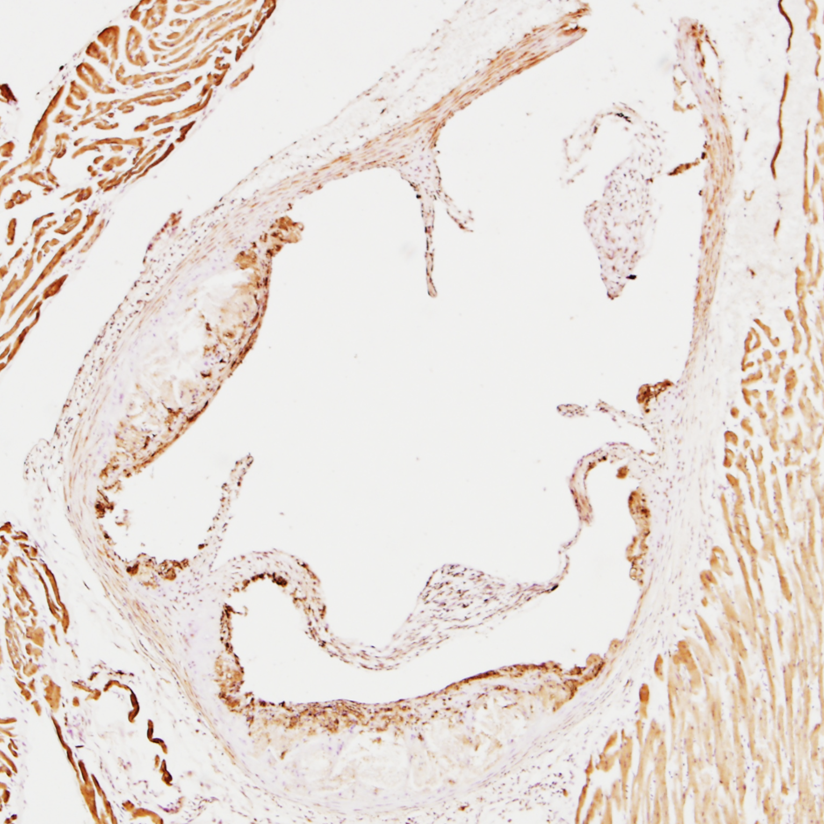

Supplement: Supplementary file 14 — Figure EV5 Source Data [file 44318_2026_754_MOESM14_ESM.zip › EV Figure5/EV 5E/EV5E_CD68_CTL.tif]

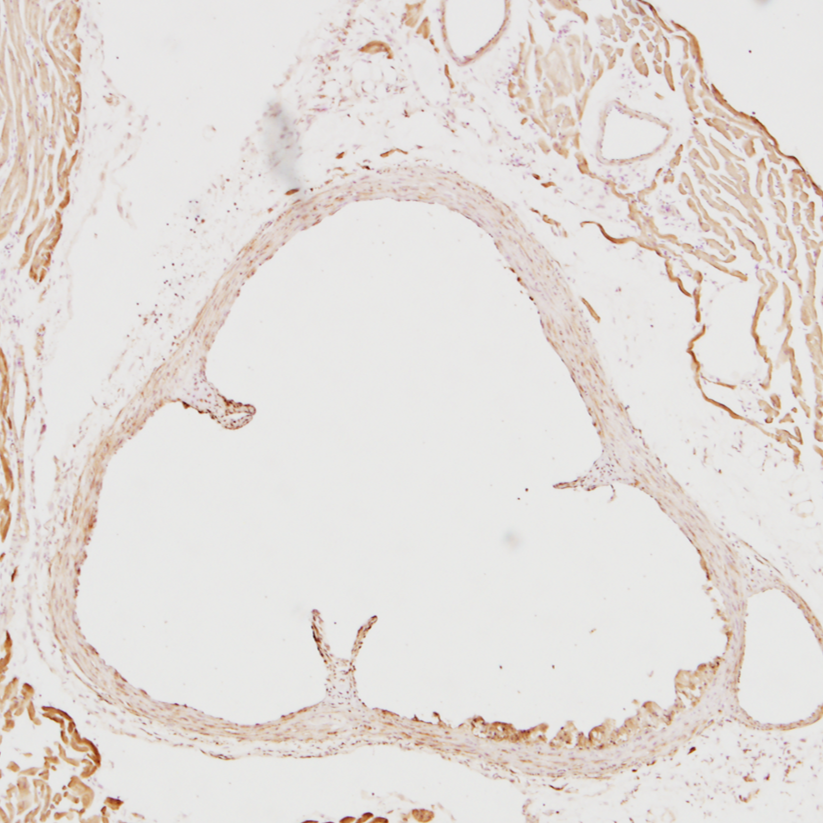

Supplement: Supplementary file 14 — Figure EV5 Source Data [file 44318_2026_754_MOESM14_ESM.zip › EV Figure5/EV 5E/EV5E_CD68_KO.tif]

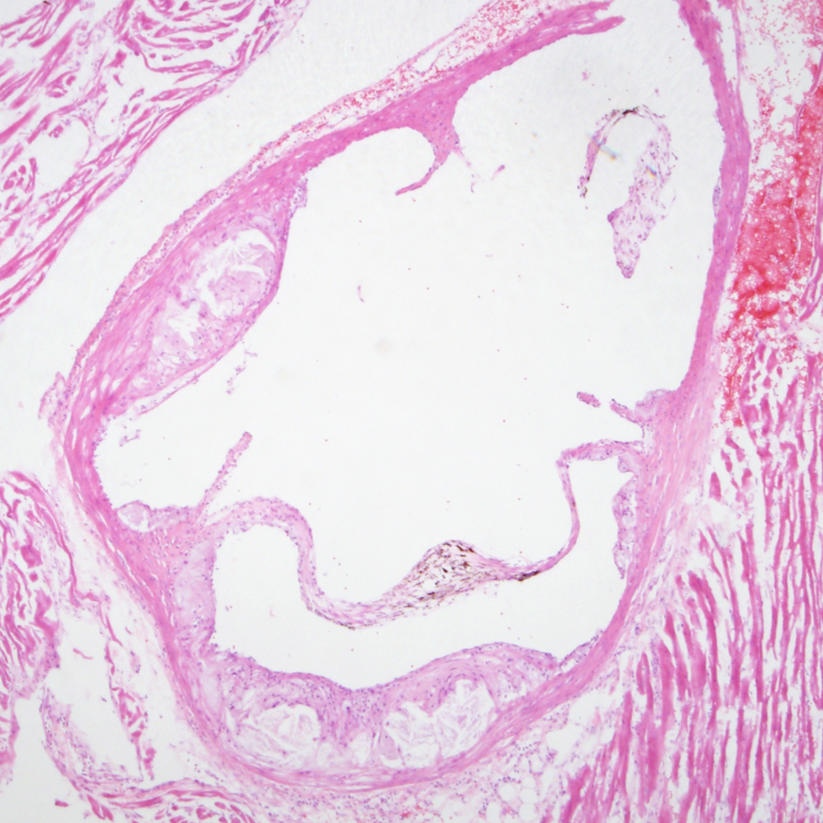

Supplement: Supplementary file 14 — Figure EV5 Source Data [file 44318_2026_754_MOESM14_ESM.zip › EV Figure5/EV 5E/EV5E_H&E_CTL.tif]

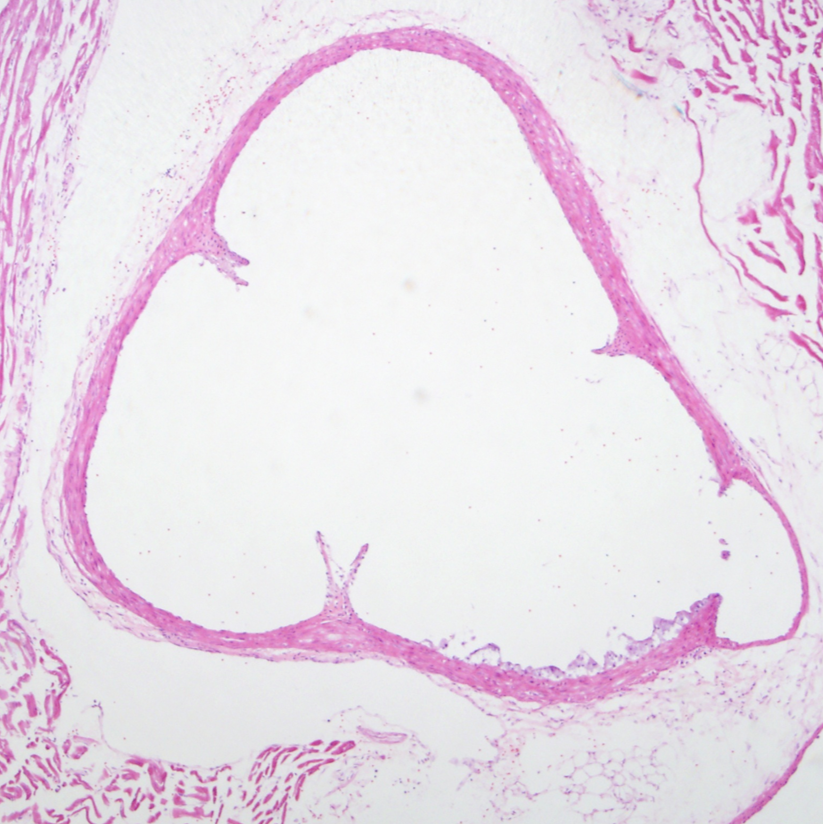

Supplement: Supplementary file 14 — Figure EV5 Source Data [file 44318_2026_754_MOESM14_ESM.zip › EV Figure5/EV 5E/EV5E_H&E_KO.tif]

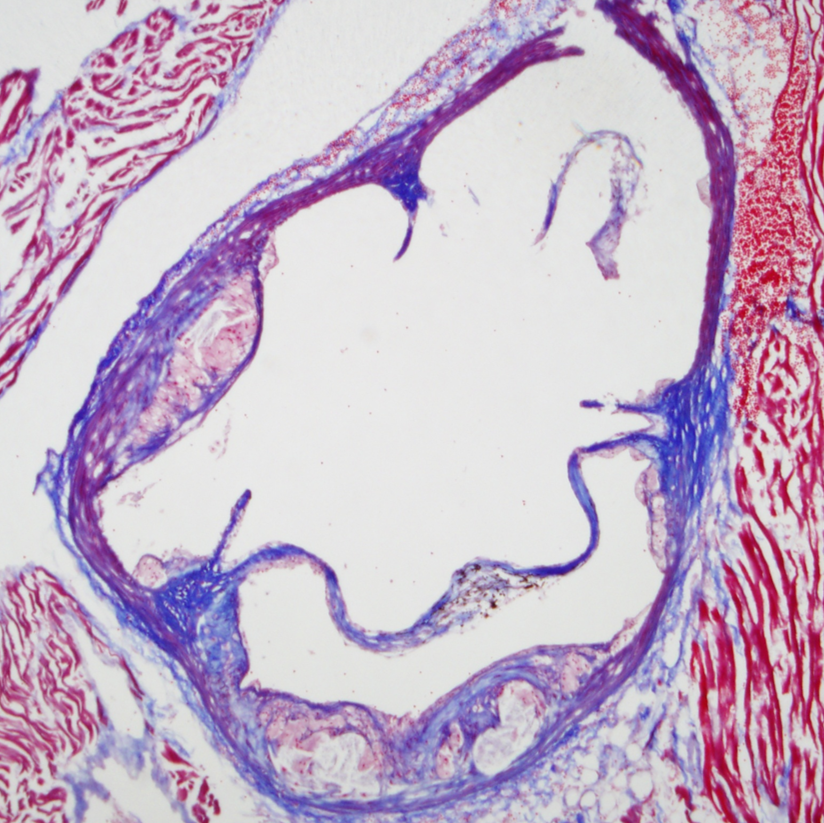

Supplement: Supplementary file 14 — Figure EV5 Source Data [file 44318_2026_754_MOESM14_ESM.zip › EV Figure5/EV 5E/EV5E_Masson_CTL.tif]

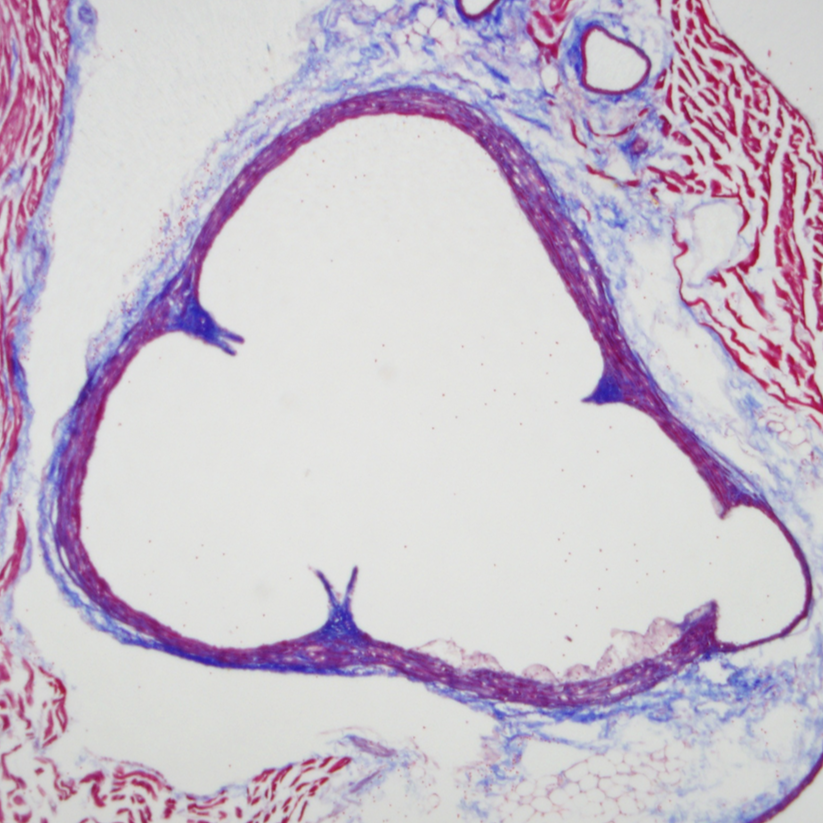

Supplement: Supplementary file 14 — Figure EV5 Source Data [file 44318_2026_754_MOESM14_ESM.zip › EV Figure5/EV 5E/EV5E_Masson_KO.tif]

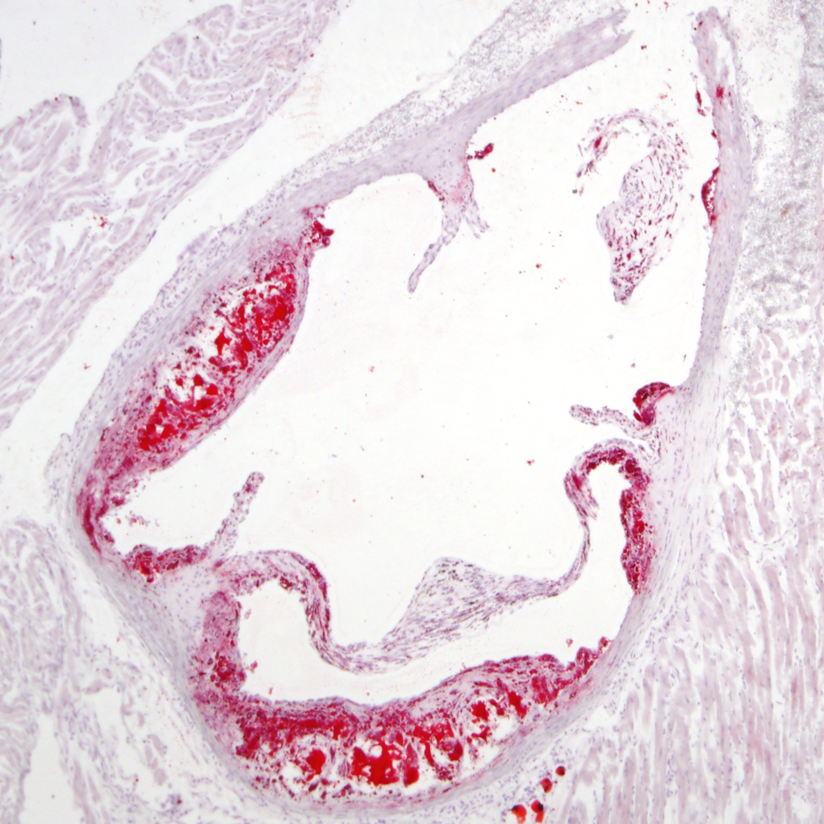

Supplement: Supplementary file 14 — Figure EV5 Source Data [file 44318_2026_754_MOESM14_ESM.zip › EV Figure5/EV 5E/EV5E_ORO_CTL.tif]

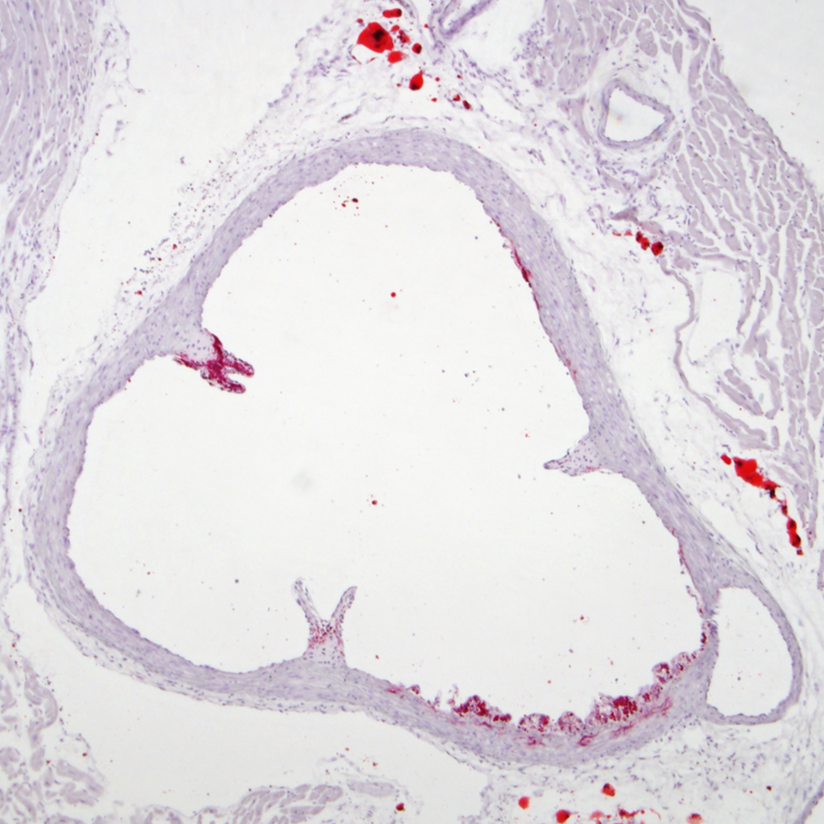

Supplement: Supplementary file 14 — Figure EV5 Source Data [file 44318_2026_754_MOESM14_ESM.zip › EV Figure5/EV 5E/EV5E_ORO_KO.tif]

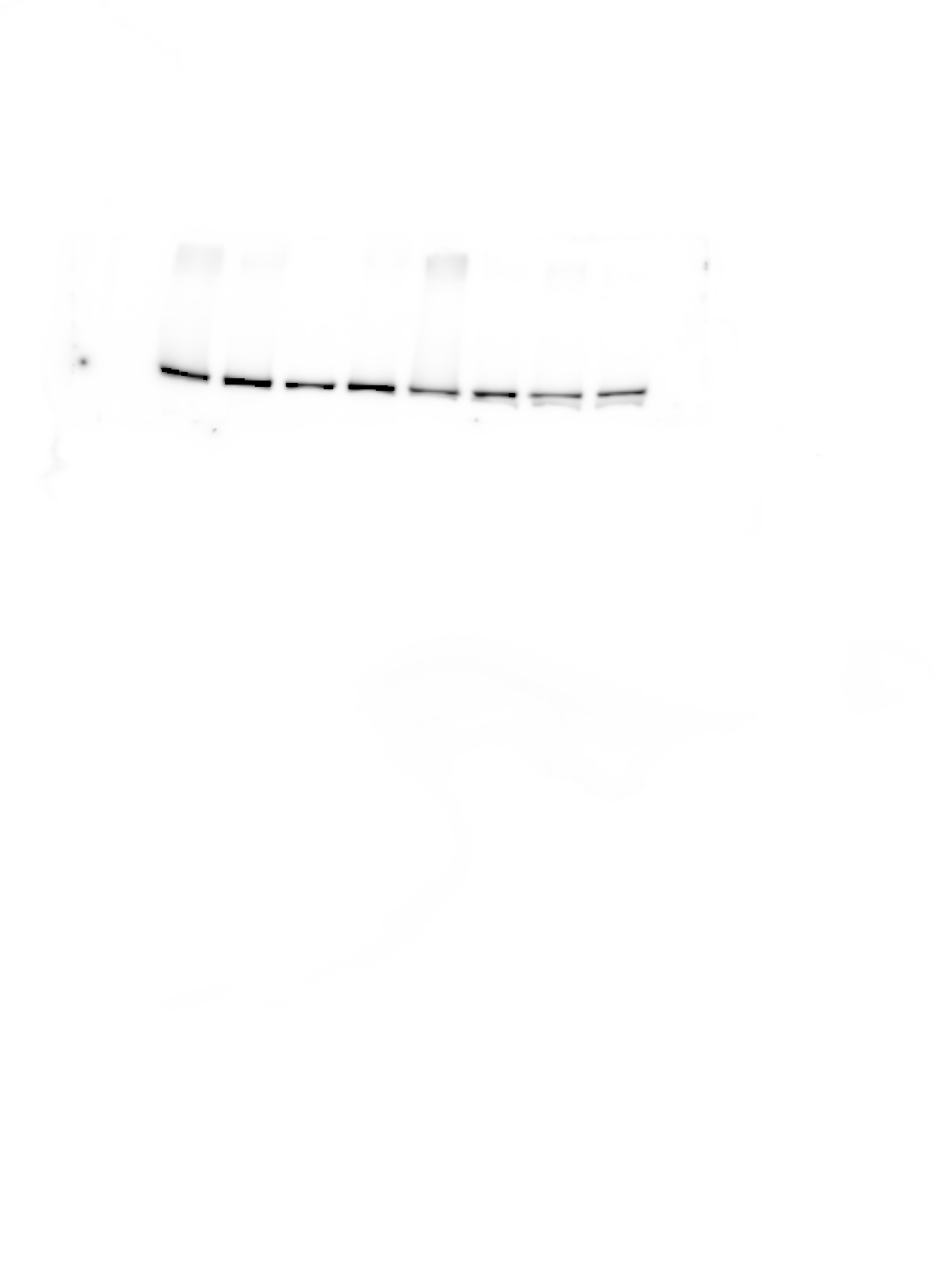

Supplement: Supplementary file 14 — Figure EV5 Source Data [file 44318_2026_754_MOESM14_ESM.zip › EV Figure5/EV 5H/EV5H_western_ACC.tif]

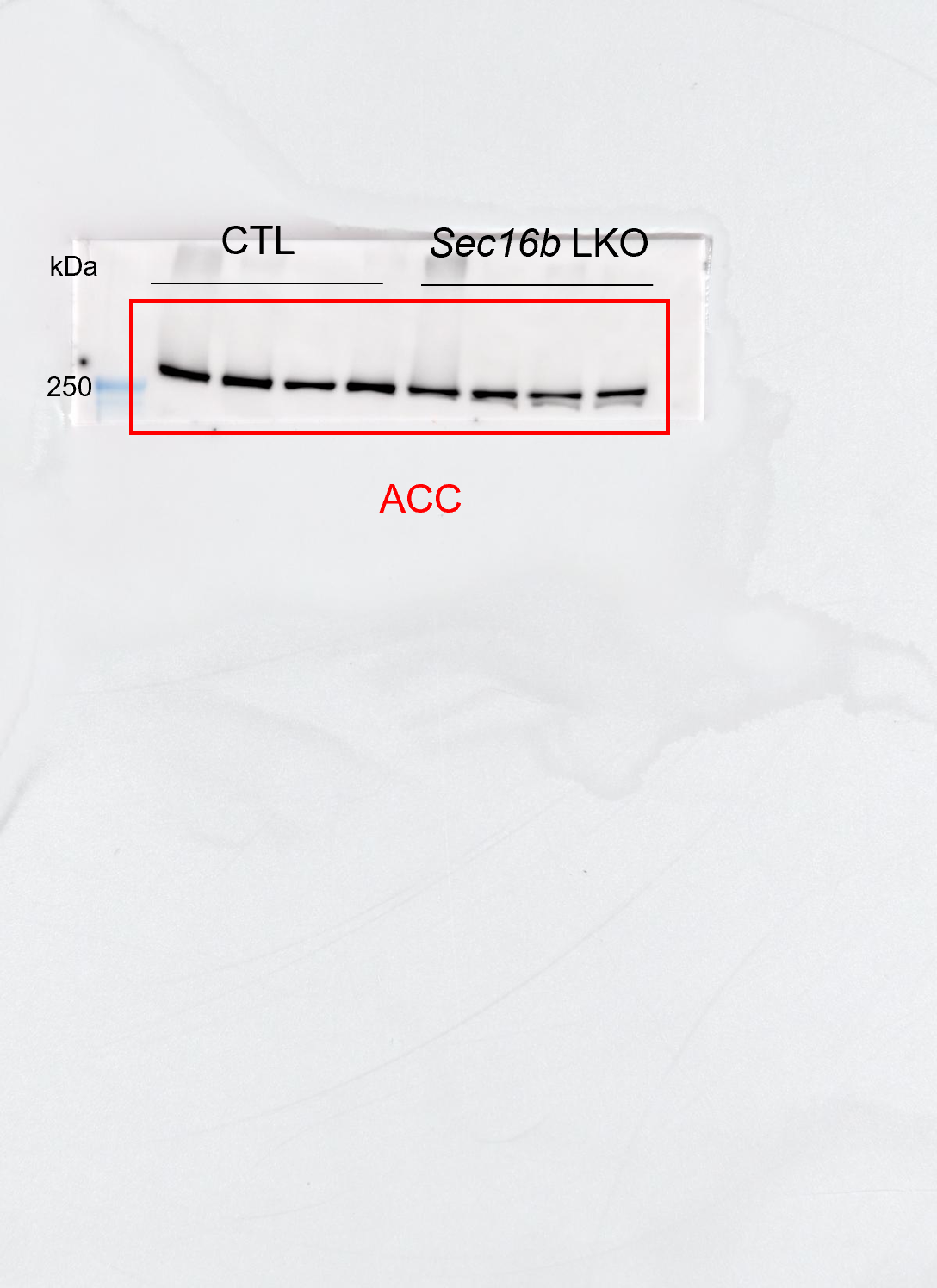

Supplement: Supplementary file 14 — Figure EV5 Source Data [file 44318_2026_754_MOESM14_ESM.zip › EV Figure5/EV 5H/EV5H_western_ACC_label.tif]

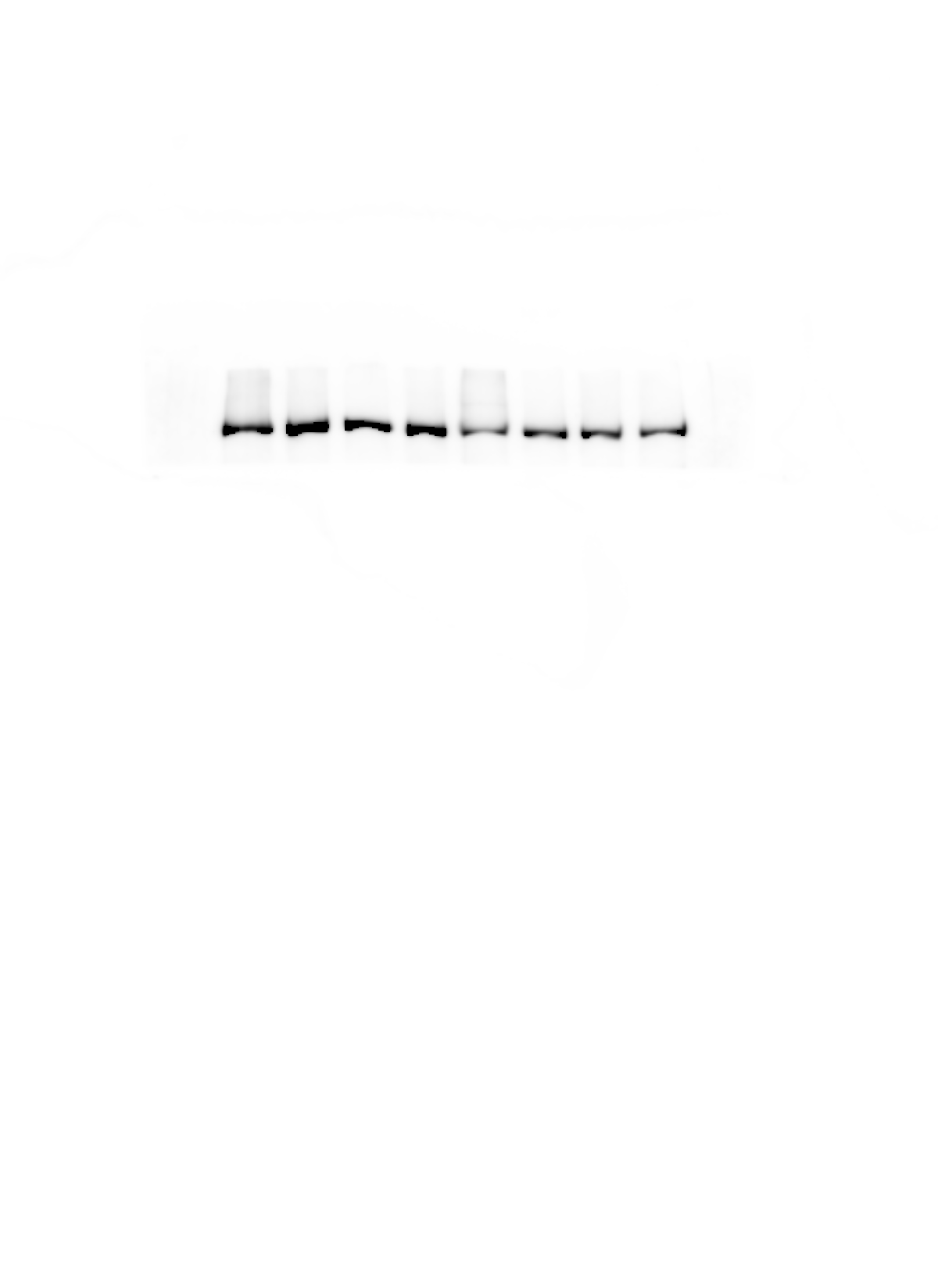

Supplement: Supplementary file 14 — Figure EV5 Source Data [file 44318_2026_754_MOESM14_ESM.zip › EV Figure5/EV 5H/EV5H_western_FASN.tif]

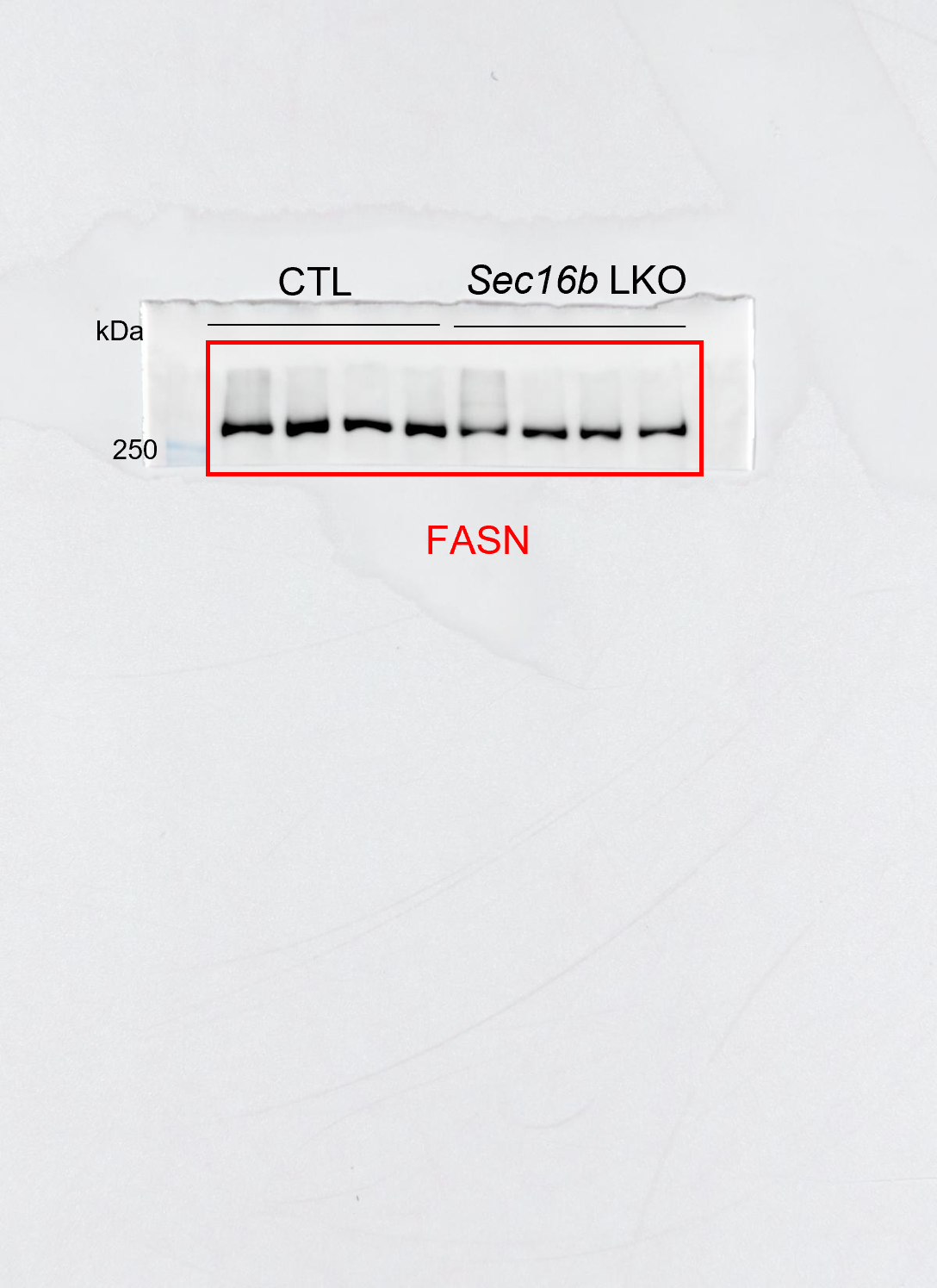

Supplement: Supplementary file 14 — Figure EV5 Source Data [file 44318_2026_754_MOESM14_ESM.zip › EV Figure5/EV 5H/EV5H_western_FASN_label.tif]

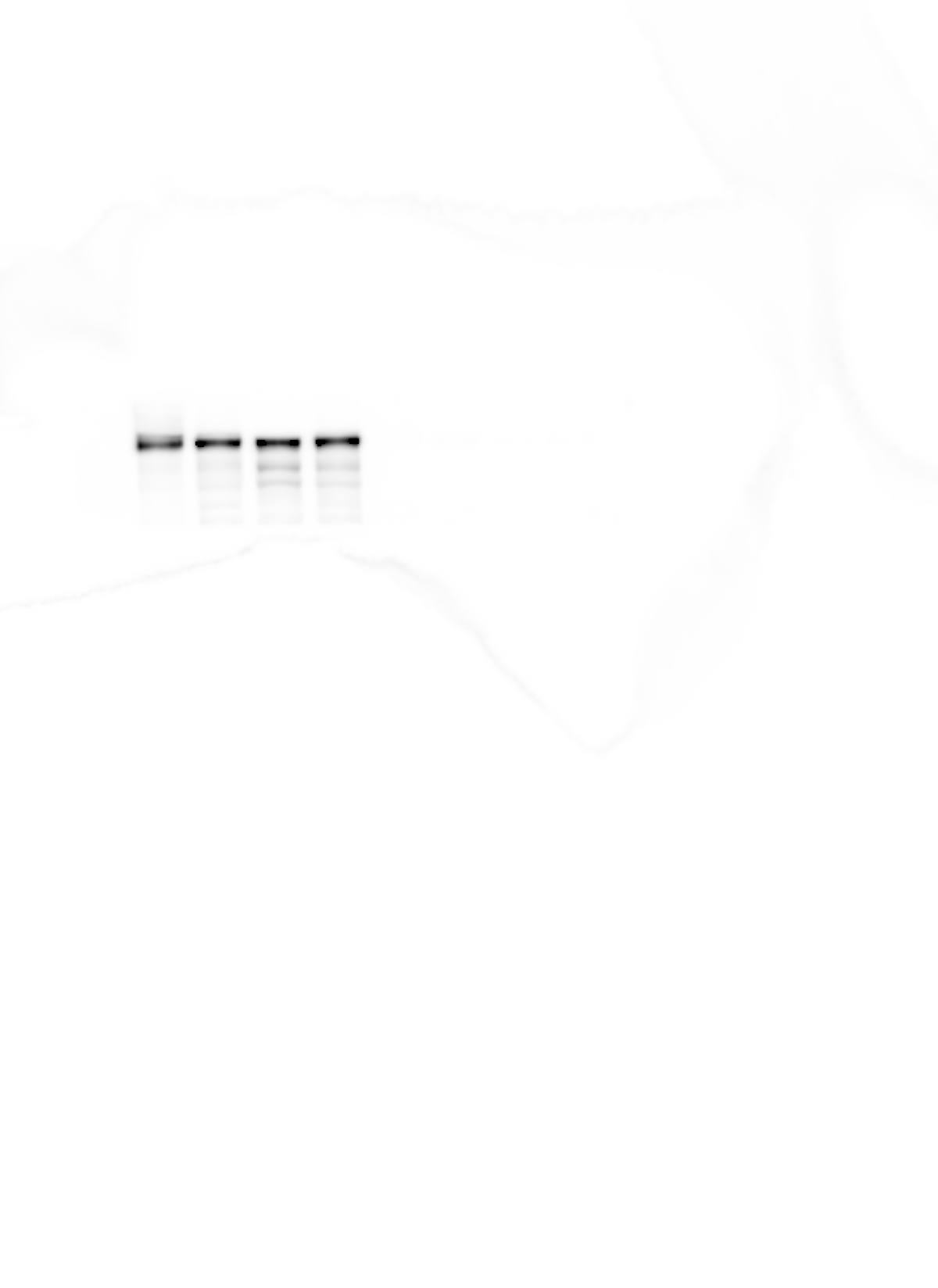

Supplement: Supplementary file 14 — Figure EV5 Source Data [file 44318_2026_754_MOESM14_ESM.zip › EV Figure5/EV 5H/EV5H_western_SEC16B.tif]

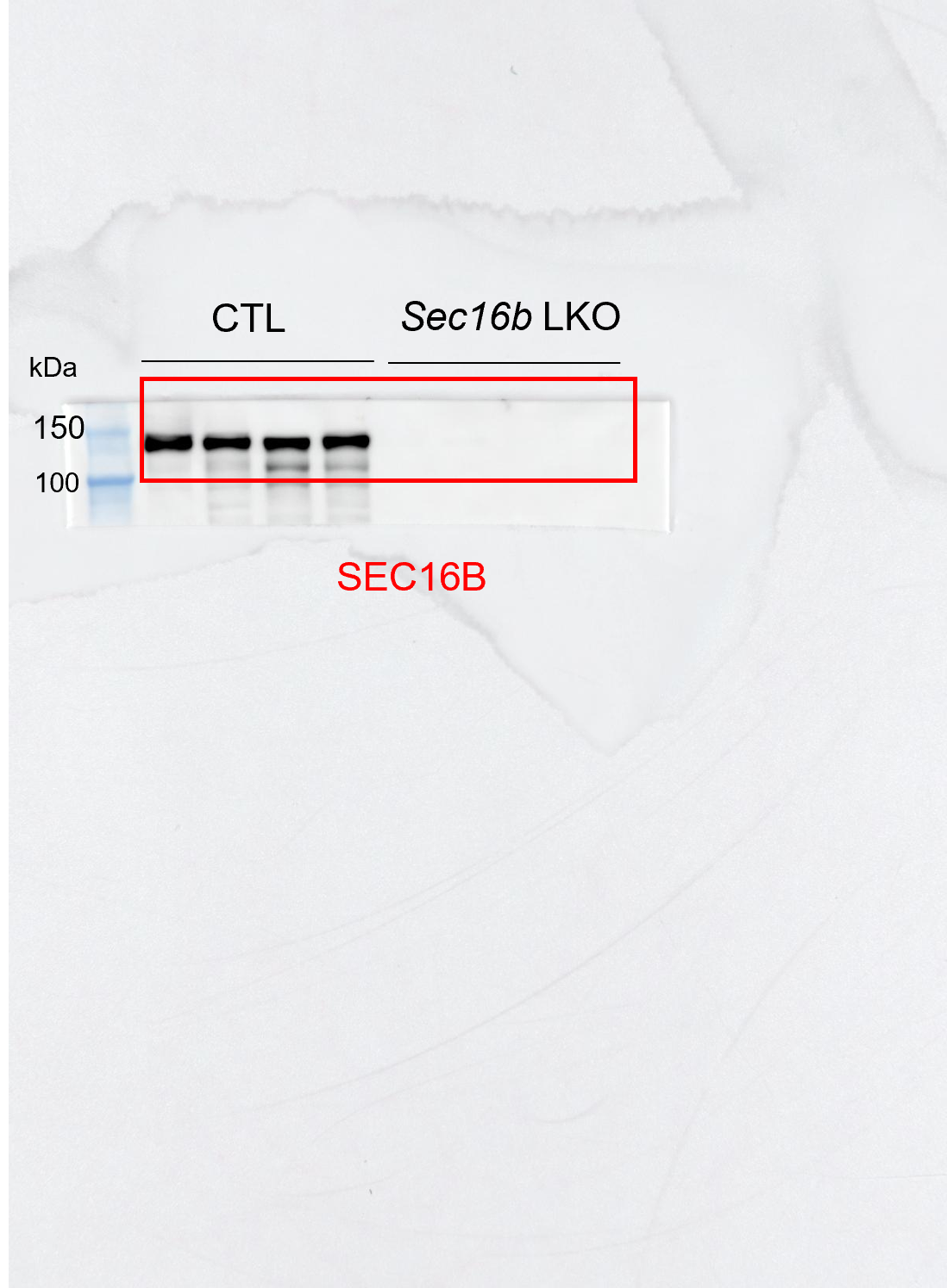

Supplement: Supplementary file 14 — Figure EV5 Source Data [file 44318_2026_754_MOESM14_ESM.zip › EV Figure5/EV 5H/EV5H_western_SEC16B_label.tif]

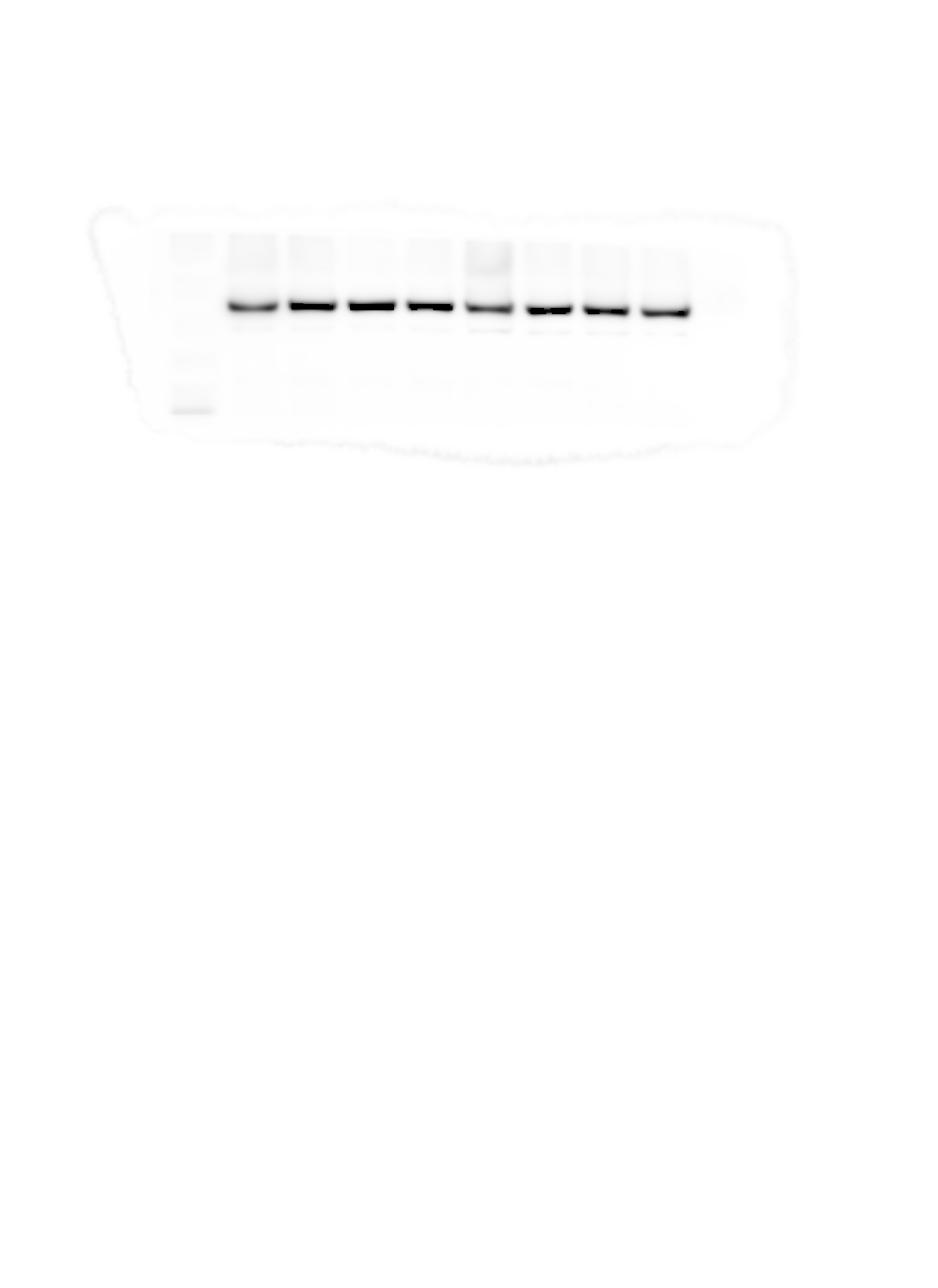

Supplement: Supplementary file 14 — Figure EV5 Source Data [file 44318_2026_754_MOESM14_ESM.zip › EV Figure5/EV 5H/EV5H_western_Tub.tif]

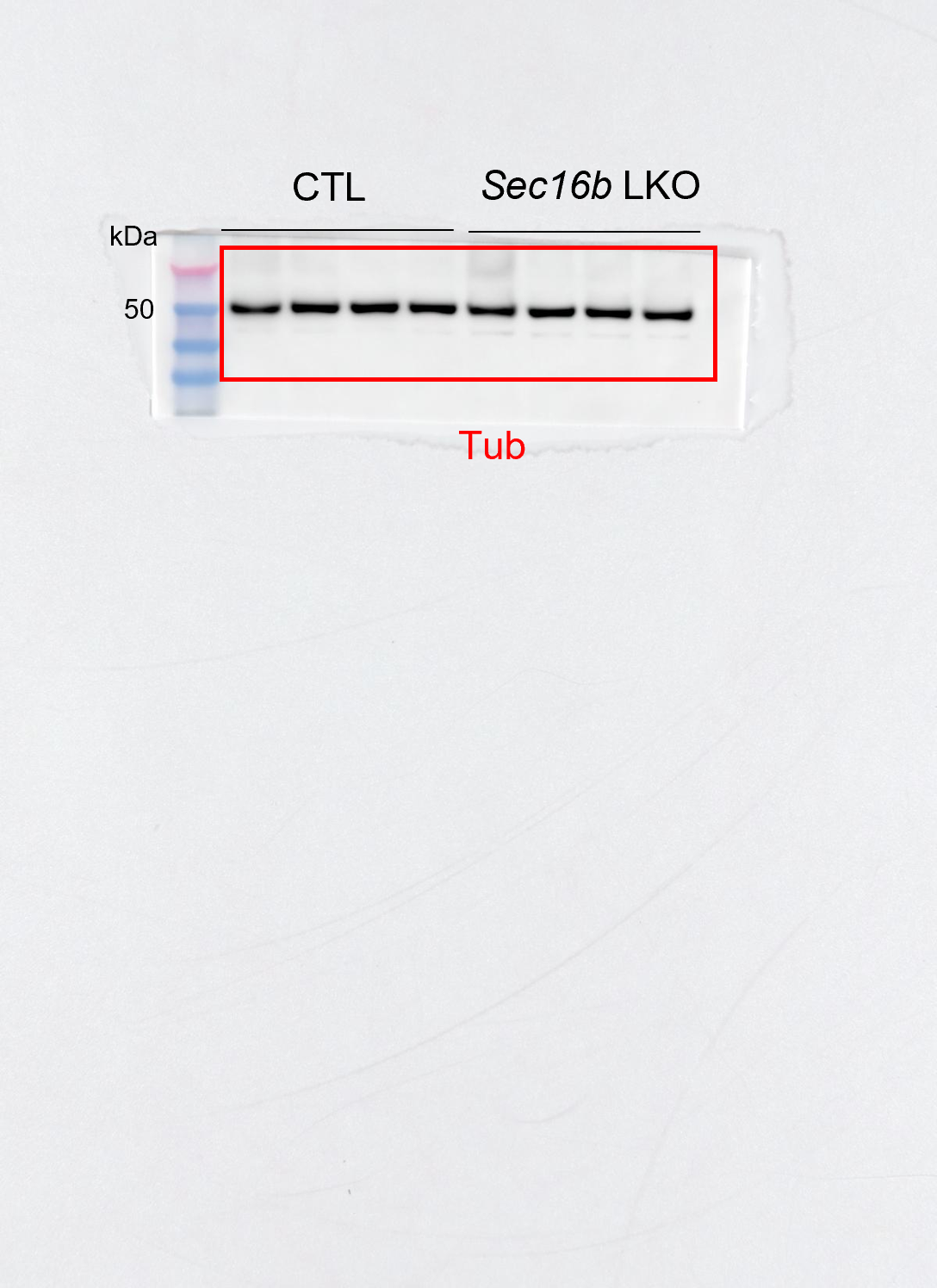

Supplement: Supplementary file 14 — Figure EV5 Source Data [file 44318_2026_754_MOESM14_ESM.zip › EV Figure5/EV 5H/EV5H_western_Tub_label.tif]
