# Supplementary material for: Tissue-selective COPII modulator SEC16B aggravates cardiovascular disease by promoting lipid export
Source: EMBO J. 2026 Apr 24;45(11):3731–62. doi: 10.1038/s44318-026-00754-8 (PMC13226660; doi:10.1038/s44318-026-00754-8)
Supplement: Supplementary file 15 — Expanded View Figures [file 44318_2026_754_MOESM15_ESM.pdf]

Expanded View Figures

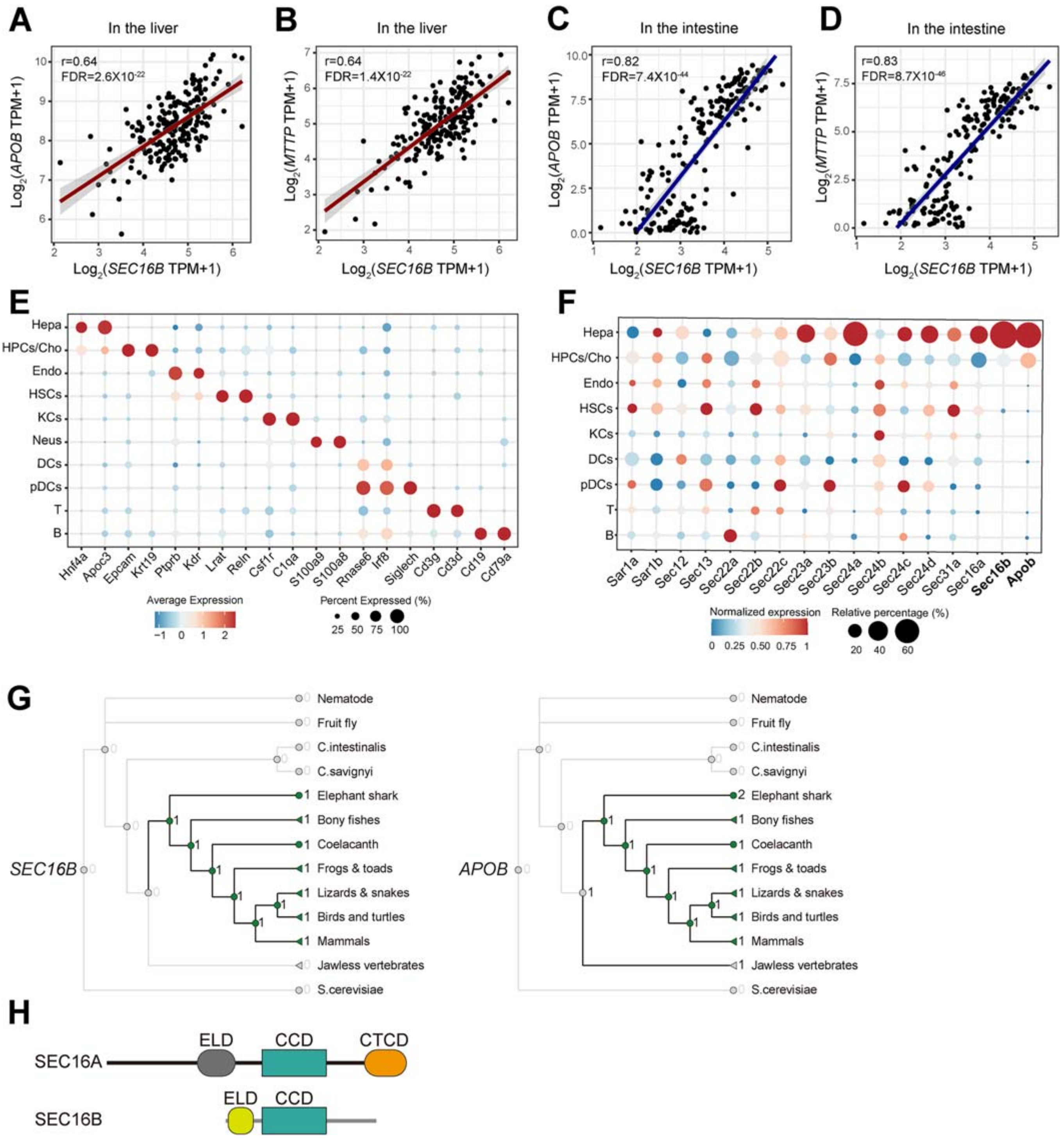

Figure EV1. Identification of a tissue-selective *SEC* gene *SEC16B*.

(**A**, **B**) Correlation analysis of *SEC16B* with *APOB* expression (**A**) and *MTTP* expression (**B**) in human liver. (**C**, **D**) Correlation analysis of *SEC16B* with *APOB* expression (**C**) and *MTTP* expression (**D**) in human intestine. (**E**) Bubble heatmap of marker gene expression for each identified cell cluster in Fig. 1F. Hepa hepatocytes, hPCS/Cho hepatic progenitor cells/cholangiocyte, Endo endothelial cells, HSCs hepatic stellate cells, KCs kupffer cells, Neus neutrophils, DCs dendritic cells, pDCs plasmacytoid dcs, T T cells, B B cells. (**F**) Bubble heatmap of *Sec* gene expression patterns across cell types from Fig. 1F. Sizes represent the percentage of cells expressing the indicated gene within each cluster. Color represents the normalized expression levels in each cell cluster. The expression of *Sec31b* was not detected. Neutrophils were excluded from the analysis due to low cell counts ($n < 10$) to avoid statistical bias. (**G**) Gene gain and loss events of *SEC16B* and *APOB* by using the Ensembl browser. (**H**) Domain schematics of SEC16A and SEC16B. ELD ER localizing domain, CCD central conserved domain, CTCD C-terminal conserved domain.

 © The Author(s)

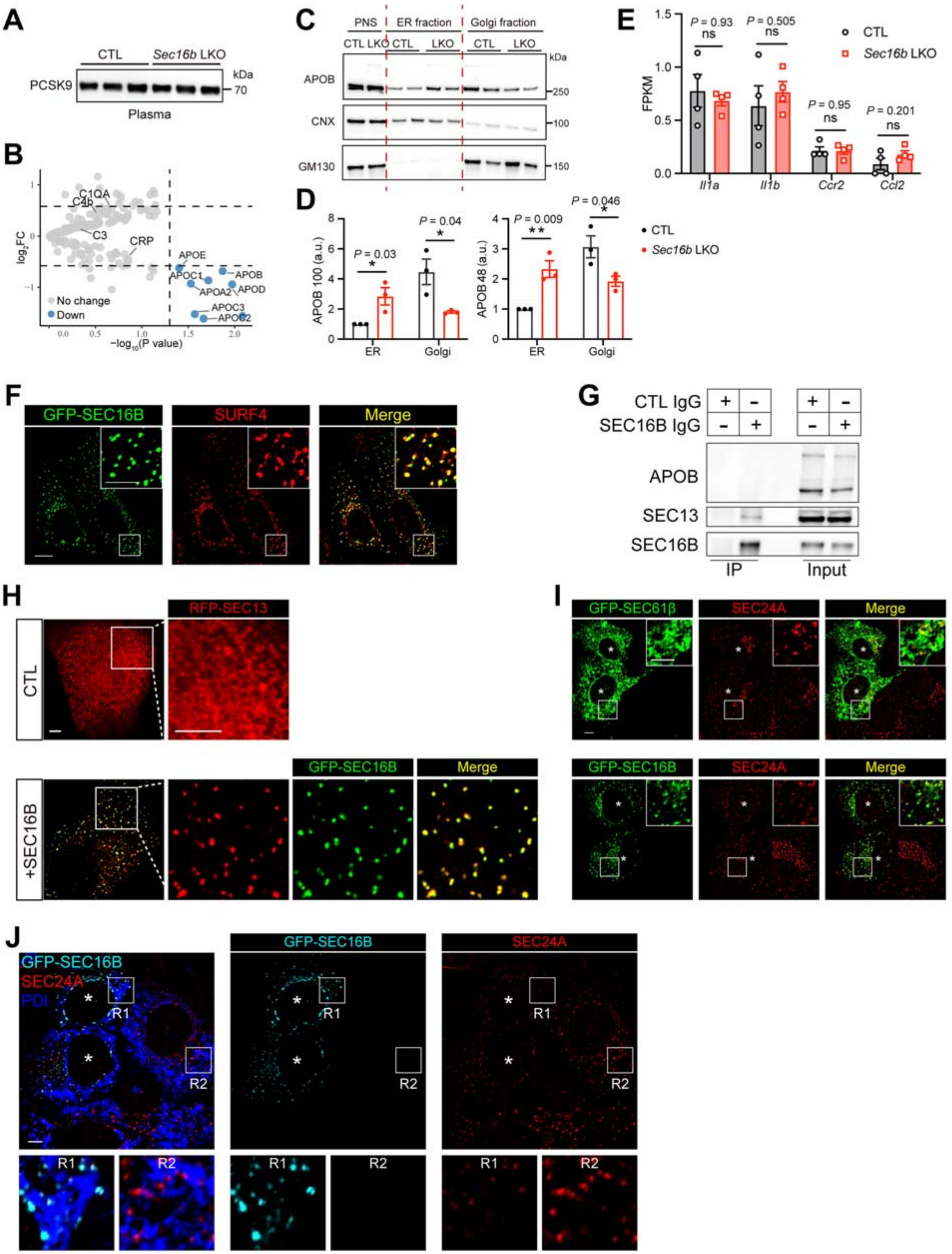
A
CTL
Sec16b LKO
kDa
PCSK9
70
Plasma
B
C1QA
C4b
C3
CRP
APOE
APOC1
APOB
APOA2
APOD
APOC3
APOC2
No change
Down
log2FC
−log10(P value)
C
PNS
ER fraction
Golgi fraction
CTL LKO CTL LKO CTL LKO
kDa
APOB
250
CNX
100
GM130
150
D
APOB 100 (a.u.)
APOB 48 (a.u.)
P = 0.03
P = 0.04
P = 0.009
P = 0.046
ER
Golgi
CTL
Sec16b LKO
E
FPKM
P = 0.93
P = 0.505
P = 0.95
P = 0.201
ns
CTL
Sec16b LKO
Il1a
Il1b
Ccr2
Ccl2
F
GFP-SEC16B
SURF4
Merge
G
CTL IgG
SEC16B IgG
APOB
SEC13
SEC16B
IP
Input
H
RFP-SEC13
CTL
+SEC16B
GFP-SEC16B
Merge
I
GFP-SEC61β
SEC24A
Merge
GFP-SEC16B
SEC24A
Merge
J
GFP-SEC16B
SEC24A
PDI
R1
R2

Figure EV2. SEC16B selectively regulates hepatic lipid secretion.

(**A**) IB analysis of PCSK9 in plasma samples from CTL and *Sec16b* LKO mice. Representative of three independent experiments is shown. (**B**) Volcano plot of quantitative proteomics in plasma samples from CTL and *Sec16b* LKO mice. Significantly altered proteins with |FC | > 1.5 and adjusted *P* value < 0.05 are colored in blue. $n = 3$ for each genotype. (**C**) IB analysis of ER-enriched fractions and Golgi-enriched fractions isolated from CTL and *Sec16b*-KO mouse livers. Representative of three independent experiments is shown. (**D**) Quantification of IB signals in (**C**). $n = 3$ biological independent replicates for each group. Data are shown as mean ± SEM. **P* < 0.05; ***P* < 0.01 (two-tailed Student's *t* test). (**E**) Analysis of inflammatory gene expression using hepatic RNA-seq data from Fig. 3G with CTL and *Sec16b* LKO mice under fasting conditions. $n = 4$ mice for each group. Statistic was analyzed using the Wald test in DESeq2 with adjustment by Benjamini-Hochberg. Data are shown as mean ± SEM. ns, no significance. (**F**) Co-localization of SEC16B with SURF4. Huh7 cells transfected with GFP-SEC16B were fixed and stained with an anti-SURF4 (red) antibody, prior to confocal microscopy. Scale bars = 5 µm. (**G**) Co-IP of endogenous SEC16B and SEC13 in mouse liver samples. Liver lysates were subjected to anti-SEC16B or CTL IgG IP, followed by SDS-PAGE and IB with the indicated antibodies. (**H**) SEC16B promotes membrane localization of outer coat proteins. Huh7 cells transfected with HA-SEC31A and RFP-SEC13, plus empty vector (upper row) or GFP-SEC16B WT (lower row) were analyzed by confocal microscopy. Scale bars = 5 µm. (**I**) SEC61β overexpression exhibits little effect on COPII coalescence compared to neighboring cells. Huh7 cells transfected with GFP-SEC61β (upper) or GFP-SEC16B (lower) were fixed and stained with an anti-SEC24A (red) antibody, prior to confocal microscopy. Scale bars = 5 µm. Asterisks, positive cells with GFP-SEC61β or GFP-SEC16B expression. (**J**) Similar localization on the ER surface of SEC24A between SEC16B-expressing and neighboring control cells. Huh7 cells transfected with GFP-SEC16B were fixed and co-stained with anti-SEC24A (red) and anti-PDI (blue) antibodies, prior to confocal microscopy. Scale bars = 5 µm. Asterisks, positive cells with GFP-SEC16B expression. Source data are available online for this figure.

 © The Author(s)

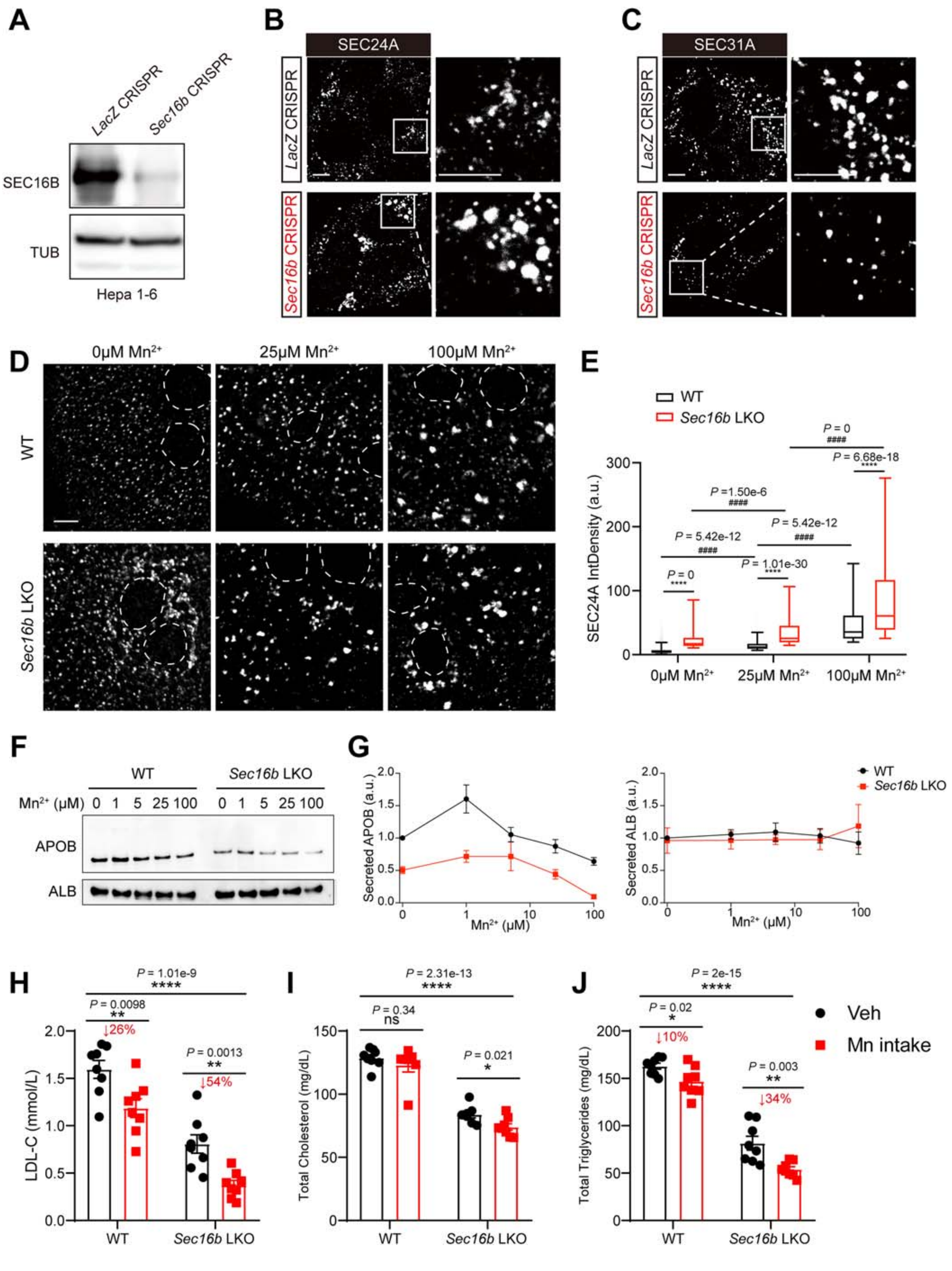
A
LacZ CRISPR
Sec16b CRISPR
SEC16B
TUB
Hepa 1-6
B
SEC24A
LacZ CRISPR
Sec16b CRISPR
C
SEC31A
LacZ CRISPR
Sec16b CRISPR
D
0μM Mn2+
25μM Mn2+
100μM Mn2+
WT
Sec16b LKO
E
WT
Sec16b LKO
SEC24A IntDensity (a.u.)
P = 0
P = 6.68e-18
P =1.50e-6
P = 5.42e-12
P = 5.42e-12
P = 1.01e-30
0μM Mn2+
25μM Mn2+
100μM Mn2+
F
WT
Sec16b LKO
Mn2+ (μM) 0 1 5 25 100 0 1 5 25 100
APOB
ALB
G
Secreted APOB (a.u.)
Secreted ALB (a.u.)
Mn2+ (μM)
WT
Sec16b LKO
H
P = 1.01e-9
P = 0.0098
↓26%
P = 0.0013
↓54%
LDL-C (mmol/L)
WT
Sec16b LKO
I
P = 2.31e-13
P = 0.34
ns
P = 0.021
Total Cholesterol (mg/dL)
J
P = 2e-15
P = 0.02
↓10%
P = 0.003
↓34%
Total Triglycerides (mg/dL)
Veh
Mn intake

Figure EV3. A cooperative effect between SEC16B loss and Mn^{2+} signals on COPII coalescence and lipid secretion.

(**A**) IB analysis of SEC16B protein depletion in Hepa 1-6 with CRISPR-mediated gene editing. (**B**) Loss of SEC16B enhances SEC24A coalescence in Hepa 1-6. CTL (*LacZ* CRISPR) or *Sec16b* KO (*Sec16b* CRISPR) Hepa 1-6 cells were stained with the anti-SEC24A antibody prior to confocal microscopy. Scale bars = 5 μm. (**C**) Loss of SEC16B decreases SEC31A signals in puncta. The same set of cells as in (**B**) were stained with the anti-SEC31A antibody prior to confocal microscopy. Scale bars = 5 μm. (**D**) SEC16B loss enhances the promoting effect of Mn^{2+} signals on COPII coalescence. Primary hepatocytes isolated from control (WT) or *Sec16b* KO mice were treated with $MnCl_2$, prior to SEC24A staining. Dashed lines: nucleus. Scale bars = 5 μm. (**E**) Quantification of the integrated fluorescence signal density of SEC24A puncta in (**D**). Data are shown as a box plot with 5–95% distribution, with the line inside the box and box edges representing median and the interquartile ranges, respectively. $n = 2615$, 747, 896, 512, 425, and 271 puncta for WT, *Sec16b* KO, WT with 25 μM $MnCl_2$ treatment, *Sec16b* KO with 25 μM $MnCl_2$ treatment, WT with 100 μM $MnCl_2$ treatment, and *Sec16b KO* with 100 μM $MnCl_2$ treatment, respectively. Data were pooled from 10 cells per condition. Statistical analysis between WT and *Sec16b* KO under the same $MnCl_2$ treatment was determined by two-tailed Student's *t* test. ****$P < 0.0001$. Statistical analysis among different $MnCl_2$ treatments within the same genotype was determined by ANOVA with the Tukey post hoc test. $^{\#\#\#\#}P < 0.0001$. a.u. arbitrary units. (**F**) Cooperative regulation of SEC16B loss and Mn^{2+} signals in APOB secretion. Primary hepatocytes isolated from WT or *Sec16b* LKO mice were treated with TPEN for 30 min, followed by $MnCl_2$ treatment for 8 h with indicated doses. APOB and albumin in the medium were analyzed by IB. Representative of three independent experiments is shown. (**G**) Quantification of IB signals in (**F**). The IB signals were normalized to the secretion level of WT at 0 μM $MnCl_2$ treatment. $n = 3$ biologically independent replicates for each genotype at each treatment. Data are shown as mean ± SEM. a.u. arbitrary units. (**H–J**) Cooperative effects between SEC16B loss and Mn^{2+} signals in lowering plasma lipids. Total LDL-C (**H**), total cholesterol (**I**) and triglyceride (**J**) levels in plasma of WT and *Sec16b* LKO mice with vehicle or $MnCl_2$ intake were measured. $n = 8$ for each group. Data are shown as mean ± SEM. *$P < 0.05$; **$P < 0.01$; ****$P < 0.0001$. ns no significance (two-tailed Student's *t* test). Source data are available online for this figure.

© The Author(s)

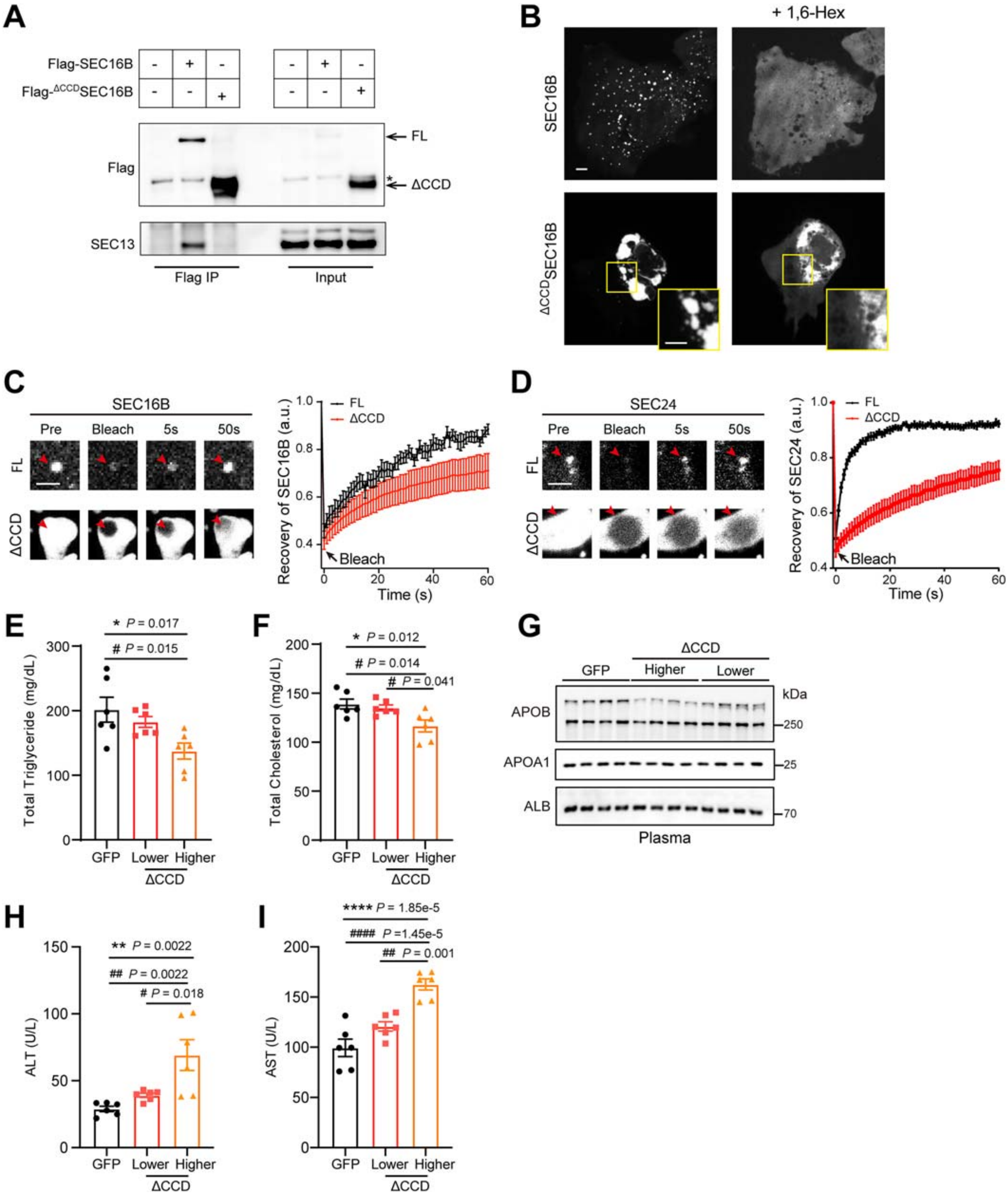
A
Flag-SEC16B
Flag-ΔCCDSEC16B
Flag
FL
ΔCCD
SEC13
Flag IP
Input
B
+ 1,6-Hex
SEC16B
ΔCCDSEC16B
C
SEC16B
Pre Bleach 5s 50s
FL
ΔCCD
Recovery of SEC16B (a.u.)
Bleach
Time (s)
D
SEC24
Pre Bleach 5s 50s
Recovery of SEC24 (a.u.)
E
* P = 0.017
# P = 0.015
Total Triglyceride (mg/dL)
GFP Lower Higher
ΔCCD
F
* P = 0.012
# P = 0.014
# P = 0.041
Total Cholesterol (mg/dL)
G
GFP Higher Lower
APOB
APOA1
ALB
kDa
250
25
70
Plasma
H
** P = 0.0022
P = 0.0022
# P = 0.018
ALT (U/L)
I
**** P = 1.85e-5
P =1.45e-5
P = 0.001
AST (U/L)

Figure EV4. Abnormal COPII assembly impairs secretion.

(**A**) SEC16B lacking CCD domain ($^{\Delta CCD}$SEC16B) fails to interact with SEC13. Mouse livers expressing Flag-SEC16B or Flag-$^{\Delta CCD}$SEC16B were lysed and subjected to anti-Flag Co-IP, followed by SDS-PAGE and IB with the indicated antibodies. (**B**) Confocal image of Huh7 cells transfected with GFP-SEC16B or GFP-$^{\Delta CCD}$SEC16B before or after 1,6-Hex treatments for 30 s. Scale bars = 5 µm. (**C**) FRAP comparison of GFP-SEC16B coalescence and $^{\Delta CCD}$SEC16B over-aggregates in Huh7 cells. Arrowheads: selected foci for photobleaching in each condition. Scale bars = 2.5 µm. Right, quantification analysis, shown as mean ± SEM. $n = 9$ for GFP-SEC16B coalescence and 7 for GFP-$^{\Delta CCD}$SEC16B aggregates, respectively. (**D**) FRAP comparison of RFP-SEC24D colocalized with GFP-SEC16B coalescence or $^{\Delta CCD}$SEC16B over-aggregates in Huh7 cells. Arrowheads: selected foci for photobleaching in each condition. Scale bars = 2.5 µm. Right, quantification analysis, shown as mean ± SEM. $n = 11$ and 9 for RFP-SEC24D colocalized with GFP-SEC16B coalescence or $^{\Delta CCD}$SEC16B aggregates, respectively. (**E**) Plasma triglyceride levels of mice receiving AAV-TBG-GFP or AAV-TBG-$^{\Delta CCD}$SEC16B. Data are shown as mean ± SEM. $n = 6$ mice for each group. $^{*}P < 0.05$ determined by ANOVA; $^{\#}P < 0.05$ determined by the Tukey post hoc test. (**F**) Plasma total cholesterol levels of mice in (**E**). Data are shown as mean ± SEM. $^{*}P < 0.05$ determined by ANOVA; $^{\#}P < 0.05$ determined by the Tukey post hoc test. (**G**) IB analysis of plasma samples from mice in (**E**). Representative of three independent experiments is shown. (**H**) Plasma ALT levels of mice in (**E**). Data are shown as mean ± SEM. $^{**}P < 0.01$ determined by ANOVA; #, $P < 0.05$; $^{\#\#}P < 0.01$ determined by the Tukey post hoc test. (**I**) Plasma AST levels of mice in (**E**). Data are shown as mean ± SEM. $^{****}P < 0.0001$ determined by ANOVA; $^{\#\#}P < 0.01$; $^{\#\#\#\#}P < 0.0001$ determined by the Tukey post hoc test. Source data are available online for this figure.

 © The Author(s)

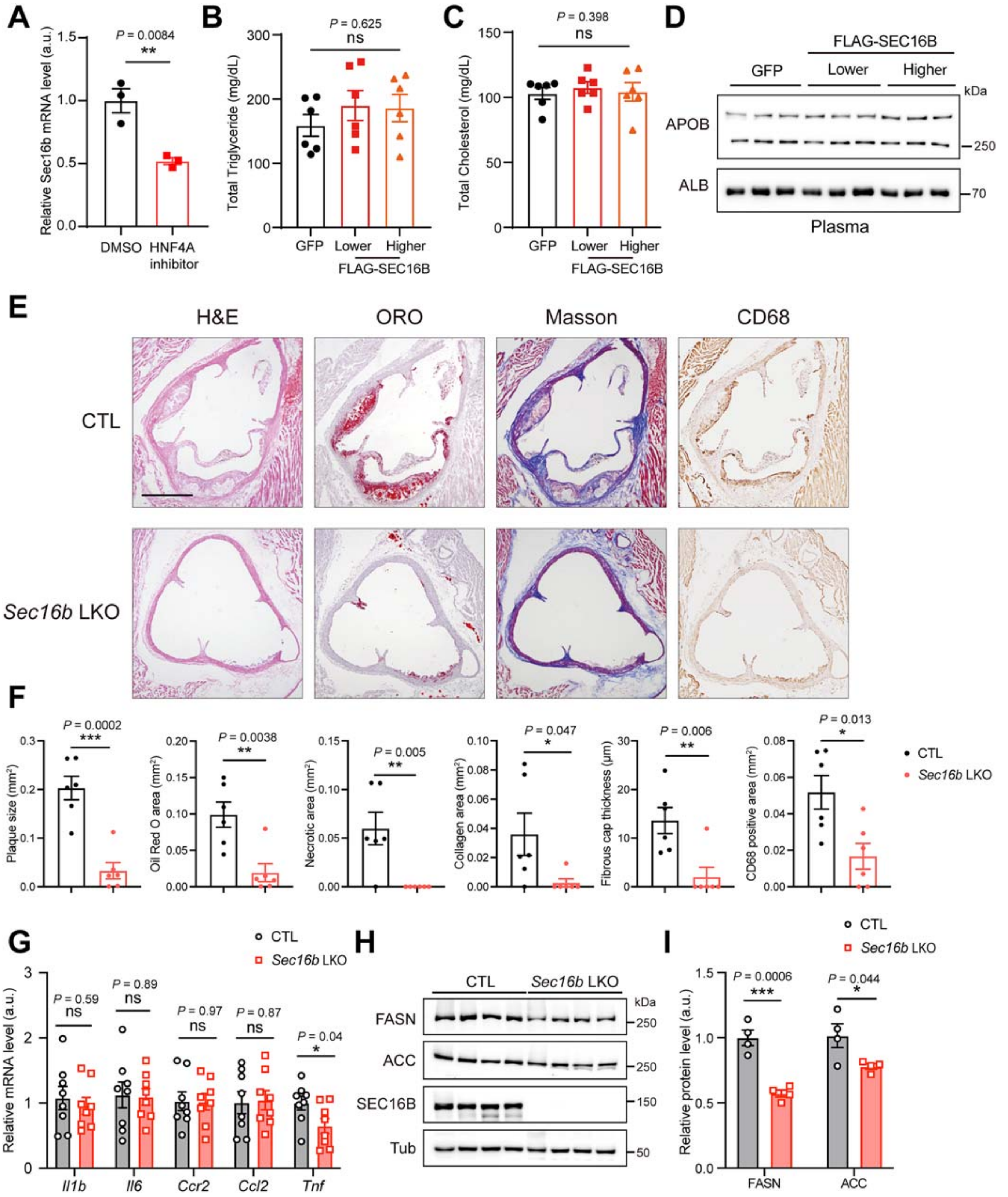
A
Relative Sec16b mRNA level (a.u.)
P = 0.0084
**
DMSO
HNF4A inhibitor
B
Total Triglyceride (mg/dL)
P = 0.625
ns
GFP
Lower
Higher
FLAG-SEC16B
C
Total Cholesterol (mg/dL)
P = 0.398
ns
D
FLAG-SEC16B
GFP
Lower
Higher
kDa
APOB
ALB
Plasma
E
H&E
ORO
Masson
CD68
CTL
Sec16b LKO
F
Plaque size (mm²)
P = 0.0002
***
Oil Red O area (mm²)
P = 0.0038
**
Necrotic area (mm²)
P = 0.005
**
Collagen area (mm²)
P = 0.047
*
Fibrous cap thickness (μm)
P = 0.006
**
CD68 positive area (mm²)
P = 0.013
*
CTL
Sec16b LKO
G
Relative mRNA level (a.u.)
P = 0.59
P = 0.89
P = 0.97
P = 0.87
P = 0.04
Il1b
Il6
Ccr2
Ccl2
Tnf
H
CTL
Sec16b LKO
FASN
ACC
SEC16B
Tub
I
Relative protein level (a.u.)
P = 0.0006
***
P = 0.044
*
FASN
ACC

Figure EV5. Therapeutic potentials of SEC16B targeting in dyslipidemia and ASCVD.

(**A**) Relative *Sec16b* mRNA levels in murine primary hepatocytes treated with DMSO control, or BI6015, a HNF4A inhibitor, as measured by reverse transcription-quantitative PCR (RT-qPCR). Data are shown as mean ± SEM. $n = 3$ biological replicates. *P* values were determined by two-tailed Student's *t* test. (**B**) Plasma triglyceride levels of mice receiving AAV-TBG-GFP or AAV-TBG-SEC16B. $n = 6$ mice for each group. Data are shown as mean ± SEM. ns, no significance determined by ANOVA test. (**C**) Plasma total cholesterol levels of mice in (**B**). Data are shown as mean ± SEM. $n = 6$ mice for each group. ns, no significance determined by the ANOVA test. (**D**) IB analysis of plasma samples from mice in (**B**). Representative of three independent experiments is shown. (**E**) Representative of H&E, ORO, Masson and CD68 IHC at aortic root cross-sections from mice in Fig. 7A. Scale bars = 100 µm. (**F**) Quantification of plaque size, ORO positive area, necrotic area, collagen area, fibrous cap and CD68 positive are using aortic root sections in (**E**). Data are shown as mean ± SEM. $n = 6$ mice for each group. *$P < 0.05$; ****$P < 0.0001$ (two-tailed Student's *t* test). (**G**) Relative expression levels of hepatic inflammatory genes in mice from Fig. 7A. Hepatic mRNA from the indicated mouse liver samples were subjected to RT-qPCR using primers as listed in Dataset EV1. $n = 8$ mice for each group. *P* values were determined by two-tailed Student's *t* test. (**H**) IB analysis of liver samples from mice in Fig. 7A. (**I**) Quantification of IB signals in (**H**). Data are shown as mean ± SEM. $n = 4$ mice for each group. *$P < 0.05$; **$P < 0.01$ (two-tailed Student's *t* test). Source data are available online for this figure.

© The Author(s)
